# Mapping of variations in child stunting, wasting and underweight within the states of India: the Global Burden of Disease Study 2000–2017

**DOI:** 10.1016/j.eclinm.2020.100317

**Published:** 2020-05-13

**Authors:** India State-Level Disease Burden Initiative CGF Collaborators, Rajkumar Hemalatha, Anamika Pandey, Damaris Kinyoki, Siddarth Ramji, Rakesh Lodha, G. Anil Kumar, Nicholas J. Kassebaum, Elaine Borghi, Deepti Agrawal, Subodh S. Gupta, Avula Laxmaiah, Anita Kar, Matthews Mathai, Chris M. Varghese, Shally Awasthi, Priyanka G. Bansal, Joy K. Chakma, Michael Collison, Supriya Dwivedi, Mahaveer J. Golechha, Zaozianlungliu Gonmei, Suparna G. Jerath, Rajni Kant, Ajay K. Khera, Rinu P. Krishnankutty, Anura V. Kurpad, Laishram Ladusingh, Ridhima Malhotra, Raja S. Mamidi, Helena Manguerra, Joseph L. Mathew, Parul Mutreja, Arlappa Nimmathota, Ashalata Pati, Manorama Purwar, Kankipati V. Radhakrishna, Neena Raina, Mari J. Sankar, Deepika S. Saraf, Megan Schipp, R.S. Sharma, Chander Shekhar, Anju Sinha, V. Sreenivas, K. Srinath Reddy, Hendrik J. Bekedam, Soumya Swaminathan, Stephen S. Lim, Rakhi Dandona, Christopher J.L. Murray, Simon I. Hay, G.S. Toteja, Lalit Dandona

**Affiliations:** a National Institute of Nutrition, Indian Council of Medical Research, Hyderabad, India; b Public Health Foundation of India, Gurugram, India; c Institute for Health Metrics and Evaluation, University of Washington, Seattle, USA; d Department of Paediatrics, Maulana Azad Medical College, New Delhi, India; e Department of Paediatrics, All India Institute of Medical Sciences, New Delhi, India; f Department of Biostatistics, All India Institute of Medical Sciences, New Delhi, India; g World Health Organisation, Geneva, Switzerland; h WHO India Country Office, New Delhi, India; i Department of Community Medicine, Mahatma Gandhi Institute of Medical Sciences, Wardha, India; j School of Health Sciences, Savitribai Phule Pune University, Pune, India; k Centre for Maternal and Newborn Health, Liverpool School of Tropical Medicine, Liverpool, UK; l Department of Pediatrics, King George's Medical University, Lucknow, India; m Indian Council of Medical Research, New Delhi, India; n Indian Institute of Public Health – Gandhinagar, Public Health Foundation of India, Gandhinagar, India; o Indian Institute of Public Health – Delhi, Public Health Foundation of India, Gurugram, India; p Ministry of Health and Family Welfare, Government of India, New Delhi, India; q Department of Physiology and Nutrition, St John's Medical College, Bengaluru, India; r Bodoland University, Kokrajhar, India; s Advanced Paediatrics Centre, Post Graduate Institute of Medical Education and Research, Chandigarh, India; t Nagpur INTERGROWTH-21st Research Centre, Ketkar Hospital, Nagpur, India; u Regional Office for South-East Asia, World Health Organization, New Delhi, India

**Keywords:** Child growth failure, District-level, Geospatial mapping, Inequality, National Nutrition Mission, Prevalence, Stunting, Time trends, Under-five, Undernutrition, Underweight, Wasting, WHO/UNICEF targets

## Abstract

**Background:**

To inform actions at the district level under the National Nutrition Mission (NNM), we assessed the prevalence trends of child growth failure (CGF) indicators for all districts in India and inequality between districts within the states.

**Methods:**

We assessed the trends of CGF indicators (stunting, wasting and underweight) from 2000 to 2017 across the districts of India, aggregated from 5 × 5 km grid estimates, using all accessible data from various surveys with subnational geographical information. The states were categorised into three groups using their Socio-demographic Index (SDI) levels calculated as part of the Global Burden of Disease Study based on per capita income, mean education and fertility rate in women younger than 25 years. Inequality between districts within the states was assessed using coefficient of variation (CV). We projected the prevalence of CGF indicators for the districts up to 2030 based on the trends from 2000 to 2017 to compare with the NNM 2022 targets for stunting and underweight, and the WHO/UNICEF 2030 targets for stunting and wasting. We assessed Pearson correlation coefficient between two major national surveys for district-level estimates of CGF indicators in the states.

**Findings:**

The prevalence of stunting ranged 3.8-fold from 16.4% (95% UI 15.2–17.8) to 62.8% (95% UI 61.5–64.0) among the 723 districts of India in 2017, wasting ranged 5.4-fold from 5.5% (95% UI 5.1–6.1) to 30.0% (95% UI 28.2–31.8), and underweight ranged 4.6-fold from 11.0% (95% UI 10.5–11.9) to 51.0% (95% UI 49.9–52.1). 36.1% of the districts in India had stunting prevalence 40% or more, with 67.0% districts in the low SDI states group and only 1.1% districts in the high SDI states with this level of stunting. The prevalence of stunting declined significantly from 2010 to 2017 in 98.5% of the districts with a maximum decline of 41.2% (95% UI 40.3–42.5), wasting in 61.3% with a maximum decline of 44.0% (95% UI 42.3–46.7), and underweight in 95.0% with a maximum decline of 53.9% (95% UI 52.8–55.4). The CV varied 7.4-fold for stunting, 12.2-fold for wasting, and 8.6-fold for underweight between the states in 2017; the CV increased for stunting in 28 out of 31 states, for wasting in 16 states, and for underweight in 20 states from 2000 to 2017. In order to reach the NNM 2022 targets for stunting and underweight individually, 82.6% and 98.5% of the districts in India would need a rate of improvement higher than they had up to 2017, respectively. To achieve the WHO/UNICEF 2030 target for wasting, all districts in India would need a rate of improvement higher than they had up to 2017. The correlation between the two national surveys for district-level estimates was poor, with Pearson correlation coefficient of 0.7 only in Odisha and four small north-eastern states out of the 27 states covered by these surveys.

**Interpretation:**

CGF indicators have improved in India, but there are substantial variations between the districts in their magnitude and rate of decline, and the inequality between districts has increased in a large proportion of the states. The poor correlation between the national surveys for CGF estimates highlights the need to standardise collection of anthropometric data in India. The district-level trends in this report provide a useful reference for targeting the efforts under NNM to reduce CGF across India and meet the Indian and global targets.

**Research in context**

**Evidence before this study**

We searched PubMed for published literature on CGF in India, Google for reports in the public domain, and references in these papers and reports, using the search terms “child growth failure”, child malnutrition”, “district-level”, “epidemiology”, “geospatial”, “geospatial mapping”, “India”, “inequality”, “national nutrition mission”, “prevalence”, “under-five”, “subnational”, “stunting”, “trends”, “undernutrition”, “underweight”, and “wasting” on August 5, 2019, without language or publication date restrictions. We found only a few previous studies that have reported district-level variations in CGF in India, using a single data source. Comprehensive mapping of the variations between districts in the prevalence of stunting, wasting and underweight, and their progress towards achieving the Indian and the global nutrition targets, using all accessible data sources in a single framework has not been compiled to inform action under the district-focused approach of the National Nutrition Mission (NNM).

**Added value of this study**

This study provides the first comprehensive analysis of district-level prevalence of CGF indicators in India by aggregating the best possible estimates at a resolution of 5 × 5 km grid, using all accessible geo-referenced survey data and gridded covariates from multiple sources. The findings highlight wide variations in prevalence, rate of reduction, and inequality between districts within the states. Majority of the districts in India would need a higher rate of improvement than they had up to 2017 to achieve the NNM 2022 and the WHO/UNICEF 2030 targets, with the findings pointing to the additional effort needed in each district. The identification of priority districts with high prevalence and low rates of reduction within each state is useful for policy makers. The findings in this report are timely as the Government of India is intensifying its efforts to accelerate the reduction in child malnutrition across the country through decentralised planning and implementation of targeted nutrition interventions at the district-level under NNM.

**Implications of all available evidence**

This granular analysis of the trends of CGF indicators from 2000 to 2017 for every district of India, and the relation of their progress to the efforts needed to achieve the India 2022 and the global 2030 targets, enables identification of districts in each state that have persistently high prevalence of stunting, wasting and underweight and low rates of improvement. Such fine-grain insights into the prevalence of CGF indicators for the 1.4 billion population of India are useful to inform decisions on policy and programmatic actions tailored precisely for each district to accelerate the progress in achieving better nutritional status and reducing inequalities within states and across the country.

Alt-text: Unlabelled box

## 1 Introduction

Child malnutrition is a major public health problem that has adverse short-term and long-term health effects. It is an important risk factor for death and disease globally [1], [2], [3], [4], [5], and often results in compromised cognitive development and physical capabilities, poor school performance, and low productivity [6,7]. Child growth failure (CGF), measured as stunting, wasting and underweight, is a subset of undernutrition characterised by insufficient height or weight against age-specific growth reference standards [8], [9], [10]. The international commitment to reduce and ultimately eliminate child malnutrition in all its forms was strengthened in 1990 with the World Declaration on the Survival, Protection and Development of Children, accelerated during the Millennium Development Goals era, and gained further momentum with the adoption of WHO global nutrition 2025 targets, the UN Sustainable Development Goals 2030, and the WHO/UNICEF 2030 nutrition targets [11], [12], [13], [14], [15].

The India State-Level Disease Burden Initiative has reported that over one-fifth of the under-5 deaths and disease burden in India can be attributed to CGF, and that there are wide variations in the prevalence of the CGF indicators across the states, ranging from 21.3 to 49.0% for stunting, 6.3 to 19.3% for wasting, and 16.5 to 42.2% for underweight in 2017 [16,17]. Variations are expected within the states as well, as many states have large population and the districts within the states often vary in terms of ecology, demography and economy, all of which affect child health. The National Nutrition Mission (NNM), also known as POSHAN Abhiyaan, launched in India in 2018 has emphasized targeting efforts at the district as well as sub-district levels to accelerate improvement in CGF and other indicators of malnutrition [18], [19], [20]. Some understanding of the sub-state level heterogeneity in the prevalence of CGF indicators and their correlates is available in India from previous reports that have used one round of the National Family Health Survey data [21], [22], [23], [24], [25]. However, there has been no comprehensive consolidation of the district-level trends of all three CGF indicators using all accessible data sources from India over a long period of time, which also relates the district-level trends with the targets set by NNM for 2022 and WHO/UNICEF for 2030.

To address this knowledge gap, in this article we report geospatial analysis of stunting, wasting and underweight in children under-five in India at the 5 × 5 km grid and district level from 2000 to 2017, and relate these trends to the NNM 2022 and WHO/UNICEF 2030 targets. This granular assessment could be useful for better targeting of efforts at sub-state levels to improve CGF across India.

## 2 Methods

### 2.1 Overview

The analysis and findings on CGF indicators presented in this report were produced by the India State-Level Disease Burden Initiative as part of the Global Burden of Diseases, Injuries, and Risk Factors Study (GBD) 2017. The work of this initiative has been approved by the Health Ministry Screening Committee of the Indian Council of Medical Research and the ethics committee of the Public Health Foundation of India. Detailed description of the metrics, data sources, and statistical modelling for CGF indicators at various geographic levels down to the 5 × 5 km grids has been reported elsewhere [5,16,26,27]. The methods relevant for this paper are summarised here and described in detail in the appendix (pp 3–21).

### 2.2 Estimation and mapping of CGF indicators

All accessible data sources from India were utilised to estimate the prevalence of stunting, wasting and underweight at the state level in GBD 2017, which included national household surveys, a variety of dietary and nutrition surveys, and other epidemiological studies (Appendix pp 22–26) [5,16]. A three-step modelling process was used which integrated multiple data inputs and borrowed information across age, time and location to produce the best possible estimates of the time trends of the CGF indicators at the state level. In the first step, an ensemble modelling technique was used to find an optimal distribution of CGF indicators by fitting a variety of distributions to the available microdata. In the second step, mean z-scores and the prevalence of CGF indicators obtained by collapsing individual-level microdata were combined with the tabulated data and then modelled using spatiotemporal Gaussian process regression to generate estimates of CGF indicators for each location, year, age, and sex. In the final step, a probability density functions of the distribution of z-scores for each location, year, age, and sex, estimated by combining mean z-scores and prevalence with ensemble weights in an optimisation framework followed by the method of moments, were integrated to determine the prevalence of CGF indicators. Stunting, wasting and underweight were defined as height-for-age, weight-for-height and weight-for-age below two standard deviations of the median in the WHO 2006 standard curves, respectively [28].

The prevalence of stunting, wasting and underweight was estimated for each year from 2000 to 2017 at a spatial resolution of a 0·042° × 0·042° grid cells over the globe, which is 5 × 5 km at the equator [26,27]. The details of this method are given in the appendix (pp 3–21). Data on individual-level height, weight and age for children under-five were extracted from large-scale national household surveys such as the National Family Health Surveys, District Level Household Surveys, National Nutrition Surveys, and other surveys in India (Appendix pp 22–26). All the extracted data for the estimation at 5 × 5 km grids were georeferenced to either global positioning system (GPS) location points or the smallest possible administrative units (polygons) in the absence of GPS coordinates. The administrative unit data were converted to points spread across the corresponding administrative division according to a resampling algorithm that accounted for population distribution. The combined dataset consisting of geo-referenced points and converted points provided the number of children and sample size for a particular location by age and time period. Boundary information for these administrative units for the year 2018 was obtained as shape files from the ML Infomap (https://www.mlinfomap.com/).

Based on geo-referenced survey data and gridded covariates over space and time, a stacked generalisation ensemble model was first implemented to capture the possible non-linear effects and complex interactions between covariates [26,27,29]. Several socioeconomic and environmental covariates at 5 × 5 km grid level were used across space and time in the first stage of initial model fits to strengthen the predictive estimates. The covariates were selected on the basis of their expected predictive power for each CGF indicator as determined by a review of available evidence in the literature and are listed in appendix (p 11).

Three machine learning sub-models were fitted to the dataset using covariate data as explanatory predictors: general additive model, boosted regression trees and lasso regression. The predictions from each of these sub-models were then used as covariates in the combined model to produce the final estimates (Appendix pp 10–18). In this combined model, binomial count data were fit in a Bayesian hierarchical modelling framework using a spatially and temporally explicit generalised linear regression model with logit link function. From the fitted posterior distribution of this combined model, 1000 draws were taken, which were combined and processed into 1000 candidate 5 × 5 km resolution maps. These 5 × 5 km gridded candidate maps were aggregated up to district, state and country levels, using a state-level calibration factor in order to harmonize the geospatial estimates with the GBD state- and country-level estimates for India [16]. These candidate maps were summarised using the mean estimates and bounds of the 95% uncertainty intervals.

### 2.3 Projection of CGF indicators to 2030

The trends of stunting, wasting and underweight from 1990 to 2017 were used to project their prevalence to 2030 for every state of India as part of GBD, giving higher weight to the more recent annual rate of change to project from 2018 to 2030 [16,30]. To project prevalence at 5 × 5 km grids up to 2030, the annual rate of change from 2000 to 2017 was applied to obtain estimates for subsequent years, using a projection methodology that has been used previously for such geospatial analyses [26]. Across 1000 draws, a logit-transformed annual rate of change from 2000 to 2017 was calculated at each pixel (5 × 5 km unit) for the CGF indicators, and was then applied to the final 2017 pixel estimates to generate the projected estimates up to 2030. Population-weighted aggregations of prevalence at the district levels were calculated from the pixel draws, which were then harmonized with the national and state level GBD projected prevalence by applying the relevant scaling factor. These methods are described in the appendix (pp 18–19) and elsewhere [5,16,26,27].

### 2.4 Analysis presented in this paper

We report prevalence of stunting, wasting and underweight per 100 under-five children and their trends from 2000 to 2017 at the 5 × 5 km grid levels across India and for the 723 districts. We estimate the change in these indicators at the draw level over time, highlighting the more recent changes from 2010 to 2017. We report inequality in the prevalence of stunting, wasting and underweight between districts within each state using coefficient of variation (CV), defined as the ratio of standard deviation to the mean of distribution of prevalence among the districts within a state expressed as percentage. We used CV as it is a simple metric of the relative spread of a distribution. We also assessed how the CV of stunting, wasting and underweight changed over time in each state. We present results based on Socio-demographic Index (SDI) by district in three groups of states. SDI is a composite indicator of development status, which ranges from 0 to 1, and is a geometric mean of the values of the indices of lag-distributed per capita income, mean education for those 15 years of age or older, and fertility rate among women younger than 25 years. The states were grouped on the basis of their SDI as calculated by GBD in 2017: low SDI (≤0.53), middle SDI (0.54–0.60), and high SDI (>0.60, Appendix p 27) [16,31,32]. For the districts created after the year 2000, geolocated data were used to arrive at estimates for these districts prior to their creation.

We present detailed analyses of trends in three states to demonstrate how differences in the magnitude of stunting, wasting and underweight and their rates of reduction from 2000 to 2017 can help identify districts that need higher priority for CGF reduction. The tertiles of prevalence in 2017 for each indicator and the tertiles of their annual rate of reduction from 2010 to 2017 were calculated to categorise districts into one of nine categories for both the state and national distributions. These categories were created by crossing high, medium and low prevalence with low, medium and high annual rates of reduction in a 3 × 3 table.

We projected the prevalence of stunting, wasting and underweight for each district up to 2030 based on the trends from 2000 to 2017 and compared these with the NNM 2022 and the WHO/UNICEF 2030 targets to highlight the rate of improvement needed in each district to individually achieve the targets. The NNM has set a target of stunting prevalence of 25.0% in 2022 and a 2 percentage points reduction annually from 2017 to 2022 for underweight [18,20]. The WHO/UNICEF 2030 targets are a 50.0% reduction from 2012 to 2030 in the number of children under-five who are stunted and a prevalence of less than 3.0% for wasting in 2030 [15]. For consistency with other indicators, we estimated relative reductions in the prevalence of stunting instead of absolute numbers, as all other targets are based on prevalence [16]. Similarly, while the NNM 2022 target for stunting and underweight is for children 0–6 years, for consistency with the WHO/UNICEF 2030 targets we estimated these for children under-five years. We applied these targets to each district of India, and computed the gap between the projected prevalence of stunting and underweight in 2022 with the NNM 2022 targets in each district of India. Similarly, we computed the gap in 2030 for the WHO/UNICEF targets for stunting and wasting.

We assessed the Pearson correlation coefficient for the district-level estimates of stunting, wasting and underweight between the National Family Health Survey-4 (NFHS-4, 2015–2016) and the two complementary nationally representative household surveys (District-Level Household Survey [DLHS-4, 2012–2014] and Annual Health Survey [AHS, 2014]) for the 27 states covered by these surveys [33], [34], [35]. DLHS-4 was conducted in states other than the nine states covered by AHS, which included Assam, Bihar, Chhattisgarh, Jharkhand, Madhya Pradesh, Odisha, Rajasthan, Uttar Pradesh, and Uttarakhand.

All estimates are reported with 95% uncertainty intervals (UIs) where relevant, and were based on 1000 draws for each estimate, with the mean taken as the point estimate and the 2·5th and 97·5th percentiles as the 95% UI (Appendix p 20) [5]. Statistically significant change was defined as the 95% UIs of the change not overlapping zero.

### 2.5 Role of the funding source

Some of the contributors to this paper work with the Indian Council of Medical Research. The other funder, the Bill & Melinda Gates Foundation, of the study had no role in the study design, data collection, data analysis, data interpretation, or writing of this paper. The corresponding author had full access to all of the data in the study and had final responsibility for the decision to submit for publication.

## 3 Results

### 3.1 District-level variations

The prevalence of stunting in India decreased from 55.8% (95% UI 54.5–57.0) in 2000 to 47.3% (95% UI 46.7–47.9) in 2010 and 39.3% (95% UI 38.7–40.1) in 2017 (Fig. 1 and Appendix pp 28–35). This prevalence varied 3.8-fold between the districts in 2017, ranging from 16.4% (95% UI 15.2–17.8) to 62.8% (95% UI 61.5–64.0). The stunting prevalence was more than 40% in 261 (36.1%), 30–40% in 309 (42.7%), and less than 30% in 153 (21.2%) of the 723 districts. 209 (67%) of the 312 districts in the low SDI states, 50 (21.4%) of the 234 districts in the middle SDI states, and 2 (1.1%) of the 177 districts in the high SDI states had prevalence more than 40%. From 2010 to 2017, the decline in stunting prevalence was statistically significant in 712 (98.5%) districts with a maximum decline of 41.2% (95% UI 40.3–42.5; Appendix pp 28–35). This reduction was more than 30% in 54 (7.5%), 20–30% in 258 (35.7%), and less than 20% in 400 (55.3%) districts. A higher proportion of the districts in the low SDI states (66.7%) had a reduction of less than 20% in stunting prevalence from 2010 to 2017 compared with the middle (54.7%) and high SDI (46.3%) states. The median annual rate of reduction from 2010 to 2017 among the districts in the low SDI states was 2.35% (interquartile range [IQR] 1.67–3.31), 2.86% (IQR 2.10–3.78) in the middle SDI states, and 3.32% (IQR 2.46–3.92) in the high SDI states.

**Fig. 1:**
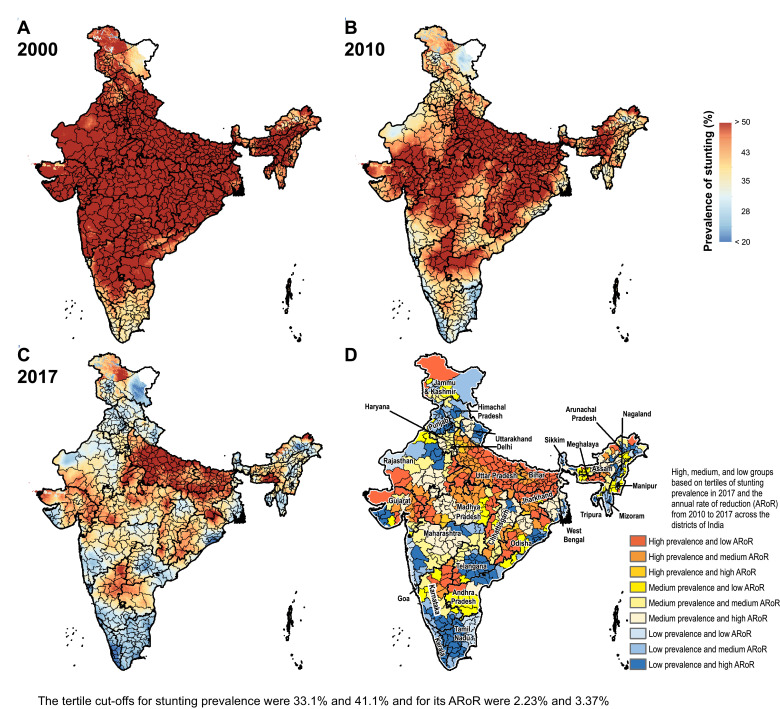
Stunting, wasting, and underweight mapping in India. (A) 2000, (B) 2010, and (C) 2017. (D) Tertile groupings of district-level prevalence in 2017 against the annual rate of reduction from 2010 to 2017.

**Fig. 1:**
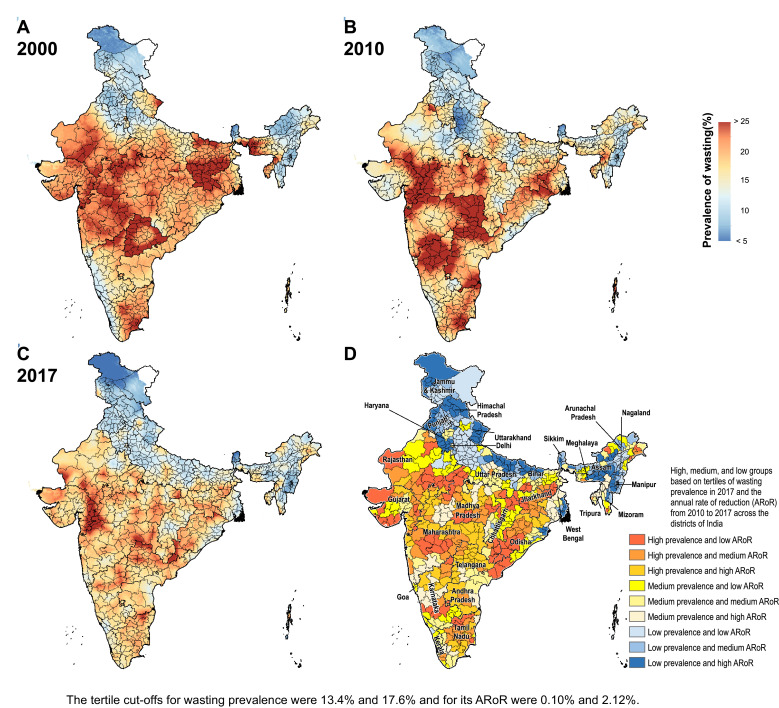

**Fig. 1:**
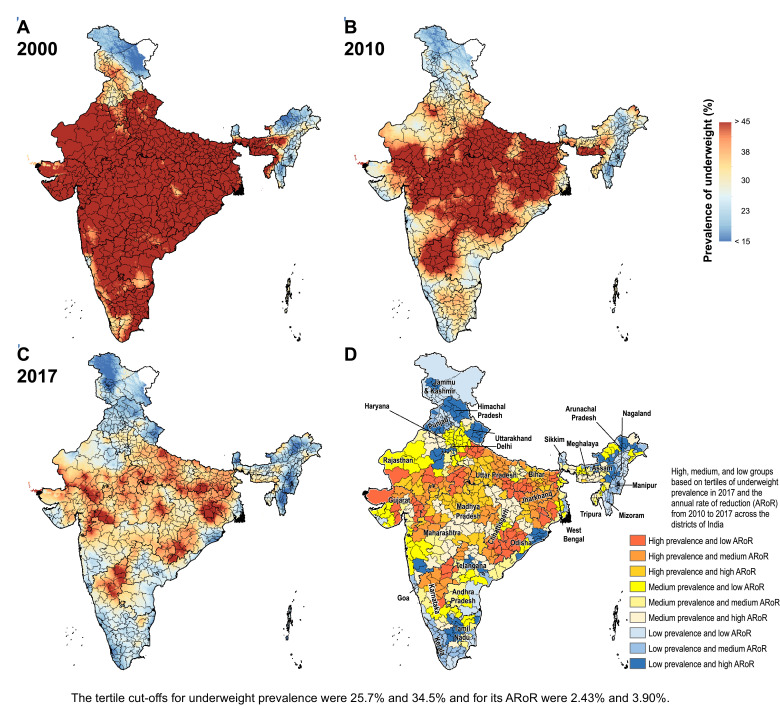

The prevalence of wasting decreased modestly from 19.2% (95% UI 18.9–19.6) in 2000 to 17.1% (95% UI 16.9–17.3) in 2010, and to 15.7% (95% UI 15.6–15.9) in 2017 in India (Fig. 1 and Appendix pp 36–43). This prevalence varied 5.4-fold between the districts in 2017 ranging from 5.5% (95% UI 5.1–6.1) to 30% (95% UI 28.2–31.8). The wasting prevalence was more than 15% in 398 (55%), 10–15% in 266 (36.8%), and less than 10% in 59 (8.2%) of the 723 districts. 184 (59%) of 312 districts in the low SDI states, 133 (56.6%) of 234 districts in the middle SDI states, and 81 (45.8%) of 177 districts in the high SDI states had wasting prevalence more than 15% in 2017. From 2010 to 2017 the wasting prevalence reduced significantly in 443 (61.3%) with a maximum decline of 44% (95% UI 42.3–46.7), increased significantly in 162 (22.4%) districts with a maximum increase of 3.8% (95% UI 0.3–6.5), and did not change significantly in 118 (16.3%) districts (Appendix 36–43). The reduction in wasting prevalence was less than 20% in 291 (40.2%), 20–30% in 112 (15.5%), and more than 30% in 40 (5.5%) of the 723 districts. A higher proportion of the districts in the middle (44%) and high (44.1%) SDI states had less than 20% reduction from 2010 to 2017 than in the low SDI states (35.3%). The median annual rate of reduction from 2010 to 2017 was 0.26% (IQR −1.22 to 1.93) among the districts in the low SDI states, 1.95% (IQR 0.19–3.37) in the middle SDI states, and 1.59% (IQR −0.13 to 3.16) in the high SDI states.

The prevalence of underweight decreased from 53.4% (95% UI 52.3–54.6) in 2000 to 40.9% (95% UI 40.3–41.5) in 2010, and 32.7% (95% UI 32.3–33.1) in 2017 in India (Fig. 1 and Appendix pp 44–51). This prevalence varied 4.6-fold between the districts ranging from 11.0% (10.5–11.9) to 51.0% (49.9–52.1) in 2017. The underweight prevalence was more than 35% in 232 (32.1%), 25–35% in 282 (39.0%), and less than 25% in 209 (28.9%) of the 723 districts. 188 (60.3%) of 312 districts in the low SDI, 40 (17.1%) of 234 districts in the middle SDI, and only 4 (2.3%) of 177 districts in the high SDI state group had underweight prevalence more than 35%. From 2010 to 2017 the decline in underweight prevalence was statistically significant in 687 (95.0%) with a maximum decline of 53.9% (95% UI 52.8–55.4, Appendix pp 44–51). This decline was more than 30% in 122 (6.9%) districts, 20–30% in 281 (38.9%) districts, and less than 20% in 284 (39.3%) of the 723 districts (Appendix pp 44–51). Similar proportion of districts in the low (39.7%), middle (41.5%), and high (35.6%) SDI states had a reduction of less than 20%. The median annual rate of reduction from 2010 to 2017 was 3.23% (IQR 2.09–4.40) among the districts in the low SDI states, 3.12% (IQR 1.80–4.04) in the middle SDI states, and 3.31% (IQR 2.11–4.51) in the high SDI states.

### 3.2 Inequality within states

**Fig. 2:**
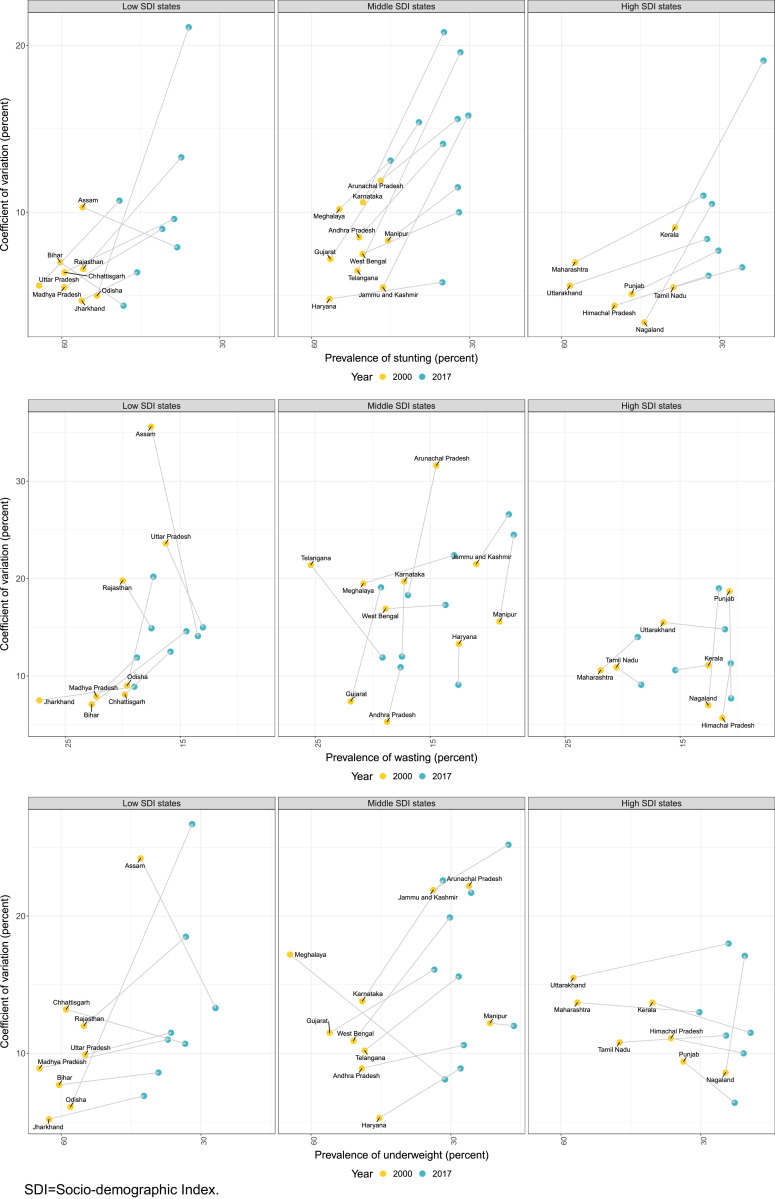
Coefficient of variation for stunting, wasting and underweight between the districts within the states of India, 2000 and 2017. Data shown for states with more than 10 districts.

Inequality between the districts within states, measured as CV, increased for stunting in 28 out of 31 states from 2010 to 2017, for wasting in 16 states, and for underweight in 20 states (Fig. 2 and Appendix p 52). There were wide variations in the magnitude of inequality for the three CGF indicators even between states at similar levels of socio-demographic development. The CV for stunting in 2017 ranged from 4.4% in Bihar to 21.1% in Odisha among the low SDI states, from 5.8% in Haryana to 20.8% in Karnataka among the middle SDI states, and from 2.9% in Delhi to 19.1% in Kerala among the high SDI states. Among the low SDI states, the CV for stunting increased four-fold for Odisha and two-fold for Madhya Pradesh, Uttar Pradesh, and Rajasthan from 2000 to 2017, and decreased in Assam and Bihar. Among the middle SDI states, the CV for stunting increased three-fold for Telangana and Jammu and Kashmir during this period. The CV for stunting increased for all high SDI states except Sikkim, ranging from a modest increase to high increases in Delhi and Nagaland (Appendix p 52).

The CV for wasting prevalence ranged from 8.9% in Jharkhand to 20.2% in Odisha for the low SDI states group, 8.3% in Tripura to 26.6% in Jammu and Kashmir in the middle SDI states group, and 2.2% in Delhi to 19.0% in Nagaland in the high SDI states group (Fig. 2 and Appendix p 52). The CV for wasting prevalence increased in some of the states, while it decreased for the others, spread across the low, middle and high SDI states from 2000 to 2017. Among the low SDI states, the CV increased two-fold for Bihar, Madhya Pradesh, Chhattisgarh, and Odisha from 2000 to 2017. On the other hand, it declined in Assam, Uttar Pradesh and Rajasthan during the same time period. Among the middle and high SDI states, the CV increased in several states and decreased in others.

The CV for underweight prevalence ranged from 6.9% in Jharkhand to 26.7% in Odisha among the low SDI states, 8.1% in Meghalaya to 25.2% in Jammu and Kashmir among the middle SDI states, and from 3.1% in Delhi to 19.2% in Mizoram among the high SDI states (Fig. 2 and Appendix p 52). Among the low SDI states, the CV for underweight increased four-fold for Odisha and two-fold for Rajasthan, but decreased in Assam and Chhattisgarh from 2000 to 2017. There was a mixed pattern of increase or decrease in CV among the middle and high SDI states.

### 3.3 Identification of priority districts in states

We use examples of three states in the low SDI group to highlight how the differences in prevalence and rate of change over time can help identify districts that need higher priority attention. We selected Odisha as it had the highest inequality between districts for all three CGF indicators in 2017, Uttar Pradesh as it had the highest level of stunting and medium level of inequality between the districts, and Bihar as it had one of the highest levels of stunting and among the lowest level of inequality between districts.

Based on tertiles of the distribution, the districts in the south-west handle of Odisha generally had high prevalence in 2017 and low rate of reduction from 2010 to 2017 for the CGF indicators (Fig. 3). Kalahandi, Koraput, and Rayagada in this group stood out as having this trend for all three CGF indicators. Balangir in this group had this trend for stunting and underweight, while Nuapada, Nabarangapur and Malkangiri in this group had this trend for underweight and wasting. In addition, districts with either high prevalence and medium rate of reduction or medium prevalence and low rate of reduction would also need attention for them not to spill over to the worst group with high prevalence and low rate of reduction. Bargarh had high prevalence and medium rate of reduction for stunting and wasting. Mayurbhanj and Kendujhar in the north-west part of the state also had high rates of stunting and underweight with low or medium rates of reduction. A cluster of districts in the south-central part of the state had a medium rate of prevalence and low rates of reduction, which included Kandhamal with this trend for all three indicators, Gajapati with this trend for stunting and wasting, and Ganjam with this trend for stunting. Baleshwar in the north-east part of the state also had this trend for stunting.

**Fig. 3:**
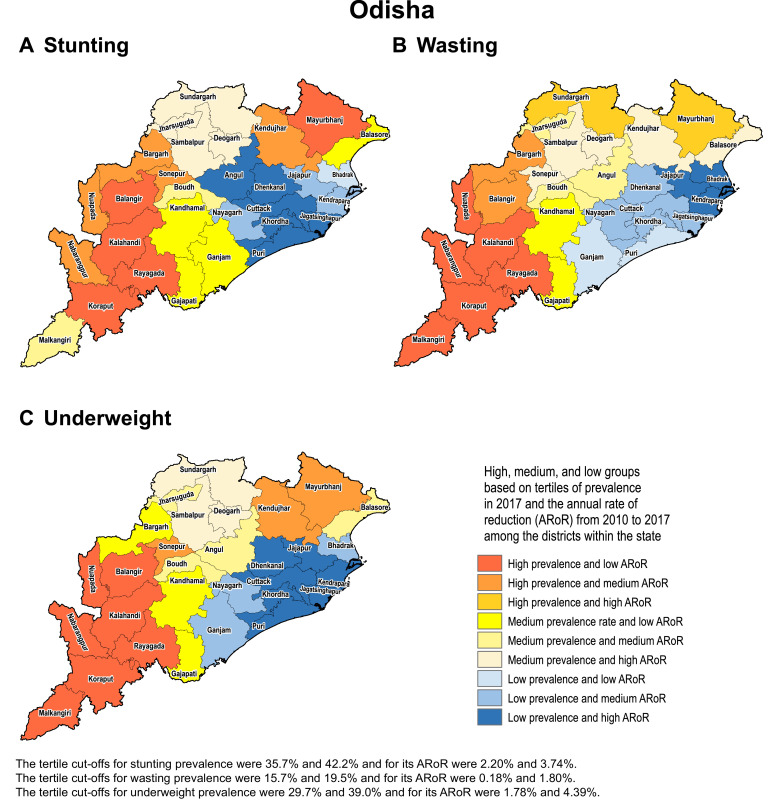

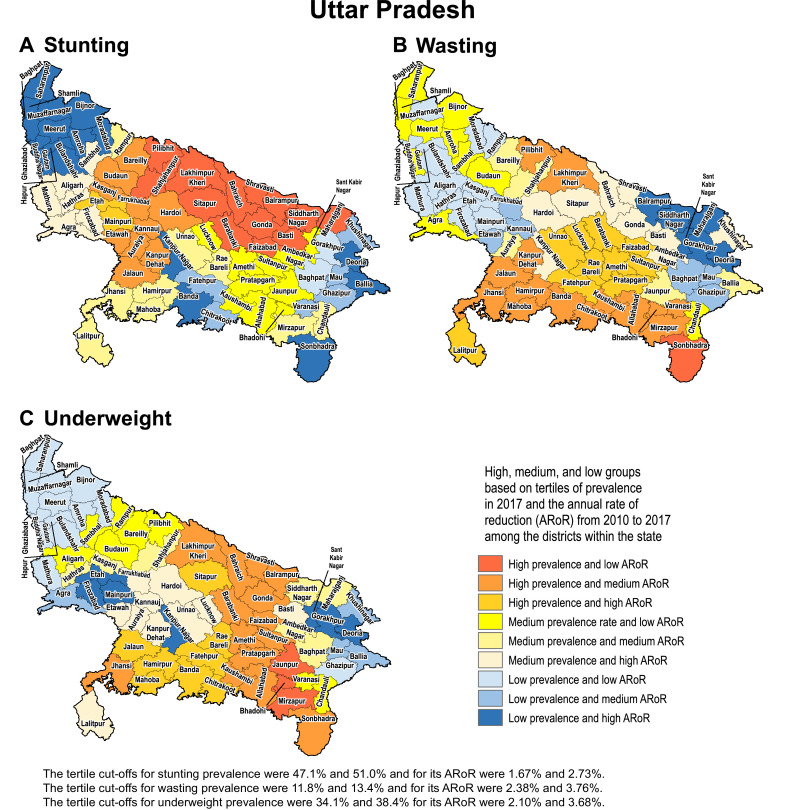
Identification of priority districts of Odisha, Uttar Pradesh and Bihar based on tertile groupings of district-level prevalence of child growth failure indicators in 2017 against the annual rate of their reduction from 2010 to 2017.

**Fig. 3:**
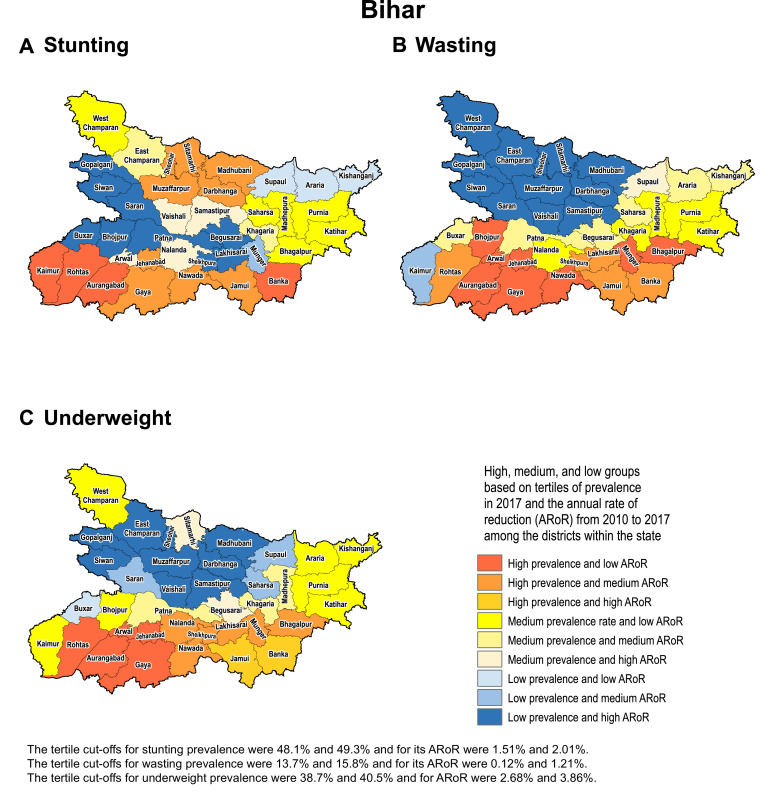

A similar approach based on tertiles of the distribution revealed that no district in Uttar Pradesh fell in the category of high prevalence and low rate of reduction for all three CGF indicators, showing a different pattern from Odisha (Fig. 3). A cluster of 13 districts in the northern part of Uttar Pradesh had high prevalence and low rate of reduction for stunting, a cluster of three districts in the south-east part of the state had this trend for underweight, and one neighbouring district had this trend for wasting. Aurangabad district in south-west of Bihar stood out as having a high prevalence and low rate of reduction for all three CGF indicators. This trend was present in the three neighbouring districts for underweight and wasting. One district in the south-west corner of the state had this trend for stunting, and one each in the west and east had this trend for wasting. As in Odisha, there were districts with high prevalence and medium reduction rate or medium prevalence and low reduction rate that would also need attention. Using this approach, the identification of priority districts in the other 15 states with 20 or more districts is shown in the appendix (pp 53–67).

Examining districts in these three states based on tertiles of the nationwide distribution of stunting, wasting and underweight and their rate of reduction provides a complementary understanding to that obtained using tertiles of the state-level distribution (Figs. 1 and 3). All 38 districts in Bihar were in the high tertile of stunting for the national distribution and 94.7% in the high tertile for underweight, and none were in the high tertile for the rate of reduction for stunting (Appendix p 68). Likewise, in Uttar Pradesh, 97.3% of the districts fell in the high tertile for stunting and only 12.0% were in the high tertile for the rate of reduction. For wasting, 60.0% of the districts in Odisha were in the high tertile of prevalence for the national distribution, which was in contrast to 66.7% districts in Uttar Pradesh in the low tertile.

### 3.4 Comparison of trends with targets

**Fig. 4:**
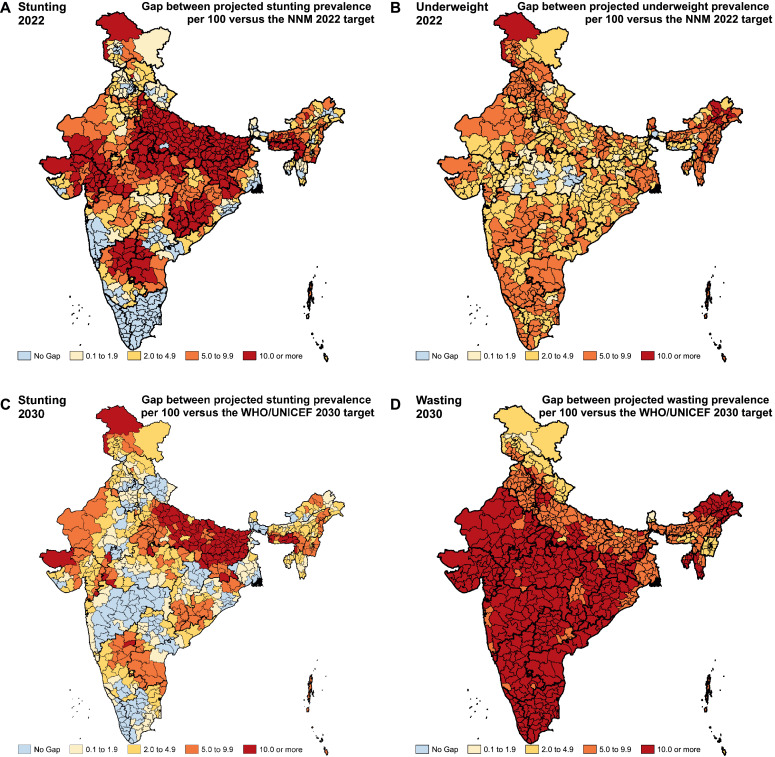
Gap between the projected prevalence of child growth failure indicators in the districts of India in 2022 and 2030 based on the trends from 2000 to 2017 versus the NNM 2022 and the WHO/UNICEF 2030 targets.

In order to reach the NNM 2022 target of 25.0% stunting prevalence individually, 597 (82.6%) of the 723 districts in India would need a rate of improvement higher than they had up to 2017 (Fig. 4 and Appendix pp 69–76). This includes 307 (98.4%) of the 312 districts in low SDI states, 191 (81.6%) of the 234 districts in middle SDI states, and 99 (55.9%) of the 177 districts in high SDI states. Similarly, to reach the WHO/UNICEF 2030 target of 50.0% reduction in stunting prevalence from 2012, individually 79.8% of the districts in India would need a higher rate of improvement than they had up to 2017; this proportion was 89.4%, 79.9% and 62.7% in the low, middle and high SDI states, respectively (Fig. 4 and Appendix pp 77–85). If the trends up to 2017 were to continue, the gap between the projected prevalence and the WHO/UNICEF 2030 stunting target would be 10.0% or more in 109 (15.1%), 5.0–9.9% in 153 (21.2%), and less than 5.0% in 315 (43.6%) of the total districts. To reach the NNM 2022 underweight target of 2 percentage point reduction annually, 98.5% of the districts would need a rate of improvement higher than they had up to 2017 (Fig. 4 and Appendix pp 69–76).

To reach the WHO/UNICEF 2030 target of wasting prevalence less than 3.0%, all districts in India would require a higher rate of improvement than they had up to 2017 (Fig. 4 and Appendix pp 77–85). If the trends up to 2017 were to continue, the gap between the projected prevalence and this target would be 10.0% or more in 398 (55%), 5.0–9.9% in 266 (36.8%), and less than 5.0% in 58 (8.0%) of the total districts in India.

### 3.5 Correlation between major national surveys

**Fig. 5:**
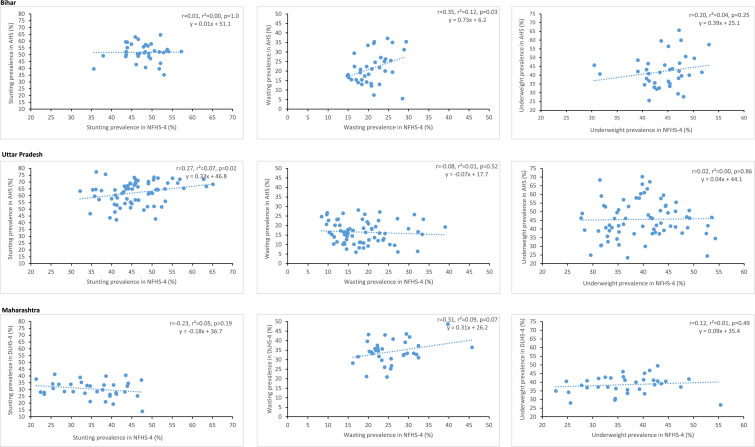
Correlation between the national surveys for district-level prevalence of stunting, wasting and underweight in Bihar, Uttar Pradesh and Maharashtra. NFHS = National Family Health Survey; DLHS = District-level household survey; AHS = Annual Health Survey; r = Pearson correlation coefficient.

The correlation between the major national surveys, NFHS-4 and AHS which covered the same nine states, for district-level estimates of the CGF indicators was significant only in three states for stunting, three states for wasting, and two states for underweight, Pearson correlation coefficient was more than 0.7 only in Odisha for stunting (r = 0.79, *p* < 0.0001) and underweight (r = 0.73, *p* < 0.0001; Appendix p 86). In the two states with the highest prevalence of stunting in 2017, there was no correlation between these two surveys in Bihar (r = 0.01, *p* = 0.95) and a very poor correlation in Uttar Pradesh (r = 0.27, *p* = 0.024), and also no or very poor correlation for underweight and wasting (Fig. 5 and Appendix p 86). Chhattisgarh had an inverse correlation for wasting between the two surveys (r = −0.72, *p* = 0.002; Appendix p 86). The correlation between NFHS-4 and DLHS-4, which covered the same 18 states, for district-level estimates of the CGF indicators was significant only in four states for stunting, in three states for wasting, and in two states for underweight, but with a r of more than 0.7 only in four small states in the northeast part of India and in none of the other larger states (Appendix p 86).

## 4 Discussion

This report provides comprehensive estimates of the prevalence of CGF indicators in every district of India from 2000 to 2017 and compares these trends with the Indian and the global targets up to 2030 to inform district-specific policy action under NNM. The prevalence of CGF indicators has declined across India, but inequality between the districts within the states has increased for most of the states, indicating opportunities for improved targeting of efforts to reduce CGF. There was a four-fold variation between the districts of India for stunting prevalence and five-fold for wasting and underweight prevalence in 2017.

The vast majority of districts in India need acceleration in their rate of CGF reduction to reach the Indian 2022 and the global 2030 targets. Our findings suggest that if the trends up to 2017 were to continue, the gap between the projected prevalence and the WHO/UNICEF 2030 target would be 5.0% or more for stunting in 36.0% of the districts in India and for wasting in 92.0% of the districts. The extent of the potential gap in each district if the past trends were to continue provides useful information for NNM as it indicates the extent of additional effort needed to meet the targets. It should be noted though that the Indian and global CGF targets are for the country as a whole, but we have applied these to each district individually.

The findings suggest a variety of dynamics among the states for the overall reduction in prevalence and the variance of prevalence across districts within the states. For example, inequality between districts in the prevalence of stunting increased in the states of Odisha, Madhya Pradesh, Uttar Pradesh, Chhattisgarh, and Rajasthan from 2000 to 2017 but decreased in Assam, whereas all of these low SDI states had a similar one-third overall reduction in stunting prevalence during this period. Likewise, with a similar overall reduction in stunting prevalence of a lower magnitude of about 20.0% in Bihar and Jharkhand, the former had a one-third decline in inequality between districts and the latter had a one-third increase. An example of varying dynamics for wasting is that Bihar had a 36.0% reduction in wasting prevalence from 2000 to 2017 and Odisha a lower 12.0% reduction, but in both states the inequality between districts doubled during this period. The states in which inequality between districts for the prevalence of CGF indicators has increased would benefit from better targeting of districts in which the reduction rates have been lower.

We use a relatively simple approach in this report of grouping districts within each state in nine groups using tertiles of the prevalence of CGF indicators and their rate of reduction, which could be useful for policy makers to identify districts that need priority attention. While the districts in the high prevalence and low rate of reduction category would need the highest attention, those in either the high prevalence and medium rate of reduction category or the medium prevalence and low rate of reduction category would also need attention for them not to spill over to the former category. NNM is being implemented at the district-level in a phased manner, with the prioritization of districts for roll out in phases one and two of NNM largely based on stunting prevalence [36]. We suggest that considering the rate of reduction in recent years in addition to the prevalence while prioritizing districts for action would be useful. Other more complex approaches have also been suggested for better targeting of investments in nutrition programming to achieve greater impact on reducing CGF [37].

We found a lower decline in wasting prevalence in India as compared with stunting, with a subset of districts even showing an increase in wasting. A temporary increase or stagnation in wasting prevalence when stunting is declining has been reported previously [38]. It is being increasingly realized that acute wasting and chronic stunting represent different but closely related aspects of malnutrition in communities, as they may occur in the same children at different stages and concurrently among children in the same population [39], [40], [41], [42], [43]. Stunting and wasting are often separated in terms of policy interventions inspite of the fact that stunting is an adaptation to repeat episodes of wasting and both are a consequence of similar determinants [41,42]. Underweight overlaps with both stunting and wasting, and there are suggestions to address the various aspect of undernutrition as part of the same continuum in a holistic manner [44].

CGF is a result of interaction between a wide variety of factors, which include economic development and urbanisation, socioeconomic status, parent's education, women's decision-making status, water and sanitation, maternal nutritional status before conception and during pregnancy, maternal age and height, birth order, child birthweight, dietary intake and diversity, and access to nutritional and health services [45], [46], [47], [48], [49], [50], [51], [52], [53], [54], [55], [56], [57]. Poor nutritional status of women, maternal age and height, and birth order have been reported to be associated with fetal growth restriction and preterm birth, which in turn increase the likelihood of CGF [46,47,49,50,53,57]. Interventions aimed at improving nutrition in the pre-conception period, during pregnancy, and early postpartum period have been shown to benefit maternal nutritional status [56]. Poor dietary diversity and delayed complementary feeding have also been reported to be associated with an increased risk of CGF [52,54]. A study has reported that the proportion of children in India who do not meet the recommended dietary allowance for calories, protein and fat intake was quite high, but there was weak correlation between this and the CGF indicators, indicating that only improving dietary intake is not enough to reduce CGF [58]. Given the multitude of factors that influence child growth, efforts at improving CGF have to address the variety of multi-sectoral determinants.

To address undernutrition, India devised its first National Nutrition Policy in 1993 aggregating various programmes under one umbrella [59], [60], [61]. Other policies such as National Health Policy 2002 and 2017 and the National Policy for Children 2013 have also set a foundation for addressing malnutrition [62,63]. The lack of focus previously on children below three years during this critical period of life has contributed to the slow progress in malnutrition. To address this need, NNM has been designed to provide a continuum of care through a comprehensive package of convergent interventions across multiple government schemes and programmes focusing on the first 1000 days of the child, which includes the nine months of pregnancy, six months of exclusive breastfeeding, and the period from 6 months to 2 years [64]. Additional one year of sustained intervention would ensure that the gains of the first 1000 days are consolidated [36]. The implementation strategy under NNM would focus on the district and sub-district levels to bring convergence in addressing the multi-sectoral and multi-dimensional nature of malnutrition [20]. Swachh Bharat Mission has made substantial efforts to improve sanitation coverage across India which is likely to be beneficial for CGF as well due to its interaction with sanitation [65]. Efforts are underway in India for targeted development of districts under the Aspirational Districts Programme [66], which is also expected to have a positive impact on child malnutrition.

Several states of India have nutrition intervention programmes aimed at reducing malnutrition and some states have made more progress than others which could offer learnings for broader application [67], [68], [69], [70], [71]. Evidence from various low- and middle-income countries suggests that successful interventions to reduce CGF include a combination of political commitment, multi-sectoral collaboration, community engagement, community-based service delivery platform, and wider programme coverage and compliance [55,72]. The success of Peru in reducing its stunting rate by more than half in less than a decade through strong political commitment, pro-poor policies, and implementation of a multi-sectoral approach to address the various determinants of stunting could offer useful insights for India and other countries that have high levels of CGF [73,74]. NNM is attempting to address many of these aspects together in a coordinated manner in India, which is expected to enhance the rate of improvement and increase the likelihood of reaching the targets. Interestingly, a cost-benefit analysis has suggested that nutrition interventions have good returns, with stunting reduction in children leading to subsequent higher wage earning as adults [75].

We found a very poor correlation between the major national surveys for district-level estimates of CGF indicators in India. This may be due to differences in the methods and quality control between the two surveys. This discrepancy has also been observed in several other countries across surveys [76]. Nutrition programmes are often based on estimates of malnutrition obtained from national surveys. However, multiple surveys with different sampling strategies and methodologies conducted over various time periods pose a challenge as to which one is closer to the true burden of undernutrition [77,78]. Obtaining high quality anthropometry data for children can be difficult in large scale surveys [79], but these data can be improved by following a quality assurance protocol and standard operating procedures [80,81]. To deal with this challenge in our analysis, we used all accessible data sources from India in a consolidated framework to produce the best possible estimates of CGF indicators.

The limitations of the estimation and mapping methods of CGF that we used are described elsewhere [5,26]. A summary of the limitations follows. The quality of anthropometry data collected in various surveys is variable as noted above. Height and weight data in surveys are often prone to biases as they may have been measured or recorded incorrectly, and there may be recall error of the child's age in less developed settings, which underscores the need for improving data collection methodology and the design of surveys. The surveys do not capture anthropometric measurements of children who died due to malnutrition or other causes before the survey, thus potentially underestimating CGF. The 95% UIs are relatively wider in years where we do not have good anthropometric data. This needs to be addressed with better quality data over time. Data for many covariates used were available only for geographic clusters and not at the child-level, which may have masked some of the heterogeneity. The spatial covariates used in this report, although comprehensive, do not include the complete set of drivers or confounders due to lack of spatially disaggregated data on these. We mapped CGF prevalence for both sexes together, which would have masked potential differences between boys and girls. The strengths of the findings in this report include the use of all accessible data sources in India to produce the best possible estimates of child growth failure indicators at the district-level by aggregating the estimates from 5 × 5 km grids. The pooling of various data sources reduces the biases associated with single survey estimates and is likely to lead to more robust estimates than from individual surveys separately. In that sense, the aggregated estimates in this paper may differ from those reported in individual national surveys. The categorisation of districts based on prevalence, rate of reduction, and socio-demographic index, as done in this study, provides various options for policy makers to formulate intervention strategies.

Substantial district-level variations in the magnitude of CGF indicators and their rate of decline, and the increasing inequality between districts in a large proportion of the states, as presented in this report provides robust district-level trends that can serve as a resource for NNM to inform the extent of effort needed in each district to meet the malnutrition reduction targets. These comprehensive and granular estimates based on composite analysis of all accessible data sources in India have substantial policy and implementation relevance, as these could enable better strategic targeting of resources at sub-state levels to reduce CGF. NNM also suggests planning of action at the sub-district block level as districts in India are relatively large with an average population of about 2 million. The fine-grid geospatial mapping approach presented in this report has the potential for providing CGF trends at the sub-district level as well.

## Declaration of Competing Interest

RH, AL, PGB, JKC, SD, ZG, RK, AN, KVR, DSS, RSS, CS, AS, RSM, GST and LD work with the Indian Council of Medical Research, which partially funded this research. All other authors declare no competing interests.

## Appendix Supplementary materials

Image, application 1

Image, application 2
