# Supplementary material for: Mapping of variations in child stunting, wasting and underweight within the states of India: the Global Burden of Disease Study 2000–2017
Source: eClinicalMedicine. 2020 May 13;22:100317. doi: 10.1016/j.eclinm.2020.100317 (PMC7264980; doi:10.1016/j.eclinm.2020.100317)
Supplement: Supplementary file 2 [file mmc2.doc]

**Mapping of variations in child stunting, wasting and underweight within the states of India: the Global Burden of Disease Study 2000-2017**

India State-Level Disease Burden Initiative CGF Collaborators

**Web appendix**

Correspondence to: Prof. Lalit Dandona, lalit.dandona@icmr.gov.in

**Table of contents**

[1. Child growth failure estimation and mapping methods 3](#_Toc33531604)

[2. Data inputs for child growth failure estimation, mapping and covariates for India 22](#_Toc33531605)

[3. Grouping of the states of India based on Socio-demographic Index, 2017 27](#_Toc33531606)

[4. Prevalence of stunting in the districts of India in 2000, 2010 and 2017 28](#_Toc33531607)

[5. Prevalence of wasting in the districts of India in 2000, 2010 and 2017 36](#_Toc33531608)

[6. Prevalence of underweight in the districts of India in 2000, 2010 and 2017 44](#_Toc33531609)

[7. Coefficient of variation for stunting, wasting and underweight between districts within the states of India, 2000 and 2017 52](#_Toc33531610)

[8. Identification of priority districts in states with 20 or more districts 53](#_Toc33531611)

[9. Distribution of districts in select states by tertiles of the national distribution of child growth failure indicators 68](#_Toc33531612)

[10. Projected prevalence of stunting and underweight for the districts of India in 2022 based on trends from 2000 to 2017 versus the NNM 2022 targets 69](#_Toc33531613)

[11. Projected prevalence of stunting and wasting in the districts of India in 2030 based on the trends from 2000 to 2017 versus the WHO/UNICEF 2030 targets 77](#_Toc33531614)

[12. Correlation between the national surveys for district-level prevalence of stunting, wasting and underweight 86](#_Toc33531615)

# **1.** **Child growth failure estimation and mapping methods**

The materials presented here are adapted from the following sources:

- GBD 2017 Risk Factors Collaborators. Global, regional, and national comparative risk assessment of 84 behavioural, environmental and occupational, and metabolic risks or clusters of risks for 195 countries and territories, 1990–2017: a systematic analysis for the Global Burden of Disease Study 2017. *Lancet* 2018; 392: 1923-94.
- India State-Level Disease Burden Initiative Malnutrition Collaborators. The burden of child and maternal malnutrition and trends in its indicators in the states of India: the Global Burden of Disease Study 1990–2017. *Lancet Child & Adolescent Health* 2019; 3: 855–70.
- Osgood-Zimmerman A, Millear AI, Stubbs RW, Shields C, Pickering BV, Earl L, et al. Mapping child growth failure in Africa between 2000 and 2015. *Nature* 2018; 555: 41-7.
- Local Burden of Disease Child Growth Failure Collaborators. Mapping child growth failure across low- and middle-income countries. *Nature* 2020; 577: 231–234
- GBD 2017 SDG Collaborators. Measuring progress from 1990 to 2017 and projecting attainment to 2030 of the health-related Sustainable Development Goals for 195 countries and territories: a systematic analysis for the Global Burden of Disease Study 2017. *Lancet* 2018; 392: 2091-138.

1. **GBD estimation process for risk factors including child growth failure**

The approach used in GBD 2017 for comparative risk assessment to estimate exposure values for risk factors is shown in the following flowchart.


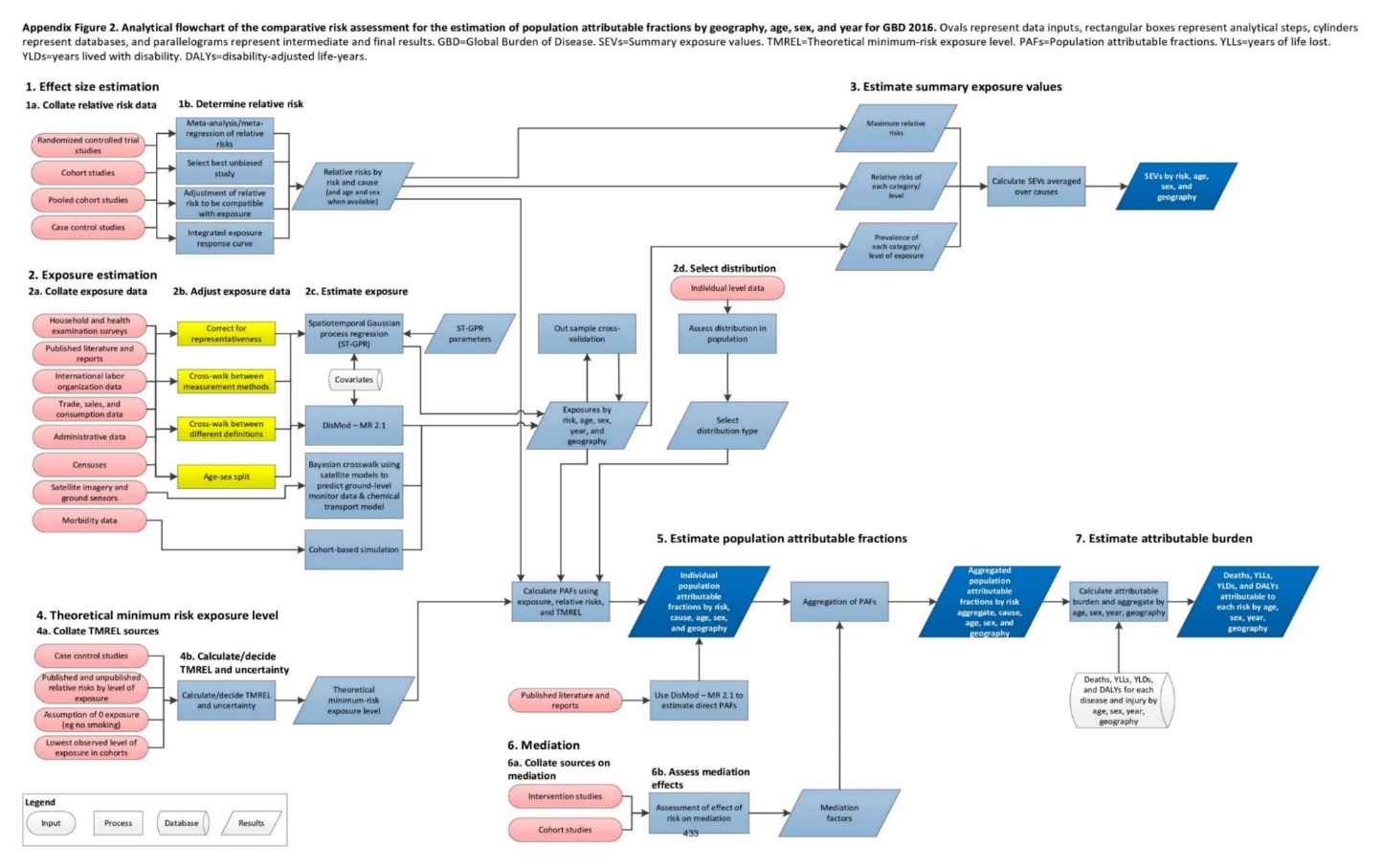


GBD is Global Burden of Disease. SEV is summary exposure value. TMREL is theoretical minimum‐risk exposure level. PAF is population attributable fraction. YLL is years of life lost. YLD is years lived with disability. DALY is disability‐adjusted life‐year. Ovals represent data inputs, rectangular boxes represent analytical steps, cylinders represent databases, and parallelograms represent intermediate and final results.

1. **Child growth failure estimation methods**

Child growth failure (CGF) was estimated using three indicators stunting, wasting and underweight all of which are based on categorical definitions using the World Health Organisation (WHO) 2006 growth standards for children 0-59 months. Stunting, wasting and underweight were defined as z-scores that were two or more standard deviations (SD) below the WHO healthy population reference median for length/height-for-age, weight-for-length/height and weight-for-age, respectively, for age- and sex-specific curves.^1^ Mild, moderate and severe categorical prevalences were estimated for each of the three indicators.

Theoretical Minimum Risk Exposure Level for stunting, wasting and underweight was assigned to be greater than or equal to -1 SD of the WHO 2006 standard height-for-age, weight-for-height and weight-for-age curves, respectively.

The steps in the estimation of child growth failure are shown in the following flowchart:

**Data**

The three main inputs for the child growth failure estimation models were: microdata from population-based surveys including anthropometric surveys, tabulated data from reports and published literature, and the WHO Global Database on Child Growth and Malnutrition.^2^ Population surveys include a variety of multi-country and country-specific survey series such as Demographic and Health Surveys, Multiple Indicator Cluster Surveys, Living Standards Measurement Surveys, as well as other one-time country-specific surveys. These microdata contain information about each individual child’s age (from which age in weeks and age in months are calculated), as well as height and/or weight. From this information, a height-for-age z-score (HAZ), weight-for-height z-score (WHZ) and weight-for-age z-score (WAZ) were calculated using the WHO 2006 Child Growth Standards and the LMS method.^3,4^

All available data from the WHO Global Database on Child Growth and Malnutrition were extracted – majority of which was from published studies. Four metrics that were sought from all sources with tabulated data were: mean z-score, prevalence <-1 z-score (mild), prevalence <-2 z-score (moderate), and prevalence <-3 z-score (severe). All data for each metric was extracted for each of stunting (HAZ), wasting (WHZ) and underweight (WAZ).

To maximise internal-consistency and comprehensiveness of the modelling dataset, three data transformations were performed. Firstly, any data that were reported using the National Center for Health Statistics 1978 growth standards were crosswalked to corresponding values on the WHO 2006 Growth Standards curves based on a study that evaluated growth standard concordance.^5^ Crosswalks from 1978 to 2006 growth standards were performed only on <-2 z-score (i.e. moderate) prevalence data as that was where the concordance was most consistent. Secondly, for any study that lacked a measure of mean z-score for any of stunting, wasting or underweight, a mean value was predicted for that study based on an ordinary-least squares regression of mean z-score versus <-2 z-score prevalence for that metric from all sources where both were available. Thirdly, any data that were presented as both sexes combined for 0-59 months, the age- and sex-pattern were used from all data sources that included that detail to split these data into corresponding age-and sex-specific data.

The major data sources from India were national surveys including National Family Health Surveys, District Level Household Surveys, Annual Health Surveys, National Nutrition Monitoring Bureau Diet and Nutritional Surveys, and Rapid Survey on Children.

**Modelling**

The following three-step modelling process was used to estimate stunting, wasting and underweight.

First, all microdata was fit using an ensemble modelling process in which a model selection algorithm was used to choose the best model for continuous risk factors.^6^ A series of 12 individual distributions (normal, log normal, log logistic, exponential, gamma, mirror gamma, inverse gamma, gumbel, mirror gumbel, Weibull, inverse Weibull, and beta) were fit to the entire set of microdata (approximately 2.5 million individual z-scores) at the individual survey level. A weighting algorithm combined each distribution to find the optimal combination of these distributions for each survey, minimising the absolute prediction error across the entire distribution. Ensemble weights for each survey were then averaged across all surveys to produce a single set of global weights of the ensemble distributions. Weights were different for each sex, but invariant across geography, time, and age group. All component distributions that were used to derive weights were parameterised using “method of moments,” meaning that each corresponding probability density function could be described as a function of the mean and variance of the quantity of interest.

Second, models were developed for mean z-scores and prevalence of moderate and severe growth failure. Individual level microdata were collapsed to calculate three metrics: mean z-score, moderate prevalence and severe prevalence. These data were combined with that derived from literature, Global Health Data Exchange review, and the WHO Global Database on Child Growth and Malnutrition. For those sources where moderate prevalence was reported without a corresponding mean, a predicted mean was calculated using an ordinary-least square regression from those sources where both metrics were present. Each of the three metrics were then modelled using spatial-temporal Gaussian process regression (ST-GPR), generating estimates for each location, year, age group, and sex. ST-GPR was used because the data density was sufficient to estimate a very flexible time trend. ST-GPR is a stochastic modelling technique that is designed to detect signals amidst noisy data. It also serves as a powerful tool for interpolating non-linear trends.^7,8^ Unlike classical linear models that assume that the trend underlying data follows a definitive functional form, GPR assumes that the specific trend of interest follows a Gaussian Process, which is defined by a mean function and a covariance function. The ST-GPR model has three main hyper-parameters that control for smoothing across time, age and location. Values for these hyper-parameters were selected based on cross-validation. Cross-validation tests were conducted for different combinations of the hyper-parameters for three types of models: one data-sparse model, one data-moderate model, and one data-dense model. In each test, 20% of the data were held out and the performance of each combination of hyper-parameters evaluated on the held out data. For each hyper-parameter combination, 10 cross-validation tests were conducted. The performance of each model in predicting the withheld 20% of the data was evaluated using a combined measure based on root mean square error (RMSE) and uncertainty interval coverage.

Third, estimates of mean and prevalence (moderate and severe) were combined with ensemble weights in an optimisation framework in order to derive the variance that would best correspond to the predicted mean and prevalence. This variance was then paired with the mean, and using the method of moments, equation for each of the component distributions of the ensemble, probability density function of the distribution of z-scores were calculated for each location, year, age group, and sex. Probability density functions were integrated to determine the prevalence between -1 and -2 z-scores (mild), between -2 and -3 z-scores (moderate), and below -3 z-scores (severe).

All models were run with the complete dataset. Data plausibility inspection began with examination of time-trends in stunting. If a given datum was judged to have led to a change in the prevalence of moderate stunting in 1-4 year olds of 50% or greater in 5 years or fewer, and was inconsistent with data prior to and after that year (a change considered implausible), the offending datum were outliered and the model was re-run. The results of moderate stunting, wasting and underweight were further visually-inspected in parallel to look for location-year-age-sex where the results were not internally-consistent (e.g. underweight rapidly increasing, and stunting and wasting decreasing). This inspection revealed very few inconsistent data.

To ensure internal consistency of the estimates between the subnational locations within a country, national estimates were either created by population-weighted aggregation or subnational estimates were adjusted by population-weighted scaling to the national estimates, depending on the data coverage of a given country compared to that of its subnational locations. For example, if there was better data coverage at the national level, relative to its corresponding subnational locations, for a given country and risk across age, sex and time, estimates were rescaled to be consistent with the national level. Conversely, if there was better data coverage at the subnational level, estimates for its parent country were generated through population-weighted aggregation of subnational estimates. In the GBD iteration, an option was incorporated to scale estimates within logit space. Scaling in logit space ensures that subnational estimates of proportion models will not exceed one after being rescaled to the national estimate.

1. **Child growth failure geospatial mapping methods**

This analyses used model-based geostatistics^9^ to generate local, administrative and national estimates of child stunting, wasting and underweight prevalence in India and trends over time. Using an ensemble modelling framework which feeds into a Bayesian generalised linear model with a correlated space-time error, and 1000 draws from the fitted posterior distribution, annual prevalence estimates were generated for each CGF indicator on a 5×5 km grid from 2000 to 2017 and aggregated to administrative and national levels. The following sections provide detailed description on the analytical process.

The steps in the geospatial estimation of CGF indicators are shown in the following flowchart:


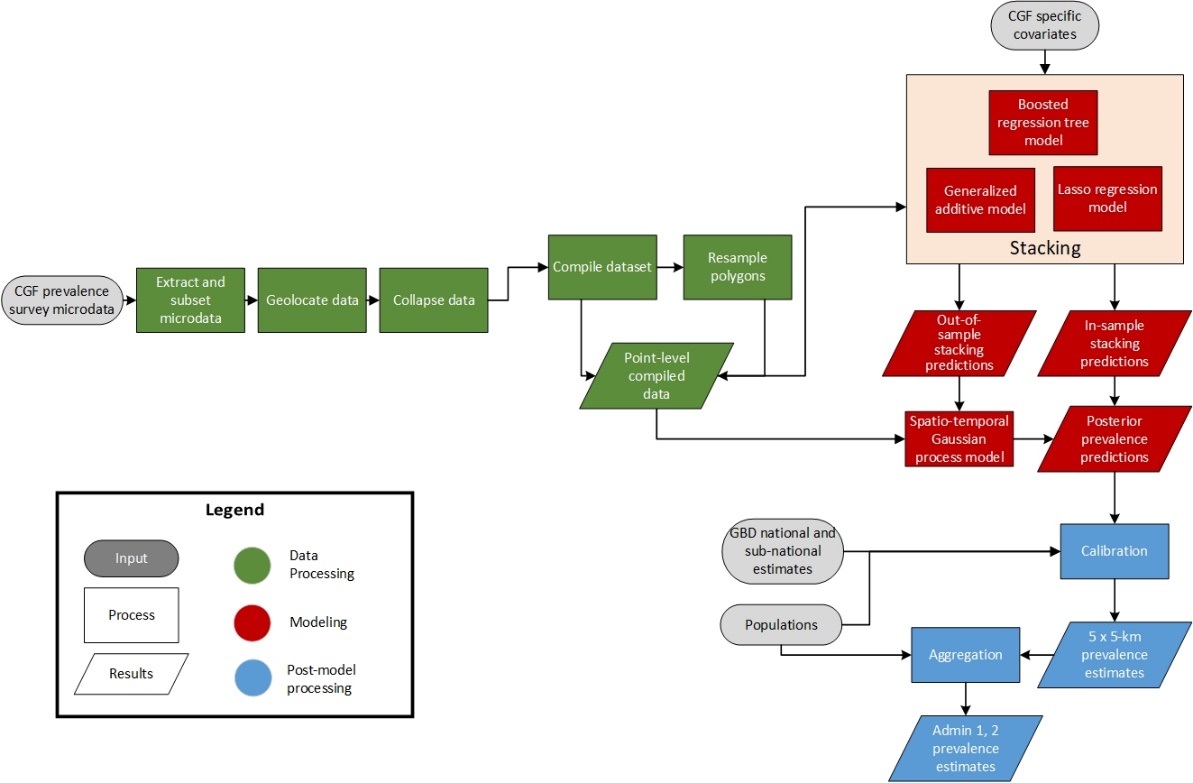


**Data sources**

The data sources used for the geospatial modelling of CGF indicators are described in the table below. The table shows information on years, source, and number of individuals, polygons, and/or geo-positioned clusters (points). India-Vellore Malnutrition and Enteric Disease Study 2009-2014 was excluded from the analysis as the anthropometric data was not collected on full set of under-five children and the India Tribal Second Repeat Survey of Diet and Nutrition Status 2007-2008 was excluded because geographies could not be mapped.

**The list of data sources for child growth failure mapping in India**

| **Survey years** | **Survey name** | **Number of children** | **Poly**gons | **Points** |
| --- | --- | --- | --- | --- |
| 1998-1999 | International Institute for Population Sciences (IIPS), Ministry of Health and Family Welfare, Government of India, ICF. India National Family Health Survey Data (NFHS-2) 1998-1999 Mumbai, India: IIPS. | 27,937 | 438 | 0 |
| 2000-2001 | National Nutrition Monitoring Bureau, National Institute of Nutrition (NIN), Indian Council of Medical Research. India Rural Survey of Diet and Nutritional Status Data 2000-2001. [Data shared for this analysis] | 9,262 | 9 | 0 |
| 2002-2004 | International Institute for Population Sciences (IIPS), Ministry of Health and Family Welfare, Government of India. India District Level Household Survey Data (DLHS-2) 2002-2004. Mumbai, India: IIPS. | 241,944 | 537 | 0 |
| 2004-2006 | National Nutrition Monitoring Bureau, National Institute of Nutrition (NIN), Indian Council of Medical Research. India Rural Survey of Diet and Nutritional Status Data 2004-2006. [Data shared for this analysis] | 6,670 | 9 | 0 |
| 2005-2006 | International Institute for Population Sciences (IIPS), Ministry of Health and Family Welfare, Government of India, ICF. India National Family Health Survey Data (NFHS-3) 2005-2006: Mumbai, India: IIPS. | 44,061 | 29 | 0 |
| 2007-2011 | Center for Vaccine Development, University of Maryland, Centers for Disease Control and Prevention, Department of Medical Microbiology and Immunology, Göteborg University, International Vaccine Institute, National Institute of Cholera and Enteric Diseases, Perry Point Cooperative Studies Program Coordinating Center, U.S. Department of Veterans Affairs, School of Medicine, University of Virginia, University of Chile. India - Kolkata Global Enteric Multicenter Study 2007-2011. Baltimore, MD, United States: Center for Vaccine Development, University of Maryland. | 2,014 | 0 | 1 |
| 2011-2012 | National Nutrition Monitoring Bureau, National Institute of Nutrition (NIN), Indian Council of Medical Research. India Rural Third Repeat Survey of Diet and Nutritional Status Data 2011-2012. [Data shared for this analysis] | 10,300 | 3 | 1,125 |
| 2011-2013 | Center for Vaccine Development, University of Maryland, Centers for Disease Control and Prevention, Department of Medical Microbiology and Immunology, Göteborg University, International Vaccine Institute, National Institute of Cholera and Enteric Diseases, Perry Point Cooperative Studies Program Coordinating Center, U.S. Department of Veterans Affairs, School of Medicine, University of Virginia, University of Chile. India - Kolkata Global Enteric Multicenter Study 2011-2013. Baltimore, United States: Center for Vaccine Development, University of Maryland. | 1,172 | 0 | 1 |
| 2012-2014 | International Institute for Population Sciences (IIPS), Ministry of Health and Family Welfare, Government of India. India District Level Household and Facility Survey Data (DLHS-4) 2012-2014. Mumbai, India: IIPS. | 73,542 | 276 | 0 |
| 2014 | Office of the Registrar General & Census Commissioner, Ministry of Home Affairs, Government of India. India Annual Health Survey- Clinical, Anthropometric and Bio-chemical survey Data 2014. New Delhi, India: Office of the Registrar General & Census Commissioner. | 108,693 | 277 | 0 |
| 2015-2016 | International Institute for Population Sciences (IIPS), Ministry of Health and Family Welfare, Government of India, ICF. India National Family Health Survey Data (NFHS-4) 2015-2016: Mumbai, India: IIPS. | 237,528 | 0 | 28,118 |
| 2015-2016 | National Nutrition Monitoring Bureau, National Institute of Nutrition (NIN), Indian Council of Medical Research. India Urban Nutrition Survey Data 2015-2016. [Data shared for this analysis] | 12,027 | 2 | 564 |

Each individual child record was associated with a cluster, a group of neighbouing households or a ‘village’ that acts as a primary sampling unit. Some surveys included geographical coordinates or precise place names for each cluster within that survey. In the absence of geographical coordinates for each cluster, data were assigned to the smallest available administrative areal unit in the survey, either state or district, while correcting for the survey sample design.^10,11^ Boundary information for these administrative units were obtained as shapefile from ML Infomap (https://www.mlinfomap.com/). These areal data were resampled to point locations using a population-weighted sampling approach over the relevant areal unit with the number of locations set proportionally to the number of grid cells in the area and the total weights of all the resampled points summing to one.^12^

The data processing pipeline began with raw survey microdata and ended with the input data for the model. Names and measurement units of relevant CGF data were extracted and standardised, and the corresponding survey clusters were matched with the finest geographies possible. Observations (representing children) were dropped due to insufficient or implausible data for age, height (≤0 cm or ≥180 cm), or weight (≤0 kg or ≥45 kg). Z-scores were calculated using height, weight and age data, and implausible z-scores^3,4,13^ (according to WHO reference population^1^) were dropped. Children that met the definitions of stunted, wasted or underweight were identified, and collapsed by survey, year and geography. Children that could not be matched to a geography were also dropped. Survey reports that were manually extracted at an aggregated level, most often at the first administrative (Admin 1) or second administrative (Admin 2) level, were appended to the collapsed data, and all data attributed to a polygon were resampled to points. The final cleaned and vetted data were used as input data for the modelling.

The processing of raw survey microdata for input in the model is shown in the flowchart below:


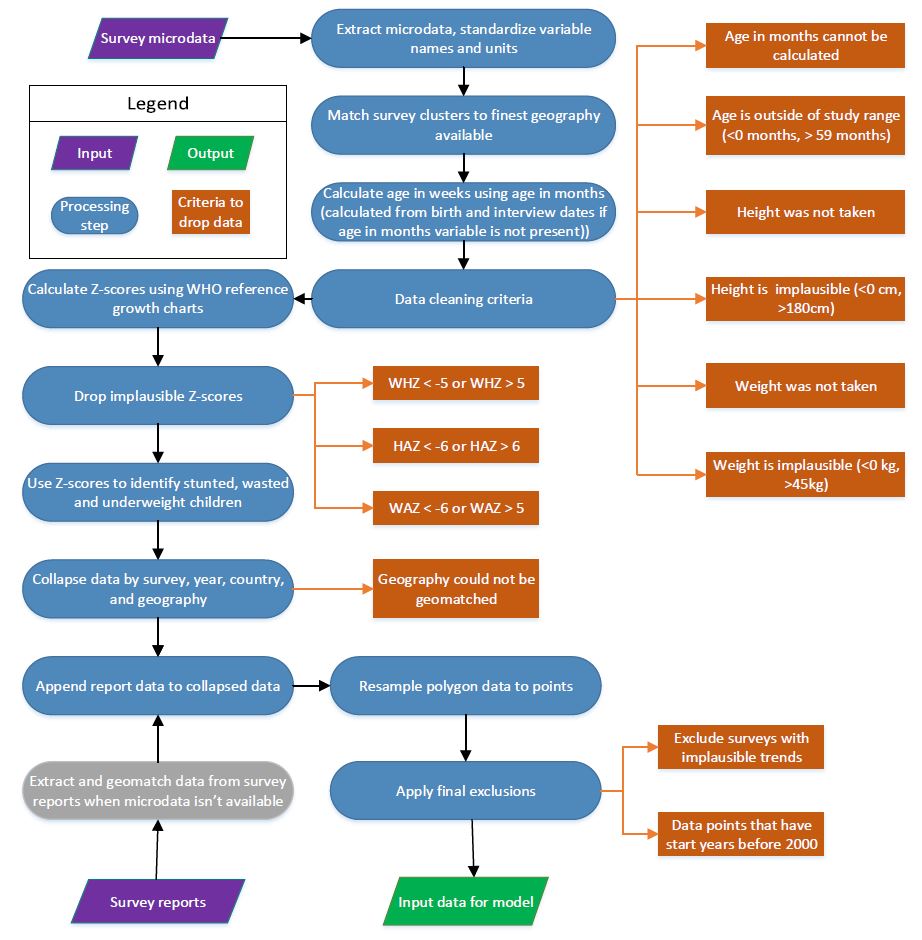


**Covariates**

A variety of socioeconomic and environmental variables were used to predict CGF outcomes. These covariates were selected on the basis of their potential to be predictive for the set of CGF indicators, after reviewing literature on evidence and plausible hypotheses as to their influence. Where available, the finest spatiotemporal resolution of gridded data sets was used. Of the 12 covariates included, eight were temporally dynamic and were reformatted as a synoptic mean over each estimation period or as a mid-period year estimate: these covariates included average daily mean rainfall (precipitation), average daily mean temperature, Enhanced Vegetation Index (EVI), fertility, malaria incidence, educational attainment in women of reproductive age (15–49 years old), population, and urbanicity. The remaining four covariate layers were static throughout the study period and were applied uniformly across all modelling years; growing season length, irrigation, nutritional yield for vitamin A, and travel time to nearest settlement >50,000 inhabitants. In addition to the covariates detailed below, some country-level variables such as lag distributed income per capita and the proportion of the population with access to adequate sanitation, were included in models for stunting, wasting and underweight.

**Covariates used in mapping**

| **Covariate** | **Temporal resolution** | **Source** | **Reference** |
| --- | --- | --- | --- |
| Average daily mean rainfall (Precipitation), Average daily mean temperature | Annual | CRUTS | Harris IP, Jones PD, Osborn TJ, Lister DH. Updated high‐resolution grids of monthly climatic observations–the CRU TS3. 10 Dataset. *Int J Climatol*. 2014; 34: 623-42.  University of East Anglia. Climatic Research Unit TS v. 3.24 dataset. https://crudata.uea.ac.uk/cru/data/hrg/cru_ts_3.24.01/. (accessed July 2017) |
| Enhanced Vegetation Index (EVI) | Annual | MODIS | Huete A, Justice C, Van Leeuwen W. MODIS vegetation index (MOD 13) algorithm theoretical basis document (ATBD) Version 3.0. EOS Project Office. 1999; 2.  Weiss DJ, Atkinson PM, Bhatt S, Mappin B, Hay SI, Gething PW. An effective approach for gap-filling continental scale remotely sensed time-series. *ISPRS J Photogramm Remote Sens*. 2014; 98: 106-18. |
| Fertility | Annual | WorldPop (derived) | Lloyd CT, Sorichetta A, Tatem AJ. High resolution global gridded data for use in population studies. *Sci. Data*. 2017; 4: 170001. |
| Growing season length | Static | FAO | Food and Agriculture Organisation of United Nations. GAEZ – Global Agro-Ecological Zones data portal. http://www.fao.org/nr/gaez/about-data-portal/en/ (accessed July 2017). |
| Irrigation | Static | University of Frankfurt | Goethe-Universität. Generation of a digital global map of irrigation areas. https://www.uni-frankfurt.de/45218039/Global_Irrigation_Map. |
| Malaria incidence | Annual | Malaria Atlas Project | Bhatt S, Weiss DJ, Cameron E, Bisanzio D, Mappin B, Dalrymple U et al. The effect of malaria control on plasmodium falciparum in Africa between 2000 and 2015. *Nature* 2015; 526: 207–211. |
| Educational attainment in women of reproductive age (15-49 years old) | Annual | Institute for Health Metrics and Evaluation, University of Washington | Local Burden of Disease Educational Attainment Collaborators. Mapping persistent local disparity in educational attainment across low- and middle-income countries. *Nature* 2020; 577: 235-238 |
| Nutritional yield for vitamin A | Static | Herrero et al (modeled) | Herrero M, Thornton PK, Power B, Bogard JR, Remans R, Fritz S, et al. Farming and the geography of nutrient production for human use: a transdisciplinary analysis. *Lancet Planetary Health*. 2017; 1: e33-42. |
| Population | Annual | WorldPop | Herrero M, Thornton PK, Power B, Bogard JR, Remans R, Fritz S, et al. Farming and the geography of nutrient production for human use: a transdisciplinary analysis. *Lancet Planetary Health*. 2017; 1: e33-42. |
| Travel time to nearest settlement >50,000 inhabitants | Static | Big Data Institute, Nuffield Department of Medicine, University of Oxford | Weiss DJ, Nelson A, Gibson HS, Temperley W, Peedell S, Lieber A, et al. A global map of travel time to cities to assess inequalities in accessibility in 2015. *Nature* 2018; 553: 333–6. |
| Urbanicity | Annual | European Commission/GHS | Pesaresi M, Ehrlich D, Ferri S, Florczyk A.J, Freire S, Halkiaet M, et al. Operating procedure for the production of the Global Human Settlement Layer from Landsat data of the epochs 1975, 1990, 2000, and 2014; JRC Technical Report EUR 27741 EN; 2016. |

**Statistical model**

***Seasonality adjustment***

WHZ were used to calculate an individual child wasting status. As a data preprocessing step, a seasonality adjustment was performed on individual-level child weights in order to account for differences in observed child weight that may have been due to food scarcity around the month in which the survey was conducted. To adjust weight measurements, a model was fit with a 12-month seasonal spline, a country-level fixed effect, and a smooth spline over the duration of data collection using the *mgcv* package in R and the following formula:

$$WHZ \sim s_{cc}\left( month \right)+ s_{tp}\left( t \right)+ as.factor\left( country \right).$$

$WHZ$ is a child’s weight-for-height z-score, $month$ is the integer-valued month of the year (1, …, 12),$t$ is the time of the interview in integer months since the earliest observation of any child in the dataset, and $country$ is a factor variable representing the country where the observation was recorded. The periodic component was modeled on months using 12 cyclic cubic regression splines basis functions ($cc)$ and a smooth longer time temporal trend was accounted for by using four thin-plate splines ($tp)$. The country effects and the long-term temporal spline were included only to help avoid confounding during fitting of the seasonal spline fit neither country effects nor the long-term trend were used in the seasonal adjustment. All observations were the adjusted to account for the difference in the seasonal period between the month of the interview and an average day of the year as determined by which days align with the mean of the periodic spline.

**Geostatistical model**

***Model geographies***

A total of three sub-models were run for each CGF indicator. The South Asia region was determined based on both proximity and epidemiological similarity. All data within the spatial region, and within a one-degree buffer from the boundaries of the region, were included in the model to minimise edge effects.

**Polygon resampling**

All data were matched to global positioning system (GPS) coordinates (latitude and longitude) wherever possible. These precisely located data are referred to as ‘point data’. In cases where point data were not available, data points were matched to the smallest possible areal unit (also referred to as polygons). In most cases these polygons represent administrative sub-divisions. Since the geostatistical model fitted requires point data, polygon data were re-sampled into pseudo-point data. This approach has been used before in mapping studies of child mortality^14^, child growth failure^15^, education^16^, and diarrhea burden.^17^

The approach to producing pseudo-points proceeded as follows for each polygon-level observation: 10,000 locations with weights proportional to the underlying population (as measured by WorldPop) were sampled at 1x1-km spatial resolution. k-means clustering were then used to derive a reduced set of points, with k set to 1 per 1000 grid cells. Each of these resulting clusters then served as pseudo-point data. Each pseudo-point was assigned a weight proportional to the number of sampled locations contained in it via k-means clustering. The observed prevalence for the polygon as a whole was assigned to each point, and the sample size for each point was taken as the sample size for the polygon multiplied by the weight. The sample sizes for all pseudo-points derived from a polygon thus sum to equal the sample size of the polygon as a whole.

***Ensemble covariate modelling***

An ensemble covariate modelling method was implemented in order to select covariates and capture possible non-linear effects and complex interactions between them.^18^ Three sub-models were fit to the dataset, using all covariate data as explanatory predictors: generalised additive models (GAM), boosted regression trees (BRT), and lasso regression. Country-level fixed effects were also included in the BRT model as dummy-coded covariates. Sample weights were used in sub-models, where applicable, such that cluster locations with latitude and longitude had a sample weight of 1, while cluster locations where the latitude and longitude were generated by the polygon resampling process had a weight based on the K-means clustering process.

Each sub-model’s predictive performance was tested using five-fold cross-validation to avoid overfitting. 20% of the data were removed to create five out-of-sample predications and were compiled into a single comprehensive set of predictions. Additionally, the same sub-models were also run using 100% of the data, and a full set of in-sample predictions were created. The five sets of out-of-sample sub-model predictions were fed into the full geostatistical model as the explanatory covariates when performing the model fit. The in-sample predictions from the sub-models were used as the covariates when generating predictions using the fitted full geostatistical model. A recent study has shown that this ensemble approach can improve predictive validity by up to 25% over an individual model.^18^

Predictions from each sub-model are generated based on patterns and relationships between the raw covariates and prevalence survey data, while predictions from the full geostatistical model are generated based on patterns and relationships between the predictions from the ensemble of sub-models and prevalence survey data. To discover the relationships between the sub-model prediction layers (used as covariates in the full geostatistical model) and the prevalence data, the only values of the covariates (sub-model prediction layers) “seen” by the model are the values underlying the locations of surveys. As such, it is possible that estimates will be generated in areas where the values of the covariates exceed the minimum and maximum values observed by the model. In these areas, the estimates are generated by extrapolating from the patterns observed within the range of covariates underlying the survey data.

The primary goal of using the stacking procedure in the analyses was to maximise the predictive power of the raster covariates by capturing the non-linear effects and complex interactions between covariates to optimise the model performance. Bhatt et al. (2017)^18^ contend that the primary purpose of the sub-model predictions is to improve the mean function of the Gaussian process.

***Model description***

Binomial count data are modeled within a Bayesian hierarchical modelling framework using a logit link function and a spatially and temporally explicit hierarchical generalised linear regression model to fit prevalence of each of CGF indicators.^19^ The hierarchy that defines the Bayesian method is explicitly written.

For each binomial CGF indicator, the prevalence of children stunted, wasted or underweight in each survey cluster, d was modelled. Survey clusters are precisely located by their GPS coordinates and year of observation, which was mapped to a spatial raster location i at time t. The number of children reported to be stunted, wasted or underweight, respectively, were observed as binomial count data $C_{d}$ among an observed sample size $N_{d}$. If multiple data clusters were observed within a given location i at time t, the probability of stunting, p, within a given cluster d was referred by its indexed location i and time t as p_i(d), t(d)_.

$$logit\left( p_{i,t} \right)= \beta_{0}+\boldsymbol{X}_{i,t}\boldsymbol{\beta+}Z_{i,t}+\epsilon_{ctr(i)}+\epsilon_{i,t} \forall i\in spatial domain \forall t\in time domain$$

$$\sum_{h=1}^{3} \beta_{h} =1$$

$$\boldsymbol{\epsilon}_{\boldsymbol{ctr}}\sim iid Normal\left( 0, \gamma^{2} \right)$$

$$\epsilon_{i,t}\sim iid Normal\left( 0, \sigma^{2} \right)$$

$\boldsymbol{Z}\sim GP(0, \Sigma^{space}\otimes\Sigma^{time}$*)*

$$\Sigma^{space}= \frac{\omega^{2}}{\Gamma\left( \nu\right)2^{v-1}}\times\left( \kappa D \right)^{\nu}\times K_{\nu}\left( \kappa D \right)$$

$$\Sigma_{j, k}^{time}=\rho^{\left| k-j \right|}$$

For indices $d$, $i$, and $t$, *(index) is the value of * at that index. The probabilities $p_{i,t}$ represent both the annual prevalence at the space-time location and the probability that an individual child was afflicted with the risk factor given that they lived at that particular location. The annual prevalence $p_{i,t}$ of each indicator was modeled as a linear combination of the three sub-models $h=3,$ GAM, BR), and lasso regression), rasterised covariate values $X_{i,t}$, a correlated spatiotemporal error term $Z_{i,t}$, country random effects $\epsilon_{ctr(i)}$ with one unstructured country random effect fit for each country in the modelling region and all $\boldsymbol{\epsilon}_{\boldsymbol{ctr}}$ sharing a common variance parameter,$\gamma^{2}$, and an independent nugget effect $\epsilon_{i,t}$ with variance parameter $\sigma^{2}$. Coefficients in the $\boldsymbol{\beta}$ vector represent their respective predictive weighting in the mean logit link, while the joint error term $Z_{i,t}$ accounts for residual spatiotemporal autocorrelation between individual data points that remains after accounting for the predictive effect of the sub-model covariates, the country-level random effect $\epsilon_{ctr(i)}$ , and the nugget independent error term,$\epsilon_{i,t}$. The residuals $Z_{i,t}$ are modeled as a three-dimensional Gaussian process ($GP$) in space-time centered at zero and with a covariance matrix constructed from a Kronecker product of spatial and temporal covariance kernels. The spatial covariance $\Sigma^{space}$ is modeled using an isotropic and stationary Matérn function^20^, and temporal covariance $\Sigma^{time}$ as an annual autoregressive (AR1) function over the 18 years represented in the model. In the stationary Matérn function, $\Gamma$ is the Gamma function, $K_{v}$ is the modified Bessel function of order $v>0, k>0$ is a scaling parameter, $D$ denotes the Euclidean distance, and $\omega^{2}$ is the marginal variance. The scaling parameter, $k$, is defined to be ${k=\sqrt{8v}}/\delta$ ^21,22^ where $\delta$ is a range parameter (which is about the distance where the covariance function approaches 0.1) and $v$ is a scaling constant, which is set to 2 rather than fit from the data. This is a notoriously hard parameter to reliably fit, and was set to 2 in many other analyses.^22-24^ The dimension of the spatial Matérn covariance matrix is equal to the number of spatial mesh points for a given modelling region squared. In the AR1 function, $\rho$ is the autocorrelation function (ACF), and $k$ and $j$ are points in the time series where $\left| k-j \right|$ defines the lag. The dimension of the AR1 covariance matrix is equal to the number of temporal mesh points (18) squared. The dimension of the space-time covariance matrix $(0, \Sigma^{space}\otimes\Sigma^{time}$*)* for a given modelling region is equal to (the number of spatial mesh points times the number of temporal mesh points)^2.

This approach leveraged the data’s residual correlation structure to more accurately predict prevalence estimates for locations with no data, while also propagating the dependence in the data through to uncertainty estimates.^9^ The posterior distributions were fit using computationally efficient and accurate approximations in R-INLA^25,26^ (integrated nested Laplace approximation) with the Stochastic Partial Differential Equations (SPDE)^21^ approximation to the Gaussian process residuals using R project version 3.5.1. The SPDE approach using INLA has been demonstrated elsewhere, including the estimation of health indicators, particulate air matter, and population age structure.^14,15,27-29^ Uncertainty intervals (UIs) were generated from 1000 draws (i.e., statistically plausible candidate maps)^30^ created from the posterior-estimated distributions of modelled parameters.

***Priors***

The following priors were used for all three CGF models:

$$\beta_{0}\sim N\left( {\mu=0,\sigma^{2}=3}^{2} \right),$$

$$\boldsymbol{\beta}\sim^{iid}N\left( {\mu=\frac{1}{\# ensemble models},\sigma^{2}=3}^{2} \right),$$

$log\left( \frac{1+\rho}{1-\rho} \right)\sim N(\mu=0,\sigma^{2}=1/0.15)$,

$$log\left( \frac{1}{\sigma_{nug}^{2}} \right)\sim loggamma\left( \alpha=1, \gamma=2 \right).$$

$$\theta_{1}=\log\left( \tau\right)\sim N(\mu_{\theta_{1}},\sigma_{\theta_{1}}^{2})$$

$\theta_{2}=\log\left( \kappa\right)\sim N(\mu_{2},\sigma_{\theta_{2}}^{2})$.

Given that the covariates used in INLA (i.e. the predicted outputs from the ensemble models) should be on the same scale as the predictive target, it is believed that the intercept in the model should be close to zero and that the regression coefficients should sum to 1. As such, the prior for the intercept was chosen to be $N(0, \sigma^{2}=3^{2})$, and the prior for the fixed effect coefficients to be $N(\frac{1}{\# ensemble models}, \sigma^{2}=3^{2})$. The prior on the temporal correlation parameter, $\rho$, is chosen to be mean zero, showing no prior preference for either positive or negative auto-correlation structure, and with a distribution that is wide enough such that within three standard deviations of the mean, the prior includes values of $\rho$ ranging from -0.95 to 0.95. The priors on the random effect variances were chosen to be relatively loose given that we believe our fixed effects covariates should be well-correlated with the outcome of interest, which might suggest relatively small random effects values. At the same time, to avoid using a prior that was so diffuse as to actually put high prior weight on large random effect variances. For stability, the uncorrelated multivariate normal priors were used that INLA automatically determines (based on the finite elements mesh) for the log-transformed spatial hyperparameters $\kappa$ and $\tau$. In parameterisation, $\alpha$ and $\gamma$ were represented in the *log(gamma)* distribution as scale and shape, respectively. Spatial hyperparameter priors: $\boldsymbol{\mu}_{\boldsymbol{\theta}_{\mathbf{1}}}=0.122237$, $\boldsymbol{\sigma}_{\boldsymbol{\theta}_{\mathbf{1}}}^{\boldsymbol{2}}=10$, $\boldsymbol{\mu}_{2}=-1.38775$, $\boldsymbol{\sigma}_{\boldsymbol{\theta}_{\boldsymbol{2}}}^{2}=10$

***Model fitting and estimate generation***

Models were fit in INLA with methods consistent with those used in geospatial modelling of CGF, under-5 mortality, and educational attainment in Africa, published previously.^15,16,31^

Where possible, the point data (GPS-positioned data) were used in the analyses. In instances where this was not possible, the data were matched to the smallest possible areal unit. The areal data were then resampled to generate pseudo-point data based on the underlying population distribution within the polygon. The methods for the resampling are consistent with those previously used in geospatial modelling of under-5 mortality.^31^ Resampling K-means weights were used within the INLA fit by multiplying the corresponding log likelihood evaluation for the specific observation by the observation’s K-means weight. These weights are used to ensure that the amount of information were not artificially inflated in the dataset by effectively using them to inflate the dispersion in the log likelihood for resampled polygon points. This is analogous to how weighting is often done in generalized additive models.^32^ Data points that could be georeferenced to latitude-longitude locations were assigned a weight of 1, ensuring that when the log-likelihood contribution from that observation was evaluated it contributed only to the log-likelihood at the observation’s space time location. For cluster locations generated based on the polygon resampling process, the log-likelihood of those points contributed proportionate to the K-means weights, effectively diffusing the evaluation of the observation across the polygon.

As part of the ensemble modelling process, prediction surfaces from the out of sample ensemble sub-models were used as covariates in the spatiotemporal model. Estimates of the fixed effects beta coefficients derived from the contribution of each of the sub-models to INLA’s predicted prevalence estimates, in conjunction with parameter estimates of the contribution of location and time. To create final estimates, the in-sample prediction surfaces of prevalence from the sub-models (serving as covariates) were used as covariates in conjunction with the fitted random effects from INLA to predict and calculate estimates of prevalence for each grid cell in each year.

The implementation of INLA using the R-INLA software relies on a Gaussian approximation of the full conditional distribution of latent variables, and uses the empirical Bayes approximation for the hyperparemeters.^24^ The full hyper parameter grid integration and CCD integration were tried in various settings and have found these models to be nearly indistinguishable. Due to its computing resource, the empirical Bayes procedure was used. In a similar setting, with malaria household survey data, the INLA results were directly compared with results from Hamiltonian Markov Chain Monte Carlo and nearly identical results were found between the two fits.^33^

All estimates were generated by taking 1000 draws from the posterior distribution, which yielded 1000 candidate maps used to summarise the grid cell and aggregated-level statistics. For estimates at the grid cell level, these draws were used directly to generate estimates and uncertainty. Aggregated estimates, in which grid cell level estimates were summarised to administrative boundaries, were generated by creating population-weighted averages for each administrative boundary, for each draw. 95% uncertainty intervals around the mean of estimates were generated.

**Model results**

Lower, median and upper quantiles (percentiles 0.025, 0.50, 0.975) are displayed for the main parameters from the stunting, wasting and underweight models. The fixed effects covariates corresponding to the predicted ensemble rasters are shown in the first five columns, while fitted values for the spatiotemporal field hyperparameters and the precisions (inverse variance) for our random effects are shown in the last five columns.

**Fitted parameters for stunting, wasting and underweight**

| **Indicator** | **Percentiles** | **int** | **gam** | **gbm** | **lasso** | **Nominal Range** | **Nominal Variance** | **AR1 rho** | **Precision for IID.ID** | **Precision for CTRY.ID** |
| --- | --- | --- | --- | --- | --- | --- | --- | --- | --- | --- |
| **Stunting** | **0.025** | -0.57 | -0.06 | 0.19 | 0.54 | 4.8 | 0.09 | 0.91 | 0.05 | 0.57 |
|  | **0.5** | -0.24 | 0.05 | 0.29 | 0.66 | 6.29 | 0.13 | 0.95 | 0.04 | 0.25 |
|  | **0.975** | 0.09 | 0.15 | 0.38 | 0.79 | 8.44 | 0.19 | 0.97 | 0.03 | 0.12 |
| **Wasting** | **0.025** | -0.41 | -0.08 | 0.44 | 0.21 | 3.03 | 0.07 | 0.55 | 0.06 | 0.42 |
|  | **0.5** | -0.13 | 0.05 | 0.59 | 0.37 | 4.02 | 0.10 | 0.76 | 0.05 | 0.19 |
|  | **0.975** | 0.14 | 0.17 | 0.73 | 0.52 | 5.33 | 0.13 | 0.86 | 0.04 | 0.09 |
| **Underweight** | **0.025** | -0.53 | -0.11 | 0.23 | 0.55 | 4.05 | 0.11 | 0.9 | 0.05 | 0.53 |
|  | **0.5** | -0.21 | -0.01 | 0.33 | 0.67 | 5.09 | 0.14 | 0.93 | 0.04 | 0.24 |
|  | **0.975** | 0.11 | 0.09 | 0.43 | 0.80 | 6.55 | 0.20 | 0.96 | 0.03 | 0.12 |

**District-level stunting prevalence and their 95% uncertainty intervals for 2017**


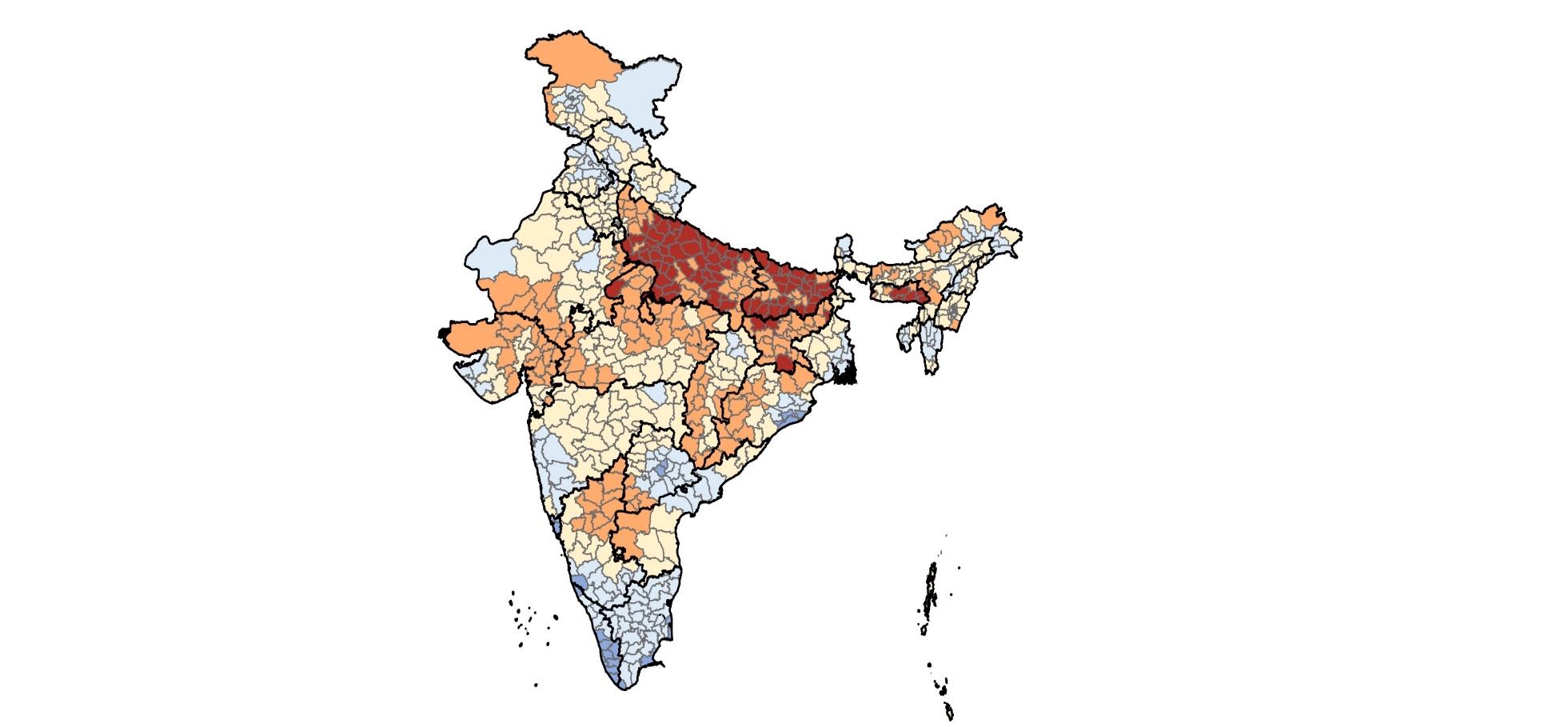


**Prevalence of stunting (%)**

48.0 or more

40.0-47.9

32.0-39.9

24.0-31.9

Less than 24.0


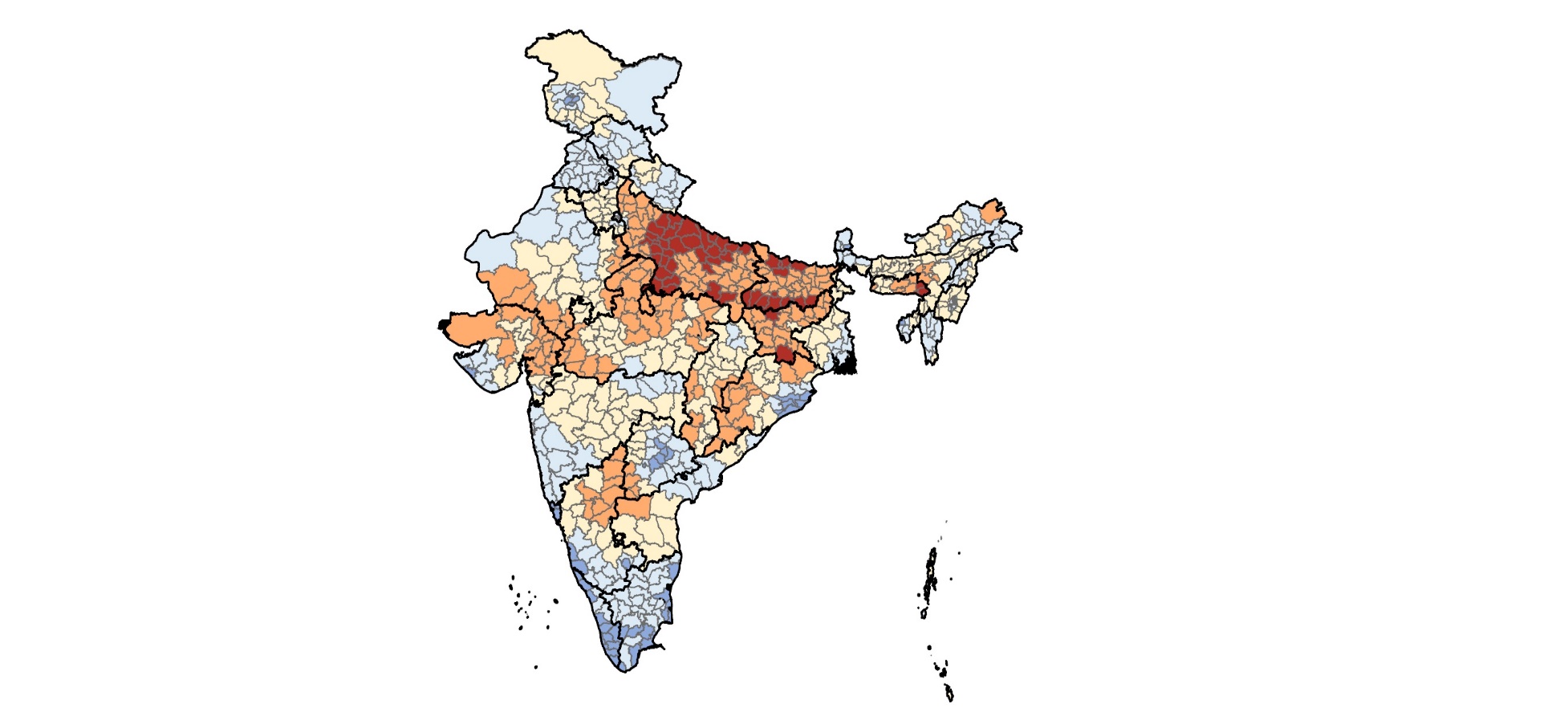

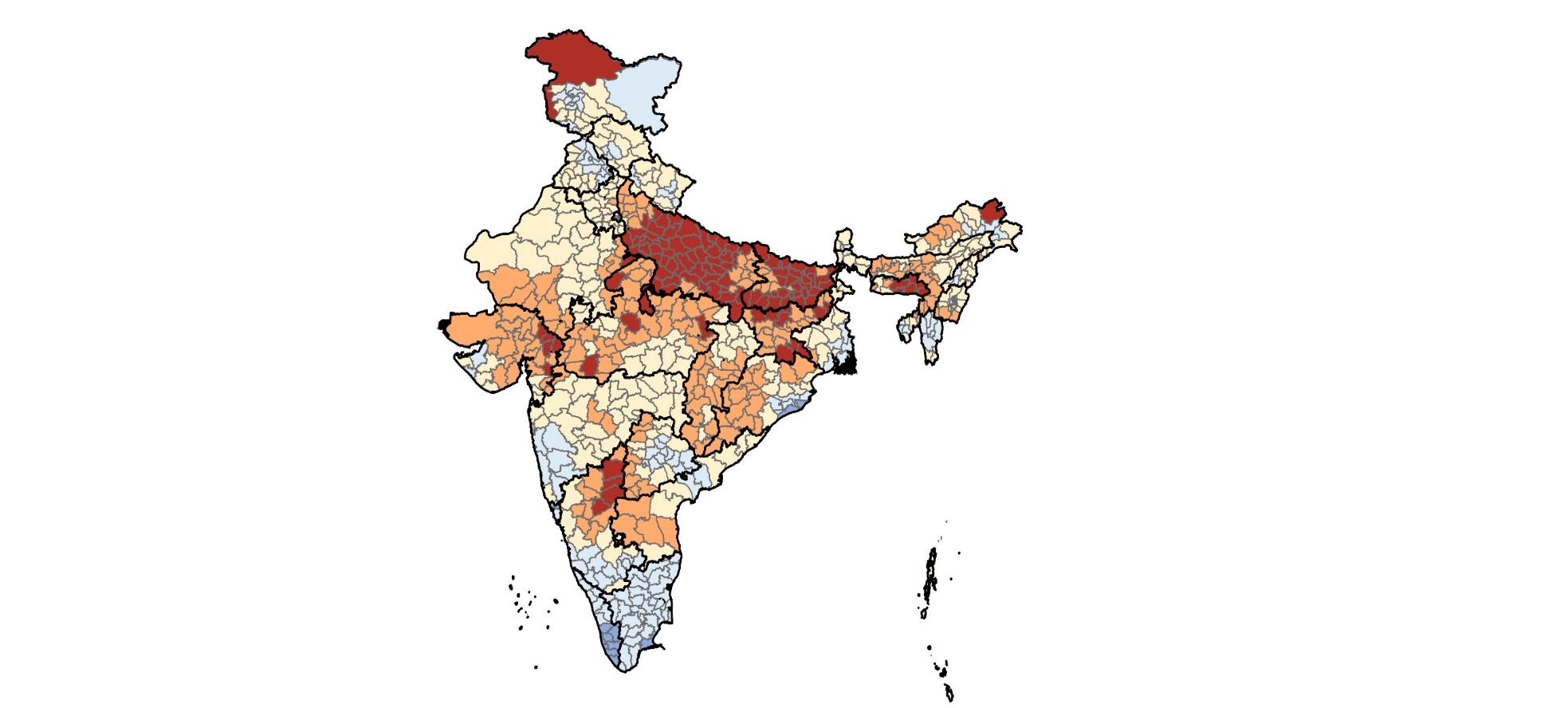


Prevalence

Lower UI

Upper UI

**District-level wasting prevalence and their 95% uncertainty intervals for 2017**


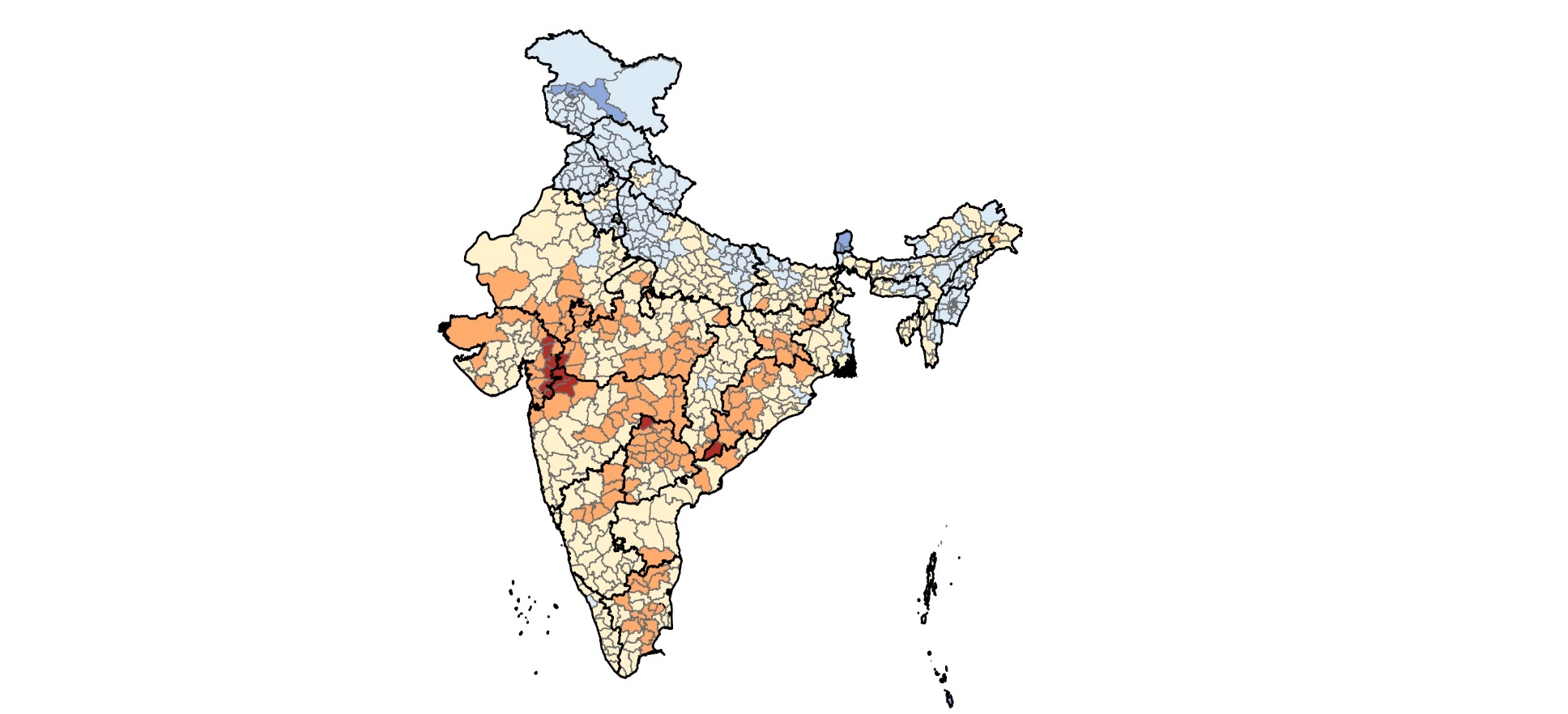


25.0 or more

19.0-24.9

13.0-18.9

7.0-12.9

Less than 7.0


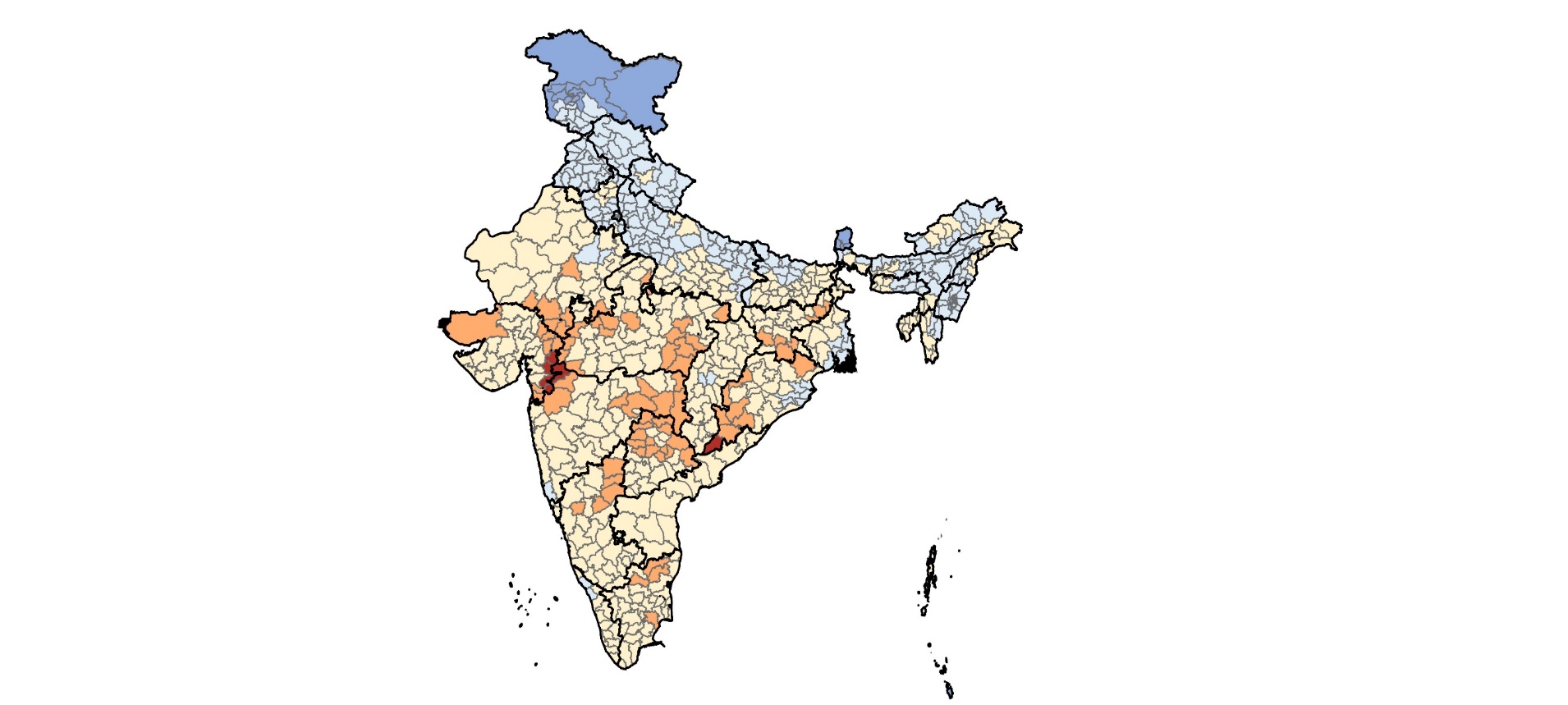

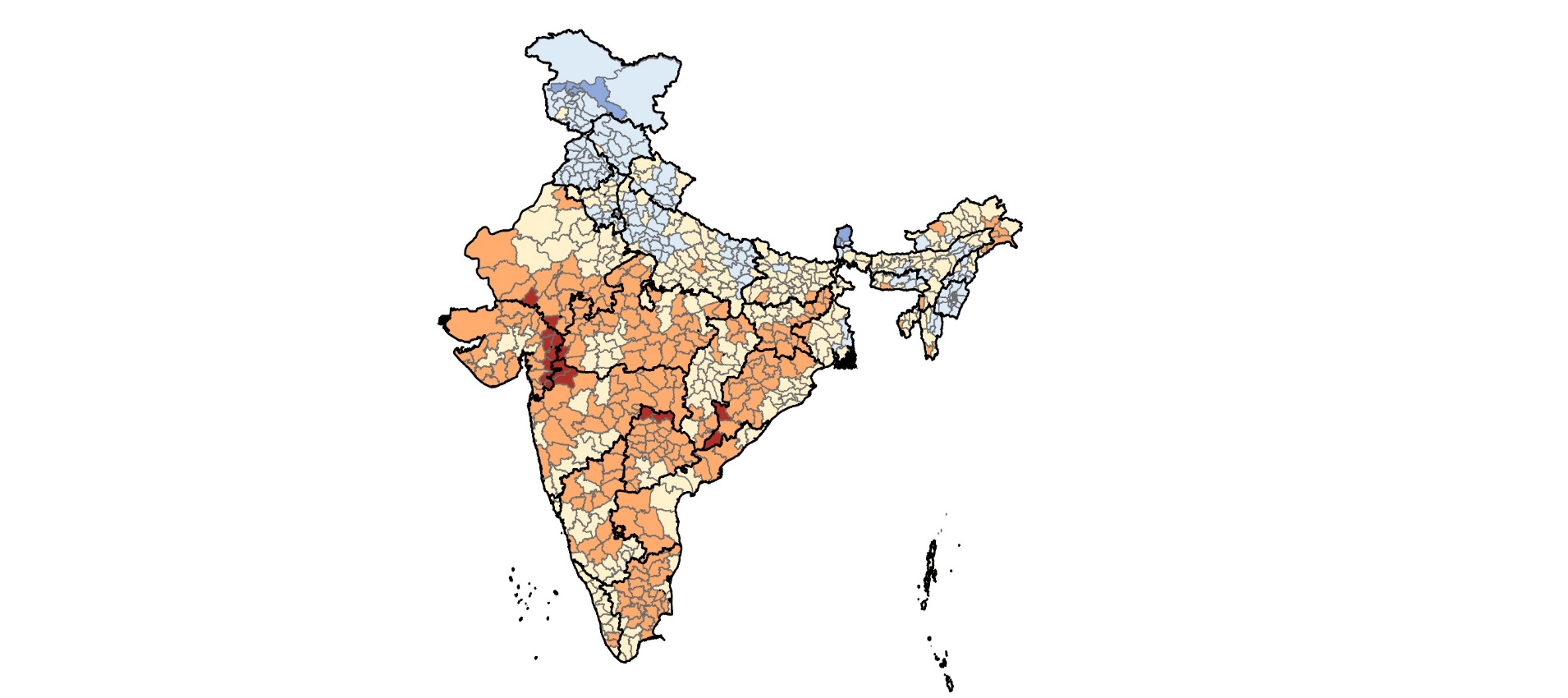


Lower UI

Upper UI

Prevalence

**Prevalence of wasting (%)**

**District-level underweight prevalence and their 95% uncertainty intervals for 2017**


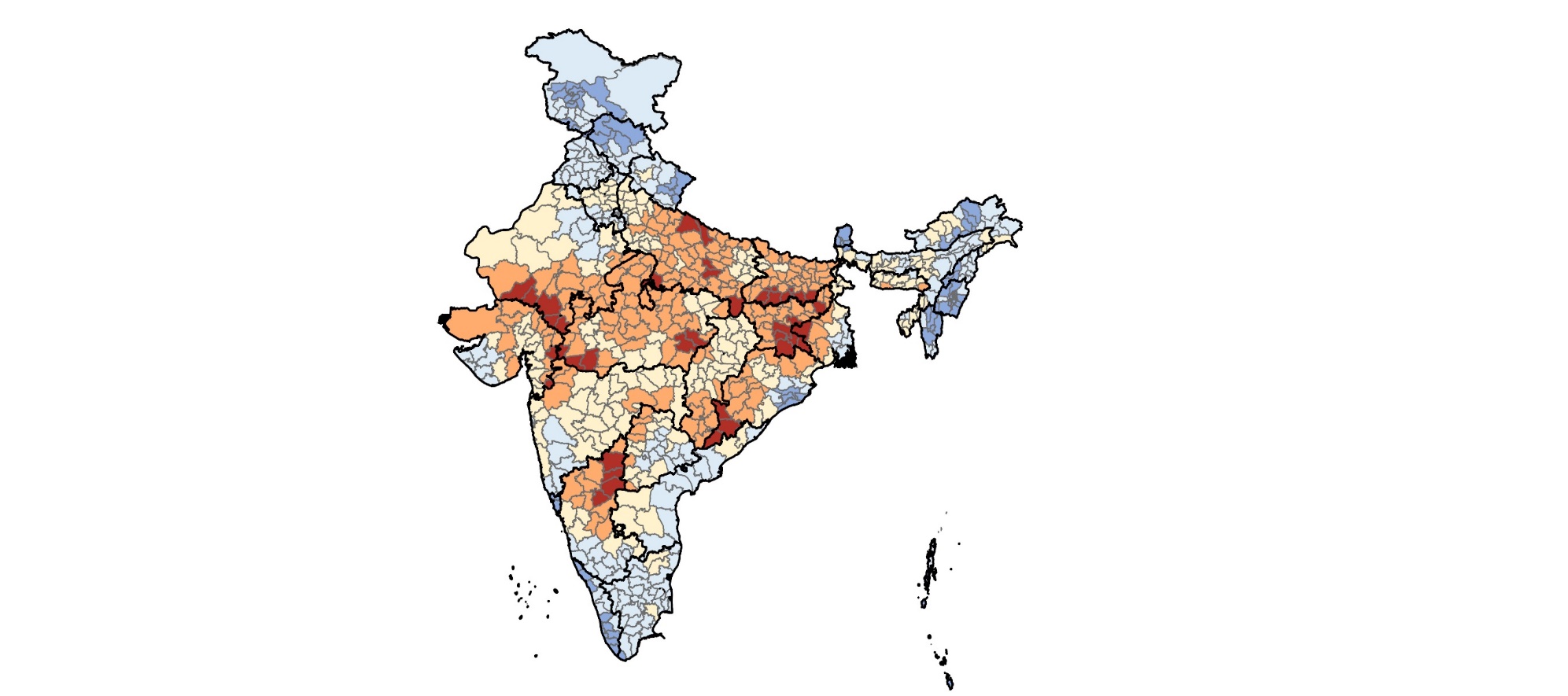


**Prevalence of underweight (%)**

42.5 or more

35.0-42.4

27.5-34.9

20.0-27.4

Less than 20.0


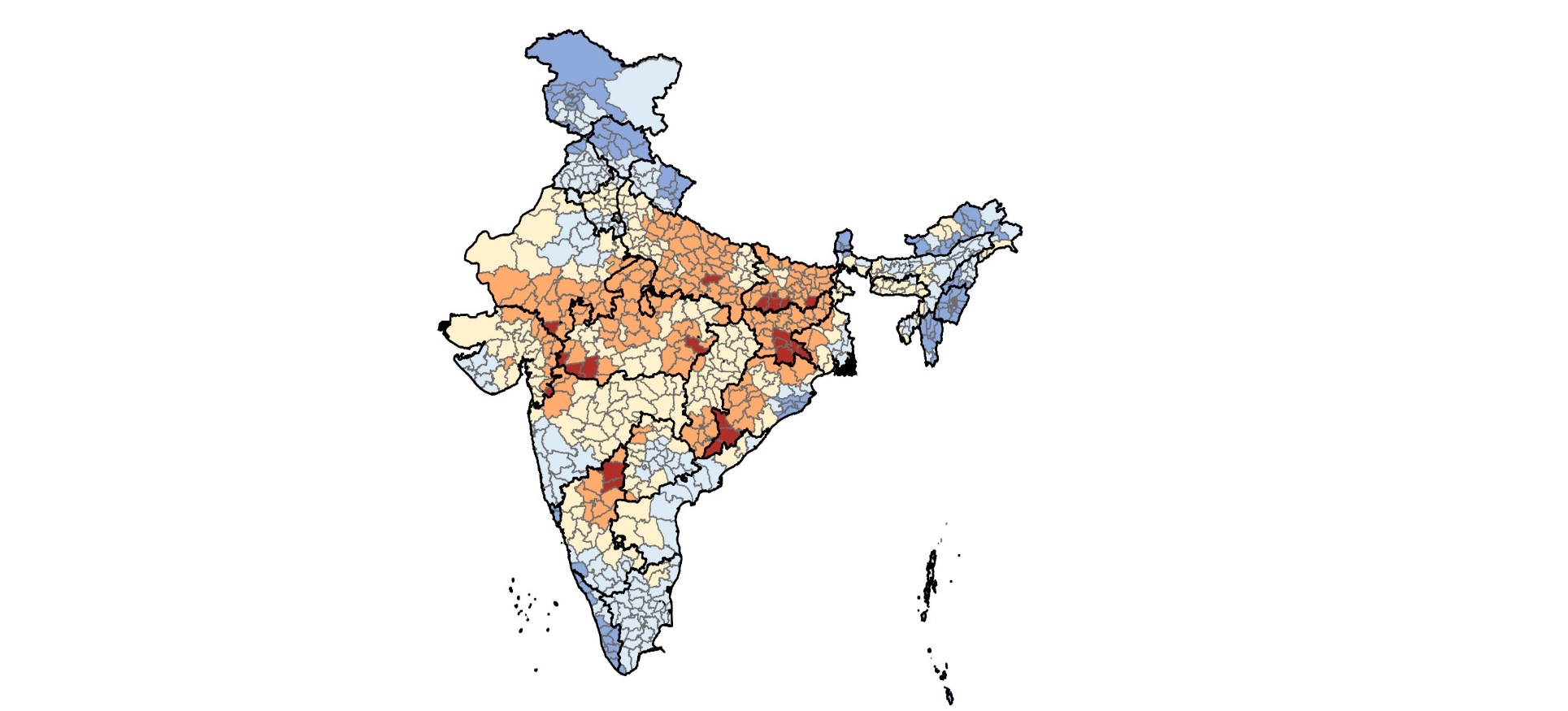

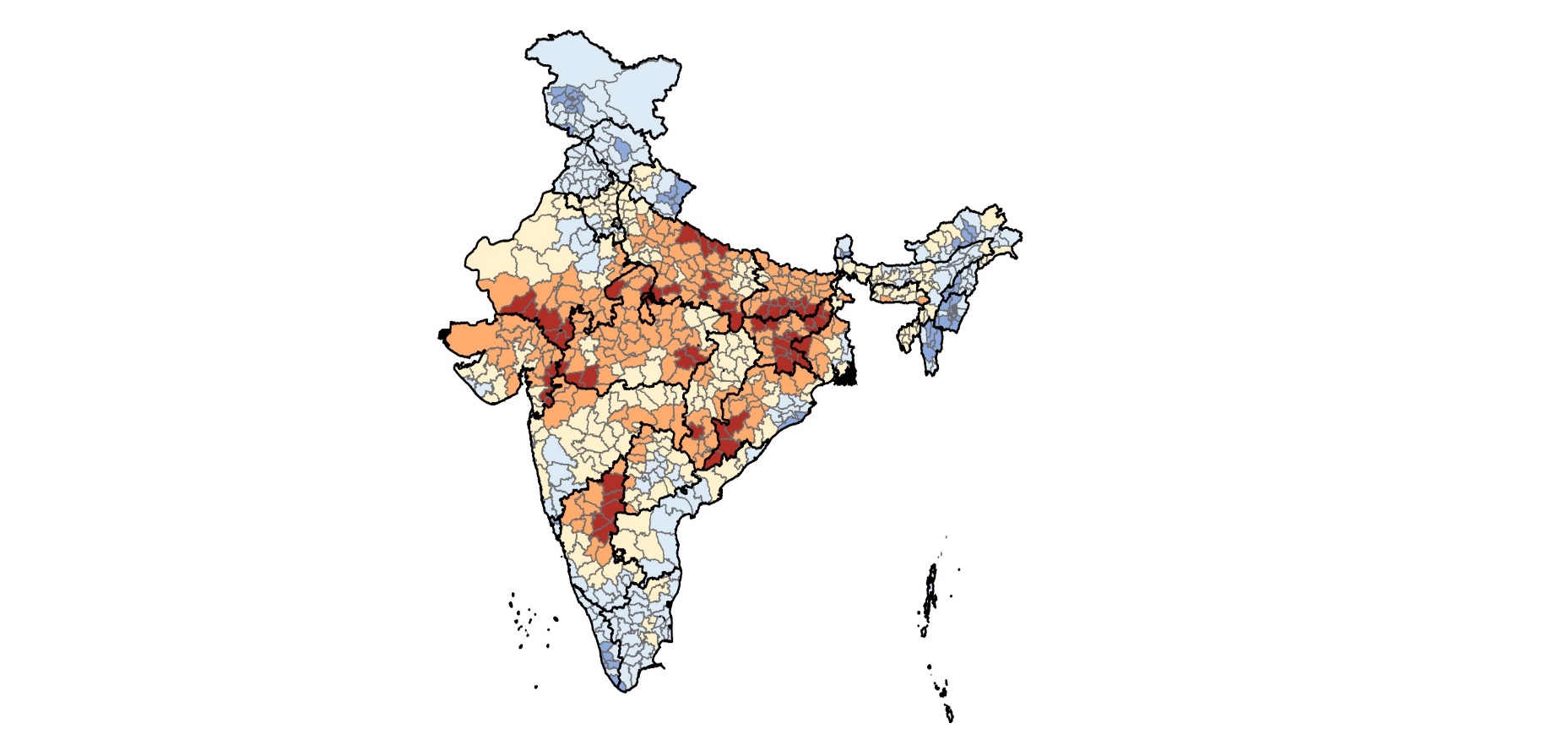


Prevalence

Lower UI

Upper UI

**Model validation**

**In-sample metrics**

In order to assess the in-sample performance of the models and compare to national-level estimates produced by GBD, a suite of diagnostic plots were generated for each CGF indicator estimates. To explore residual error over space and time, absolute error (data minus predicted posterior mean estimates at the corresponding grid cells) were produced.

**Metrics of predictive validity**

In order to assess the predictive validity of estimates, models were validated using spatially stratified five-fold out of sample cross-validation.^34^ To construct each spatial fold, a modified bi-tree algorithm was used to spatially aggregate data points. This algorithm recursively partitions two dimensional space, alternating between horizontal and vertical splits on the weighted data sample size medians, until the data contained within each spatial partition are of a similar sample size. The depth of recursive partitioning is constrained by the target sample size within a partition and the minimum number of clusters or pseudo-clusters allowed within each spatial partition (in this case, a minimum sample size of 500 was used). These spatial partitions are then allocated to one of five folds for cross validation. For validation, each geostatistical model was run five times, each time holding out data from one of the folds, generating a set of out-of-sample predictions for the held-out data. For each indicator, a full suite of out-of-sample predictions over the entire dataset was generated by combining the out of sample predictions from the five cross-validation runs.

Using these out-of-sample predictions, mean error (ME, or bias), root-mean-squared-error (RMSE, which summarises total variance), coefficient of variation (CoV, defined to be the standard deviation divided by the mean and multiplied by 100, which is a measure of relative variability), and 95% coverage of our predictive intervals (the proportion of observed out-of-sample data that fall within our predicted 95% credible intervals) aggregated up to different administrative levels (levels 0, 1 and 2) were calculated. Administrative level 0 (Admin 0) borders correspond to national boundaries, first administrative level (Admin 1) borders correspond to state-level boundaries within India, and second administrative level (Admin 2) borders correspond to the next finer unit-level, districts, within each states. Included in the sample tables for comparison are the same metrics calculated on in-sample predictions.

1. **Calibration with Global Burden of Disease 2017**

In order to leverage national-level data included in GBD 2017, but outside the scope of the current geospatial modelling framework, and to ensure calibration between these estimates and GBD 2017 estimates, a post hoc calibration was performed to each of the 1000 candidate maps. For India, the GBD produced estimates at the state-level.^34^ For each posterior draw, population-weighted grid cell aggregations were calculated at the state level and these estimates for each year were compared to the analogous GBD 2017 estimates from 2000 to 2017. The scaling factor for a given state and year was defined as the ratio between the GBD 2017 mean estimate and the aggregated geospatial mean estimate for that state and year. Finally, each of the grid cells falling within a given state and year was multiplied by its associated raking factor. This ensured calibration between the geospatial estimates and GBD 2017 estimates, while preserving the estimated geospatial and temporal variation across India.

**E.**  **Projections at the national and state-level**

GBD 2017 produced projections for the health-related Sustainable Development Goal indicators up to 2030 based on past trends, using a new advanced modelling framework.^35^ The steps used to produce projections for the CGF indicators are as follows.

For each CGF indicator, the annual change from the previous year was first calculated from 1990 to 2017 using the logit of the prevalence for each year. The weight for each year was calculated using this formula. Then annual rate of change was used to project the prevalence up to 2030 for India and states as part of GBD, giving higher weight to the more recent annual rate of change to project from 2018 to 2030.

$${weight}_{year}=({t-1990+1)}^{\omega}$$

Where ω is the weight function, the value of which denotes how much higher impact recent years would have compared with the past years when calculating the annual rate of change for the projection. To determine the appropriate value of ω for each indicator, an out-of-sample predictive validity test was done using data from 1990 to 2007 to predicted values for the years from 2008 to 2017. Assuming a range of values, in the increments of 0.25, from 0 to 10 for 𝜔, the best predicted value for the period 2008 to 2017 was tested for each indicator. The final value for the weight function (ω) specific to each indicator for projection was chosen that minimised the root mean squared error in the 2008–2017 projections based on the 1990–2007 data. Using this approach, the weight functions computed was 1.7 for stunting, 1.8 for underweight, and 1.9 for wasting.

The inverse of the weighted logit mean of the annualised rate of change from 1991 to 2017 was then applied to the years 2018 onward to estimate the prevalence of each CGF indicator up to 2030.

**F. Projections at the sub-state level**

To estimate the prevalence of CGF indicators in 2022 and 2030 at 5x5 km grids, a simple projection methodology that has been used previously for such geospatial analysis was performed using the estimated annual rates of change (AROCs) from 2000 to 2017 to obtain the estimates for subsequent years.^15~~14~~^

For each CGF indicator *u,* AROC was calculated at each grid cell m, by calculating the AROC between each pair of adjacent years t:

$${AROC}_{u,m,yr}=\mathrm{logit}\left( \frac{p_{u,m,t}}{p_{u,m,t-1}} \right)$$

A weighted AROC was calculated for each indicator–pixel by taking a weighted average across the years, where more recent AROCs were given more weight in the average. Weights were defined to be:

$$W_{t}={(t-2000+1)}^{\omega}$$

Where $\omega$ may be chosen to give varying amounts of weight across the years. For this set of projections, $\omega$ was selected as 1.7 for stunting, 1.9 for wasting, and 1.8 for underweight similar to that used by GBD. This weighting scheme would give high weightage to the change in the most recent years. For any indicator and for any pixel, the average AROC was calculated to be:

$$AROC_{u,m}=\mathrm{logit}\left( \sum_{2001}^{2017} W_{t}*AROC_{u,m,t} \right)$$

The raw projections to 2030 were then calculated by applying the annual rate of change estimates to 2017 estimates for each draw using the following projection calculation:

$$Proj_{u,m,y}=\mathrm{logi}t^{-1}\left( \mathrm{logit}\left( p_{u,m,2017} \right)+{AROC}_{u,m}\times(year-2017) \right).$$

The population-weighted aggregates of the prevalence from these pixel-level projections were created for the district level. The projections for the districts for 2022 and 2030 were computed using the mean, 2.5^th^ percentile, and 97.5^th^ percentiles of the draw estimates. The mean estimates were harmonized to the state and national level estimates produced by GBD by applying the relevant raking factor.

**G. Uncertainty intervals**

Point estimates for each quantity of interest were derived from the mean of the draws, while 95% uncertainty intervals (UIs) were derived from the 2.5^th^ and 97.5^th^ percentiles of the 1000 draw level values. Uncertainty in the estimation is attributable to sample size variability within data sources, different availability of data by age, sex, year, or location, and cause-specific model specifications. The UIs were determined for components of cause-specific estimation based on 1000 draws from the posterior distribution of cause specific mortality by age, sex, and location for each year included in the GBD 2017 analysis. With this approach, uncertainty could be quantified and propagated into the final quantities of interest.

**References**

1. WHO Multicentre Growth Reference Study Group, Onis M. WHO Child Growth Standards based on length/height, weight and age: WHO Child Growth Standards. *Acta Paediatrica* 2007;95: 76–85.
2. World Health Organisation. WHO Global Database on Child Growth and Malnutrition. http://www.who.int/nutgrowthdb/en/.
3. Wang Y, Chen HJ. Use of percentiles and Z -scores in anthropometry. Handbook of Anthropometry: Physical Measures of Human Form in Health and Disease. 2012; 29-48.
4. WHO, UNICEF. WHO child growth standards and the identification of severe acute malnutrition in infants and children: joint statement by the World Health Organization and the United Nations Children’s Fund. https://apps.who.int/iris/bitstream/handle/10665/44129/9789241598163_eng.pdf;jsessionid=CB1647B54282805D49317F58A3117B92?sequence=1.
5. Álvarez Uribe MC, López Gaviria A, Estrada Restrepo A. Concordance between Z scores from WHO 2006 and the NCHS 1978 growth standards of children younger than five. Antioquia-Colombia*. Perspectivas en Nutrición Humana* 2008; 10: 177-87.
6. Massey Jr FJ. The Kolmogorov-Smirnov test for goodness of fit. *Journal of the American Statistical Association* 1951; 46: 68–78.
7. Vasudevan S, Ramos F, Nettleton E, Durrant-Whyte H, Blair A. Gaussian Process modeling of large scale terrain. In: 2009 IEEE International Conference on Robotics and Automation. 2009: 1047–53.
8. Rasmussen CE, Williams CKI. Gaussian Processes for Machine Learning. Cambridge, Mass: The MIT Press, 2005.
9. Diggle P, Ribeiro Jr P. Model-based geostatistics. *Springer*; 2007.
10. Lumley T. Complex surveys: a guide to analysis using R. https://www.wiley.com/en us/Complex+Surveys%3A+A+Guide+to+Analysis+Using+R-p-9780470284308.
11. Lumley T. Analysis of complex survey samples. *J Stat Softw* 2004; 9:1–19.
12. Tatem AJ. Worldpop, open data for spatial demography. *Sci Data* 2017; 4: 170004.
13. Waterlow J, Buzina R, Keller W, et al. The presentation and use of height and weight data for comparing the nutritional status of groups of children under the age of 10 years. *Bull. World Health Organ* 1977;55: 489-98.
14. Golding N, Burstein R, Longbottom J, et al. Mapping under-5 and neonatal mortality in Africa, 2000–15: a baseline analysis for the Sustainable Development Goals. *Lancet* 2017; 390: 2171-82.
15. Osgood-Zimmerman A, Millear AI, Stubbs RW, et al. Mapping child growth failure in Africa between 2000 and 2015. *Nature* 2018; 555: 41.
16. Graetz N, Friedman J, Osgood-Zimmerman A, et al. Mapping local variation in educational attainment across Africa. *Nature* 2018; 555: 48.
17. Reiner Jr RC, Graetz N, Casey DC, et al. Variation in childhood diarrheal morbidity and mortality in Africa, 2000–2015. *New Eng J Med* 2018; 379: 1128-38.
18. Bhatt S, Cameron E, Flaxman SR, et al. Improved prediction accuracy for disease risk mapping using Gaussian process stacked generalization. *J R Soc Interface* 2017; 14: 20170520.
19. Murray CJ, Ezzati M, Flaxman AD, et al. GBD 2010: design, definitions, and metrics. *Lancet* 2012; 380: 2063-6.
20. Stein ML. Interpolation of spatial data. *Springer*; 1999.
21. Lindgren F, Rue H, Lindström J. An explicit link between Gaussian fields and Gaussian Markov random fields: the stochastic partial differential equation approach. *J R Stat Soc Ser B* 2011; 73: 423-98.
22. Lindgren F, Rue H. Bayesian spatial modelling with R-INLA. *J Stat Softw* 2015; 63: 1-25.
23. Rozanov YA. Markov random fields. *Springer;* 1982: 55-102.
24. Whittle P. On stationary processes in the plane. *Biometrika* 1954: 434-49.
25. Rue H, Martino S, Chopin N. Approximate Bayesian inference for latent Gaussian models by using integrated nested Laplace approximations. *J R Stat Soc Ser B* 2009; 71: 319-92.
26. Martins TG, Simpson D, Lindgren F, Rue H. Bayesian computing with INLA: new features. *Comput Stat Data An* 2013; 67: 68-83.
27. Cameletti M, Lindgren F, Simpson D, Rue H. Spatio-temporal modeling of particulate matter concentration through the SPDE approach. *Asta-adv Stat Anal* 2013; 97: 109-31.
28. Alegana VA, Atkinson PM, Pezzulo C, et al. Fine resolution mapping of population age-structures for health and development applications. *J R Soc Interface* 2015; 12: 20150073.
29. Kinyoki DK, Kandala N-B, Manda SO, et al. Assessing comorbidity and correlates of wasting and stunting among children in Somalia using cross-sectional household surveys: 2007 to 2010. *BMJ Open* 2016; 6: e009854.
30. Patil AP, Gething PW, Piel FB, Hay SI. Bayesian geostatistics in health cartography: the perspective of malaria. *Trends Parasitol* 2011; 27: 246-53.
31. Wood S. Mixed GAM computation vehicle with automatic smoothness estimation 2019. https://cran.r-project.org/web/packages/mgcv/mgcv.pdf.
32. Bhatt S, Weiss D, Cameron E, et al. The effect of malaria control on Plasmodium falciparum in Africa between 2000 and 2015. *Nature* 2015; 526: 207.
33. Roberts DR, Bahn V, Ciuti S, et al. Cross‐validation strategies for data with temporal, spatial, hierarchical, or phylogenetic structure. *Ecography* 2017; 40: 913-29.
34. India State-Level Disease Burden Initiative Malnutrition Collaborators. The burden of child and maternal malnutrition and trends in its indicators in the states of India: the Global Burden of Disease Study 1990–2017. *Lancet Child & Adolescent Health* 2019; 3: 855–70.
35. GBD 2017 SDG Collaborators. Measuring progress from 1990 to 2017 and projecting attainment to 2030 of the health-related Sustainable Development Goals for 195 countries and territories: a systematic analysis for the Global Burden of Disease Study 2017. *Lancet* 2018; 392: 2091–138.

# **2. Data inputs for child growth failure estimation, mapping and covariates for India**

| Abel R, Sampathkumar V. Tamil Nadu nutritional survey comparing children aged 0-3 years with the NCHS/CDC reference population. Indian J Pediatr. 1998; 65: 565-72. |
| --- |
| Awasthi S, Das R, Verma T, Vir S. Anemia and undernutrition among preschool children in Uttar Pradesh, India. Indian Pediatr. 2003; 40: 985-90. |
| Awasthi S, Pande VK. Cause-specific mortality in under-fives in the urban slums of Lucknow, north India. J Trop Pediatr. 1998; 44: 358-61. |
| Bhatt S, Weiss DJ, Cameron E, Bisanzio D, Mappin B, Dalrymple U et al. The effect of malaria control on plasmodium falciparum in Africa between 2000 and 2015. Nature, 2015; 526: 207–211 |
| Center for Vaccine Development, University of Maryland, Centers for Disease Control and Prevention, Department of Medical Microbiology and Immunology, Göteborg University, International Vaccine Institute, National Institute of Cholera and Enteric Diseases, Perry Point Cooperative Studies Program Coordinating Center, U.S. Department of Veterans Affairs, School of Medicine, University of Virginia, University of Chile. India - Kolkata Global Enteric Multicenter Study 2007-2011. Baltimore, MD, United States: Center for Vaccine Development, University of Maryland. |
| Center for Vaccine Development, University of Maryland, Centers for Disease Control and Prevention, Department of Medical Microbiology and Immunology, Göteborg University, International Vaccine Institute, National Institute of Cholera and Enteric Diseases, Perry Point Cooperative Studies Program Coordinating Center, U.S. Department of Veterans Affairs, School of Medicine, University of Virginia, University of Chile. India - Kolkata Global Enteric Multicenter Study 2011-2013. Baltimore, United States: Center for Vaccine Development, University of Maryland. |
| Das S, Bapat U, More NS, Alcock G, Fernandez A, Osrin D. Nutritional status of young children in Mumbai slums: a follow-up anthropometric study. Open Nutr J. 2012; 11: 100. |
| Department of Women and Child Development, Ministry of Human Resource Development, Government of India. India Nutrition Profile Survey 1995-1996. New Delhi, India: Ministry of Human Resource Development. |
| Desai, Sonalde, Reeve Vanneman, National Council of Applied Economic Research, University of Michigan. India Human Development Survey 2005. Ann Arbor, Michigan: Inter-University Consortium for Political and Social Research. |
| Food and Agriculture Organisation of United Nations. GAEZ – Global Agro-Ecological Zones data portal. http://www.fao.org/nr/gaez/about-data-portal/en/ (accessed July 2017). |
| Ford Foundation, Nutrition Foundation of India. India Profiles of Undernutrition and Underdevelopment: Studies of Poor Communities in Seven Regions of the Country. New Delhi, India: Nutrition Foundation of India. (Scientific Report Series No. 8). |
| Global Burden of Disease Health Financing Collaborator Network, Institute for Health Metrics and Evaluation (IHME). Global Development Assistance for Health, Government, Prepaid Private, and Out-of-Pocket Health Spending 1995-2014. Seattle, United States: IHME; 2017. |
| Goethe-Universität. Generation of a digital global map of irrigation areas. https://www.uni-frankfurt.de/45218039/Global_Irrigation_Map. |
| Goudar SS, Goco N, Somannavar MS, Vernekar SS, Mallapur AA, Moore JL, Wallace DD, Sloan NL, Patel A, Hibberd PL, Koso-Thomas M, McClure EM, Goldenberg RL. Institutional deliveries and perinatal and neonatal mortality in Southern and Central India. Reprod Health. 2015; 12: S13. |
| Local Burden of Disease Educational Attainment Collaborators. Mapping persistent local disparity in educational attainment across low- and middle-income countries. Nature 2020; 577: 235-238 |
| Harris IP, Jones PD, Osborn TJ, Lister DH. Updated high‐resolution grids of monthly climatic observations–the CRU TS3. 10 Dataset. International Journal of Climatology. 2014; 34: 623-42. |
| Herrero M, Thornton PK, Power B, Bogard JR, Remans R, Fritz S, et al. Farming and the geography of nutrient production for human use: a transdisciplinary analysis. The Lancet Planetary Health. 2017; 1: e33-42. |
| Hirve S, Ganatra B. A prospective cohort study on the survival experience of under five children in rural western India. Indian Pediatr. 1997; 34: 995-1001. |
| Huete A, Justice C, Van Leeuwen W. MODIS vegetation index (MOD 13) algorithm theoretical basis document (ATBD) Version 3.0. EOS Project Office. 1999; 2. |
| International Institute for Population Sciences (IIPS), Ministry of Health and Family Welfare, Government of India, Macro International. India National Family Health Survey Data (NFHS-1) 1992-1993. Mumbai, India: IIPS. |
| International Institute for Population Sciences (IIPS), Ministry of Health and Family Welfare, Government of India, Macro International. India National Family Health Survey (NFHS-1) 1992-1993: National Report. Mumbai, India: IIPS. |
| International Institute for Population Sciences (IIPS), Ministry of Health and Family Welfare, Government of India, ORC Macro. India National Family Health Survey Data (NFHS-2) 1998-1999. Mumbai, India: IIPS. |
| International Institute for Population Sciences (IIPS), Ministry of Health and Family Welfare, Government of India, ORC Macro. India National Family Health Survey (NFHS-2) 1998-1999: National and State Reports. Mumbai, India: IIPS. |
| International Institute for Population Sciences (IIPS), Ministry of Health and Family Welfare, Government of India, Macro International. India National Family Health Survey Data (NFHS-3) 2005-2006. Mumbai, India: IIPS. |
| International Institute for Population Sciences (IIPS), Ministry of Health and Family Welfare, Government of India, Macro International. India National Family Health Survey (NFHS-3) 2005-2006: National and State Reports. Mumbai, India: IIPS. |
| International Institute for Population Sciences (IIPS), Ministry of Health and Family Welfare, Government of India, ICF. India National Family Health Survey Data (NFHS-4) 2015-2016: Mumbai, India: IIPS. |
| International Institute for Population Sciences (IIPS), Ministry of Health and Family Welfare, Government of India, ICF. India National Family Health Survey (NFHS-4) 2015-2016: National and State Reports. Mumbai, India: IIPS. |
| International Institute for Population Sciences (IIPS), Ministry of Health and Family Welfare, Government of India, ICF. India National Family Health Survey (NFHS-4) 2015-2016: National and State-level Factsheets. Mumbai, India: IIPS. |
| International Institute for Population Sciences (IIPS), Ministry of Health and Family Welfare, Government of India. India District Level Household Survey Data (DLHS-2) 2002-2004. Mumbai, India: IIPS. |
| International Institute for Population Sciences (IIPS), Ministry of Health and Family Welfare, Government of India. India District Level Household Survey (DLHS-2) 2002-2004: National and State Reports. Mumbai, India: IIPS. |
| International Institute for Population Sciences (IIPS), Ministry of Health and Family Welfare, Government of India. India District Level Household and Facility Survey Data (DLHS-4) 2012-2014. Mumbai, India: IIPS. |
| International Institute for Population Sciences (IIPS), Ministry of Health and Family Welfare, Government of India. India District Level Household and Facility Survey (DLHS-4) 2012-2013: State Reports. Mumbai, India: IIPS. |
| International Institute for Population Sciences, World Health Organization. India World Health Survey 2003. |
| International Social Survey Programme (ISSP), GESIS - Leibniz Institute of Social Sciences. ISSP: Family and Changing Gender Roles IV, ZA5900 (Version 4.0.0). Mannheim, Germany: GESIS - Leibniz Institute of Social Sciences; 2012. |
| Kodkany BS, Derman RJ, Honnungar NV, Tyagi NK, Goudar SS, Mastiholi SC, Moore JL, McClure EM, Sloan N, Goldenberg RL. Establishment of a Maternal Newborn Health Registry in the Belgaum District of Karnataka, India. Reprod Health. 2015; 12 Suppl 2: S3. |
| Lloyd CT, Sorichetta A, Tatem AJ. High resolution global gridded data for use in population studies. Scientific Data. 2017; 4: 170001. |
| Mahapatra A, Geddam JJ, Marai N, Murmu B, Mallick G, Bulliyya G, Acharya AS, Satyanarayana K. Nutritional status of preschool children in the drought affected Kalahandi district of Orissa. Indian J Med Res. 2000; 111: 90-4. |
| Mathad V, Metgud C, Mallapur MD. Nutritional status of under-fives in rural area of South India. Indian J Med Sci. 2011; 65: 151-6. |
| Meshram II, Arlappa N, Balakrishna N, Laxmaiah A, Mallikarjun Rao K, Gal Reddy C, Ravindranath M, Sharad Kumar S, Brahmam GNV. Prevalence and determinants of undernutrition and its trends among pre-school tribal children of Maharashtra State, India. J Trop Pediatr. 2012; 58: 125-32. |
| Meshram II, Arlappa N, Balakrishna N, Mallikharjuna Rao K, Laxmaiah A, Brahmam GN. Trends in the prevalence of undernutrition, nutrient & food intake and predictors of undernutrition among under five year tribal children in India. Asia Pac J Clin Nutr. 2012; 21: 568-76. |
| Meshram II, Balakrishna N, Arlappa N, Rao KM, Laxmaiah A, Brahmam GNV. Prevalence of Undernutrition, Its Determinants, and Seasonal Variation Among Tribal Preschool Children of Odisha State, India. Asia Pac J Public Health. 2014; 26: 470-80. |
| Meshram II, Kodavanti MR, Chitty GR, Manchala R, Kumar S, Kakani SK, Kodavalla V, Avula L, Ginnela Narsimhachary Veera B. Influence of Feeding Practices and Associated Factors on the Nutritional Status of Infants in Rural Areas of Madhya Pradesh State, India. Asia Pac J Public Health. 2015; 27: NP1345-61. |
| Meshram II, Laxmaiah A, Venkaiah K, Brahmam GNV. Impact of feeding and breastfeeding practices on the nutritional status of infants in a district of Andhra Pradesh, India. Natl Med J India. 2012; 25: 2016. |
| Ministry of Women and Child Development, Government of India, United Nations Children's Fund. India Rapid Survey on Children 2013-2014. New Delhi, India: Ministry of Women and Child Development. |
| National Nutrition Monitoring Bureau, National Institute of Nutrition (NIN), Indian Council of Medical Research. India Rural First Repeat Survey of Diet and Nutritional Status Data 1988-1990. [Data shared for this analysis]. |
| National Nutrition Monitoring Bureau, National Institute of Nutrition (NIN), Indian Council of Medical Research. India Rural First Repeat Survey of Diet and Nutritional Status Report 1988-1990. Hyderabad, India: NIN. |
| National Nutrition Monitoring Bureau, National Institute of Nutrition (NIN), Indian Council of Medical Research. India Rural Second Repeat Survey of Diet and Nutritional Status Data 1996-1997. [Data shared for this analysis]. |
| National Nutrition Monitoring Bureau, National Institute of Nutrition (NIN), Indian Council of Medical Research. India Rural Second Repeat Survey of Diet and Nutritional Status Report 1996-1997. Hyderabad, India: NIN. |
| National Nutrition Monitoring Bureau, National Institute of Nutrition (NIN), Indian Council of Medical Research. India Rural Survey of Diet and Nutritional Status Data 1994-1995. [Data shared for this analysis]. |
| National Nutrition Monitoring Bureau, National Institute of Nutrition (NIN), Indian Council of Medical Research. India Rural Survey of Diet and Nutritional Status Data 2000-2001. [Data shared for this analysis] |
| National Nutrition Monitoring Bureau, National Institute of Nutrition (NIN), Indian Council of Medical Research. India Rural Survey of Diet and Nutritional Status Data 2000-2001. [Data shared for this analysis]. |
| National Nutrition Monitoring Bureau, National Institute of Nutrition (NIN), Indian Council of Medical Research. India Rural Survey of Diet and Nutritional Status Data 2004-2006. [Data shared for this analysis]. |
| National Nutrition Monitoring Bureau, National Institute of Nutrition (NIN), Indian Council of Medical Research. India Rural Survey of Diet and Nutritional Status Report 1994-1995. Hyderabad, India: NIN. |
| National Nutrition Monitoring Bureau, National Institute of Nutrition (NIN), Indian Council of Medical Research. India Rural Survey of Diet and Nutritional Status Report 2000-2001. Hyderabad, India: NIN. |
| National Nutrition Monitoring Bureau, National Institute of Nutrition (NIN), Indian Council of Medical Research. India Rural Survey of Diet and Nutritional Status Report 2004-2006. Hyderabad, India: NIN. |
| National Nutrition Monitoring Bureau, National Institute of Nutrition (NIN), Indian Council of Medical Research. India Rural Third Repeat Survey of Diet and Nutritional Status Data 2011-2012. [Data shared for this analysis]. |
| National Nutrition Monitoring Bureau, National Institute of Nutrition (NIN), Indian Council of Medical Research. India Rural Third Repeat Survey of Diet and Nutritional Status Report 2011-2012. Hyderabad, India: NIN. |
| National Nutrition Monitoring Bureau, National Institute of Nutrition (NIN), Indian Council of Medical Research. India Survey of Diet and Nutritional Status Data 1990-1992. [Data shared for this analysis] |
| National Nutrition Monitoring Bureau, National Institute of Nutrition (NIN), Indian Council of Medical Research. India Survey of Diet and Nutritional Status Data 1991-1992. [Data shared for this analysis] |
| National Nutrition Monitoring Bureau, National Institute of Nutrition (NIN), Indian Council of Medical Research. India Survey of Diet and Nutritional Status Report 1990-1992. Hyderabad, India: NIN. |
| National Nutrition Monitoring Bureau, National Institute of Nutrition (NIN), Indian Council of Medical Research. India Survey of Diet and Nutritional Status Report 1991-1992. Hyderabad, India: NIN. |
| National Nutrition Monitoring Bureau, National Institute of Nutrition (NIN), Indian Council of Medical Research. India Tribal First Repeat Survey of Diet and Nutritional Status Data 1998-1999. [Data shared for this analysis]. |
| National Nutrition Monitoring Bureau, National Institute of Nutrition (NIN), Indian Council of Medical Research. India Tribal First Repeat Survey of Diet and Nutritional Status Report 1998-1999. Hyderabad, India: NIN. |
| National Nutrition Monitoring Bureau, National Institute of Nutrition (NIN), Indian Council of Medical Research. India Tribal Second Repeat Survey of Diet and Nutritional Status Data 2007-2008. [Data shared for this analysis]. |
| National Nutrition Monitoring Bureau, National Institute of Nutrition (NIN), Indian Council of Medical Research. India Tribal Second Repeat Survey of Diet and Nutritional Status Report 2007-2008. Hyderabad, India: NIN. |
| National Nutrition Monitoring Bureau, National Institute of Nutrition (NIN), Indian Council of Medical Research. India Urban Nutrition Survey Data 2015-2016. [Data shared for this analysis] |
| National Nutrition Monitoring Bureau, National Institute of Nutrition (NIN), Indian Council of Medical Research. India Urban Slums Survey of Diet and Nutritional Status Data 1993-1994. [Data shared for this analysis]. |
| National Nutrition Monitoring Bureau, National Institute of Nutrition (NIN), Indian Council of Medical Research. India Urban Slums Survey of Diet and Nutritional Status Report 1993-1994. Hyderabad, India: NIN. |
| National Nutrition Monitoring Bureau, National Institute of Nutrition, Indian Council of Medical Research. India National Nutrition Monitoring Bureau Eight States Pooled Data 1991-1992. [Data shared for this analysis] |
| Office of the Registrar General & Census Commissioner, Ministry of Home Affairs, Government of India. India Annual Health Survey Data 2010-2011. New Delhi, India: Office of the Registrar General & Census Commissioner. |
| Office of the Registrar General & Census Commissioner, Ministry of Home Affairs, Government of India. India Annual Health Survey Data 2010-2013. New Delhi, India: Office of the Registrar General & Census Commissioner. |
| Office of the Registrar General & Census Commissioner, Ministry of Home Affairs, Government of India. India Annual Health Survey Data 2011-2012. New Delhi, India: Office of the Registrar General & Census Commissioner. |
| Office of the Registrar General & Census Commissioner, Ministry of Home Affairs, Government of India. India Annual Health Survey Data 2012-2013. New Delhi, India: Office of the Registrar General & Census Commissioner. |
| Office of the Registrar General & Census Commissioner, Ministry of Home Affairs, Government of India. India Annual Health Survey Report 2010-2011. New Delhi, India: Office of the Registrar General & Census Commissioner. |
| Office of the Registrar General & Census Commissioner, Ministry of Home Affairs, Government of India. India Annual Health Survey Report 2010-2013. New Delhi, India: Office of the Registrar General & Census Commissioner. |
| Office of the Registrar General & Census Commissioner, Ministry of Home Affairs, Government of India. India Annual Health Survey Report 2011-2012. New Delhi, India: Office of the Registrar General & Census Commissioner. |
| Office of the Registrar General & Census Commissioner, Ministry of Home Affairs, Government of India. India Annual Health Survey Report 2012-2013. New Delhi, India: Office of the Registrar General & Census Commissioner. |
| Office of the Registrar General and Census Commissioner, Ministry of Health and Family Welfare, Government of India, National Institute of Health and Family Welfare, Nutrition Foundation of India, National Institute of Nutrition, Indian Council of Medical Research. India Clinical, Anthropometric and Bio-chemical (CAB) Survey Data 2014 [Biomarker Component of Annual Health Survey]. New Delhi, India: Office of the Registrar General and Census Commissioner. |
| Office of the Registrar General and Census Commissioner, Ministry of Health and Family Welfare, Government of India, National Institute of Health and Family Welfare, Nutrition Foundation of India, National Institute of Nutrition, Indian Council of Medical Research. India Clinical, Anthropometric and Bio-chemical (CAB) Survey Report 2014 [Biomarker Component of Annual Health Survey]. New Delhi, India: Office of the Registrar General and Census Commissioner. |
| Olofin I, McDonald CM, Ezzati M, Flaxman S, Black RE, Fawzi WW, Caulfield LE, Danaei G; Nutrition Impact Model Study (anthropometry cohort pooling). Associations of suboptimal growth with all-cause and cause-specific mortality in children under five years: a pooled analysis of ten prospective studies. PLoS One. 2013; 8: e64636. |
| Pesaresi M, Ehrlich D, Ferri S, Florczyk A.J, Freire S, Halkiaet M, et al. Operating procedure for the production of the Global Human Settlement Layer from Landsat data of the epochs 1975, 1990, 2000, and 2014; JRC Technical Report EUR 27741 EN; 2016. |
| Prinja S, Thakur JS, Bhatia SS. Pilot testing of WHO child growth standards in Chandigarh: implications for India's child health programmes. Bull World Health Organ. 2009; 87: 116-22. |
| Randomised trial to assess benefits and safety of vitamin A supplementation linked to immunisation in early infancy. WHO/CHD Immunisation-Linked Vitamin A Supplementation Study Group.Lancet. 1998; 352: 1257-63. |
| Reddaiah VP, Kapoor SK. Socio-biological factors in underfive deaths in a rural area. Indian J Pediatr. 1992; 59: 567-71. |
| Sachdev HPS, Osmond C, Fall CHD, Lakshmy R, Ramji S, Dey Biswas SK, Prabhakaran D, Tandon N, Reddy KS, Barker DJP, Bhargava SK. Predicting adult metabolic syndrome from childhood body mass index: follow-up of the New Delhi birth cohort. Arch Dis Child. 2009; 94: 768-74. |
| Saleem S, McClure EM, Goudar SS, Patel A, Esamai F, Garces A, Chomba E, Althabe F, Moore J, Kodkany B, Pasha O, Belizan J, Mayansyan A, Derman RJ, Hibberd PL, Liechty EA, Krebs NF, Hambidge KM, Buekens P, Carlo WA, Wright LL, Koso-Thomas M, Jobe AH, Goldenberg RL, on behalf of the Global Network Maternal Newborn Health Registry Study Investigators. A prospective study of maternal, fetal and neonatal deaths in low- and middle-income countries. Bull World Health Organ. 2014; 92: 605-12. |
| Saxena N, Nayar D, Kapil U. Prevalence of underweight, stunting and wasting. Indian Pediatr. 1997; 34: 627-31. |
| Scott W, Mathew NT. A Development Monitoring Service at the Local Level: Monitoring Change in Kerala: The First Five Years. Geneva, Switzerland: United Nations Research Institute for Social Development; 1985. |
| Singhal PK, Mathur GP, Mathur S, Singh YD. Mortality patterns in under six children in I.C.D.S. urban slum. Indian Pediatr. 1986; 23: 617-22. |
| Spector JM, Agrawal P, Kodkany B, Lipsitz S, Lashoher A, Dziekan G, Bahl R, Merialdi M, Mathai M, Lemer C, Gawande A. Improving Quality of Care for Maternal and Newborn Health: Prospective Pilot Study of the WHO Safe Childbirth Checklist Program. PLoS One. 2012; 7: e35151. |
| Toth G, Kozlowski B, Prieler S, Wiberg D. Global Agro-ecological Zones (GAEZ v3. 0): User’s Guide. 2012. |
| University of East Anglia. Climatic Research Unit TS v. 3.24 dataset. https://crudata.uea.ac.uk/cru/data/hrg/cru_ts_3.24.01/. (accessed July 2017 ) |
| USGS & NASA. MOD13A1.005 Vegetation Indices 16-Day L3 Global 500m dataset. https://lpdaac.usgs.gov/dataset_discovery/modis/modis_products_table/mod13a1 (accessed July 2017). |
| Vijayaraghavan K, Rao DH. Diet & nutrition situation in rural India. Indian J Med Res. 1998; 108: 243-53. |
| Weiss DJ, Atkinson PM, Bhatt S, Mappin B, Hay SI, Gething PW. An effective approach for gap-filling continental scale remotely sensed time-series. ISPRS Journal of Photogrammetry and Remote Sensing. 2014; 98: 106-18 (accessed July 2017). |
| Weiss DJ, Nelson A, Gibson HS, Temperley W, Peedell S, Lieber A, et al. A global map of travel time to cities to assess inequalities in accessibility in 2015. Nature. 2018; 553: 333–6. |
| World Bank. Survey of Living Conditions 1997-1998, Uttar Pradesh and Bihar. Washington D.C., United States: World Bank. |
| World Health Organization (WHO). Global Database on Child Growth and Malnutrition - Historical. Geneva, Switzerland: WHO. |
| World Health Organization (WHO). WHO Global Survey on Maternal and Perinatal Health 2004-2008. Geneva, Switzerland: WHO. |
| World Health Organization. Study on Global Ageing and Adult Health (SAGE) Pilot Study 2005 Data from the Data Archive of Social Research on Aging. Los Altos, United States: Sociometrics Corporation. Available from: http://home.socio.com/age2728.php |

# **3****. Grouping of the states of India based on Socio-demographic Index, 2017**

| **State group**  **(population in 2017)** | **States of India^*^** | **SDI in 2017** |
| --- | --- | --- |
| **Low SDI states (675 million)** | Bihar | 0.43 |
|  | Madhya Pradesh | 0.49 |
|  | Jharkhand | 0.49 |
|  | Uttar Pradesh | 0.49 |
|  | Rajasthan | 0.49 |
|  | Chhattisgarh | 0.51 |
|  | Odisha | 0.52 |
|  | Assam | 0.53 |
| **Middle SDI states (387 million)** | Andhra Pradesh | 0.54 |
|  | West Bengal | 0.54 |
|  | Tripura | 0.54 |
|  | Arunachal Pradesh | 0.56 |
|  | Meghalaya | 0.56 |
|  | Karnataka | 0.57 |
|  | Telangana | 0.58 |
|  | Gujarat | 0.58 |
|  | Manipur | 0.59 |
|  | Jammu and Kashmir^†^ | 0.59 |
|  | Haryana | 0.60 |
| **High SDI states (318 million)** | Uttarakhand | 0.61 |
|  | Tamil Nadu | 0.62 |
|  | Mizoram | 0.62 |
|  | Maharashtra | 0.62 |
|  | Punjab | 0.62 |
|  | Sikkim | 0.63 |
|  | Nagaland | 0.63 |
|  | Himachal Pradesh | 0.63 |
|  | UTs other than Delhi | 0.65 |
|  | Kerala | 0.66 |
|  | Delhi | 0.72 |
|  | Goa | 0.74 |

SDI=Socio-demographic Index, UTs=Union Territories.

SDI as computed by GBD in 2017 as described elsewhere (Lancet 2018; 392: 1995-2051).

*The states are listed in increasing order of SDI in 2017.

^†^The state of Jammu and Kashmir was divided into two union territories in August 2019; as we are reporting findings up to 2017, we report findings for the undivided state of Jammu and Kashmir.

#

# **4. Prevalence of stunting in the districts of India in 2000, 2010 and 2017**

| **States*/Districts** | **Stunting percent prevalence (95% uncertainty interval)** | | | | | | |
| --- | --- | --- | --- | --- | --- | --- | --- |
|  | **2000** | **2010** | **2017** | **Percentage change, 2000-2017** | **Annual rate of change, 2000-2017 (%)** | **Percentage change, 2010-2017** | **Annual rate of change 2010-2017 (%)** |
| **India** | **55.8 (54.5-57.0)** | **47.3 (46.7-47.9)** | **39.3 (38.7-40.1)** | **-30.5 (-30.6 to -30.3)** | **-2.03 (-2.05 to -2.02)** | **-17.8 (-18.0 to -17.7)** | **-2.63 (-2.94 to -2.27)** |
| **Bihar** | **60.3 (56.2-65.6)** | **54.8 (52.7-57.4)** | **48.3 (45.6-52.4)** | **-20.9 (-21.5 to -20.5)** | **-1.30 (-1.34 to -1.27)** | **-12.9 (-13.6 to -12.5)** | **-1.82 (-2.97 to -0.38)** |
| Araria | 63.0 (59.0-66.6) | 50.0 (46.0-54.2) | 46.7 (45.4-47.8) | -26.8 (-27.7 to -26.1) | -1.74 (-1.81 to -1.68) | -7.6 (-8.9 to -6.7) | -0.98 (-1.17 to -0.83) |
| Arwal | 54.8 (47.1-62.4) | 55.8 (50.7-61.1) | 48.2 (46.0-50.5) | -12.6 (-14.8 to -11.2) | -0.74 (-0.87 to -0.63) | -14.5 (-16.1 to -13.2) | -2.07 (-2.31 to -1.84) |
| Aurangabad | 55.6 (48.0-63.2) | 56.6 (51.8-61.3) | 51.2 (50.0-52.7) | -8.5 (-10.4 to -7.3) | -0.47 (-0.58 to -0.38) | -10.4 (-11.7 to -9.4) | -1.42 (-1.60 to -1.25) |
| Banka | 60.0 (51.6-67.9) | 55.7 (50.3-61.4) | 50.1 (48.7-51.5) | -17.2 (-19.4 to -16.1) | -1.05 (-1.19 to -0.96) | -11.0 (-12.3 to -9.6) | -1.51 (-1.70 to -1.27) |
| Begusarai | 57.5 (50.1-65.2) | 54.7 (49.6-60.3) | 46.7 (45.3-47.9) | -19.5 (-21.7 to -18.2) | -1.21 (-1.35 to -1.11) | -15.5 (-16.8 to -14.5) | -2.23 (-2.42 to -2.04) |
| Bhagalpur | 59.5 (52.7-65.7) | 53.9 (49.5-58.5) | 48.6 (47.5-49.8) | -18.9 (-20.7 to -18.2) | -1.17 (-1.28 to -1.10) | -10.6 (-11.8 to -9.6) | -1.45 (-1.62 to -1.27) |
| Bhojpur | 54.9 (48.2-61.6) | 54.4 (49.3-58.8) | 44.5 (43.1-45.8) | -19.7 (-21.3 to -18.2) | -1.22 (-1.32 to -1.10) | -19.0 (-20.2 to -18.1) | -2.82 (-3.00 to -2.64) |
| Buxar | 55.7 (49.5-61.5) | 53.1 (48.5-57.6) | 45.1 (43.9-46.1) | -19.9 (-21.2 to -19.0) | -1.23 (-1.32 to -1.16) | -16.0 (-17.2 to -15.1) | -2.31 (-2.49 to -2.15) |
| Darbhanga | 63.4 (58.4-68.4) | 55.8 (51.3-60.7) | 49.6 (47.9-51.4) | -22.7 (-24.0 to -21.6) | -1.43 (-1.52 to -1.35) | -11.9 (-13.5 to -10.9) | -1.65 (-1.90 to -1.48) |
| East Champaran | 65.7 (62.3-68.8) | 55.7 (51.9-59.2) | 49.3 (48.5-50.3) | -25.9 (-26.7 to -25.5) | -1.68 (-1.73 to -1.64) | -12.3 (-13.4 to -11.7) | -1.70 (-1.87 to -1.60) |
| Gaya | 54.8 (47.1-62.6) | 56.3 (51.9-60.8) | 50.3 (49.4-51.4) | -8.6 (-10.4 to -7.1) | -0.48 (-0.58 to -0.37) | -11.4 (-12.7 to -10.7) | -1.57 (-1.77 to -1.44) |
| Gopalganj | 56.0 (50.6-60.6) | 50.6 (46.0-55.0) | 43.2 (42.1-44.6) | -23.8 (-25.1 to -23.0) | -1.52 (-1.61 to -1.45) | -15.6 (-17.1 to -14.8) | -2.24 (-2.47 to -2.10) |
| Jamui | 58.3 (50.2-65.4) | 56.0 (51.5-60.9) | 49.8 (48.9-51.0) | -15.2 (-17.2 to -14.5) | -0.91 (-1.04 to -0.85) | -11.8 (-12.9 to -10.8) | -1.64 (-1.80 to -1.47) |
| Jehanabad | 56.2 (49.0-63.6) | 56.8 (51.7-61.5) | 49.4 (47.9-50.9) | -12.7 (-14.6 to -11.1) | -0.74 (-0.86 to -0.63) | -13.9 (-15.1 to -13.0) | -1.96 (-2.14 to -1.82) |
| Kaimur | 57.4 (50.2-63.7) | 53.9 (49.5-58.4) | 50.0 (48.9-51.1) | -13.6 (-15.2 to -12.4) | -0.80 (-0.90 to -0.71) | -8.0 (-9.2 to -7.3) | -1.05 (-1.21 to -0.92) |
| Katihar | 60.9 (55.1-65.7) | 52.0 (48.6-55.6) | 48.1 (46.8-49.5) | -21.8 (-23.1 to -21.1) | -1.37 (-1.46 to -1.31) | -8.4 (-9.7 to -7.8) | -1.11 (-1.28 to -0.99) |
| Khagaria | 59.6 (52.3-66.0) | 54.6 (49.7-59.1) | 48.4 (47.1-49.9) | -19.5 (-21.1 to -18.9) | -1.21 (-1.31 to -1.15) | -12.2 (-13.6 to -11.4) | -1.70 (-1.90 to -1.56) |
| Kishanganj | 60.0 (56.2-63.8) | 50.9 (47.2-54.4) | 47.7 (46.2-49.1) | -21.5 (-22.4 to -20.8) | -1.35 (-1.41 to -1.29) | -7.2 (-8.4 to -6.2) | -0.93 (-1.10 to -0.75) |
| Lakhisarai | 58.0 (49.7-65.4) | 55.4 (50.1-60.4) | 47.9 (46.4-49.7) | -18.1 (-20.0 to -16.8) | -1.11 (-1.23 to -1.01) | -14.4 (-15.8 to -13.6) | -2.05 (-2.26 to -1.91) |
| Madhepura | 62.1 (56.1-67.4) | 53.6 (49.2-58.1) | 49.1 (47.8-50.6) | -21.7 (-23.0 to -20.9) | -1.36 (-1.45 to -1.30) | -9.2 (-10.5 to -8.1) | -1.23 (-1.42 to -1.05) |
| Madhubani | 68.9 (65.5-72.2) | 56.1 (52.1-59.6) | 50.4 (49.0-51.6) | -27.8 (-28.4 to -27.3) | -1.82 (-1.86 to -1.78) | -11.1 (-12.2 to -10.4) | -1.52 (-1.68 to -1.39) |
| Munger | 58.4 (50.8-65.9) | 54.9 (49.5-59.9) | 47.9 (45.6-49.8) | -18.6 (-20.4 to -17.8) | -1.15 (-1.26 to -1.08) | -13.5 (-14.9 to -12.7) | -1.92 (-2.12 to -1.76) |
| Muzaffarpur | 64.0 (59.8-67.8) | 57.2 (53.8-60.9) | 49.7 (48.8-50.5) | -23.2 (-24.2 to -22.7) | -1.47 (-1.54 to -1.43) | -14.1 (-15.1 to -13.3) | -2.00 (-2.14 to -1.86) |
| Nalanda | 57.4 (50.6-64.2) | 56.9 (52.2-61.4) | 48.8 (47.7-49.8) | -15.7 (-17.5 to -14.7) | -0.94 (-1.06 to -0.86) | -15.2 (-16.5 to -14.5) | -2.18 (-2.38 to -2.04) |
| Nawada | 56.5 (48.9-64.2) | 56.0 (50.7-60.6) | 49.6 (48.4-50.5) | -12.8 (-14.7 to -11.6) | -0.75 (-0.86 to -0.65) | -12.4 (-13.4 to -11.5) | -1.72 (-1.87 to -1.57) |
| Patna | 55.6 (48.9-61.7) | 55.3 (50.6-60.0) | 45.5 (44.2-46.9) | -18.9 (-20.6 to -17.8) | -1.16 (-1.27 to -1.08) | -18.5 (-19.7 to -17.8) | -2.73 (-2.91 to -2.60) |
| Purnia | 62.3 (57.5-66.5) | 52.2 (48.4-55.6) | 48.3 (47.3-49.1) | -23.4 (-24.4 to -22.8) | -1.49 (-1.56 to -1.44) | -8.4 (-9.5 to -7.5) | -1.10 (-1.25 to -0.96) |
| Rohtas | 56.1 (49.6-61.9) | 54.8 (50.7-59.0) | 49.3 (48.5-50.2) | -12.8 (-14.7 to -11.7) | -0.74 (-0.86 to -0.67) | -10.8 (-12.1 to -10.0) | -1.48 (-1.67 to -1.34) |
| Saharsa | 61.2 (55.2-66.3) | 54.4 (49.9-58.8) | 49.0 (47.7-50.2) | -20.8 (-22.2 to -20.0) | -1.30 (-1.39 to -1.23) | -10.9 (-12.1 to -10.1) | -1.49 (-1.66 to -1.35) |
| Samastipur | 60.4 (55.1-65.5) | 56.4 (51.7-60.8) | 48.9 (47.9-50.0) | -19.9 (-21.2 to -18.9) | -1.23 (-1.32 to -1.15) | -14.2 (-15.4 to -13.5) | -2.01 (-2.20 to -1.88) |
| Saran | 58.6 (53.4-63.9) | 55.3 (51.2-59.1) | 45.3 (44.2-46.3) | -23.5 (-24.6 to -22.6) | -1.50 (-1.57 to -1.42) | -18.9 (-20.0 to -18.2) | -2.80 (-2.96 to -2.66) |
| Sheikhpura | 57.2 (49.7-64.2) | 55.7 (50.6-60.4) | 48.1 (46.0-50.2) | -16.6 (-18.5 to -15.6) | -1.01 (-1.12 to -0.92) | -14.5 (-16.1 to -13.6) | -2.08 (-2.31 to -1.91) |
| Sheohar | 68.4 (63.4-72.8) | 58.4 (53.4-63.0) | 51.8 (49.1-54.8) | -25.2 (-26.5 to -24.4) | -1.63 (-1.72 to -1.56) | -12.1 (-13.7 to -11.0) | -1.69 (-1.92 to -1.50) |
| Sitamarhi | 70.6 (66.8-74.0) | 58.3 (53.9-62.3) | 52.1 (50.7-53.5) | -27.1 (-28.0 to -26.6) | -1.77 (-1.83 to -1.72) | -11.5 (-12.6 to -10.7) | -1.58 (-1.74 to -1.45) |
| Siwan | 55.2 (50.2-60.0) | 51.8 (47.4-56.4) | 42.8 (41.7-43.8) | -23.4 (-24.9 to -22.7) | -1.49 (-1.59 to -1.43) | -18.3 (-19.3 to -17.2) | -2.68 (-2.85 to -2.49) |
| Supaul | 64.2 (60.4-68.0) | 52.1 (48.1-56.0) | 48.1 (46.9-49.4) | -26.0 (-26.9 to -25.5) | -1.68 (-1.75 to -1.64) | -8.6 (-9.9 to -7.8) | -1.13 (-1.32 to -1.00) |
| Vaishali | 60.1 (53.8-65.7) | 57.3 (53.3-61.6) | 48.4 (47.2-49.7) | -20.2 (-21.7 to -19.6) | -1.26 (-1.35 to -1.20) | -16.4 (-17.3 to -15.5) | -2.37 (-2.51 to -2.22) |
| West Champaran | 65.1 (61.1-68.6) | 52.6 (48.4-56.6) | 48.8 (47.5-50.5) | -26.0 (-26.7 to -25.4) | -1.68 (-1.74 to -1.63) | -8.0 (-9.0 to -6.9) | -1.04 (-1.18 to -0.86) |
| **Madhya Pradesh** | **59.5 (57.6-61.8)** | **50.2 (48.7-51.9)** | **40.9 (39.2-43.0)** | **-32.2 (-32.5 to -32.0)** | **-2.18 (-2.20 to -2.16)** | **-19.5 (-20.0 to -19.2)** | **-2.93 (-3.70 to -2.10)** |
| Agar Malwa | 59.7 (54.0-65.4) | 53.1 (48.4-57.8) | 38.0 (36.6-39.4) | -37.3 (-38.3 to -36.3) | -2.62 (-2.71 to -2.53) | -29.3 (-30.6 to -28.6) | -4.66 (-4.89 to -4.51) |
| Alirajpur | 57.2 (50.8-63.3) | 50.6 (45.8-55.1) | 45.5 (43.7-47.3) | -21.1 (-22.7 to -20.0) | -1.33 (-1.43 to -1.23) | -10.8 (-12.2 to -9.8) | -1.49 (-1.69 to -1.31) |
| Anuppur | 63.5 (56.7-69.6) | 50.6 (45.2-55.6) | 40.6 (39.3-42.0) | -36.9 (-38.2 to -36.3) | -2.59 (-2.70 to -2.53) | -20.6 (-22.1 to -19.4) | -3.09 (-3.33 to -2.87) |
| Ashoknagar | 60.4 (53.8-65.9) | 49.4 (44.7-54.1) | 41.4 (40.2-42.9) | -32.3 (-33.3 to -31.4) | -2.19 (-2.27 to -2.11) | -17.0 (-18.3 to -15.9) | -2.49 (-2.67 to -2.29) |
| Balaghat | 59.3 (53.1-65.5) | 41.4 (37.0-45.5) | 35.8 (34.6-37.0) | -40.5 (-41.5 to -39.9) | -2.92 (-3.01 to -2.86) | -14.4 (-15.9 to -13.2) | -2.05 (-2.27 to -1.83) |
| Barwani | 54.8 (48.7-60.3) | 51.8 (47.0-56.2) | 45.3 (44.0-46.6) | -18.2 (-20.0 to -17.1) | -1.11 (-1.23 to -1.03) | -13.3 (-14.6 to -12.6) | -1.88 (-2.06 to -1.74) |
| Betul | 55.2 (50.0-60.6) | 47.5 (42.8-52.0) | 33.1 (31.8-34.5) | -40.8 (-41.9 to -40.1) | -2.95 (-3.05 to -2.88) | -31.0 (-32.5 to -30.3) | -5.00 (-5.27 to -4.83) |
| Bhind | 60.5 (55.6-65.7) | 52.4 (47.7-56.8) | 44.3 (43.4-45.3) | -27.6 (-28.7 to -27.1) | -1.81 (-1.89 to -1.77) | -16.2 (-17.5 to -15.4) | -2.34 (-2.55 to -2.20) |
| Bhopal | 56.5 (50.4-62.6) | 51.5 (45.7-57.7) | 39.1 (36.4-41.5) | -31.7 (-33.5 to -30.8) | -2.15 (-2.28 to -2.06) | -24.9 (-26.4 to -23.6) | -3.85 (-4.10 to -3.59) |
| Burhanpur | 52.2 (46.8-58.3) | 56.9 (52.1-61.6) | 43.5 (41.7-45.3) | -17.4 (-18.5 to -16.0) | -1.06 (-1.12 to -0.95) | -24.4 (-25.9 to -23.7) | -3.75 (-4.00 to -3.62) |
| Chhatarpur | 62.2 (56.7-67.9) | 53.2 (49.4-56.9) | 43.5 (42.4-44.7) | -31.0 (-32.1 to -30.2) | -2.08 (-2.17 to -2.01) | -19.2 (-20.3 to -18.5) | -2.84 (-3.01 to -2.72) |
| Chhindwara | 57.0 (51.4-62.8) | 44.5 (39.5-49.1) | 34.3 (33.1-35.4) | -40.7 (-41.6 to -39.7) | -2.94 (-3.02 to -2.84) | -23.7 (-25.2 to -22.4) | -3.63 (-3.88 to -3.39) |
| Damoh | 62.1 (56.4-67.9) | 50.6 (46.5-55.0) | 43.0 (41.5-44.6) | -31.7 (-32.9 to -30.9) | -2.14 (-2.24 to -2.07) | -16.0 (-17.2 to -15.1) | -2.30 (-2.49 to -2.15) |
| Datia | 59.6 (54.0-65.4) | 48.9 (44.0-53.7) | 42.4 (41.1-43.8) | -29.7 (-30.7 to -28.8) | -1.98 (-2.05 to -1.90) | -13.9 (-15.3 to -12.9) | -1.98 (-2.18 to -1.79) |
| Dewas | 55.9 (50.9-60.9) | 51.6 (47.1-56.3) | 38.9 (37.8-40.0) | -31.3 (-32.5 to -30.3) | -2.11 (-2.20 to -2.02) | -25.6 (-26.6 to -24.7) | -3.96 (-4.14 to -3.79) |
| Dhar | 56.3 (50.8-61.3) | 48.3 (44.5-52.4) | 43.4 (42.1-44.5) | -23.7 (-25.1 to -22.9) | -1.51 (-1.61 to -1.45) | -11.0 (-12.3 to -9.9) | -1.51 (-1.69 to -1.33) |
| Dindori | 64.5 (59.0-69.8) | 48.8 (44.6-53.5) | 43.3 (42.3-44.5) | -33.7 (-34.9 to -33.0) | -2.31 (-2.41 to -2.24) | -12.0 (-13.4 to -10.5) | -1.67 (-1.87 to -1.41) |
| Guna | 63.0 (57.1-68.9) | 52.5 (47.9-56.9) | 42.6 (41.5-43.9) | -33.2 (-34.4 to -32.4) | -2.27 (-2.36 to -2.19) | -19.8 (-21.0 to -18.9) | -2.94 (-3.13 to -2.77) |
| Gwalior | 61.6 (56.3-67.3) | 51.0 (46.3-55.8) | 43.6 (42.4-45.0) | -30.1 (-31.1 to -29.3) | -2.01 (-2.09 to -1.94) | -15.3 (-16.9 to -14.3) | -2.20 (-2.44 to -2.01) |
| Harda | 56.0 (49.3-62.3) | 53.8 (48.4-58.8) | 39.4 (37.7-41.2) | -30.3 (-32.1 to -29.5) | -2.04 (-2.17 to -1.95) | -27.6 (-29.0 to -26.9) | -4.34 (-4.58 to -4.19) |
| Hoshangabad | 56.9 (52.1-62.2) | 50.2 (45.1-55.2) | 37.3 (36.3-38.5) | -35.4 (-36.7 to -34.9) | -2.46 (-2.56 to -2.40) | -26.6 (-28.1 to -25.9) | -4.16 (-4.41 to -4.01) |
| Indore | 53.8 (46.7-60.4) | 48.1 (42.0-54.0) | 37.2 (35.0-39.8) | -31.5 (-33.4 to -30.4) | -2.13 (-2.28 to -2.03) | -23.3 (-25.2 to -22.1) | -3.58 (-3.88 to -3.33) |
| Jabalpur | 61.0 (55.6-66.4) | 43.0 (38.6-47.4) | 36.8 (35.3-38.4) | -40.5 (-41.3 to -39.9) | -2.92 (-2.99 to -2.86) | -15.2 (-16.5 to -14.2) | -2.18 (-2.38 to -2.00) |
| Jhabua | 58.7 (53.2-63.9) | 50.0 (45.5-54.1) | 44.6 (43.3-45.9) | -24.9 (-26.0 to -23.9) | -1.60 (-1.68 to -1.52) | -11.6 (-13.0 to -10.9) | -1.61 (-1.80 to -1.47) |
| Katni | 62.2 (55.7-69.0) | 46.8 (41.9-51.8) | 40.7 (39.3-42.1) | -35.4 (-36.7 to -34.4) | -2.46 (-2.57 to -2.37) | -13.8 (-15.6 to -12.9) | -1.96 (-2.22 to -1.79) |
| Khandwa | 55.5 (50.2-60.9) | 56.5 (51.8-60.8) | 43.7 (42.5-44.6) | -22.2 (-23.7 to -21.2) | -1.40 (-1.51 to -1.32) | -23.7 (-24.9 to -23.0) | -3.62 (-3.82 to -3.49) |
| Khargone | 56.5 (51.2-62.2) | 56.9 (52.2-61.1) | 47.5 (46.3-48.5) | -16.8 (-18.1 to -16.1) | -1.02 (-1.10 to -0.96) | -17.4 (-18.7 to -16.7) | -2.55 (-2.75 to -2.40) |
| Mandla | 63.3 (57.2-69.3) | 42.7 (37.8-47.6) | 37.8 (36.4-39.1) | -41.2 (-42.2 to -40.5) | -2.99 (-3.08 to -2.91) | -12.2 (-14.4 to -10.9) | -1.72 (-2.03 to -1.47) |
| Mandsaur | 60.9 (55.3-66.5) | 52.5 (48.7-56.4) | 37.2 (36.1-38.4) | -39.7 (-40.8 to -39.0) | -2.85 (-2.94 to -2.77) | -29.9 (-31.0 to -29.1) | -4.78 (-4.97 to -4.59) |
| Morena | 64.6 (59.7-69.2) | 55.4 (50.6-59.7) | 46.7 (45.7-47.8) | -28.7 (-29.5 to -28.1) | -1.89 (-1.96 to -1.84) | -16.6 (-17.5 to -15.6) | -2.40 (-2.55 to -2.24) |
| Narsinghpur | 58.5 (53.0-63.7) | 44.8 (40.0-49.5) | 36.6 (35.1-37.7) | -38.3 (-39.2 to -37.7) | -2.72 (-2.79 to -2.65) | -19.0 (-20.7 to -18.0) | -2.83 (-3.08 to -2.63) |
| Neemuch | 61.0 (54.7-67.3) | 51.7 (47.4-55.9) | 35.1 (33.8-36.7) | -43.4 (-44.4 to -42.8) | -3.20 (-3.29 to -3.13) | -33.1 (-34.1 to -32.3) | -5.39 (-5.57 to -5.21) |
| Niwari | 60.2 (54.6-66.2) | 49.6 (45.0-54.0) | 42.7 (41.1-44.3) | -29.8 (-31.3 to -28.8) | -1.99 (-2.10 to -1.90) | -14.7 (-16.2 to -13.7) | -2.11 (-2.33 to -1.92) |
| Panna | 62.3 (56.5-67.8) | 50.4 (46.7-54.0) | 41.2 (40.3-42.2) | -34.7 (-35.8 to -33.9) | -2.40 (-2.48 to -2.32) | -19.0 (-20.0 to -18.4) | -2.81 (-2.96 to -2.70) |
| Raisen | 59.3 (54.7-63.7) | 53.4 (48.3-58.2) | 42.9 (42.0-43.9) | -28.5 (-29.6 to -27.9) | -1.88 (-1.97 to -1.83) | -20.3 (-21.9 to -19.4) | -3.04 (-3.29 to -2.87) |
| Rajgarh | 58.3 (52.5-64.2) | 51.5 (46.1-56.8) | 37.6 (36.5-38.8) | -36.2 (-37.5 to -35.2) | -2.53 (-2.64 to -2.43) | -27.7 (-29.1 to -26.7) | -4.35 (-4.60 to -4.16) |
| Ratlam | 58.7 (53.4-64.5) | 50.9 (46.5-55.1) | 41.2 (40.0-42.2) | -30.8 (-31.6 to -29.7) | -2.06 (-2.13 to -1.97) | -20.0 (-21.3 to -19.2) | -2.98 (-3.19 to -2.84) |
| Rewa | 61.8 (56.9-66.4) | 49.6 (45.9-53.3) | 42.3 (41.4-43.3) | -32.4 (-33.6 to -31.8) | -2.20 (-2.29 to -2.14) | -15.4 (-16.5 to -14.6) | -2.22 (-2.37 to -2.06) |
| Sagar | 60.2 (55.3-65.4) | 51.9 (46.7-56.8) | 44.2 (42.9-45.4) | -27.4 (-28.6 to -26.8) | -1.80 (-1.88 to -1.74) | -15.5 (-16.7 to -14.7) | -2.23 (-2.42 to -2.08) |
| Satna | 61.3 (55.6-66.5) | 46.7 (42.7-50.4) | 39.3 (38.5-40.2) | -36.8 (-37.8 to -36.3) | -2.58 (-2.66 to -2.53) | -16.7 (-17.7 to -15.9) | -2.42 (-2.58 to -2.29) |
| Sehore | 57.4 (52.3-62.4) | 52.9 (47.9-57.7) | 39.3 (38.5-40.4) | -32.4 (-33.2 to -31.8) | -2.20 (-2.26 to -2.14) | -26.6 (-27.8 to -25.7) | -4.15 (-4.35 to -3.98) |
| Seoni | 61.8 (56.0-67.6) | 44.4 (39.1-48.9) | 37.7 (36.5-38.9) | -39.8 (-40.8 to -38.9) | -2.86 (-2.94 to -2.76) | -15.7 (-17.3 to -14.7) | -2.27 (-2.52 to -2.09) |
| Shahdol | 64.7 (58.0-70.3) | 53.6 (48.6-58.3) | 46.6 (45.4-47.9) | -28.8 (-30.1 to -28.1) | -1.91 (-2.00 to -1.84) | -13.9 (-15.3 to -12.9) | -1.97 (-2.18 to -1.80) |
| Shajapur | 56.6 (50.8-62.3) | 51.4 (46.4-56.5) | 37.7 (36.6-38.7) | -34.2 (-35.6 to -33.5) | -2.36 (-2.47 to -2.29) | -27.5 (-28.7 to -26.8) | -4.32 (-4.52 to -4.17) |
| Sheopur | 69.1 (63.8-74.1) | 57.8 (52.9-62.5) | 48.1 (46.9-49.3) | -31.4 (-32.2 to -30.6) | -2.11 (-2.18 to -2.04) | -17.7 (-18.9 to -16.9) | -2.59 (-2.77 to -2.44) |
| Shivpuri | 63.5 (58.2-68.6) | 51.1 (46.8-55.9) | 43.9 (42.7-45.0) | -31.8 (-32.6 to -31.1) | -2.15 (-2.21 to -2.09) | -15.0 (-16.2 to -14.2) | -2.14 (-2.33 to -2.00) |
| Sidhi | 62.3 (56.8-67.1) | 50.5 (46.0-54.9) | 41.9 (40.6-43.0) | -33.6 (-34.6 to -32.9) | -2.30 (-2.38 to -2.23) | -17.8 (-18.9 to -17.0) | -2.60 (-2.77 to -2.46) |
| Singrauli | 59.8 (53.5-65.4) | 48.2 (42.9-53.5) | 36.2 (34.9-37.5) | -40.2 (-41.2 to -39.5) | -2.90 (-2.98 to -2.82) | -25.7 (-26.8 to -24.7) | -3.99 (-4.18 to -3.79) |
| Tikamgarh | 60.6 (54.3-66.7) | 51.4 (46.6-56.1) | 44.6 (43.0-46.0) | -27.1 (-28.6 to -26.1) | -1.78 (-1.88 to -1.69) | -14.0 (-15.6 to -12.9) | -1.98 (-2.22 to -1.79) |
| Ujjain | 56.7 (51.6-62.1) | 50.4 (46.0-55.0) | 38.8 (37.7-39.9) | -32.5 (-33.7 to -31.6) | -2.21 (-2.30 to -2.13) | -23.9 (-24.9 to -23.0) | -3.65 (-3.82 to -3.49) |
| Umaria | 64.4 (58.2-70.5) | 51.2 (46.7-56.0) | 45.3 (44.2-46.4) | -30.5 (-31.9 to -29.6) | -2.04 (-2.15 to -1.96) | -12.4 (-14.0 to -11.3) | -1.73 (-1.97 to -1.55) |
| Vidisha | 60.9 (56.0-66.2) | 55.7 (50.4-61.4) | 47.0 (45.8-48.5) | -23.6 (-24.7 to -22.9) | -1.51 (-1.58 to -1.44) | -16.3 (-17.8 to -15.6) | -2.37 (-2.59 to -2.23) |
| **Jharkhand** | **56.2 (52.5-60.9)** | **51.2 (49.5-53.4)** | **45.7 (43.4-48.7)** | **-19.5 (-20.7 to -18.5)** | **-1.20 (-1.28 to -1.13)** | **-11.7 (-12.5 to -11.2)** | **-1.63 (-2.59 to -0.56)** |
| Bokaro | 52.9 (43.8-61.4) | 47.3 (42.0-52.1) | 41.0 (39.2-43.0) | -22.9 (-25.8 to -21.7) | -1.47 (-1.66 to -1.35) | -14.2 (-16.0 to -13.0) | -2.02 (-2.30 to -1.80) |
| Chatra | 55.7 (45.8-65.3) | 55.2 (49.6-60.9) | 49.6 (48.2-51.2) | -11.2 (-14.1 to -9.4) | -0.66 (-0.82 to -0.52) | -11.0 (-12.4 to -9.6) | -1.52 (-1.72 to -1.28) |
| Deoghar | 57.1 (47.6-66.0) | 49.3 (44.0-54.8) | 45.7 (44.1-47.5) | -20.4 (-22.8 to -19.1) | -1.28 (-1.43 to -1.16) | -8.1 (-9.7 to -6.9) | -1.06 (-1.30 to -0.86) |
| Dhanbad | 52.7 (44.2-60.4) | 45.5 (40.9-50.2) | 41.8 (40.5-43.3) | -21.1 (-23.5 to -19.7) | -1.34 (-1.49 to -1.21) | -8.9 (-10.5 to -7.8) | -1.19 (-1.42 to -1.00) |
| Dumka | 60.0 (53.3-66.4) | 48.5 (44.6-52.9) | 47.0 (45.8-48.2) | -22.5 (-24.1 to -21.5) | -1.43 (-1.53 to -1.34) | -3.9 (-5.1 to -2.7) | -0.44 (-0.60 to -0.25) |
| East Singhbhum | 52.9 (45.7-60.0) | 50.8 (45.2-56.5) | 45.9 (43.1-48.6) | -13.8 (-15.8 to -12.5) | -0.82 (-0.94 to -0.71) | -10.4 (-12.3 to -9.2) | -1.42 (-1.70 to -1.22) |
| Garhwa | 58.1 (49.0-66.8) | 52.8 (47.5-58.6) | 45.6 (44.0-47.5) | -21.9 (-23.8 to -20.3) | -1.39 (-1.51 to -1.26) | -14.3 (-15.7 to -13.0) | -2.04 (-2.25 to -1.81) |
| Giridih | 55.6 (45.8-64.8) | 50.6 (45.5-55.7) | 46.2 (45.2-47.3) | -17.3 (-20.2 to -16.0) | -1.06 (-1.25 to -0.95) | -9.5 (-10.9 to -8.4) | -1.28 (-1.48 to -1.09) |
| Godda | 59.9 (52.1-67.0) | 50.0 (44.9-55.0) | 45.8 (44.3-47.3) | -24.3 (-26.1 to -23.1) | -1.56 (-1.69 to -1.46) | -9.2 (-10.9 to -8.2) | -1.23 (-1.48 to -1.07) |
| Gumla | 58.1 (49.9-67.0) | 54.3 (48.9-60.0) | 42.9 (41.6-44.1) | -26.8 (-28.2 to -25.5) | -1.75 (-1.85 to -1.64) | -22.0 (-23.2 to -21.0) | -3.32 (-3.53 to -3.13) |
| Hazaribagh | 54.9 (45.1-64.3) | 53.0 (47.4-58.3) | 47.2 (46.0-48.5) | -14.3 (-17.4 to -13.0) | -0.86 (-1.05 to -0.75) | -11.7 (-13.3 to -10.9) | -1.63 (-1.85 to -1.48) |
| Jamtara | 57.5 (48.7-65.4) | 47.7 (42.7-52.5) | 46.0 (44.5-47.6) | -20.5 (-22.6 to -19.5) | -1.28 (-1.42 to -1.19) | -4.3 (-6.4 to -3.2) | -0.50 (-0.80 to -0.32) |
| Khunti | 56.9 (47.5-66.5) | 54.9 (48.9-60.3) | 44.5 (42.6-46.4) | -22.2 (-24.4 to -20.6) | -1.41 (-1.55 to -1.28) | -19.7 (-21.4 to -18.6) | -2.93 (-3.21 to -2.73) |
| Koderma | 54.2 (43.3-64.8) | 51.1 (44.3-57.1) | 46.3 (44.4-48.7) | -14.5 (-17.8 to -13.0) | -0.88 (-1.08 to -0.75) | -10.0 (-12.0 to -8.8) | -1.37 (-1.65 to -1.15) |
| Latehar | 57.3 (47.5-66.8) | 54.4 (48.8-60.7) | 46.1 (44.7-47.9) | -20.0 (-22.0 to -18.6) | -1.25 (-1.37 to -1.13) | -16.0 (-17.2 to -15.0) | -2.31 (-2.50 to -2.13) |
| Lohardaga | 56.1 (46.8-65.9) | 53.9 (48.1-59.7) | 44.1 (42.2-46.0) | -21.8 (-23.9 to -20.1) | -1.39 (-1.52 to -1.24) | -19.0 (-20.7 to -18.0) | -2.82 (-3.08 to -2.63) |
| Pakur | 62.0 (55.3-68.1) | 47.8 (43.6-52.2) | 47.9 (46.0-49.9) | -23.5 (-25.0 to -22.5) | -1.50 (-1.60 to -1.41) | -0.5 (-2.2 to 0.6) | 0.06 (-0.17 to 0.22) |
| Palamu | 56.4 (46.9-66.1) | 54.4 (49.0-60.1) | 48.4 (46.8-50.1) | -14.6 (-17.1 to -12.9) | -0.87 (-1.03 to -0.74) | -11.7 (-13.1 to -10.8) | -1.63 (-1.83 to -1.47) |
| Ramgarh | 53.7 (44.1-63.3) | 50.6 (44.8-56.1) | 42.2 (40.0-44.4) | -21.7 (-24.5 to -20.9) | -1.38 (-1.57 to -1.29) | -17.2 (-18.7 to -16.2) | -2.52 (-2.75 to -2.33) |
| Ranchi | 55.1 (46.1-63.8) | 52.8 (47.4-58.0) | 43.1 (41.6-44.3) | -22.3 (-24.9 to -21.1) | -1.42 (-1.59 to -1.31) | -19.1 (-20.5 to -18.3) | -2.84 (-3.05 to -2.68) |
| Sahibganj | 61.3 (54.5-67.4) | 48.4 (44.4-52.4) | 46.2 (44.8-47.9) | -25.4 (-27.0 to -25.0) | -1.64 (-1.75 to -1.60) | -5.3 (-6.9 to -4.4) | -0.65 (-0.87 to -0.49) |
| Saraikela Kharsawan | 55.2 (48.1-62.4) | 53.6 (48.7-58.2) | 47.6 (45.6-49.8) | -14.4 (-16.3 to -13.2) | -0.86 (-0.97 to -0.77) | -12.0 (-13.6 to -10.9) | -1.68 (-1.91 to -1.47) |
| Simdega | 60.0 (53.7-67.4) | 54.4 (49.9-58.9) | 41.7 (40.5-43.0) | -31.3 (-32.5 to -30.1) | -2.11 (-2.20 to -2.00) | -24.3 (-25.4 to -23.4) | -3.73 (-3.92 to -3.55) |
| West Singhbhum | 58.7 (52.4-64.9) | 59.8 (55.6-64.1) | 54.6 (52.9-56.3) | -7.8 (-9.5 to -6.7) | -0.42 (-0.52 to -0.34) | -9.6 (-10.6 to -8.8) | -1.29 (-1.42 to -1.16) |
| **Uttar Pradesh** | **64.3 (59.2-69.0)** | **56.5 (54.7-59.0)** | **49.0 (46.6-52.2)** | **-24.7 (-25.0 to -24.5)** | **-1.58 (-1.60 to -1.56)** | **-14.2 (-14.5 to -13.9)** | **-2.02 (-3.02 to -0.88)** |
| Agra | 66.4 (61.6-71.2) | 59.6 (54.5-64.7) | 48.1 (46.7-49.8) | -28.5 (-29.6 to -27.6) | -1.88 (-1.96 to -1.80) | -20.2 (-21.4 to -19.4) | -3.01 (-3.20 to -2.86) |
| Aligarh | 66.7 (61.2-71.6) | 61.3 (55.9-66.0) | 48.4 (46.6-50.7) | -28.3 (-29.4 to -27.7) | -1.87 (-1.95 to -1.81) | -21.9 (-23.1 to -21.1) | -3.31 (-3.50 to -3.15) |
| Allahabad | 64.4 (59.5-69.2) | 55.4 (51.7-58.8) | 50.5 (49.6-51.6) | -22.5 (-23.5 to -21.8) | -1.42 (-1.49 to -1.36) | -9.8 (-10.7 to -9.1) | -1.31 (-1.45 to -1.20) |
| Ambedkar Nagar | 61.1 (56.2-65.7) | 49.5 (45.7-53.4) | 47.6 (46.3-48.6) | -23.0 (-24.2 to -22.2) | -1.46 (-1.54 to -1.39) | -4.8 (-6.3 to -3.9) | -0.57 (-0.77 to -0.42) |
| Amethi | 62.6 (57.6-67.9) | 51.7 (47.9-55.1) | 49.5 (48.0-50.8) | -21.8 (-23.0 to -20.9) | -1.37 (-1.45 to -1.30) | -5.2 (-6.3 to -4.6) | -0.62 (-0.77 to -0.52) |
| Amroha | 64.6 (59.1-69.1) | 57.4 (53.1-61.4) | 44.4 (43.3-45.6) | -32.0 (-33.1 to -31.4) | -2.17 (-2.25 to -2.11) | -23.5 (-24.4 to -22.8) | -3.59 (-3.74 to -3.46) |
| Auraiya | 66.0 (60.4-71.6) | 62.0 (56.9-66.5) | 52.4 (51.1-53.6) | -21.4 (-22.3 to -20.3) | -1.34 (-1.40 to -1.26) | -16.3 (-17.7 to -15.5) | -2.36 (-2.57 to -2.21) |
| Azamgarh | 57.4 (52.1-62.9) | 48.4 (44.2-52.4) | 44.8 (43.8-45.6) | -22.7 (-24.1 to -21.5) | -1.44 (-1.53 to -1.34) | -8.2 (-9.2 to -7.0) | -1.08 (-1.21 to -0.88) |
| Baghpat | 68.7 (64.2-73.0) | 56.6 (52.4-60.9) | 43.5 (41.8-44.8) | -37.6 (-38.4 to -37.1) | -2.65 (-2.72 to -2.60) | -24.1 (-25.2 to -23.3) | -3.70 (-3.89 to -3.54) |
| Bahraich | 67.1 (62.7-71.2) | 58.7 (55.2-62.6) | 60.3 (59.1-61.5) | -11.1 (-11.9 to -10.3) | -0.63 (-0.68 to -0.58) | 1.8 (0.9 to 2.5) | 0.39 (0.27 to 0.49) |
| Ballia | 55.7 (50.6-60.8) | 54.4 (50.1-58.9) | 42.3 (41.5-43.1) | -24.9 (-26.3 to -24.0) | -1.60 (-1.70 to -1.53) | -23.1 (-24.1 to -22.3) | -3.52 (-3.68 to -3.36) |
| Balrampur | 73.4 (68.4-78.0) | 63.4 (58.9-67.2) | 62.8 (61.5-64.0) | -15.3 (-16.3 to -14.8) | -0.91 (-0.97 to -0.87) | -1.8 (-3.2 to -0.8) | -0.13 (-0.32 to 0.03) |
| Banda | 66.6 (61.2-71.5) | 57.7 (54.4-61.2) | 46.9 (45.9-47.7) | -30.6 (-31.7 to -30.0) | -2.05 (-2.14 to -1.99) | -19.8 (-20.6 to -19.0) | -2.94 (-3.07 to -2.80) |
| Barabanki | 64.5 (59.6-68.9) | 57.3 (53.8-60.7) | 56.0 (55.3-57.1) | -14.0 (-15.1 to -13.2) | -0.82 (-0.89 to -0.76) | -3.1 (-4.1 to -2.5) | -0.31 (-0.45 to -0.21) |
| Bareilly | 66.0 (61.0-70.6) | 58.7 (55.1-62.3) | 51.4 (50.7-52.5) | -23.0 (-24.0 to -22.3) | -1.46 (-1.52 to -1.40) | -13.4 (-14.3 to -12.5) | -1.88 (-2.02 to -1.73) |
| Basti | 64.7 (59.8-69.4) | 53.9 (50.4-57.7) | 51.8 (50.6-52.9) | -20.9 (-22.0 to -20.2) | -1.30 (-1.38 to -1.25) | -4.9 (-6.1 to -3.9) | -0.57 (-0.75 to -0.42) |
| Bhadohi | 62.2 (56.0-67.1) | 54.1 (49.3-58.6) | 49.1 (46.8-51.2) | -21.9 (-23.3 to -21.0) | -1.38 (-1.47 to -1.30) | -10.1 (-11.6 to -9.1) | -1.37 (-1.59 to -1.20) |
| Bijnor | 64.9 (59.6-70.2) | 54.3 (49.4-58.8) | 43.4 (42.1-44.8) | -34.0 (-35.0 to -33.5) | -2.33 (-2.41 to -2.28) | -20.9 (-22.1 to -20.2) | -3.13 (-3.33 to -3.00) |
| Budaun | 67.9 (62.7-72.7) | 63.2 (59.3-67.4) | 54.5 (53.5-55.4) | -20.6 (-21.7 to -19.7) | -1.28 (-1.35 to -1.21) | -14.7 (-15.3 to -13.9) | -2.08 (-2.18 to -1.96) |
| Bulandshahr | 67.2 (62.5-71.3) | 60.5 (56.1-64.3) | 46.2 (45.2-47.3) | -32.1 (-33.0 to -31.5) | -2.17 (-2.24 to -2.12) | -24.5 (-25.5 to -23.9) | -3.76 (-3.93 to -3.65) |
| Chandauli | 59.6 (51.8-66.2) | 55.0 (49.7-60.1) | 47.5 (46.1-49.1) | -20.9 (-22.7 to -20.1) | -1.31 (-1.43 to -1.24) | -14.5 (-15.7 to -13.2) | -2.07 (-2.24 to -1.84) |
| Chitrakoot | 65.7 (59.6-72.0) | 54.1 (49.6-58.6) | 45.5 (44.2-47.1) | -31.6 (-32.9 to -30.8) | -2.13 (-2.24 to -2.06) | -16.8 (-17.9 to -15.7) | -2.43 (-2.62 to -2.24) |
| Deoria | 54.7 (48.7-60.7) | 50.6 (45.1-55.8) | 41.4 (40.2-42.6) | -25.1 (-26.7 to -24.5) | -1.62 (-1.73 to -1.56) | -19.1 (-20.4 to -18.4) | -2.83 (-3.04 to -2.70) |
| Etah | 67.9 (62.5-72.8) | 62.5 (57.9-67.1) | 51.3 (49.9-52.6) | -25.4 (-26.4 to -24.6) | -1.64 (-1.71 to -1.57) | -18.8 (-19.8 to -17.9) | -2.77 (-2.94 to -2.62) |
| Etawah | 67.2 (62.1-73.2) | 62.5 (58.0-66.4) | 52.1 (50.9-53.2) | -23.4 (-24.3 to -22.5) | -1.49 (-1.55 to -1.41) | -17.5 (-18.7 to -16.8) | -2.56 (-2.75 to -2.42) |
| Faizabad | 64.0 (58.3-69.1) | 52.8 (49.1-56.6) | 52.2 (50.9-53.9) | -19.3 (-20.7 to -18.6) | -1.19 (-1.28 to -1.13) | -1.9 (-3.1 to -1.0) | -0.14 (-0.31 to 0.01) |
| Farrukhabad | 67.4 (62.0-73.0) | 63.4 (59.0-67.5) | 53.9 (52.4-55.0) | -20.8 (-21.9 to -19.9) | -1.30 (-1.37 to -1.23) | -15.9 (-17.0 to -15.2) | -2.29 (-2.46 to -2.16) |
| Fatehpur | 66.1 (59.9-71.6) | 56.6 (52.4-60.7) | 47.0 (45.7-48.2) | -29.8 (-31.4 to -29.1) | -1.98 (-2.11 to -1.92) | -18.0 (-18.9 to -17.2) | -2.63 (-2.79 to -2.49) |
| Firozabad | 67.2 (61.0-72.6) | 60.6 (55.2-65.7) | 48.4 (46.5-50.3) | -28.9 (-29.9 to -28.0) | -1.91 (-1.99 to -1.84) | -21.0 (-22.5 to -20.1) | -3.16 (-3.40 to -2.98) |
| Gautam Buddha Nagar | 61.1 (54.1-68.3) | 51.5 (44.6-57.7) | 34.9 (31.6-38.3) | -43.6 (-45.4 to -42.6) | -3.23 (-3.39 to -3.11) | -32.9 (-34.7 to -31.4) | -5.39 (-5.70 to -5.05) |
| Ghaziabad | 64.1 (57.9-69.5) | 53.6 (47.9-59.0) | 37.7 (35.4-40.2) | -42.0 (-43.2 to -41.2) | -3.07 (-3.17 to -2.98) | -30.4 (-32.0 to -29.5) | -4.88 (-5.16 to -4.68) |
| Ghazipur | 57.1 (50.6-63.2) | 53.1 (48.4-57.4) | 45.3 (44.3-46.2) | -21.5 (-23.0 to -20.2) | -1.35 (-1.45 to -1.25) | -15.5 (-16.7 to -14.9) | -2.23 (-2.42 to -2.11) |
| Gonda | 68.8 (64.3-73.0) | 60.2 (56.5-63.7) | 60.1 (59.2-60.9) | -13.5 (-14.5 to -12.8) | -0.79 (-0.85 to -0.73) | -1.0 (-2.0 to -0.3) | -0.01 (-0.14 to 0.09) |
| Gorakhpur | 58.8 (54.0-63.5) | 50.6 (46.5-54.7) | 45.0 (43.8-46.0) | -24.3 (-25.4 to -23.5) | -1.55 (-1.63 to -1.49) | -11.9 (-13.2 to -10.8) | -1.65 (-1.84 to -1.47) |
| Hamirpur | 66.7 (61.8-71.2) | 61.6 (57.7-65.3) | 51.0 (50.0-52.1) | -24.5 (-25.4 to -23.8) | -1.57 (-1.63 to -1.51) | -18.1 (-19.1 to -17.5) | -2.66 (-2.82 to -2.55) |
| Hapur | 66.1 (60.9-70.5) | 57.4 (53.2-61.8) | 42.6 (41.3-44.2) | -36.5 (-37.4 to -35.8) | -2.55 (-2.63 to -2.48) | -26.7 (-27.8 to -25.8) | -4.17 (-4.35 to -3.99) |
| Hardoi | 65.8 (60.8-70.3) | 61.0 (57.2-64.7) | 54.0 (53.3-54.9) | -18.8 (-20.1 to -18.1) | -1.15 (-1.24 to -1.09) | -12.3 (-13.3 to -11.5) | -1.71 (-1.87 to -1.58) |
| Hathras | 66.9 (61.9-72.3) | 60.3 (55.0-65.0) | 47.4 (45.9-48.7) | -30.1 (-30.8 to -29.1) | -2.01 (-2.06 to -1.92) | -22.3 (-23.4 to -21.5) | -3.37 (-3.56 to -3.23) |
| Jalaun | 64.4 (58.9-69.8) | 59.6 (54.4-64.5) | 52.0 (50.5-53.7) | -20.1 (-21.3 to -19.0) | -1.24 (-1.33 to -1.16) | -13.5 (-14.9 to -12.6) | -1.91 (-2.12 to -1.74) |
| Jaunpur | 61.0 (55.6-66.5) | 50.2 (46.1-54.5) | 47.6 (46.5-48.7) | -22.8 (-24.0 to -22.0) | -1.44 (-1.53 to -1.38) | -6.1 (-7.1 to -5.0) | -0.75 (-0.89 to -0.59) |
| Jhansi | 63.4 (58.7-68.4) | 56.9 (53.0-60.4) | 49.8 (48.8-50.8) | -22.4 (-23.4 to -21.5) | -1.41 (-1.48 to -1.34) | -13.5 (-14.4 to -12.6) | -1.90 (-2.03 to -1.74) |
| Kannauj | 66.6 (59.7-73.5) | 63.1 (57.8-68.1) | 52.4 (50.9-53.6) | -22.1 (-23.8 to -21.1) | -1.39 (-1.51 to -1.31) | -17.7 (-18.7 to -16.9) | -2.59 (-2.74 to -2.44) |
| Kanpur Dehat | 66.0 (60.8-71.3) | 61.8 (57.3-66.2) | 51.2 (50.2-52.4) | -23.2 (-24.1 to -22.4) | -1.47 (-1.54 to -1.41) | -17.9 (-19.0 to -17.3) | -2.63 (-2.80 to -2.51) |
| Kanpur Nagar | 62.6 (56.4-68.6) | 56.9 (51.8-61.9) | 46.1 (43.8-48.2) | -27.3 (-28.5 to -26.3) | -1.79 (-1.87 to -1.70) | -19.9 (-21.2 to -18.5) | -2.96 (-3.17 to -2.72) |
| Kasganj | 68.7 (63.1-74.1) | 64.3 (59.4-69.2) | 54.7 (52.9-56.2) | -21.3 (-22.6 to -20.5) | -1.33 (-1.42 to -1.27) | -15.8 (-17.0 to -15.0) | -2.28 (-2.45 to -2.14) |
| Kaushambi | 62.4 (56.1-68.1) | 51.7 (47.7-55.8) | 47.1 (45.2-49.2) | -25.3 (-26.7 to -24.4) | -1.63 (-1.73 to -1.55) | -9.7 (-11.0 to -8.7) | -1.31 (-1.50 to -1.14) |
| Khushinagar | 62.0 (57.2-66.7) | 53.8 (49.3-58.2) | 45.5 (44.3-46.5) | -27.6 (-28.4 to -26.9) | -1.80 (-1.87 to -1.75) | -16.3 (-17.2 to -15.3) | -2.35 (-2.50 to -2.18) |
| Lakhimpur Kheri | 66.9 (62.8-71.0) | 60.3 (56.8-63.7) | 59.6 (58.4-60.7) | -11.9 (-12.6 to -11.2) | -0.68 (-0.72 to -0.63) | -2.1 (-3.1 to -1.3) | -0.17 (-0.30 to -0.04) |
| Lalitpur | 63.8 (57.7-69.8) | 56.7 (51.5-61.9) | 47.8 (46.3-49.7) | -25.9 (-27.2 to -25.0) | -1.68 (-1.77 to -1.60) | -16.4 (-17.7 to -15.2) | -2.38 (-2.58 to -2.17) |
| Lucknow | 61.1 (55.3-65.8) | 53.5 (49.2-57.9) | 47.8 (45.9-49.9) | -22.5 (-24.2 to -21.6) | -1.42 (-1.54 to -1.35) | -11.4 (-12.7 to -10.4) | -1.58 (-1.76 to -1.41) |
| Maharajganj | 70.6 (67.0-74.2) | 56.7 (52.6-60.7) | 52.0 (50.6-53.5) | -27.3 (-28.0 to -26.7) | -1.78 (-1.83 to -1.73) | -9.2 (-10.2 to -8.2) | -1.22 (-1.37 to -1.06) |
| Mahoba | 65.9 (60.9-70.7) | 60.8 (56.9-64.5) | 50.1 (48.9-51.4) | -24.8 (-25.9 to -24.2) | -1.59 (-1.67 to -1.54) | -18.5 (-19.5 to -17.8) | -2.71 (-2.87 to -2.59) |
| Mainpuri | 67.8 (63.2-72.5) | 62.7 (58.0-66.9) | 51.2 (50.0-52.4) | -25.4 (-26.3 to -24.6) | -1.64 (-1.70 to -1.57) | -19.3 (-20.3 to -18.5) | -2.86 (-3.02 to -2.71) |
| Mathura | 67.2 (61.8-72.2) | 59.9 (55.4-64.2) | 48.1 (46.7-49.9) | -29.2 (-30.3 to -28.7) | -1.94 (-2.02 to -1.89) | -20.5 (-21.8 to -19.6) | -3.07 (-3.27 to -2.90) |
| Mau | 56.2 (51.1-61.7) | 51.0 (46.5-55.7) | 43.6 (42.4-45.0) | -23.3 (-24.4 to -22.3) | -1.48 (-1.55 to -1.40) | -15.4 (-16.6 to -14.3) | -2.21 (-2.40 to -2.01) |
| Meerut | 66.2 (61.4-70.7) | 55.5 (51.2-59.4) | 41.5 (40.4-42.7) | -38.1 (-39.0 to -37.5) | -2.70 (-2.77 to -2.64) | -26.0 (-27.2 to -25.3) | -4.04 (-4.25 to -3.90) |
| Mirzapur | 63.1 (57.0-68.5) | 57.1 (52.7-62.0) | 49.7 (48.7-50.5) | -22.1 (-23.2 to -21.5) | -1.39 (-1.47 to -1.34) | -13.9 (-15.1 to -12.8) | -1.96 (-2.15 to -1.78) |
| Moradabad | 64.0 (58.3-69.1) | 56.6 (52.2-60.8) | 45.4 (44.1-46.6) | -29.9 (-31.0 to -29.5) | -1.99 (-2.07 to -1.96) | -20.7 (-21.6 to -20.0) | -3.09 (-3.25 to -2.96) |
| Muzaffarnagar | 66.6 (61.7-71.7) | 54.5 (49.7-58.9) | 43.0 (41.9-44.2) | -36.4 (-37.2 to -35.8) | -2.54 (-2.61 to -2.49) | -21.9 (-23.2 to -21.1) | -3.31 (-3.52 to -3.15) |
| Pilibhit | 66.2 (60.4-71.2) | 57.3 (52.8-61.9) | 51.5 (50.4-52.8) | -23.0 (-24.1 to -22.1) | -1.46 (-1.53 to -1.38) | -10.8 (-11.8 to -10.1) | -1.48 (-1.62 to -1.35) |
| Pratapgarh | 62.6 (57.6-67.3) | 50.8 (47.6-54.3) | 48.8 (47.7-49.9) | -22.9 (-24.1 to -22.5) | -1.45 (-1.53 to -1.41) | -4.9 (-5.8 to -4.0) | -0.58 (-0.70 to -0.44) |
| Rae Bareli | 64.4 (58.5-69.6) | 54.8 (50.9-58.7) | 48.2 (46.8-49.5) | -26.0 (-27.4 to -25.4) | -1.69 (-1.79 to -1.63) | -12.9 (-13.8 to -11.8) | -1.81 (-1.94 to -1.62) |
| Rampur | 65.3 (59.5-70.5) | 57.5 (52.7-61.3) | 48.2 (47.1-49.4) | -27.0 (-28.1 to -26.2) | -1.76 (-1.84 to -1.69) | -17.0 (-18.2 to -16.4) | -2.47 (-2.66 to -2.36) |
| Saharanpur | 66.8 (61.8-72.1) | 54.6 (49.6-59.3) | 42.7 (41.4-43.7) | -37.1 (-38.0 to -36.5) | -2.60 (-2.68 to -2.55) | -22.8 (-24.0 to -21.8) | -3.46 (-3.66 to -3.28) |
| Sambhal | 65.2 (59.7-70.1) | 60.1 (56.1-64.3) | 48.1 (47.1-49.2) | -27.1 (-28.0 to -26.5) | -1.77 (-1.83 to -1.72) | -20.9 (-21.9 to -20.1) | -3.13 (-3.29 to -2.99) |
| Sant Kabir Nagar | 62.0 (56.1-68.2) | 51.4 (46.5-56.4) | 48.3 (46.9-50.0) | -22.9 (-24.2 to -21.8) | -1.45 (-1.54 to -1.36) | -6.8 (-8.4 to -5.9) | -0.87 (-1.09 to -0.71) |
| Shahjahanpur | 66.1 (60.7-70.9) | 60.5 (56.2-64.4) | 54.7 (53.6-55.5) | -18.1 (-19.1 to -17.4) | -1.10 (-1.17 to -1.05) | -10.4 (-11.4 to -9.7) | -1.41 (-1.55 to -1.28) |
| Shamli | 68.2 (63.6-72.5) | 55.2 (50.3-60.0) | 44.6 (43.2-46.0) | -35.6 (-36.4 to -35.1) | -2.47 (-2.53 to -2.42) | -20.1 (-21.6 to -19.1) | -3.00 (-3.23 to -2.82) |
| Shravasti | 70.9 (66.6-74.8) | 61.4 (57.9-64.5) | 61.8 (60.3-63.0) | -13.8 (-14.6 to -13.2) | -0.81 (-0.86 to -0.76) | -0.3 (-1.3 to 0.6) | 0.10 (-0.04 to 0.23) |
| Siddharth Nagar | 70.9 (66.9-74.8) | 58.9 (55.0-62.5) | 57.1 (56.3-58.0) | -20.5 (-21.3 to -20.0) | -1.27 (-1.33 to -1.23) | -4.0 (-5.2 to -3.4) | -0.45 (-0.61 to -0.35) |
| Sitapur | 65.5 (60.6-69.9) | 60.2 (56.7-63.3) | 58.7 (57.7-59.6) | -11.2 (-12.2 to -10.5) | -0.64 (-0.70 to -0.58) | -3.4 (-4.3 to -2.7) | -0.35 (-0.48 to -0.25) |
| Sonbhadra | 61.9 (54.8-68.6) | 57.8 (52.0-63.8) | 46.9 (45.6-48.3) | -25.0 (-26.6 to -24.0) | -1.61 (-1.72 to -1.52) | -19.7 (-21.0 to -18.6) | -2.93 (-3.13 to -2.73) |
| Sultanpur | 61.1 (55.4-66.3) | 49.3 (45.1-53.1) | 48.6 (47.2-50.0) | -21.4 (-22.6 to -20.5) | -1.34 (-1.42 to -1.27) | -2.2 (-3.6 to -0.9) | -0.19 (-0.38 to 0.01) |
| Unnao | 65.1 (59.9-69.9) | 57.8 (53.9-61.7) | 48.6 (47.6-49.6) | -26.1 (-27.2 to -25.5) | -1.69 (-1.77 to -1.64) | -16.7 (-17.7 to -15.9) | -2.42 (-2.58 to -2.28) |
| Varanasi | 58.1 (50.9-64.3) | 51.3 (46.0-56.7) | 45.6 (43.3-47.8) | -22.3 (-24.0 to -21.4) | -1.41 (-1.53 to -1.34) | -12.0 (-13.5 to -10.8) | -1.68 (-1.89 to -1.46) |
| **Rajasthan** | **55.9 (53.1-60.0)** | **46.1 (44.9-47.4)** | **37.3 (35.7-38.9)** | **-34.3 (-34.7 to -34.0)** | **-2.35 (-2.38 to -2.33)** | **-20.1 (-20.6 to -19.7)** | **-3.03 (-3.69 to -2.33)** |
| Ajmer | 52.7 (48.0-57.9) | 45.7 (40.8-50.6) | 34.9 (33.7-36.0) | -34.7 (-35.8 to -33.8) | -2.40 (-2.48 to -2.31) | -24.4 (-26.0 to -23.5) | -3.76 (-4.02 to -3.58) |
| Alwar | 56.6 (52.5-61.0) | 45.2 (41.6-49.1) | 37.4 (36.7-38.2) | -34.8 (-35.7 to -34.1) | -2.40 (-2.48 to -2.34) | -18.1 (-19.0 to -17.1) | -2.65 (-2.80 to -2.47) |
| Banswara | 57.5 (52.8-62.6) | 49.0 (44.5-52.8) | 45.2 (43.9-46.3) | -22.3 (-23.5 to -21.4) | -1.41 (-1.48 to -1.33) | -8.8 (-10.1 to -7.9) | -1.16 (-1.36 to -1.01) |
| Baran | 63.6 (58.8-69.2) | 51.0 (46.6-54.9) | 43.7 (42.4-44.9) | -32.1 (-33.0 to -31.5) | -2.17 (-2.24 to -2.12) | -15.0 (-16.4 to -14.3) | -2.15 (-2.36 to -2.01) |
| Barmer | 59.4 (52.6-65.2) | 47.8 (42.9-53.0) | 42.4 (40.7-44.3) | -29.3 (-30.8 to -28.3) | -1.95 (-2.06 to -1.86) | -11.9 (-13.3 to -10.7) | -1.66 (-1.86 to -1.45) |
| Bharatpur | 60.0 (54.2-65.3) | 49.0 (45.1-52.9) | 44.3 (42.9-45.7) | -27.0 (-28.3 to -26.3) | -1.76 (-1.86 to -1.70) | -10.5 (-11.9 to -9.4) | -1.44 (-1.63 to -1.24) |
| Bhilwara | 56.5 (51.6-61.3) | 47.7 (43.3-51.6) | 35.5 (34.4-36.7) | -37.9 (-38.9 to -37.2) | -2.68 (-2.77 to -2.61) | -26.4 (-27.7 to -25.5) | -4.11 (-4.34 to -3.94) |
| Bikaner | 49.0 (42.4-55.3) | 38.4 (33.7-43.1) | 32.2 (31.1-33.2) | -35.0 (-36.5 to -34.2) | -2.43 (-2.55 to -2.35) | -16.7 (-18.4 to -15.7) | -2.44 (-2.69 to -2.24) |
| Bundi | 59.7 (55.1-64.3) | 45.6 (40.0-51.0) | 33.8 (32.6-34.9) | -44.3 (-45.3 to -43.6) | -3.29 (-3.38 to -3.21) | -26.7 (-28.4 to -25.8) | -4.19 (-4.47 to -3.99) |
| Chittorgarh | 58.2 (52.8-63.3) | 48.9 (44.4-53.0) | 36.5 (35.1-38.1) | -38.1 (-39.3 to -37.5) | -2.70 (-2.80 to -2.64) | -26.3 (-27.5 to -25.4) | -4.09 (-4.30 to -3.92) |
| Churu | 51.0 (45.8-56.3) | 41.7 (37.0-46.4) | 32.0 (30.9-33.2) | -38.1 (-39.1 to -37.3) | -2.70 (-2.78 to -2.62) | -24.1 (-25.8 to -23.1) | -3.70 (-3.98 to -3.51) |
| Dausa | 56.5 (51.1-62.1) | 45.6 (40.6-50.3) | 36.1 (34.6-37.7) | -37.0 (-38.0 to -36.0) | -2.60 (-2.68 to -2.50) | -21.7 (-23.1 to -20.2) | -3.28 (-3.50 to -3.00) |
| Dholpur | 61.1 (55.8-65.6) | 51.1 (47.0-55.0) | 47.3 (46.0-48.9) | -23.6 (-24.6 to -22.9) | -1.50 (-1.57 to -1.44) | -8.4 (-9.5 to -7.4) | -1.10 (-1.26 to -0.95) |
| Dungarpur | 57.7 (52.7-62.5) | 51.7 (47.3-55.8) | 44.4 (42.9-46.0) | -23.9 (-25.0 to -23.0) | -1.52 (-1.60 to -1.45) | -14.9 (-16.3 to -14.1) | -2.13 (-2.34 to -1.99) |
| Hanumangarh | 53.9 (47.8-59.7) | 39.6 (34.7-44.8) | 34.0 (32.8-35.5) | -37.6 (-39.0 to -37.0) | -2.66 (-2.77 to -2.59) | -14.7 (-16.6 to -13.3) | -2.11 (-2.40 to -1.85) |
| Jaipur | 52.1 (47.7-56.4) | 42.9 (38.8-47.6) | 31.3 (30.5-32.2) | -40.8 (-41.8 to -40.1) | -2.95 (-3.04 to -2.88) | -27.8 (-29.0 to -27.0) | -4.38 (-4.58 to -4.21) |
| Jaisalmer | 53.3 (44.7-62.1) | 35.8 (30.8-40.8) | 30.8 (28.5-32.6) | -42.8 (-44.9 to -41.9) | -3.16 (-3.34 to -3.05) | -14.5 (-17.1 to -12.8) | -2.09 (-2.48 to -1.78) |
| Jalore | 60.4 (54.6-65.0) | 52.4 (48.5-56.4) | 45.5 (44.0-46.8) | -25.4 (-26.6 to -24.8) | -1.64 (-1.72 to -1.59) | -14.0 (-15.1 to -13.2) | -1.99 (-2.15 to -1.85) |
| Jhalawar | 60.0 (54.5-65.5) | 49.3 (45.2-53.4) | 38.0 (36.7-39.5) | -37.5 (-38.6 to -36.8) | -2.65 (-2.74 to -2.57) | -23.8 (-24.9 to -23.0) | -3.65 (-3.82 to -3.49) |
| Jhunjhunu | 51.7 (46.2-57.1) | 42.0 (37.7-46.2) | 31.3 (30.0-32.7) | -40.3 (-41.4 to -39.6) | -2.91 (-3.00 to -2.83) | -26.3 (-27.7 to -25.2) | -4.11 (-4.34 to -3.89) |
| Jodhpur | 55.6 (49.4-61.4) | 44.6 (40.5-48.8) | 36.7 (35.6-37.8) | -34.9 (-35.9 to -34.3) | -2.42 (-2.50 to -2.35) | -18.7 (-19.9 to -17.7) | -2.76 (-2.95 to -2.57) |
| Karauli | 62.1 (56.5-67.2) | 51.7 (47.1-56.1) | 45.7 (44.6-46.9) | -27.3 (-28.4 to -26.8) | -1.78 (-1.87 to -1.74) | -12.3 (-13.5 to -11.2) | -1.72 (-1.90 to -1.53) |
| Kota | 60.8 (56.2-65.9) | 47.0 (41.9-52.1) | 35.4 (34.2-36.5) | -42.7 (-43.6 to -41.9) | -3.14 (-3.22 to -3.05) | -25.4 (-26.7 to -24.2) | -3.95 (-4.15 to -3.71) |
| Nagaur | 51.7 (46.9-56.0) | 45.1 (40.3-49.7) | 35.8 (34.5-37.0) | -31.5 (-32.7 to -30.6) | -2.13 (-2.22 to -2.05) | -21.3 (-22.7 to -20.4) | -3.21 (-3.43 to -3.03) |
| Pali | 58.3 (52.8-63.7) | 50.8 (46.6-54.9) | 39.9 (38.8-41.5) | -32.4 (-33.6 to -31.6) | -2.20 (-2.29 to -2.12) | -22.3 (-23.7 to -21.6) | -3.37 (-3.62 to -3.24) |
| Pratapgarh | 57.5 (52.1-63.0) | 48.9 (44.5-53.0) | 41.5 (40.0-43.0) | -28.7 (-29.8 to -27.8) | -1.90 (-1.98 to -1.82) | -16.0 (-17.6 to -15.2) | -2.31 (-2.56 to -2.17) |
| Rajsamand | 58.6 (52.5-64.6) | 52.1 (47.7-56.2) | 40.2 (38.6-42.0) | -32.1 (-33.3 to -31.2) | -2.18 (-2.26 to -2.10) | -23.6 (-24.9 to -22.8) | -3.61 (-3.83 to -3.45) |
| Sawai Madhopur | 59.6 (54.2-64.6) | 48.4 (43.8-53.2) | 39.5 (38.3-40.7) | -34.7 (-35.8 to -34.1) | -2.40 (-2.48 to -2.34) | -19.3 (-20.6 to -18.0) | -2.86 (-3.06 to -2.63) |
| Sikar | 50.6 (45.1-55.7) | 43.2 (38.4-47.6) | 32.1 (31.0-33.4) | -37.4 (-38.6 to -36.4) | -2.63 (-2.74 to -2.54) | -26.4 (-27.8 to -25.5) | -4.12 (-4.36 to -3.94) |
| Sirohi | 59.7 (53.6-65.2) | 53.8 (49.6-57.9) | 44.5 (42.4-46.4) | -26.3 (-27.4 to -25.5) | -1.71 (-1.79 to -1.64) | -18.2 (-19.4 to -17.2) | -2.67 (-2.87 to -2.49) |
| Sri Ganganagar | 52.2 (44.5-59.6) | 35.9 (31.7-40.1) | 33.7 (31.8-35.6) | -36.1 (-38.2 to -35.2) | -2.53 (-2.70 to -2.43) | -6.8 (-8.6 to -5.2) | -0.88 (-1.13 to -0.62) |
| Tonk | 56.0 (51.3-61.0) | 44.7 (39.7-49.8) | 33.4 (31.9-34.9) | -41.2 (-42.0 to -40.6) | -2.99 (-3.06 to -2.92) | -26.1 (-27.8 to -25.2) | -4.08 (-4.36 to -3.89) |
| Udaipur | 58.7 (54.2-63.1) | 52.8 (48.9-56.7) | 42.6 (41.5-43.9) | -28.3 (-29.2 to -27.4) | -1.86 (-1.93 to -1.79) | -20.2 (-21.4 to -19.7) | -3.02 (-3.21 to -2.92) |
| **Chhattisgarh** | **59.5 (54.8-65.0)** | **49.4 (47.1-52.2)** | **38.6 (36.5-41.5)** | **-36.0 (-36.8 to -35.5)** | **-2.50 (-2.57 to -2.46)** | **-22.7 (-23.4 to -22.2)** | **-3.51 (-4.63 to -2.27)** |
| Balod | 58.9 (51.2-66.2) | 49.3 (44.1-54.3) | 43.1 (41.4-44.7) | -27.5 (-29.2 to -26.6) | -1.81 (-1.93 to -1.72) | -13.3 (-15.3 to -12.0) | -1.88 (-2.18 to -1.65) |
| Baloda Bazar | 61.7 (54.5-68.1) | 49.3 (44.1-54.4) | 38.8 (37.7-40.1) | -37.9 (-39.3 to -37.1) | -2.68 (-2.80 to -2.60) | -22.0 (-23.5 to -20.8) | -3.34 (-3.57 to -3.11) |
| Balrampur | 56.0 (47.8-64.6) | 49.7 (44.0-55.8) | 34.3 (32.8-36.0) | -39.4 (-41.1 to -38.5) | -2.83 (-2.97 to -2.73) | -31.8 (-33.0 to -30.9) | -5.15 (-5.36 to -4.94) |
| Bastar | 52.6 (45.1-59.9) | 50.2 (43.1-57.3) | 39.9 (38.0-41.8) | -24.8 (-26.5 to -23.7) | -1.60 (-1.72 to -1.50) | -21.2 (-23.1 to -19.9) | -3.21 (-3.50 to -2.95) |
| Bemetara | 62.2 (54.8-69.1) | 47.7 (42.7-52.6) | 40.7 (39.4-42.5) | -35.4 (-36.6 to -34.3) | -2.46 (-2.56 to -2.36) | -15.4 (-17.2 to -14.3) | -2.22 (-2.49 to -2.03) |
| Bijapur | 54.1 (45.3-63.2) | 53.1 (45.8-59.7) | 44.7 (43.0-46.5) | -17.8 (-20.4 to -16.6) | -1.10 (-1.26 to -0.99) | -16.4 (-18.8 to -15.2) | -2.40 (-2.76 to -2.17) |
| Bilaspur | 61.7 (55.1-68.2) | 46.9 (41.9-52.1) | 35.8 (34.5-36.9) | -42.8 (-44.2 to -42.3) | -3.15 (-3.27 to -3.09) | -24.5 (-26.1 to -23.6) | -3.78 (-4.04 to -3.59) |
| Dantewada | 50.6 (41.9-58.7) | 48.0 (41.0-55.0) | 37.8 (36.2-39.6) | -25.8 (-27.8 to -24.5) | -1.68 (-1.82 to -1.56) | -21.7 (-23.8 to -20.5) | -3.30 (-3.64 to -3.05) |
| Dhamtari | 58.2 (51.0-64.7) | 47.9 (42.5-52.9) | 38.9 (37.6-40.2) | -33.9 (-35.2 to -33.2) | -2.33 (-2.44 to -2.26) | -19.5 (-20.9 to -18.5) | -2.91 (-3.11 to -2.72) |
| Durg | 59.6 (51.5-66.8) | 47.3 (41.7-53.2) | 40.0 (38.0-42.3) | -33.6 (-35.0 to -32.6) | -2.31 (-2.41 to -2.21) | -16.1 (-17.3 to -14.6) | -2.33 (-2.51 to -2.06) |
| Gariaband | 58.5 (53.1-64.2) | 51.6 (46.9-56.3) | 42.2 (40.9-43.6) | -28.6 (-29.7 to -27.7) | -1.89 (-1.97 to -1.81) | -19.0 (-20.3 to -18.2) | -2.82 (-3.02 to -2.66) |
| Janjgir-Champa | 63.0 (55.8-70.5) | 53.1 (47.8-59.4) | 39.2 (37.7-40.5) | -38.6 (-39.6 to -37.8) | -2.75 (-2.83 to -2.66) | -27.1 (-28.2 to -25.9) | -4.24 (-4.44 to -4.00) |
| Jashpur | 57.4 (49.0-65.1) | 51.9 (46.2-57.9) | 34.8 (33.4-36.4) | -40.2 (-41.6 to -39.3) | -2.90 (-3.01 to -2.80) | -33.9 (-35.1 to -32.9) | -5.56 (-5.78 to -5.35) |
| Kabirdham | 62.7 (54.5-70.0) | 46.5 (40.4-52.7) | 41.6 (39.6-43.2) | -34.4 (-36.3 to -33.5) | -2.38 (-2.53 to -2.29) | -11.2 (-13.3 to -9.8) | -1.56 (-1.86 to -1.30) |
| Kondagaon | 53.9 (45.8-62.7) | 47.3 (40.9-54.0) | 37.7 (36.1-39.4) | -30.6 (-32.4 to -29.3) | -2.06 (-2.19 to -1.94) | -20.9 (-23.0 to -19.7) | -3.16 (-3.48 to -2.91) |
| Korba | 61.3 (54.4-67.8) | 49.1 (44.3-54.8) | 33.7 (32.6-35.1) | -45.8 (-46.9 to -45.0) | -3.44 (-3.55 to -3.36) | -32.0 (-33.1 to -31.0) | -5.19 (-5.37 to -4.96) |
| Korea | 60.6 (54.1-67.1) | 48.5 (43.2-54.5) | 33.7 (32.8-34.8) | -45.3 (-46.5 to -44.6) | -3.39 (-3.51 to -3.31) | -31.4 (-33.0 to -30.5) | -5.06 (-5.35 to -4.88) |
| Mahasamund | 60.2 (54.5-66.6) | 52.3 (47.4-57.0) | 41.2 (40.0-42.1) | -32.3 (-33.6 to -31.4) | -2.19 (-2.30 to -2.11) | -22.0 (-23.2 to -21.1) | -3.33 (-3.52 to -3.15) |
| Mungeli | 62.3 (54.1-69.7) | 47.0 (41.1-52.9) | 39.4 (37.5-41.4) | -37.4 (-39.0 to -36.9) | -2.65 (-2.78 to -2.58) | -16.7 (-18.6 to -15.4) | -2.44 (-2.73 to -2.20) |
| Narayanpur | 54.7 (45.1-64.0) | 51.2 (44.5-58.0) | 43.5 (41.6-45.6) | -20.8 (-23.3 to -19.5) | -1.31 (-1.47 to -1.20) | -15.6 (-17.5 to -14.3) | -2.27 (-2.54 to -2.02) |
| North Bastar Kanker | 53.5 (45.1-61.8) | 47.4 (41.8-53.6) | 40.4 (39.1-42.2) | -25.0 (-27.0 to -23.9) | -1.62 (-1.76 to -1.51) | -15.5 (-17.3 to -14.5) | -2.24 (-2.50 to -2.05) |
| Raigarh | 61.7 (55.5-68.9) | 55.3 (50.0-61.0) | 39.3 (38.0-40.7) | -37.1 (-38.0 to -36.3) | -2.61 (-2.68 to -2.52) | -29.7 (-30.8 to -28.7) | -4.74 (-4.93 to -4.52) |
| Raipur | 59.8 (51.4-67.0) | 47.2 (41.9-53.2) | 38.0 (36.1-39.8) | -37.1 (-38.9 to -36.3) | -2.62 (-2.77 to -2.53) | -20.1 (-21.9 to -18.9) | -3.01 (-3.30 to -2.79) |
| Rajnandgaon | 59.5 (52.1-66.4) | 49.2 (44.3-54.1) | 44.5 (43.0-46.0) | -25.9 (-27.8 to -24.9) | -1.68 (-1.82 to -1.60) | -10.4 (-11.9 to -9.0) | -1.42 (-1.64 to -1.19) |
| Sukma | 50.3 (41.9-59.2) | 50.0 (42.3-57.5) | 40.2 (38.2-42.1) | -20.4 (-22.8 to -19.0) | -1.28 (-1.44 to -1.16) | -20.1 (-22.2 to -18.9) | -3.03 (-3.34 to -2.77) |
| Surajpur | 58.0 (51.0-65.2) | 47.3 (41.9-53.5) | 31.0 (30.0-32.0) | -47.4 (-48.9 to -46.8) | -3.62 (-3.76 to -3.54) | -35.4 (-36.7 to -34.4) | -5.87 (-6.11 to -5.64) |
| Surguja | 58.5 (50.5-65.4) | 49.0 (43.1-55.1) | 31.6 (30.4-33.1) | -46.7 (-48.2 to -45.8) | -3.55 (-3.69 to -3.44) | -36.2 (-37.5 to -35.2) | -6.03 (-6.27 to -5.80) |
| **Odisha** | **53.3 (51.4-55.4)** | **44.7 (43.2-46.5)** | **35.9 (34.2-37.8)** | **-33.7 (-34.1 to -33.3)** | **-2.30 (-2.34 to -2.27)** | **-20.7 (-21.6 to -20.1)** | **-3.15 (-3.97 to -2.23)** |
| Angul | 54.0 (49.1-58.8) | 47.7 (43.0-52.3) | 35.6 (34.6-36.8) | -34.9 (-36.2 to -34.1) | -2.41 (-2.52 to -2.34) | -26.2 (-27.6 to -25.4) | -4.08 (-4.32 to -3.91) |
| Balangir | 56.9 (51.2-62.0) | 51.6 (47.7-55.2) | 44.2 (42.9-45.3) | -23.1 (-24.4 to -22.4) | -1.47 (-1.55 to -1.40) | -15.2 (-16.1 to -14.4) | -2.18 (-2.30 to -2.03) |
| Balasore | 53.1 (47.6-58.5) | 42.2 (37.8-46.6) | 37.7 (35.9-39.2) | -29.9 (-31.0 to -28.8) | -1.99 (-2.08 to -1.89) | -11.5 (-13.1 to -10.6) | -1.59 (-1.83 to -1.44) |
| Bargarh | 58.7 (52.9-64.4) | 54.3 (49.7-58.6) | 42.5 (41.4-43.8) | -28.3 (-29.5 to -27.6) | -1.87 (-1.95 to -1.81) | -22.5 (-23.6 to -21.6) | -3.42 (-3.59 to -3.25) |
| Bhadrak | 53.1 (47.3-58.8) | 38.6 (34.5-43.2) | 33.3 (31.9-34.7) | -38.1 (-39.6 to -37.3) | -2.71 (-2.83 to -2.61) | -14.6 (-16.1 to -13.4) | -2.10 (-2.31 to -1.87) |
| Boudh | 56.1 (50.3-62.0) | 49.8 (45.0-54.0) | 42.1 (40.5-43.9) | -25.7 (-27.0 to -24.5) | -1.66 (-1.75 to -1.56) | -16.1 (-17.4 to -15.4) | -2.34 (-2.52 to -2.19) |
| Cuttack | 51.5 (46.8-56.7) | 36.1 (31.6-41.3) | 24.4 (23.1-25.9) | -53.4 (-54.3 to -52.7) | -4.29 (-4.38 to -4.19) | -33.0 (-34.6 to -31.9) | -5.39 (-5.68 to -5.14) |
| Deogarh | 54.0 (47.0-60.5) | 51.6 (46.4-56.8) | 36.6 (34.9-38.6) | -32.9 (-34.7 to -31.7) | -2.25 (-2.38 to -2.14) | -29.8 (-31.2 to -28.4) | -4.76 (-5.01 to -4.48) |
| Dhenkanal | 53.5 (48.2-58.4) | 43.3 (38.3-48.4) | 31.0 (29.9-32.2) | -42.9 (-44.0 to -42.1) | -3.15 (-3.25 to -3.06) | -29.1 (-30.4 to -28.3) | -4.64 (-4.85 to -4.45) |
| Gajapati | 48.3 (41.9-54.7) | 43.4 (37.7-49.1) | 39.1 (36.5-41.6) | -19.8 (-21.7 to -18.6) | -1.23 (-1.36 to -1.13) | -10.5 (-12.9 to -9.0) | -1.46 (-1.80 to -1.19) |
| Ganjam | 52.5 (46.9-58.3) | 40.4 (35.5-45.5) | 35.7 (33.7-37.6) | -32.7 (-33.9 to -31.9) | -2.23 (-2.32 to -2.15) | -12.2 (-14.2 to -11.0) | -1.72 (-2.00 to -1.49) |
| Jagatsinghapur | 51.9 (46.0-56.9) | 32.3 (27.6-37.0) | 22.1 (21.0-23.5) | -58.2 (-59.3 to -57.6) | -4.89 (-5.02 to -4.80) | -32.1 (-34.6 to -30.8) | -5.22 (-5.68 to -4.94) |
| Jajapur | 53.2 (47.9-58.1) | 39.9 (35.9-43.6) | 31.8 (30.7-33.1) | -41.1 (-42.4 to -40.5) | -2.98 (-3.09 to -2.91) | -21.1 (-22.9 to -20.1) | -3.18 (-3.47 to -2.99) |
| Jharsuguda | 57.2 (51.3-64.0) | 54.3 (49.3-59.0) | 39.1 (37.5-40.7) | -32.4 (-34.1 to -31.6) | -2.21 (-2.33 to -2.12) | -28.7 (-30.1 to -28.0) | -4.56 (-4.80 to -4.40) |
| Kalahandi | 55.3 (50.5-60.0) | 51.4 (47.4-55.6) | 44.6 (43.4-45.8) | -20.2 (-21.4 to -19.5) | -1.26 (-1.33 to -1.20) | -14.0 (-15.4 to -13.1) | -1.99 (-2.19 to -1.83) |
| Kandhamal | 53.0 (47.9-58.1) | 43.9 (39.7-47.7) | 38.8 (37.4-40.5) | -27.5 (-29.0 to -26.5) | -1.80 (-1.91 to -1.72) | -12.4 (-13.9 to -11.4) | -1.73 (-1.95 to -1.56) |
| Kendrapara | 51.2 (44.4-58.1) | 32.9 (28.1-38.2) | 25.4 (23.8-26.9) | -51.1 (-52.4 to -50.1) | -4.03 (-4.16 to -3.89) | -23.2 (-25.7 to -21.9) | -3.58 (-3.97 to -3.30) |
| Kendujhar | 54.2 (49.5-58.9) | 52.3 (48.3-56.3) | 42.7 (41.6-43.8) | -22.1 (-23.3 to -21.3) | -1.40 (-1.48 to -1.33) | -19.3 (-20.3 to -18.5) | -2.85 (-3.02 to -2.71) |
| Khordha | 50.0 (43.9-56.5) | 33.7 (28.3-39.9) | 21.6 (20.0-23.9) | -57.7 (-59.0 to -57.1) | -4.83 (-4.98 to -4.73) | -36.4 (-39.0 to -35.5) | -6.13 (-6.61 to -5.86) |
| Koraput | 51.6 (45.9-57.3) | 52.3 (47.2-57.8) | 46.3 (44.9-47.7) | -11.1 (-12.7 to -10.2) | -0.64 (-0.73 to -0.57) | -12.3 (-13.6 to -11.5) | -1.72 (-1.91 to -1.57) |
| Malkangiri | 48.3 (40.9-55.4) | 48.9 (42.3-56.1) | 41.8 (40.0-43.4) | -14.0 (-15.9 to -12.9) | -0.83 (-0.95 to -0.74) | -15.3 (-16.9 to -14.3) | -2.21 (-2.44 to -2.02) |
| Mayurbhanj | 53.3 (48.0-57.8) | 52.0 (48.0-55.5) | 45.8 (44.6-46.7) | -14.8 (-16.3 to -13.9) | -0.88 (-0.97 to -0.81) | -12.8 (-14.0 to -11.9) | -1.78 (-1.97 to -1.63) |
| Nabarangpur | 55.4 (48.9-62.4) | 51.8 (45.9-57.5) | 43.5 (41.6-45.1) | -22.2 (-23.7 to -21.0) | -1.40 (-1.50 to -1.30) | -16.8 (-18.3 to -15.6) | -2.45 (-2.68 to -2.23) |
| Nayagarh | 51.4 (46.6-56.4) | 37.7 (33.5-42.0) | 29.6 (28.5-30.9) | -43.3 (-44.2 to -42.6) | -3.19 (-3.27 to -3.11) | -22.3 (-23.9 to -21.3) | -3.39 (-3.65 to -3.19) |
| Nuapada | 57.3 (51.4-63.9) | 50.4 (45.0-55.5) | 42.4 (40.8-44.1) | -26.7 (-28.0 to -25.3) | -1.74 (-1.83 to -1.63) | -16.7 (-18.3 to -15.5) | -2.42 (-2.68 to -2.21) |
| Puri | 50.6 (44.9-57.4) | 32.8 (28.0-38.3) | 20.2 (19.2-21.3) | -60.9 (-61.9 to -60.3) | -5.25 (-5.37 to -5.15) | -39.0 (-40.6 to -37.5) | -6.64 (-6.95 to -6.29) |
| Rayagada | 51.9 (45.8-58.0) | 49.1 (43.9-54.3) | 43.6 (41.9-45.4) | -16.7 (-18.3 to -15.3) | -1.01 (-1.11 to -0.91) | -11.9 (-14.0 to -10.8) | -1.67 (-1.97 to -1.46) |
| Sambalpur | 56.7 (51.2-62.8) | 54.4 (49.5-59.2) | 40.2 (38.3-41.8) | -29.9 (-31.2 to -29.0) | -2.00 (-2.09 to -1.92) | -27.0 (-28.5 to -26.0) | -4.23 (-4.48 to -4.03) |
| Sonepur | 57.7 (51.8-63.0) | 53.5 (49.1-57.6) | 44.1 (42.5-45.8) | -24.4 (-25.8 to -23.7) | -1.57 (-1.66 to -1.50) | -18.6 (-20.0 to -17.8) | -2.74 (-2.96 to -2.60) |
| Sundargarh | 53.7 (47.4-60.2) | 51.9 (47.2-56.9) | 36.2 (34.7-37.6) | -33.3 (-34.8 to -32.4) | -2.28 (-2.40 to -2.19) | -31.1 (-32.5 to -30.2) | -5.00 (-5.26 to -4.82) |
| **Assam** | **56.0 (52.2-61.4)** | **46.1 (44.2-48.5)** | **38.1 (36.2-40.8)** | **-33.0 (-33.6 to -32.4)** | **-2.25 (-2.30 to -2.19)** | **-18.2 (-19.4 to -17.5)** | **-2.73 (-3.80 to -1.60)** |
| Baksa | 55.8 (47.6-63.1) | 47.9 (41.5-54.5) | 36.0 (34.4-37.5) | -36.1 (-38.1 to -35.1) | -2.53 (-2.69 to -2.42) | -25.5 (-27.2 to -24.2) | -3.97 (-4.25 to -3.70) |
| Barpeta | 53.8 (46.3-60.0) | 46.9 (40.7-53.1) | 40.2 (39.1-41.7) | -25.8 (-27.4 to -24.6) | -1.68 (-1.78 to -1.57) | -14.9 (-16.8 to -13.7) | -2.14 (-2.43 to -1.92) |
| Biswanath | 60.7 (52.7-69.1) | 48.6 (39.8-57.1) | 35.7 (34.0-37.6) | -41.9 (-43.0 to -40.8) | -3.06 (-3.16 to -2.94) | -26.9 (-29.2 to -25.8) | -4.25 (-4.62 to -3.99) |
| Bongaigaon | 48.6 (40.6-57.2) | 42.9 (36.2-49.6) | 39.8 (37.3-42.7) | -18.5 (-21.1 to -17.2) | -1.15 (-1.31 to -1.03) | -7.7 (-10.4 to -5.7) | -1.03 (-1.41 to -0.68) |
| Cachar | 57.4 (51.0-64.2) | 46.4 (40.2-52.4) | 39.1 (37.7-40.8) | -32.6 (-33.9 to -31.3) | -2.22 (-2.32 to -2.10) | -16.3 (-17.9 to -15.4) | -2.37 (-2.61 to -2.19) |
| Charaideo | 51.6 (44.5-58.1) | 40.9 (33.4-49.4) | 37.0 (34.4-39.8) | -29.0 (-30.7 to -28.0) | -1.93 (-2.05 to -1.83) | -9.6 (-13.0 to -7.9) | -1.36 (-1.80 to -1.01) |
| Chirang | 49.0 (40.0-58.5) | 44.2 (36.9-51.0) | 41.5 (39.4-43.4) | -15.6 (-18.9 to -14.4) | -0.95 (-1.15 to -0.84) | -6.4 (-9.2 to -4.8) | -0.84 (-1.21 to -0.56) |
| Darrang | 60.5 (53.4-67.4) | 51.0 (44.7-57.6) | 39.7 (38.3-41.3) | -35.1 (-36.7 to -34.4) | -2.44 (-2.56 to -2.36) | -22.8 (-24.3 to -21.7) | -3.48 (-3.72 to -3.26) |
| Dhemaji | 53.3 (46.0-60.0) | 41.7 (33.8-50.3) | 35.7 (34.5-37.3) | -33.7 (-35.7 to -32.7) | -2.32 (-2.47 to -2.21) | -14.4 (-18.1 to -12.2) | -2.12 (-2.64 to -1.68) |
| Dhubri | 45.7 (39.3-52.4) | 40.1 (35.8-45.3) | 38.8 (36.8-41.0) | -15.7 (-17.4 to -13.9) | -0.95 (-1.05 to -0.81) | -4.1 (-5.8 to -2.5) | -0.48 (-0.70 to -0.21) |
| Dibrugarh | 52.4 (45.8-59.5) | 41.2 (33.1-50.4) | 37.1 (35.5-38.8) | -29.9 (-31.4 to -28.8) | -2.00 (-2.11 to -1.90) | -10.0 (-13.2 to -8.2) | -1.41 (-1.84 to -1.06) |
| Dima Hasao | 62.0 (55.1-68.7) | 49.4 (42.3-56.1) | 40.6 (39.2-42.1) | -35.3 (-36.3 to -34.5) | -2.45 (-2.53 to -2.37) | -18.4 (-20.4 to -17.0) | -2.73 (-3.03 to -2.46) |
| Goalpara | 48.1 (41.7-54.2) | 41.3 (36.1-46.3) | 36.7 (35.5-38.2) | -24.3 (-25.9 to -23.1) | -1.56 (-1.67 to -1.46) | -11.6 (-13.8 to -10.2) | -1.62 (-1.94 to -1.37) |
| Golaghat | 56.7 (50.0-62.8) | 44.1 (36.8-51.9) | 31.5 (30.8-32.4) | -45.3 (-46.3 to -44.4) | -3.39 (-3.48 to -3.29) | -29.0 (-31.3 to -28.0) | -4.64 (-5.03 to -4.40) |
| Hailakandi | 56.1 (48.5-63.1) | 44.7 (38.4-51.0) | 38.4 (36.2-40.6) | -32.3 (-33.9 to -31.1) | -2.20 (-2.32 to -2.08) | -14.7 (-16.6 to -13.0) | -2.13 (-2.39 to -1.81) |
| Hojai | 65.9 (56.5-74.6) | 52.5 (44.2-60.3) | 42.5 (39.1-45.9) | -36.2 (-37.8 to -35.0) | -2.54 (-2.66 to -2.41) | -19.5 (-22.6 to -18.1) | -2.95 (-3.41 to -2.64) |
| Jorhat | 55.6 (48.6-62.1) | 43.8 (36.0-52.2) | 33.6 (32.2-35.3) | -40.4 (-41.5 to -39.4) | -2.92 (-3.01 to -2.81) | -23.7 (-26.2 to -22.5) | -3.67 (-4.06 to -3.40) |
| Kamrup | 58.5 (51.4-65.2) | 49.2 (43.0-55.1) | 38.9 (37.8-40.4) | -34.3 (-35.5 to -33.5) | -2.36 (-2.46 to -2.29) | -21.7 (-23.2 to -20.5) | -3.28 (-3.52 to -3.06) |
| Kamrup Metropolitan | 59.4 (50.8-67.9) | 49.2 (41.8-56.5) | 39.2 (35.9-42.6) | -34.5 (-36.5 to -33.9) | -2.40 (-2.55 to -2.32) | -20.7 (-23.3 to -19.4) | -3.14 (-3.54 to -2.86) |
| Karbi Anglong | 62.3 (55.4-68.8) | 49.1 (41.7-56.1) | 36.9 (35.5-38.4) | -41.6 (-42.7 to -40.9) | -3.03 (-3.12 to -2.95) | -25.3 (-27.3 to -24.3) | -3.95 (-4.27 to -3.72) |
| Karimganj | 55.5 (49.5-61.8) | 43.8 (38.9-49.1) | 39.5 (37.4-41.5) | -29.6 (-31.1 to -28.6) | -1.98 (-2.09 to -1.88) | -10.5 (-12.2 to -9.4) | -1.45 (-1.68 to -1.25) |
| Kokrajhar | 46.4 (39.0-53.6) | 42.0 (36.9-47.8) | 41.1 (39.5-42.8) | -12.0 (-14.3 to -10.7) | -0.70 (-0.84 to -0.60) | -2.9 (-5.3 to -1.5) | -0.30 (-0.63 to -0.08) |
| Lakhimpur | 56.5 (48.8-63.7) | 44.6 (36.6-53.3) | 34.3 (33.1-35.5) | -40.1 (-41.4 to -39.2) | -2.89 (-3.00 to -2.79) | -23.6 (-25.8 to -22.2) | -3.65 (-4.00 to -3.35) |
| Majuli | 56.9 (49.8-64.2) | 45.0 (37.5-53.3) | 34.9 (33.3-36.7) | -39.4 (-40.9 to -38.6) | -2.82 (-2.95 to -2.74) | -22.7 (-25.4 to -21.5) | -3.49 (-3.91 to -3.22) |
| Morigaon | 64.5 (55.5-72.7) | 52.5 (45.4-59.4) | 43.1 (41.0-45.1) | -33.9 (-35.3 to -32.9) | -2.33 (-2.44 to -2.24) | -18.4 (-20.1 to -17.2) | -2.73 (-2.98 to -2.49) |
| Nagaon | 65.0 (57.6-71.9) | 52.2 (44.9-59.5) | 41.8 (40.0-43.2) | -36.5 (-37.5 to -35.6) | -2.56 (-2.64 to -2.47) | -20.5 (-22.6 to -19.5) | -3.09 (-3.43 to -2.87) |
| Nalbari | 56.8 (47.9-65.3) | 48.2 (40.9-55.1) | 36.3 (34.3-38.7) | -36.6 (-38.2 to -35.9) | -2.58 (-2.70 to -2.49) | -25.2 (-27.4 to -24.0) | -3.92 (-4.29 to -3.66) |
| Sivasagar | 53.4 (47.2-59.5) | 42.0 (34.5-49.9) | 35.4 (33.7-37.0) | -34.4 (-36.1 to -33.3) | -2.38 (-2.51 to -2.27) | -15.8 (-18.9 to -14.0) | -2.33 (-2.78 to -1.97) |
| Sonitpur | 60.6 (51.3-68.9) | 51.1 (43.0-58.4) | 39.7 (38.0-41.1) | -35.1 (-37.0 to -33.9) | -2.44 (-2.59 to -2.32) | -22.8 (-24.8 to -21.4) | -3.50 (-3.81 to -3.21) |
| South Salmara Mancachar | 48.0 (41.6-54.7) | 39.6 (34.9-44.2) | 36.6 (33.7-39.7) | -24.4 (-26.9 to -23.0) | -1.58 (-1.75 to -1.45) | -8.3 (-10.7 to -6.9) | -1.12 (-1.44 to -0.86) |
| Tinsukia | 47.6 (41.2-53.5) | 37.2 (30.4-45.3) | 32.5 (30.9-34.1) | -32.4 (-34.2 to -31.4) | -2.21 (-2.34 to -2.11) | -12.6 (-15.9 to -10.1) | -1.82 (-2.28 to -1.36) |
| Udalguri | 57.2 (49.5-64.8) | 49.3 (42.5-55.8) | 37.0 (35.2-38.5) | -36.0 (-38.1 to -35.1) | -2.52 (-2.69 to -2.43) | -25.7 (-27.4 to -24.7) | -4.01 (-4.29 to -3.79) |
| West Karbi Anglong | 66.1 (58.8-73.2) | 52.4 (45.3-59.1) | 43.8 (41.3-46.0) | -34.5 (-35.8 to -33.6) | -2.39 (-2.48 to -2.30) | -17.0 (-18.7 to -15.9) | -2.49 (-2.74 to -2.27) |
| **Andhra Pradesh** | **51.0 (48.4-54.1)** | **42.9 (41.5-44.2)** | **35.1 (33.5-36.7)** | **-32.2 (-32.7 to -31.5)** | **-2.18 (-2.21 to -2.12)** | **-19.1 (-19.8 to -18.3)** | **-2.87 (-3.66 to -2.12)** |
| Anantapur | 59.8 (53.1-66.2) | 46.6 (42.0-50.9) | 41.0 (39.5-42.3) | -32.3 (-33.6 to -31.4) | -2.19 (-2.29 to -2.11) | -12.8 (-14.2 to -11.6) | -1.80 (-2.01 to -1.59) |
| Chittoor | 51.9 (46.9-57.0) | 32.5 (28.5-36.6) | 33.9 (32.8-35.1) | -35.5 (-36.9 to -34.7) | -2.47 (-2.58 to -2.39) | 3.6 (1.2 to 5.1) | 0.62 (0.31 to 0.84) |
| East Godavari | 45.9 (38.7-53.4) | 39.1 (32.3-46.2) | 31.6 (29.9-33.6) | -31.6 (-33.5 to -30.3) | -2.15 (-2.28 to -2.02) | -19.5 (-21.8 to -17.8) | -2.94 (-3.27 to -2.60) |
| Guntur | 50.8 (44.8-57.0) | 47.6 (43.0-53.1) | 31.7 (30.2-33.3) | -38.3 (-39.5 to -37.3) | -2.72 (-2.82 to -2.61) | -34.2 (-35.3 to -33.1) | -5.62 (-5.83 to -5.37) |
| Krishna | 48.7 (42.5-55.5) | 43.1 (37.7-49.6) | 27.9 (25.8-29.9) | -43.5 (-44.8 to -42.4) | -3.22 (-3.33 to -3.09) | -36.1 (-37.5 to -34.8) | -6.02 (-6.29 to -5.73) |
| Kurnool | 59.6 (53.1-66.8) | 53.1 (47.5-58.6) | 45.4 (43.8-47.2) | -24.6 (-26.4 to -23.2) | -1.58 (-1.71 to -1.47) | -15.3 (-16.9 to -13.8) | -2.20 (-2.44 to -1.94) |
| Prakasam | 49.4 (42.6-56.6) | 47.8 (41.7-54.1) | 35.4 (33.5-37.3) | -28.9 (-30.7 to -28.1) | -1.92 (-2.06 to -1.84) | -26.6 (-27.9 to -25.6) | -4.16 (-4.37 to -3.95) |
| Sri Potti Sriramulu Nellore | 50.8 (43.6-58.5) | 40.4 (34.3-46.9) | 37.9 (35.5-40.1) | -25.9 (-27.5 to -24.3) | -1.69 (-1.80 to -1.55) | -6.7 (-9.3 to -5.5) | -0.88 (-1.24 to -0.65) |
| Srikakulam | 51.7 (44.2-58.9) | 39.5 (33.6-45.7) | 33.1 (31.2-35.5) | -36.7 (-38.6 to -35.8) | -2.58 (-2.74 to -2.49) | -16.6 (-19.1 to -15.2) | -2.45 (-2.81 to -2.17) |
| Visakhapatnam | 46.8 (39.4-54.5) | 40.6 (35.3-46.0) | 36.2 (33.8-39.1) | -23.1 (-25.7 to -21.6) | -1.48 (-1.65 to -1.34) | -11.4 (-13.7 to -9.6) | -1.59 (-1.92 to -1.28) |
| Vizianagaram | 49.9 (43.8-57.0) | 41.2 (35.7-46.6) | 36.3 (34.3-38.2) | -28.0 (-30.0 to -26.8) | -1.86 (-2.00 to -1.74) | -12.6 (-14.7 to -11.1) | -1.78 (-2.08 to -1.51) |
| West Godavari | 46.6 (40.3-53.4) | 39.9 (33.9-46.9) | 28.5 (26.4-31.1) | -39.5 (-41.2 to -38.4) | -2.84 (-2.98 to -2.71) | -29.1 (-30.8 to -27.5) | -4.65 (-4.92 to -4.30) |
| YSR | 51.4 (44.9-57.7) | 44.1 (38.7-49.7) | 39.8 (38.0-41.8) | -23.2 (-25.2 to -22.0) | -1.48 (-1.62 to -1.38) | -10.4 (-12.2 to -8.7) | -1.42 (-1.68 to -1.14) |
| **West Bengal** | **50.3 (47.7-53.5)** | **42.1 (40.8-43.9)** | **32.0 (30.6-33.7)** | **-37.4 (-37.8 to -36.8)** | **-2.63 (-2.66 to -2.58)** | **-25.0 (-25.8 to -24.4)** | **-3.92 (-4.81 to -3.07)** |
| Alipurduar | 43.7 (36.8-50.4) | 46.7 (41.8-52.2) | 32.5 (30.3-34.5) | -26.2 (-28.4 to -25.1) | -1.71 (-1.87 to -1.61) | -31.2 (-32.4 to -30.0) | -5.03 (-5.25 to -4.77) |
| Bankura | 52.0 (47.0-57.2) | 47.9 (43.0-52.3) | 36.5 (35.1-37.8) | -30.7 (-31.8 to -29.8) | -2.06 (-2.15 to -1.98) | -24.7 (-26.4 to -23.8) | -3.81 (-4.10 to -3.63) |
| Birbhum | 57.0 (51.7-62.2) | 49.6 (44.6-53.9) | 36.6 (35.3-37.9) | -36.6 (-37.5 to -35.5) | -2.56 (-2.64 to -2.46) | -27.0 (-28.3 to -26.7) | -4.22 (-4.46 to -4.15) |
| Cooch Behar | 46.8 (40.8-52.4) | 48.1 (43.7-52.3) | 35.5 (33.9-36.8) | -24.9 (-26.6 to -23.9) | -1.61 (-1.72 to -1.52) | -27.1 (-28.5 to -26.5) | -4.25 (-4.49 to -4.12) |
| Dakshin Dinajpur | 54.9 (49.6-61.0) | 46.3 (42.0-51.1) | 34.5 (32.3-37.0) | -38.1 (-39.4 to -37.1) | -2.70 (-2.81 to -2.60) | -26.4 (-28.1 to -25.3) | -4.13 (-4.41 to -3.89) |
| Darjeeling | 54.3 (48.2-60.5) | 51.8 (46.4-57.6) | 33.7 (31.6-36.3) | -38.7 (-40.2 to -37.8) | -2.76 (-2.89 to -2.66) | -35.8 (-36.7 to -34.5) | -5.95 (-6.11 to -5.66) |
| Hooghly | 47.4 (42.6-52.5) | 36.2 (32.3-40.5) | 29.2 (27.9-30.7) | -39.2 (-40.1 to -38.4) | -2.80 (-2.88 to -2.72) | -20.0 (-21.9 to -18.8) | -3.00 (-3.29 to -2.77) |
| Howrah | 45.3 (39.0-52.8) | 33.5 (27.9-39.1) | 28.5 (25.5-32.4) | -37.6 (-39.3 to -36.2) | -2.68 (-2.80 to -2.52) | -15.0 (-18.1 to -13.1) | -2.22 (-2.64 to -1.82) |
| Jalpaiguri | 49.2 (43.4-55.1) | 49.8 (45.1-54.9) | 33.2 (31.2-35.0) | -33.4 (-34.6 to -32.5) | -2.29 (-2.38 to -2.20) | -34.3 (-35.5 to -33.3) | -5.64 (-5.86 to -5.42) |
| Jhargram | 53.8 (47.5-59.8) | 54.8 (48.5-60.5) | 38.5 (36.9-40.3) | -29.1 (-30.8 to -28.2) | -1.93 (-2.06 to -1.85) | -30.5 (-32.0 to -29.6) | -4.90 (-5.17 to -4.71) |
| Kalimpong | 46.7 (39.9-53.4) | 45.8 (40.1-52.1) | 27.3 (25.7-29.1) | -42.3 (-44.0 to -41.3) | -3.10 (-3.25 to -2.99) | -41.2 (-42.5 to -40.3) | -7.12 (-7.36 to -6.88) |
| Kolkata | 45.0 (37.7-53.0) | 33.0 (25.7-40.0) | 27.8 (25.6-30.8) | -38.6 (-41.4 to -37.4) | -2.79 (-3.00 to -2.62) | -15.8 (-20.5 to -13.7) | -2.39 (-3.05 to -1.93) |
| Maldah | 56.9 (51.6-61.5) | 50.8 (46.8-55.0) | 35.2 (34.0-36.6) | -39.1 (-40.4 to -38.4) | -2.79 (-2.90 to -2.71) | -31.6 (-32.5 to -30.8) | -5.10 (-5.26 to -4.93) |
| Murshidabad | 53.5 (49.3-58.1) | 43.2 (39.5-46.8) | 33.3 (32.2-34.6) | -38.7 (-39.8 to -37.7) | -2.75 (-2.85 to -2.66) | -23.9 (-25.1 to -22.9) | -3.66 (-3.87 to -3.47) |
| Nadia | 49.7 (45.4-54.8) | 38.1 (34.3-41.6) | 29.8 (28.2-31.5) | -40.9 (-41.8 to -39.8) | -2.96 (-3.04 to -2.84) | -22.6 (-24.0 to -21.4) | -3.44 (-3.66 to -3.21) |
| North 24 Parganas | 47.6 (42.9-52.8) | 35.5 (31.6-39.9) | 28.3 (26.3-30.2) | -41.4 (-42.6 to -40.2) | -3.02 (-3.12 to -2.89) | -21.2 (-23.3 to -20.1) | -3.20 (-3.55 to -2.98) |
| Paschim Burdwan | 50.4 (43.7-57.3) | 46.4 (40.4-52.0) | 34.3 (32.3-36.3) | -32.5 (-34.2 to -31.4) | -2.22 (-2.34 to -2.11) | -26.7 (-28.3 to -25.8) | -4.18 (-4.45 to -3.98) |
| Pashchim Medinipur | 51.1 (46.3-55.7) | 43.8 (38.8-48.2) | 34.2 (33.0-35.2) | -33.9 (-35.0 to -33.1) | -2.33 (-2.41 to -2.25) | -22.7 (-24.4 to -21.7) | -3.46 (-3.74 to -3.25) |
| Purba Burdwan | 53.2 (48.7-58.0) | 43.7 (39.7-47.7) | 33.1 (32.1-34.7) | -38.7 (-39.7 to -37.8) | -2.75 (-2.83 to -2.66) | -25.0 (-26.5 to -24.3) | -3.86 (-4.11 to -3.71) |
| Purba Medinipur | 49.8 (44.5-55.1) | 37.9 (32.7-43.0) | 29.6 (28.1-31.1) | -41.3 (-42.7 to -40.1) | -3.01 (-3.12 to -2.87) | -22.5 (-24.8 to -21.5) | -3.44 (-3.81 to -3.23) |
| Puruliya | 51.0 (43.5-57.6) | 52.9 (47.8-57.3) | 36.0 (35.2-37.0) | -30.1 (-32.2 to -29.2) | -2.02 (-2.17 to -1.93) | -32.8 (-33.8 to -32.0) | -5.34 (-5.52 to -5.17) |
| South 24 Parganas | 49.3 (43.8-54.7) | 37.1 (32.3-42.1) | 29.8 (28.4-31.6) | -40.4 (-41.9 to -39.4) | -2.92 (-3.05 to -2.81) | -20.5 (-22.1 to -19.4) | -3.08 (-3.34 to -2.86) |
| Uttar Dinajpur | 54.7 (49.6-59.4) | 51.5 (47.6-55.6) | 35.7 (34.6-37.0) | -35.6 (-36.7 to -34.7) | -2.48 (-2.56 to -2.39) | -31.5 (-32.3 to -30.6) | -5.08 (-5.22 to -4.89) |
| **Tripura** | **49.3 (44.2-56.4)** | **37.2 (35.6-38.8)** | **30.4 (28.6-32.0)** | **-39.2 (-40.6 to -38.3)** | **-2.81 (-2.93 to -2.71)** | **-19.1 (-20.7 to -17.9)** | **-2.89 (-3.81 to -1.94)** |
| Dhalai | 50.6 (44.3-56.5) | 39.8 (35.2-44.1) | 34.3 (32.4-36.6) | -33.0 (-34.6 to -31.9) | -2.26 (-2.38 to -2.15) | -14.6 (-16.3 to -13.3) | -2.10 (-2.35 to -1.86) |
| Gomati | 47.3 (42.4-52.5) | 35.8 (32.3-39.8) | 27.8 (25.9-30.1) | -42.1 (-43.3 to -41.2) | -3.08 (-3.18 to -2.98) | -23.1 (-24.8 to -21.6) | -3.55 (-3.81 to -3.25) |
| Khowai | 50.1 (44.4-55.3) | 37.5 (33.2-41.9) | 31.1 (28.5-33.7) | -38.8 (-40.2 to -37.7) | -2.77 (-2.88 to -2.65) | -17.9 (-20.2 to -16.4) | -2.65 (-3.00 to -2.36) |
| North Tripura | 51.9 (46.7-57.7) | 41.9 (36.9-46.7) | 38.4 (36.2-40.5) | -26.7 (-28.2 to -25.5) | -1.75 (-1.85 to -1.64) | -8.9 (-11.2 to -7.2) | -1.20 (-1.52 to -0.91) |
| Sepahijala | 48.3 (42.8-53.8) | 34.9 (30.9-39.0) | 26.6 (24.1-29.2) | -45.6 (-47.2 to -44.5) | -3.44 (-3.58 to -3.30) | -24.4 (-26.2 to -22.8) | -3.78 (-4.07 to -3.46) |
| South Tripura | 48.7 (43.2-54.1) | 38.2 (34.2-42.2) | 31.4 (28.3-34.7) | -36.4 (-38.4 to -35.3) | -2.56 (-2.72 to -2.44) | -18.6 (-20.2 to -17.1) | -2.77 (-3.01 to -2.47) |
| Unakoti | 51.6 (44.5-58.6) | 41.4 (35.9-47.4) | 38.9 (36.4-41.6) | -25.3 (-27.7 to -23.5) | -1.65 (-1.81 to -1.49) | -6.7 (-9.5 to -5.5) | -0.89 (-1.26 to -0.65) |
| West Tripura | 48.5 (39.2-57.7) | 34.1 (27.2-41.2) | 25.5 (23.4-28.6) | -47.8 (-50.5 to -46.7) | -3.71 (-3.94 to -3.53) | -25.3 (-29.7 to -22.3) | -4.06 (-4.72 to -3.36) |
| **Arunachal Pradesh** | **46.9 (42.5-54.2)** | **39.7 (38.1-41.4)** | **32.3 (30.8-33.9)** | **-32.0 (-33.5 to -31.1)** | **-2.17 (-2.28 to -2.08)** | **-19.2 (-21.3 to -17.6)** | **-2.98 (-3.87 to -2.02)** |
| Anjaw | 46.8 (36.6-56.6) | 40.8 (31.9-50.0) | 34.0 (31.1-37.1) | -27.5 (-30.5 to -26.3) | -1.83 (-2.03 to -1.70) | -16.6 (-20.6 to -14.4) | -2.50 (-3.06 to -2.03) |
| Changlang | 47.7 (40.5-54.6) | 38.2 (31.1-46.6) | 31.3 (29.4-33.3) | -34.9 (-37.0 to -33.9) | -2.43 (-2.59 to -2.32) | -18.1 (-21.4 to -16.3) | -2.73 (-3.21 to -2.35) |
| Dibang Valley | 51.8 (40.6-62.1) | 51.1 (41.4-60.2) | 45.2 (42.1-48.3) | -12.7 (-16.1 to -11.4) | -0.76 (-0.96 to -0.64) | -11.6 (-14.5 to -10.3) | -1.65 (-2.04 to -1.38) |
| East Kameng | 43.1 (34.4-52.5) | 51.4 (41.8-61.5) | 41.2 (37.8-44.4) | -4.1 (-8.3 to -1.3) | -0.23 (-0.44 to -0.02) | -20.1 (-23.3 to -18.4) | -3.06 (-3.54 to -2.70) |
| East Siang | 44.5 (37.6-51.3) | 34.5 (26.1-43.3) | 25.9 (24.2-27.7) | -42.3 (-44.5 to -40.9) | -3.11 (-3.31 to -2.95) | -24.7 (-28.2 to -22.9) | -3.90 (-4.43 to -3.48) |
| Kamle | 51.8 (39.1-64.2) | 44.2 (32.5-57.3) | 33.6 (31.3-37.6) | -35.0 (-40.2 to -33.1) | -2.51 (-2.88 to -2.25) | -23.4 (-28.6 to -20.8) | -3.79 (-4.50 to -3.10) |
| Kra Daddi | 48.1 (36.5-59.6) | 51.2 (40.3-63.3) | 43.5 (40.3-46.6) | -9.3 (-14.0 to -7.2) | -0.57 (-0.82 to -0.38) | -15.1 (-18.7 to -12.8) | -2.26 (-2.75 to -1.78) |
| Kurung Kumey | 40.0 (31.6-49.7) | 49.4 (39.8-58.8) | 40.6 (38.1-43.6) | 1.9 (-2.3 to 4.0) | 0.12 (-0.08 to 0.29) | -17.9 (-20.6 to -16.7) | -2.69 (-3.07 to -2.41) |
| Lohit | 44.6 (37.3-51.8) | 36.2 (28.4-45.4) | 28.5 (27.0-30.5) | -36.5 (-38.5 to -35.6) | -2.58 (-2.73 to -2.47) | -21.1 (-24.4 to -19.3) | -3.26 (-3.73 to -2.84) |
| Longding | 52.9 (45.4-59.9) | 43.0 (34.8-51.5) | 37.6 (35.1-40.4) | -29.6 (-31.6 to -28.5) | -1.98 (-2.12 to -1.87) | -12.7 (-16.6 to -10.4) | -1.84 (-2.39 to -1.40) |
| Lower Dibang Valley | 42.2 (34.6-50.8) | 33.8 (26.2-42.7) | 25.7 (24.1-27.2) | -39.7 (-41.4 to -38.3) | -2.86 (-3.00 to -2.71) | -23.9 (-26.9 to -22.2) | -3.75 (-4.19 to -3.35) |
| Lower Siang | 50.4 (41.5-59.8) | 39.9 (30.5-50.2) | 31.2 (28.3-34.6) | -38.6 (-41.4 to -37.2) | -2.78 (-3.00 to -2.61) | -21.4 (-25.3 to -19.3) | -3.35 (-3.90 to -2.85) |
| Lower Subansiri | 54.3 (45.1-63.5) | 47.3 (38.0-56.7) | 35.2 (32.9-37.5) | -35.7 (-37.7 to -34.6) | -2.50 (-2.65 to -2.38) | -25.8 (-28.5 to -24.1) | -4.07 (-4.49 to -3.67) |
| Namsai | 45.9 (38.3-54.0) | 36.0 (28.0-45.2) | 27.9 (25.6-30.9) | -39.7 (-42.2 to -38.1) | -2.87 (-3.08 to -2.69) | -22.2 (-26.0 to -19.6) | -3.47 (-4.03 to -2.90) |
| Pakke Kessang | 59.4 (50.4-66.9) | 51.0 (43.0-58.8) | 36.3 (34.4-38.0) | -39.5 (-41.1 to -38.4) | -2.84 (-2.97 to -2.72) | -29.4 (-31.3 to -28.4) | -4.71 (-5.03 to -4.48) |
| Papum Pare | 58.2 (47.5-68.0) | 46.8 (37.5-56.9) | 31.8 (28.8-35.3) | -45.9 (-48.3 to -45.1) | -3.48 (-3.69 to -3.36) | -32.2 (-35.3 to -31.0) | -5.29 (-5.83 to -4.98) |
| Shi Yomi | 46.9 (39.0-54.3) | 38.8 (30.3-47.4) | 29.6 (27.7-31.4) | -37.4 (-39.5 to -36.2) | -2.65 (-2.82 to -2.52) | -23.8 (-27.0 to -22.0) | -3.71 (-4.20 to -3.32) |
| Siang | 42.2 (32.7-51.8) | 39.4 (30.4-49.0) | 31.1 (28.1-34.4) | -26.1 (-29.5 to -23.8) | -1.74 (-1.96 to -1.51) | -20.7 (-24.5 to -19.8) | -3.21 (-3.76 to -2.93) |
| Tawang | 38.0 (29.7-47.4) | 44.9 (36.3-54.1) | 30.6 (27.1-34.0) | -19.4 (-22.9 to -17.6) | -1.24 (-1.44 to -1.06) | -32.3 (-34.8 to -31.3) | -5.31 (-5.73 to -5.02) |
| Tirap | 51.6 (44.5-58.8) | 41.9 (34.3-50.3) | 36.2 (34.0-38.6) | -30.4 (-32.1 to -29.0) | -2.05 (-2.17 to -1.91) | -13.5 (-16.6 to -11.8) | -1.97 (-2.39 to -1.62) |
| Upper Siang | 41.9 (32.5-52.7) | 40.0 (30.6-50.1) | 33.5 (31.3-36.3) | -20.0 (-23.4 to -17.7) | -1.27 (-1.48 to -1.07) | -15.9 (-20.0 to -14.8) | -2.38 (-2.96 to -2.10) |
| Upper Subansiri | 43.4 (34.8-52.6) | 48.5 (38.4-58.0) | 41.6 (38.2-45.1) | -4.1 (-8.0 to -2.2) | -0.23 (-0.43 to -0.07) | -14.3 (-17.6 to -12.6) | -2.09 (-2.56 to -1.75) |
| West Kameng | 47.2 (38.9-57.7) | 49.6 (41.6-58.0) | 35.1 (32.3-38.4) | -25.8 (-28.4 to -24.0) | -1.70 (-1.87 to -1.52) | -29.6 (-32.0 to -27.7) | -4.77 (-5.17 to -4.35) |
| West Siang | 41.2 (29.5-53.9) | 42.5 (31.0-53.4) | 35.8 (33.3-38.4) | -12.2 (-16.4 to -9.9) | -0.77 (-0.98 to -0.55) | -15.2 (-19.9 to -14.2) | -2.31 (-2.95 to -2.00) |
| **Meghalaya** | **54.7 (51.0-60.1)** | **49.0 (47.8-50.5)** | **45.0 (43.3-47.3)** | **-18.6 (-19.7 to -17.8)** | **-1.14 (-1.21 to -1.08)** | **-9.1 (-10.1 to -8.1)** | **-1.22 (-1.97 to -0.44)** |
| East Garo Hills | 49.5 (44.7-54.2) | 45.0 (41.0-49.9) | 40.5 (39.3-41.9) | -19.0 (-20.5 to -17.8) | -1.17 (-1.27 to -1.08) | -10.8 (-12.6 to -9.5) | -1.48 (-1.75 to -1.26) |
| East Jaintia Hills | 61.8 (56.3-67.3) | 54.7 (49.1-60.7) | 52.7 (50.5-54.5) | -15.5 (-16.9 to -14.5) | -0.93 (-1.02 to -0.85) | -4.4 (-5.9 to -3.2) | -0.51 (-0.71 to -0.32) |
| East Khasi Hills | 57.8 (51.5-64.0) | 52.0 (44.6-58.5) | 48.9 (45.8-52.4) | -16.1 (-18.4 to -14.5) | -0.98 (-1.12 to -0.85) | -6.6 (-9.1 to -5.4) | -0.86 (-1.21 to -0.64) |
| North Garo Hills | 46.5 (41.3-51.9) | 42.8 (37.8-48.2) | 38.7 (36.1-41.0) | -17.5 (-19.4 to -16.2) | -1.07 (-1.19 to -0.97) | -10.3 (-12.4 to -9.1) | -1.42 (-1.72 to -1.20) |
| Ri Bhoi | 57.2 (50.5-63.2) | 51.8 (45.5-58.1) | 46.2 (43.4-49.4) | -19.9 (-22.1 to -18.9) | -1.24 (-1.38 to -1.16) | -11.4 (-13.6 to -10.1) | -1.60 (-1.91 to -1.36) |
| South Garo Hills | 53.5 (48.7-58.4) | 46.3 (42.3-50.4) | 37.7 (35.8-39.6) | -30.4 (-31.6 to -29.6) | -2.03 (-2.13 to -1.97) | -19.5 (-20.9 to -18.4) | -2.89 (-3.13 to -2.70) |
| South West Garo Hills | 50.7 (45.1-56.2) | 44.8 (39.6-49.7) | 42.7 (40.1-45.4) | -16.6 (-19.2 to -15.4) | -1.01 (-1.17 to -0.91) | -5.4 (-7.4 to -3.6) | -0.67 (-0.93 to -0.38) |
| South West Khasi Hills | 59.3 (54.1-64.4) | 53.5 (48.4-58.4) | 49.3 (46.3-52.5) | -17.6 (-19.4 to -16.1) | -1.08 (-1.19 to -0.96) | -8.6 (-10.5 to -7.4) | -1.15 (-1.41 to -0.94) |
| West Garo Hills | 46.1 (41.2-51.0) | 40.6 (36.4-45.2) | 35.8 (34.2-37.7) | -22.9 (-24.6 to -21.6) | -1.46 (-1.57 to -1.35) | -12.3 (-14.1 to -11.1) | -1.73 (-1.99 to -1.51) |
| West Jaintia Hills | 60.3 (55.2-65.4) | 53.5 (47.9-59.2) | 50.8 (48.1-53.7) | -16.6 (-18.1 to -15.3) | -1.00 (-1.10 to -0.90) | -5.7 (-7.3 to -4.2) | -0.72 (-0.92 to -0.47) |
| West Khasi Hills | 57.2 (52.2-62.4) | 52.6 (48.0-57.2) | 48.8 (47.4-50.2) | -15.6 (-16.7 to -14.9) | -0.93 (-1.00 to -0.88) | -8.2 (-9.8 to -6.9) | -1.07 (-1.30 to -0.86) |
| **Karnataka** | **50.3 (48.0-52.9)** | **42.8 (41.7-44.0)** | **34.9 (33.5-36.3)** | **-31.6 (-31.9 to -31.1)** | **-2.13 (-2.15 to -2.08)** | **-19.5 (-20.1 to -19.1)** | **-2.94 (-3.65 to -2.27)** |
| Bagalkot | 56.9 (51.8-62.0) | 48.4 (45.5-51.4) | 41.8 (40.2-43.6) | -27.4 (-28.6 to -26.6) | -1.80 (-1.88 to -1.72) | -14.6 (-15.7 to -13.9) | -2.07 (-2.25 to -1.95) |
| Ballari | 57.2 (51.7-61.8) | 54.6 (50.4-58.9) | 45.8 (44.1-47.3) | -20.7 (-22.2 to -19.6) | -1.29 (-1.39 to -1.21) | -16.9 (-18.1 to -15.8) | -2.46 (-2.65 to -2.26) |
| Belagavi | 51.9 (47.8-56.2) | 43.4 (40.3-46.8) | 35.9 (34.6-37.3) | -31.7 (-32.9 to -30.8) | -2.14 (-2.23 to -2.06) | -18.1 (-19.3 to -17.2) | -2.67 (-2.84 to -2.49) |
| Bengaluru Rural | 46.8 (41.7-51.6) | 38.3 (33.8-42.6) | 30.3 (28.9-31.6) | -36.0 (-37.3 to -35.2) | -2.52 (-2.62 to -2.43) | -21.5 (-23.0 to -20.4) | -3.25 (-3.49 to -3.04) |
| Bengaluru Urban | 43.8 (38.4-49.6) | 35.0 (29.6-40.1) | 25.9 (23.8-28.1) | -41.7 (-43.5 to -40.6) | -3.05 (-3.20 to -2.92) | -26.7 (-29.1 to -25.8) | -4.21 (-4.61 to -3.99) |
| Bidar | 49.9 (43.5-55.6) | 52.5 (48.6-56.4) | 41.5 (40.0-42.8) | -17.6 (-19.4 to -16.2) | -1.07 (-1.19 to -0.97) | -21.8 (-22.8 to -20.9) | -3.29 (-3.45 to -3.13) |
| Chamarajanagar | 45.9 (40.8-51.3) | 41.4 (37.5-45.0) | 31.6 (30.6-32.8) | -31.9 (-33.1 to -31.1) | -2.16 (-2.25 to -2.09) | -24.5 (-25.9 to -23.7) | -3.78 (-4.01 to -3.61) |
| Chikballapur | 45.0 (39.7-49.9) | 39.7 (34.9-44.5) | 34.7 (33.2-36.1) | -23.7 (-25.2 to -22.5) | -1.52 (-1.62 to -1.41) | -13.4 (-15.1 to -12.1) | -1.91 (-2.14 to -1.66) |
| Chikkamagaluru | 51.4 (46.0-57.1) | 39.4 (34.5-44.2) | 30.0 (28.9-31.2) | -42.5 (-43.6 to -41.5) | -3.12 (-3.21 to -3.01) | -24.6 (-26.6 to -23.5) | -3.81 (-4.14 to -3.57) |
| Chitradurga | 53.4 (47.6-59.3) | 45.8 (40.6-50.9) | 37.7 (35.9-39.2) | -30.2 (-31.5 to -29.2) | -2.03 (-2.12 to -1.93) | -18.4 (-19.9 to -17.1) | -2.71 (-2.95 to -2.48) |
| Dakshina Kannada | 40.8 (33.4-48.7) | 29.5 (23.6-35.6) | 23.1 (21.1-25.5) | -43.8 (-46.1 to -42.5) | -3.28 (-3.47 to -3.11) | -21.8 (-25.2 to -19.9) | -3.37 (-3.89 to -2.94) |
| Davanagere | 56.5 (50.9-61.4) | 46.8 (42.6-51.2) | 38.7 (37.5-40.0) | -32.3 (-33.6 to -31.5) | -2.20 (-2.30 to -2.12) | -18.2 (-19.5 to -17.2) | -2.67 (-2.89 to -2.49) |
| Dharwad | 57.8 (52.2-63.5) | 47.7 (43.9-51.4) | 40.9 (38.8-43.0) | -30.0 (-31.2 to -29.0) | -2.00 (-2.09 to -1.92) | -15.1 (-16.2 to -13.7) | -2.16 (-2.33 to -1.92) |
| Gadag | 60.7 (55.8-65.6) | 51.9 (49.1-54.7) | 44.7 (43.2-46.5) | -27.2 (-28.2 to -26.2) | -1.78 (-1.85 to -1.69) | -14.7 (-15.7 to -14.0) | -2.10 (-2.24 to -1.96) |
| Hassan | 49.0 (43.9-54.8) | 37.8 (32.5-42.9) | 28.5 (27.0-30.1) | -42.6 (-43.7 to -41.5) | -3.12 (-3.23 to -3.01) | -25.0 (-26.9 to -23.8) | -3.89 (-4.19 to -3.63) |
| Haveri | 57.8 (52.3-63.6) | 46.9 (43.7-50.3) | 39.6 (37.8-41.1) | -32.3 (-33.4 to -31.3) | -2.20 (-2.28 to -2.10) | -16.6 (-17.7 to -15.6) | -2.40 (-2.58 to -2.22) |
| Kalaburgi | 52.2 (46.6-57.6) | 53.7 (49.5-57.9) | 47.6 (46.0-48.8) | -9.6 (-11.3 to -8.6) | -0.54 (-0.64 to -0.46) | -12.2 (-13.4 to -11.6) | -1.70 (-1.87 to -1.58) |
| Kodagu | 42.9 (37.9-48.2) | 34.5 (30.3-38.7) | 28.4 (27.2-29.7) | -34.5 (-36.3 to -33.8) | -2.39 (-2.53 to -2.31) | -18.4 (-19.9 to -17.1) | -2.72 (-2.94 to -2.48) |
| Kolar | 43.4 (38.4-48.0) | 35.8 (30.9-40.2) | 29.9 (28.5-31.0) | -31.9 (-33.3 to -31.0) | -2.16 (-2.27 to -2.07) | -17.2 (-19.1 to -16.2) | -2.52 (-2.81 to -2.33) |
| Koppal | 60.6 (55.0-66.3) | 55.2 (52.0-58.3) | 47.1 (45.6-48.6) | -23.2 (-24.5 to -22.4) | -1.47 (-1.56 to -1.41) | -15.6 (-16.4 to -14.9) | -2.24 (-2.35 to -2.12) |
| Mandya | 49.7 (44.7-54.6) | 40.5 (35.9-44.6) | 28.8 (27.6-30.1) | -42.9 (-44.0 to -42.4) | -3.16 (-3.25 to -3.10) | -29.6 (-31.1 to -28.7) | -4.73 (-4.98 to -4.52) |
| Mysuru | 47.6 (42.0-53.5) | 40.2 (35.8-44.8) | 29.2 (27.4-30.9) | -39.5 (-40.9 to -38.5) | -2.84 (-2.95 to -2.73) | -28.2 (-29.8 to -27.3) | -4.47 (-4.74 to -4.26) |
| Raichur | 54.6 (48.8-60.1) | 53.6 (49.6-57.6) | 46.6 (45.2-48.0) | -15.4 (-17.1 to -14.4) | -0.92 (-1.03 to -0.84) | -13.9 (-15.0 to -13.1) | -1.96 (-2.14 to -1.83) |
| Ramanagara | 49.0 (43.5-54.4) | 40.4 (35.2-45.1) | 29.2 (27.6-30.9) | -41.3 (-42.4 to -40.3) | -3.00 (-3.10 to -2.90) | -28.5 (-30.0 to -27.4) | -4.52 (-4.77 to -4.28) |
| Shivamogga | 52.5 (46.1-59.0) | 40.3 (36.0-45.2) | 32.8 (31.4-34.7) | -38.3 (-39.6 to -37.5) | -2.73 (-2.83 to -2.64) | -19.4 (-20.9 to -18.2) | -2.89 (-3.13 to -2.66) |
| Tumakuru | 50.4 (45.3-55.4) | 41.1 (37.0-45.4) | 33.8 (32.5-34.8) | -33.8 (-34.9 to -32.9) | -2.32 (-2.40 to -2.24) | -18.6 (-20.2 to -17.5) | -2.75 (-3.01 to -2.55) |
| Udupi | 44.7 (36.8-53.0) | 30.7 (25.7-36.1) | 23.9 (22.4-25.6) | -47.1 (-48.6 to -46.0) | -3.60 (-3.73 to -3.46) | -22.4 (-24.6 to -21.0) | -3.44 (-3.78 to -3.13) |
| Uttara Kannada | 49.0 (43.0-54.5) | 38.8 (34.6-43.0) | 35.2 (33.7-37.0) | -28.9 (-30.1 to -27.8) | -1.92 (-2.00 to -1.82) | -10.0 (-11.5 to -8.6) | -1.36 (-1.58 to -1.12) |
| Vijaypura | 50.8 (45.6-56.2) | 45.7 (42.2-49.0) | 40.1 (38.5-42.2) | -21.9 (-23.4 to -21.1) | -1.38 (-1.48 to -1.31) | -13.1 (-14.5 to -12.3) | -1.84 (-2.06 to -1.69) |
| Yadgir | 53.0 (48.1-57.6) | 52.2 (48.4-56.0) | 46.8 (45.8-47.9) | -12.4 (-13.9 to -11.6) | -0.72 (-0.81 to -0.66) | -11.2 (-12.2 to -10.3) | -1.53 (-1.69 to -1.38) |
| **Telangana** | **51.3 (48.0-54.5)** | **40.9 (38.7-43.4)** | **31.7 (29.4-33.9)** | **-39.0 (-39.9 to -38.3)** | **-2.78 (-2.85 to -2.71)** | **-23.3 (-24.1 to -22.7)** | **-3.63 (-4.67 to -2.64)** |
| Adilabad | 48.8 (40.8-57.3) | 44.8 (39.4-49.6) | 37.9 (35.6-40.4) | -22.7 (-25.4 to -21.5) | -1.46 (-1.63 to -1.34) | -16.1 (-17.8 to -14.9) | -2.35 (-2.59 to -2.12) |
| Bhadradri Kothagudem | 45.8 (37.7-55.3) | 38.9 (32.7-45.1) | 29.7 (27.8-31.9) | -35.6 (-37.8 to -34.0) | -2.49 (-2.66 to -2.33) | -24.2 (-26.7 to -22.9) | -3.75 (-4.15 to -3.47) |
| Hyderabad | 47.7 (39.4-55.9) | 35.9 (30.6-41.7) | 25.9 (23.1-29.3) | -46.2 (-48.4 to -45.0) | -3.51 (-3.71 to -3.35) | -28.3 (-30.8 to -26.8) | -4.53 (-4.93 to -4.18) |
| Jagitial | 53.3 (44.7-63.4) | 43.7 (37.9-49.4) | 33.8 (30.8-37.1) | -37.1 (-39.3 to -35.9) | -2.63 (-2.80 to -2.50) | -23.4 (-25.3 to -22.1) | -3.60 (-3.91 to -3.34) |
| Jangoan | 51.2 (43.1-59.8) | 36.1 (31.4-40.7) | 23.1 (21.4-25.1) | -55.6 (-57.0 to -54.8) | -4.57 (-4.71 to -4.44) | -36.8 (-38.9 to -35.7) | -6.18 (-6.58 to -5.91) |
| Jayashankar Bhupalpally | 52.3 (44.0-62.1) | 41.3 (35.1-47.1) | 31.8 (29.1-34.2) | -39.7 (-41.8 to -38.5) | -2.87 (-3.03 to -2.73) | -23.6 (-26.0 to -22.4) | -3.65 (-4.02 to -3.38) |
| Jogulamba Gadwal | 53.8 (47.2-60.9) | 46.1 (41.1-50.6) | 45.3 (43.4-47.3) | -16.6 (-17.8 to -15.1) | -1.01 (-1.07 to -0.89) | -2.4 (-4.2 to -1.4) | -0.23 (-0.47 to -0.06) |
| Kamareddy | 57.6 (48.3-66.3) | 45.6 (41.4-49.7) | 36.7 (34.5-38.9) | -36.9 (-38.8 to -36.1) | -2.61 (-2.76 to -2.51) | -20.4 (-21.7 to -19.3) | -3.06 (-3.26 to -2.85) |
| Karimnagar | 51.3 (42.3-59.5) | 37.5 (31.6-43.5) | 26.0 (23.6-28.9) | -49.8 (-51.8 to -49.0) | -3.90 (-4.08 to -3.77) | -31.2 (-33.3 to -30.0) | -5.06 (-5.42 to -4.78) |
| Khammam | 44.7 (37.7-52.6) | 39.5 (34.0-45.0) | 28.3 (26.2-30.3) | -37.2 (-39.3 to -36.2) | -2.64 (-2.81 to -2.52) | -29.1 (-30.7 to -27.7) | -4.64 (-4.91 to -4.34) |
| Kumuram Bheem Asifabad | 48.6 (39.8-57.4) | 41.8 (36.2-47.5) | 34.9 (32.9-36.9) | -28.6 (-30.7 to -27.2) | -1.91 (-2.05 to -1.77) | -17.2 (-18.8 to -16.2) | -2.53 (-2.75 to -2.33) |
| Mahabubnagar | 55.5 (48.9-62.1) | 45.1 (40.8-49.2) | 41.6 (39.9-43.0) | -25.9 (-27.6 to -24.8) | -1.68 (-1.80 to -1.59) | -8.6 (-10.3 to -7.3) | -1.15 (-1.38 to -0.93) |
| Mahuababad | 48.2 (40.6-56.7) | 38.3 (32.9-44.0) | 26.3 (24.4-28.4) | -46.1 (-48.1 to -44.9) | -3.49 (-3.67 to -3.34) | -32.2 (-34.0 to -30.9) | -5.24 (-5.56 to -4.95) |
| Mancherial | 50.1 (39.9-59.6) | 40.8 (35.0-47.9) | 32.7 (30.1-35.9) | -35.0 (-37.7 to -33.8) | -2.46 (-2.65 to -2.31) | -20.5 (-22.8 to -19.1) | -3.11 (-3.45 to -2.80) |
| Medak | 56.7 (48.7-64.4) | 43.7 (39.9-47.4) | 34.1 (32.2-35.8) | -40.7 (-42.1 to -39.8) | -2.95 (-3.06 to -2.85) | -22.9 (-24.4 to -22.0) | -3.49 (-3.74 to -3.31) |
| Medchal Malkajgiri | 49.9 (41.6-57.7) | 37.4 (32.2-42.4) | 27.1 (24.2-30.4) | -46.3 (-48.2 to -44.8) | -3.51 (-3.69 to -3.34) | -28.0 (-30.2 to -27.3) | -4.47 (-4.81 to -4.26) |
| Nagarkurnool | 52.5 (43.7-61.3) | 46.3 (40.3-51.9) | 40.9 (38.1-43.7) | -22.6 (-25.4 to -21.2) | -1.45 (-1.63 to -1.32) | -12.3 (-14.7 to -11.2) | -1.74 (-2.08 to -1.52) |
| Nalgonda | 49.6 (43.3-56.3) | 42.1 (38.3-46.0) | 31.5 (29.7-33.5) | -37.3 (-38.7 to -36.6) | -2.63 (-2.74 to -2.56) | -26.0 (-27.5 to -25.1) | -4.05 (-4.30 to -3.86) |
| Nirmal | 53.2 (45.4-62.5) | 46.9 (42.5-51.1) | 39.0 (36.9-41.2) | -27.3 (-29.4 to -26.1) | -1.80 (-1.94 to -1.69) | -17.8 (-19.1 to -16.9) | -2.62 (-2.80 to -2.45) |
| Nizamabad | 56.2 (49.1-64.9) | 46.4 (42.5-50.5) | 37.9 (35.8-40.0) | -33.2 (-35.1 to -32.0) | -2.28 (-2.42 to -2.16) | -19.1 (-20.4 to -18.2) | -2.84 (-3.04 to -2.66) |
| Peddapalli | 50.8 (41.5-60.0) | 38.7 (32.3-44.9) | 28.4 (26.3-30.9) | -44.5 (-46.7 to -43.2) | -3.34 (-3.52 to -3.18) | -27.1 (-28.9 to -25.7) | -4.27 (-4.57 to -3.98) |
| Rajanna Sircilla | 54.1 (46.5-61.6) | 41.9 (37.1-46.4) | 30.9 (28.7-33.3) | -43.5 (-45.2 to -42.4) | -3.23 (-3.37 to -3.09) | -26.9 (-28.6 to -25.9) | -4.22 (-4.52 to -4.00) |
| Rangareddy | 52.0 (45.0-59.3) | 39.9 (35.6-44.6) | 30.4 (28.3-32.3) | -42.3 (-43.7 to -41.2) | -3.10 (-3.23 to -2.98) | -24.7 (-26.2 to -23.6) | -3.82 (-4.06 to -3.59) |
| Sangareddy | 56.4 (49.2-63.3) | 45.1 (41.5-48.9) | 38.4 (36.9-39.9) | -32.7 (-34.0 to -31.6) | -2.23 (-2.33 to -2.12) | -15.8 (-17.0 to -14.7) | -2.28 (-2.47 to -2.08) |
| Siddipet | 53.3 (46.3-59.9) | 39.6 (35.6-43.6) | 27.7 (26.4-29.3) | -48.8 (-50.3 to -47.9) | -3.77 (-3.92 to -3.65) | -30.9 (-32.7 to -30.0) | -4.98 (-5.30 to -4.78) |
| Suryapet | 47.5 (40.1-54.6) | 41.8 (37.7-46.1) | 29.9 (27.9-32.1) | -37.6 (-39.4 to -36.3) | -2.67 (-2.81 to -2.53) | -29.1 (-30.6 to -28.0) | -4.64 (-4.89 to -4.40) |
| Vikarabad | 57.5 (50.7-64.7) | 46.3 (42.0-50.4) | 41.7 (40.0-43.6) | -28.2 (-29.8 to -27.1) | -1.86 (-1.98 to -1.76) | -10.7 (-12.1 to -9.8) | -1.46 (-1.66 to -1.31) |
| Wanaparthy | 53.8 (46.8-61.2) | 46.1 (40.6-51.1) | 43.3 (41.1-45.6) | -20.2 (-21.8 to -18.5) | -1.26 (-1.36 to -1.13) | -6.8 (-8.5 to -5.4) | -0.87 (-1.11 to -0.64) |
| Warangal Rural | 49.7 (41.7-58.1) | 37.9 (33.1-43.6) | 25.7 (23.6-27.4) | -49.0 (-50.5 to -47.9) | -3.80 (-3.94 to -3.66) | -32.9 (-34.5 to -31.3) | -5.39 (-5.65 to -5.04) |
| Warangal Urban | 49.3 (39.5-58.5) | 35.7 (29.8-42.5) | 22.9 (20.1-25.5) | -54.0 (-56.1 to -52.8) | -4.40 (-4.60 to -4.20) | -36.2 (-38.6 to -35.0) | -6.09 (-6.51 to -5.77) |
| Yadadri Bhuvanagiri | 50.5 (42.8-58.8) | 36.4 (32.4-41.1) | 23.9 (21.8-26.1) | -53.3 (-54.4 to -52.3) | -4.29 (-4.40 to -4.14) | -35.1 (-36.7 to -34.0) | -5.83 (-6.11 to -5.55) |
| **Gujarat** | **56.5 (53.5-59.8)** | **48.4 (46.5-50.6)** | **39.6 (38.0-41.5)** | **-30.8 (-31.3 to -30.3)** | **-2.06 (-2.10 to -2.02)** | **-19.1 (-19.9 to -18.6)** | **-2.86 (-3.74 to -2.01)** |
| Ahmedabad | 56.9 (51.2-63.0) | 49.0 (45.0-53.5) | 37.4 (34.9-39.9) | -35.2 (-36.6 to -34.1) | -2.45 (-2.55 to -2.34) | -24.6 (-26.2 to -23.3) | -3.80 (-4.06 to -3.54) |
| Amreli | 55.0 (48.5-62.0) | 46.3 (40.0-51.4) | 39.9 (37.1-42.4) | -28.3 (-30.0 to -27.1) | -1.87 (-2.00 to -1.76) | -14.6 (-16.5 to -13.1) | -2.10 (-2.38 to -1.83) |
| Anand | 60.9 (55.3-66.6) | 54.7 (50.1-58.6) | 43.6 (41.0-46.4) | -29.3 (-30.9 to -28.6) | -1.95 (-2.07 to -1.88) | -21.1 (-22.5 to -20.3) | -3.18 (-3.41 to -3.02) |
| Arvalli | 63.2 (57.5-69.5) | 54.6 (50.0-59.0) | 45.8 (42.8-48.6) | -28.4 (-29.7 to -27.6) | -1.87 (-1.97 to -1.80) | -16.9 (-18.0 to -15.6) | -2.46 (-2.62 to -2.22) |
| Banaskantha | 61.9 (55.9-67.8) | 52.3 (48.4-56.1) | 45.2 (43.4-47.1) | -27.7 (-29.0 to -26.6) | -1.82 (-1.91 to -1.72) | -14.4 (-15.5 to -13.5) | -2.05 (-2.22 to -1.88) |
| Bharuch | 55.5 (49.4-61.5) | 51.5 (46.7-55.8) | 43.3 (40.8-46.0) | -22.7 (-24.4 to -21.5) | -1.44 (-1.56 to -1.34) | -16.6 (-18.2 to -15.6) | -2.43 (-2.66 to -2.22) |
| Bhavnagar | 54.2 (47.7-60.2) | 47.3 (41.7-52.5) | 41.2 (38.2-43.8) | -24.8 (-26.3 to -23.7) | -1.60 (-1.70 to -1.51) | -13.6 (-16.1 to -12.4) | -1.94 (-2.31 to -1.71) |
| Botad | 56.0 (48.9-63.0) | 49.6 (44.0-55.0) | 43.3 (40.4-46.7) | -23.4 (-24.9 to -21.7) | -1.50 (-1.59 to -1.36) | -13.5 (-15.4 to -12.2) | -1.91 (-2.19 to -1.68) |
| Chhotaudepur | 57.7 (52.3-63.1) | 50.3 (46.3-54.4) | 46.1 (44.3-47.9) | -20.9 (-22.3 to -20.2) | -1.31 (-1.40 to -1.25) | -9.2 (-10.5 to -8.3) | -1.23 (-1.42 to -1.08) |
| Dahod | 61.1 (55.2-66.8) | 48.8 (44.8-52.5) | 46.5 (44.8-48.5) | -24.7 (-26.1 to -23.6) | -1.59 (-1.69 to -1.50) | -5.6 (-7.1 to -5.2) | -0.69 (-0.89 to -0.60) |
| Dang | 54.4 (47.3-61.0) | 49.2 (44.6-53.7) | 40.6 (38.3-42.9) | -26.2 (-27.6 to -25.0) | -1.71 (-1.80 to -1.60) | -18.3 (-19.8 to -17.6) | -2.71 (-2.93 to -2.56) |
| Devbhumi Dwarka | 52.8 (43.2-61.4) | 41.5 (34.0-49.4) | 29.4 (26.4-32.4) | -45.0 (-47.2 to -43.7) | -3.38 (-3.58 to -3.22) | -29.6 (-32.2 to -28.3) | -4.78 (-5.20 to -4.46) |
| Gandhinagar | 58.1 (52.0-64.7) | 49.2 (44.3-54.4) | 37.4 (34.6-40.8) | -36.4 (-38.0 to -35.4) | -2.55 (-2.68 to -2.45) | -24.8 (-26.4 to -23.7) | -3.84 (-4.11 to -3.61) |
| Gir Somnath | 53.9 (46.0-61.4) | 42.3 (36.4-49.0) | 32.4 (30.2-34.6) | -40.6 (-42.1 to -39.6) | -2.94 (-3.07 to -2.83) | -24.0 (-25.7 to -22.5) | -3.72 (-3.97 to -3.41) |
| Jamnagar | 52.6 (45.4-60.3) | 42.0 (36.2-47.9) | 29.0 (26.6-31.4) | -45.6 (-47.0 to -44.7) | -3.44 (-3.56 to -3.33) | -31.6 (-33.6 to -30.6) | -5.14 (-5.48 to -4.89) |
| Junagadh | 53.8 (46.4-61.8) | 42.2 (35.2-49.2) | 28.7 (25.8-32.0) | -47.3 (-49.2 to -46.3) | -3.62 (-3.80 to -3.49) | -32.6 (-34.8 to -30.9) | -5.34 (-5.71 to -4.95) |
| Kachchh | 56.7 (50.1-63.8) | 48.2 (42.1-54.0) | 44.0 (40.7-47.1) | -23.0 (-25.0 to -21.6) | -1.47 (-1.60 to -1.35) | -9.3 (-11.3 to -8.2) | -1.26 (-1.53 to -1.06) |
| Kheda | 61.7 (56.4-67.4) | 54.8 (50.7-59.2) | 43.2 (40.9-45.5) | -30.8 (-32.2 to -29.8) | -2.07 (-2.18 to -1.98) | -22.0 (-23.2 to -21.1) | -3.33 (-3.53 to -3.16) |
| Mahesana | 59.0 (52.9-65.1) | 49.1 (44.5-53.2) | 38.1 (35.8-40.7) | -36.3 (-37.8 to -35.2) | -2.54 (-2.66 to -2.43) | -23.3 (-24.7 to -22.2) | -3.56 (-3.78 to -3.34) |
| Mahisagar | 63.2 (58.5-68.6) | 52.9 (49.3-56.6) | 46.7 (45.0-48.5) | -27.0 (-28.1 to -26.1) | -1.76 (-1.84 to -1.68) | -12.7 (-13.7 to -11.3) | -1.77 (-1.93 to -1.54) |
| Morbi | 54.7 (47.8-61.8) | 47.5 (42.0-53.2) | 38.7 (35.3-41.8) | -30.0 (-31.7 to -28.9) | -2.02 (-2.13 to -1.90) | -19.3 (-20.9 to -18.2) | -2.89 (-3.12 to -2.66) |
| Narmada | 57.2 (50.5-63.5) | 53.5 (48.8-58.0) | 47.3 (44.6-49.8) | -18.1 (-19.7 to -17.1) | -1.11 (-1.21 to -1.03) | -12.5 (-13.8 to -11.2) | -1.74 (-1.94 to -1.52) |
| Navsari | 48.8 (42.2-55.5) | 40.1 (34.2-45.3) | 32.8 (30.1-35.3) | -33.3 (-35.2 to -32.2) | -2.29 (-2.43 to -2.17) | -18.7 (-21.1 to -17.5) | -2.79 (-3.15 to -2.54) |
| Panchmahal | 61.5 (55.5-67.2) | 54.6 (50.2-59.0) | 46.8 (44.1-49.5) | -24.7 (-26.0 to -23.6) | -1.59 (-1.68 to -1.50) | -15.1 (-16.4 to -13.8) | -2.16 (-2.36 to -1.93) |
| Patan | 58.6 (52.5-65.4) | 50.0 (45.1-54.8) | 40.7 (38.3-43.3) | -31.3 (-32.7 to -30.5) | -2.11 (-2.22 to -2.03) | -19.4 (-21.0 to -18.3) | -2.89 (-3.14 to -2.67) |
| Porbandar | 51.8 (42.4-59.9) | 39.6 (32.6-47.9) | 25.3 (22.7-28.4) | -51.7 (-53.6 to -50.8) | -4.11 (-4.29 to -3.97) | -36.4 (-38.7 to -35.1) | -6.15 (-6.53 to -5.77) |
| Rajkot | 52.1 (46.5-58.4) | 42.9 (37.9-48.4) | 32.5 (30.5-34.3) | -38.4 (-39.5 to -37.4) | -2.74 (-2.82 to -2.62) | -25.0 (-26.9 to -24.0) | -3.88 (-4.19 to -3.67) |
| Sabar Kantha | 63.0 (57.2-68.8) | 54.5 (50.1-58.3) | 45.0 (42.5-47.5) | -29.5 (-30.5 to -28.4) | -1.97 (-2.04 to -1.87) | -18.3 (-19.5 to -17.1) | -2.70 (-2.88 to -2.48) |
| Surat | 50.6 (44.6-57.0) | 43.6 (38.8-48.6) | 36.0 (33.9-38.8) | -29.6 (-31.6 to -28.3) | -1.98 (-2.12 to -1.85) | -18.1 (-19.7 to -16.5) | -2.67 (-2.92 to -2.38) |
| Surendranagar | 58.0 (51.7-64.5) | 51.1 (46.2-55.4) | 43.0 (40.5-45.5) | -26.6 (-28.2 to -25.6) | -1.74 (-1.85 to -1.65) | -16.6 (-17.9 to -15.5) | -2.42 (-2.61 to -2.21) |
| Tapi | 52.7 (46.8-58.7) | 47.2 (43.1-51.2) | 39.6 (37.9-41.3) | -25.6 (-27.2 to -25.0) | -1.66 (-1.77 to -1.60) | -17.0 (-18.4 to -16.1) | -2.48 (-2.69 to -2.31) |
| Vadodara | 57.5 (51.7-62.9) | 53.4 (49.0-57.6) | 44.1 (41.8-47.2) | -24.2 (-25.8 to -23.2) | -1.55 (-1.66 to -1.46) | -18.3 (-19.8 to -17.2) | -2.70 (-2.93 to -2.50) |
| Valsad | 50.0 (43.3-56.7) | 40.4 (34.8-46.4) | 35.1 (32.2-38.1) | -30.5 (-32.5 to -29.2) | -2.06 (-2.20 to -1.93) | -13.8 (-16.4 to -12.4) | -1.98 (-2.35 to -1.72) |
| **Manipur** | **45.5 (41.9-51.0)** | **37.9 (36.6-39.2)** | **32.2 (30.6-33.9)** | **-29.9 (-31.1 to -28.9)** | **-2.00 (-2.09 to -1.90)** | **-15.4 (-17.8 to -14.3)** | **-2.29 (-3.13 to -1.50)** |
| Bishnupur | 44.9 (38.8-52.0) | 38.2 (31.7-44.8) | 32.6 (29.4-36.3) | -27.9 (-30.3 to -26.4) | -1.85 (-2.02 to -1.71) | -14.9 (-18.4 to -13.3) | -2.18 (-2.69 to -1.86) |
| Chandel | 48.6 (42.7-54.4) | 44.6 (39.1-50.3) | 41.7 (39.7-44.7) | -14.9 (-16.8 to -13.8) | -0.89 (-1.01 to -0.80) | -7.0 (-9.2 to -5.9) | -0.92 (-1.21 to -0.72) |
| Churachandpur | 49.4 (44.0-55.3) | 42.9 (37.0-48.3) | 38.6 (36.8-40.3) | -22.6 (-24.0 to -21.9) | -1.43 (-1.53 to -1.37) | -10.7 (-12.8 to -9.8) | -1.48 (-1.78 to -1.31) |
| Imphal East | 43.3 (36.9-49.8) | 35.3 (28.7-41.9) | 28.8 (26.8-31.1) | -34.1 (-36.5 to -32.6) | -2.36 (-2.55 to -2.21) | -18.8 (-22.2 to -17.8) | -2.83 (-3.35 to -2.59) |
| Imphal West | 42.1 (33.5-52.1) | 34.2 (26.4-42.5) | 27.3 (25.0-31.0) | -35.1 (-38.9 to -33.4) | -2.49 (-2.76 to -2.27) | -19.7 (-24.1 to -17.3) | -3.08 (-3.69 to -2.51) |
| Jiribam | 51.7 (42.6-60.8) | 40.6 (32.9-48.3) | 35.8 (33.0-39.1) | -31.1 (-33.6 to -30.2) | -2.13 (-2.30 to -2.01) | -11.7 (-15.3 to -10.2) | -1.71 (-2.18 to -1.36) |
| Kakching | 44.8 (38.1-52.2) | 39.1 (32.4-45.7) | 33.5 (30.5-37.0) | -25.8 (-28.3 to -24.1) | -1.69 (-1.86 to -1.53) | -14.8 (-18.0 to -13.5) | -2.17 (-2.63 to -1.90) |
| Kamjong | 43.7 (37.6-50.2) | 38.0 (31.9-44.3) | 35.2 (32.6-37.6) | -19.9 (-22.7 to -18.6) | -1.25 (-1.43 to -1.13) | -7.8 (-11.1 to -6.2) | -1.05 (-1.51 to -0.75) |
| Kangpokpi | 44.3 (37.6-51.1) | 36.4 (28.9-43.9) | 30.2 (27.9-33.1) | -32.4 (-34.1 to -31.4) | -2.22 (-2.34 to -2.11) | -17.3 (-21.0 to -15.5) | -2.59 (-3.13 to -2.21) |
| Noney | 51.6 (45.1-57.1) | 42.3 (36.4-48.2) | 37.0 (35.2-38.7) | -29.1 (-30.5 to -28.3) | -1.93 (-2.03 to -1.86) | -13.2 (-15.2 to -11.5) | -1.87 (-2.17 to -1.57) |
| Pherzawl | 53.2 (47.4-59.1) | 43.1 (37.4-48.4) | 38.8 (36.6-40.9) | -27.8 (-29.5 to -26.6) | -1.84 (-1.96 to -1.73) | -10.5 (-12.8 to -9.0) | -1.45 (-1.78 to -1.19) |
| Senapati | 48.2 (42.9-54.1) | 38.5 (32.2-44.9) | 33.0 (31.9-34.1) | -32.3 (-34.0 to -31.4) | -2.20 (-2.33 to -2.11) | -14.8 (-17.1 to -13.3) | -2.15 (-2.48 to -1.86) |
| Tamenglong | 53.8 (47.8-60.2) | 42.2 (36.2-48.7) | 36.4 (35.1-37.8) | -33.1 (-34.3 to -32.1) | -2.26 (-2.35 to -2.17) | -14.3 (-16.6 to -12.7) | -2.06 (-2.39 to -1.76) |
| Tengnoupal | 44.5 (37.6-52.1) | 39.0 (32.4-45.8) | 34.9 (32.1-37.6) | -22.1 (-24.9 to -21.0) | -1.41 (-1.59 to -1.30) | -10.9 (-13.6 to -9.0) | -1.54 (-1.91 to -1.18) |
| Thoubal | 42.6 (35.7-49.8) | 36.1 (29.8-42.6) | 29.5 (27.4-32.1) | -31.2 (-33.4 to -29.9) | -2.12 (-2.28 to -1.98) | -18.4 (-21.3 to -16.9) | -2.77 (-3.19 to -2.45) |
| Ukhrul | 45.5 (39.2-52.4) | 38.3 (31.5-45.2) | 35.1 (33.3-37.0) | -23.4 (-25.3 to -22.6) | -1.50 (-1.63 to -1.42) | -8.6 (-11.6 to -7.2) | -1.18 (-1.58 to -0.92) |
| **Jammu and Kashmir**† | **46.5 (43.4-51.3)** | **36.0 (34.6-37.5)** | **30.2 (28.7-31.9)** | **-35.8 (-36.8 to -34.8)** | **-2.49 (-2.57 to -2.40)** | **-16.8 (-18.3 to -15.5)** | **-2.51 (-3.37 to -1.67)** |
| Anantnag | 46.0 (40.1-52.3) | 34.1 (27.9-41.0) | 28.9 (27.2-30.2) | -37.9 (-39.4 to -36.5) | -2.69 (-2.81 to -2.55) | -15.6 (-18.2 to -14.1) | -2.29 (-2.66 to -1.98) |
| Badgam | 43.9 (37.5-50.5) | 31.4 (25.3-38.7) | 25.0 (23.2-27.0) | -43.7 (-45.7 to -42.6) | -3.25 (-3.43 to -3.11) | -20.5 (-23.8 to -18.8) | -3.13 (-3.63 to -2.76) |
| Bandipore | 45.7 (38.2-52.5) | 32.8 (26.5-39.1) | 29.6 (27.4-31.8) | -35.8 (-38.0 to -34.6) | -2.51 (-2.68 to -2.38) | -10.0 (-13.1 to -8.5) | -1.41 (-1.82 to -1.11) |
| Baramulla | 47.1 (39.8-54.7) | 34.3 (27.7-40.9) | 29.4 (27.8-31.4) | -38.1 (-40.0 to -37.3) | -2.71 (-2.87 to -2.61) | -14.5 (-16.9 to -12.7) | -2.11 (-2.44 to -1.76) |
| Doda | 49.4 (43.4-56.5) | 42.2 (35.3-49.5) | 35.4 (33.7-37.6) | -28.9 (-30.3 to -27.6) | -1.93 (-2.02 to -1.80) | -16.5 (-18.9 to -15.4) | -2.43 (-2.78 to -2.19) |
| Ganderbal | 45.1 (38.9-51.4) | 31.9 (25.6-37.5) | 28.0 (26.2-30.1) | -38.5 (-40.0 to -37.3) | -2.75 (-2.86 to -2.62) | -12.3 (-15.1 to -10.8) | -1.77 (-2.15 to -1.47) |
| Jammu | 44.4 (38.2-51.6) | 35.8 (31.3-41.1) | 27.6 (25.6-29.9) | -38.4 (-40.3 to -37.4) | -2.75 (-2.90 to -2.63) | -23.5 (-25.6 to -22.1) | -3.63 (-3.96 to -3.34) |
| Kargil | 45.3 (37.8-53.4) | 37.6 (30.7-44.8) | 35.1 (32.2-37.9) | -22.9 (-25.7 to -21.1) | -1.47 (-1.65 to -1.31) | -6.8 (-9.0 to -4.8) | -0.91 (-1.19 to -0.55) |
| Kathua | 49.2 (43.7-55.3) | 40.2 (34.7-45.7) | 32.4 (30.7-34.4) | -34.8 (-36.4 to -33.9) | -2.42 (-2.54 to -2.32) | -19.9 (-21.7 to -18.6) | -2.98 (-3.27 to -2.73) |
| Kishtwar | 49.3 (42.0-56.2) | 41.8 (35.2-49.7) | 37.3 (35.3-39.5) | -25.0 (-26.9 to -23.7) | -1.62 (-1.75 to -1.50) | -11.2 (-13.9 to -9.4) | -1.58 (-1.95 to -1.25) |
| Kulgam | 45.1 (38.9-51.8) | 34.3 (27.8-41.3) | 27.2 (25.8-28.8) | -40.4 (-41.9 to -39.3) | -2.93 (-3.05 to -2.80) | -20.9 (-24.1 to -18.8) | -3.20 (-3.68 to -2.76) |
| Kupwara | 49.3 (40.7-58.4) | 36.2 (28.5-43.2) | 33.8 (31.4-36.1) | -31.9 (-34.5 to -30.7) | -2.18 (-2.37 to -2.05) | -6.8 (-9.7 to -4.8) | -0.93 (-1.29 to -0.55) |
| Leh | 40.7 (29.3-51.6) | 33.5 (25.5-42.5) | 26.9 (24.0-29.8) | -33.4 (-37.3 to -31.6) | -2.35 (-2.61 to -2.13) | -19.5 (-23.5 to -17.0) | -2.99 (-3.57 to -2.46) |
| PoJK | 50.1 (39.9-59.9) | 42.2 (37.3-47.3) | 41.1 (34.5-48.4) | -18.1 (-21.2 to -16.8) | -1.15 (-1.33 to -1.02) | -3.4 (-6.0 to -1.4) | -0.42 (-0.75 to -0.45) |
| Poonch | 47.5 (39.9-56.1) | 39.1 (33.1-44.9) | 33.1 (31.0-35.3) | -30.9 (-33.1 to -29.7) | -2.09 (-2.25 to -1.97) | -15.9 (-18.0 to -14.2) | -2.32 (-2.62 to -2.01) |
| Pulwama | 45.0 (38.4-51.5) | 31.7 (25.1-38.0) | 25.3 (23.3-27.7) | -44.3 (-46.1 to -43.2) | -3.31 (-3.47 to -3.18) | -20.2 (-23.7 to -18.6) | -3.10 (-3.61 to -2.72) |
| Rajouri | 48.3 (40.5-56.5) | 41.3 (35.7-47.4) | 34.5 (32.2-37.0) | -29.0 (-31.3 to -27.3) | -1.94 (-2.10 to -1.78) | -17.0 (-19.0 to -15.6) | -2.50 (-2.80 to -2.23) |
| Ramban | 48.7 (42.2-55.6) | 41.0 (33.4-48.3) | 34.0 (31.9-36.5) | -30.9 (-32.6 to -29.7) | -2.09 (-2.21 to -1.97) | -17.4 (-20.1 to -16.4) | -2.59 (-2.98 to -2.36) |
| Reasi | 48.3 (42.8-54.9) | 41.3 (35.3-47.3) | 33.5 (31.9-35.4) | -31.5 (-32.9 to -30.3) | -2.13 (-2.23 to -2.02) | -19.6 (-21.5 to -18.4) | -2.94 (-3.23 to -2.70) |
| Samba | 45.2 (39.1-52.7) | 36.1 (30.9-41.5) | 27.7 (25.4-30.5) | -39.3 (-41.2 to -38.2) | -2.83 (-2.98 to -2.70) | -23.8 (-25.9 to -22.1) | -3.68 (-4.00 to -3.33) |
| Shopian | 44.1 (37.8-50.7) | 33.6 (26.3-40.5) | 26.0 (23.5-28.9) | -41.7 (-43.6 to -40.5) | -3.06 (-3.22 to -2.91) | -22.5 (-26.6 to -20.6) | -3.51 (-4.14 to -3.07) |
| Srinagar | 43.0 (35.7-50.9) | 29.1 (21.8-36.9) | 23.7 (21.4-26.8) | -45.4 (-48.0 to -44.1) | -3.45 (-3.66 to -3.26) | -18.2 (-22.8 to -16.0) | -2.81 (-3.46 to -2.29) |
| Udhampur | 49.1 (43.8-55.0) | 41.2 (35.3-46.8) | 33.8 (32.3-35.4) | -31.9 (-33.4 to -31.0) | -2.17 (-2.28 to -2.07) | -18.5 (-20.5 to -17.1) | -2.75 (-3.05 to -2.47) |
| **Haryana** | **56.6 (52.5-62.0)** | **44.7 (43.3-46.0)** | **35.2 (33.6-36.7)** | **-38.8 (-39.3 to -38.3)** | **-2.76 (-2.80 to -2.71)** | **-22.2 (-23.2 to -21.5)** | **-3.42 (-4.17 to -2.61)** |
| Ambala | 51.4 (46.0-56.9) | 41.6 (37.1-46.2) | 31.6 (30.1-32.8) | -39.4 (-40.3 to -38.5) | -2.82 (-2.89 to -2.73) | -24.7 (-26.4 to -23.5) | -3.82 (-4.09 to -3.58) |
| Bhiwani | 56.8 (51.4-62.2) | 45.9 (41.0-50.5) | 35.0 (33.9-36.1) | -39.2 (-40.4 to -38.5) | -2.80 (-2.90 to -2.73) | -24.5 (-25.9 to -23.4) | -3.77 (-4.00 to -3.56) |
| Charkhi Dadri | 56.8 (51.6-62.1) | 46.1 (41.8-50.7) | 36.0 (34.5-37.8) | -37.5 (-38.6 to -36.7) | -2.65 (-2.73 to -2.57) | -22.7 (-24.2 to -21.5) | -3.45 (-3.70 to -3.22) |
| Faridabad | 57.4 (49.8-64.7) | 46.0 (39.9-51.6) | 33.4 (30.4-37.0) | -42.5 (-43.9 to -41.1) | -3.13 (-3.25 to -2.97) | -28.1 (-29.9 to -27.0) | -4.47 (-4.76 to -4.20) |
| Fatehabad | 55.7 (50.5-60.9) | 43.6 (38.7-48.6) | 35.3 (33.9-36.6) | -37.4 (-38.6 to -36.6) | -2.63 (-2.73 to -2.55) | -19.7 (-21.2 to -18.2) | -2.94 (-3.17 to -2.67) |
| Gurugram | 57.0 (51.1-63.4) | 45.4 (39.9-50.2) | 34.1 (31.4-37.6) | -41.0 (-42.6 to -39.9) | -2.98 (-3.11 to -2.86) | -25.6 (-27.6 to -24.6) | -4.00 (-4.33 to -3.77) |
| Hisar | 57.6 (51.1-63.9) | 45.4 (40.0-51.0) | 35.2 (33.6-37.1) | -39.6 (-41.0 to -38.8) | -2.84 (-2.96 to -2.75) | -23.1 (-24.8 to -22.1) | -3.54 (-3.81 to -3.32) |
| Jhajjar | 58.2 (52.9-63.2) | 45.9 (41.8-50.3) | 37.2 (35.8-38.9) | -36.9 (-38.1 to -36.1) | -2.59 (-2.69 to -2.51) | -19.8 (-21.0 to -18.5) | -2.95 (-3.13 to -2.71) |
| Jind | 58.5 (53.9-63.5) | 45.2 (40.6-49.8) | 37.5 (36.4-38.5) | -36.9 (-37.8 to -36.2) | -2.59 (-2.66 to -2.52) | -17.9 (-19.5 to -16.8) | -2.63 (-2.88 to -2.43) |
| Kaithal | 54.4 (49.6-58.7) | 42.2 (37.8-47.1) | 35.4 (34.1-36.8) | -35.8 (-37.0 to -35.0) | -2.49 (-2.59 to -2.41) | -16.8 (-18.6 to -15.6) | -2.46 (-2.73 to -2.23) |
| Karnal | 56.7 (51.6-62.1) | 43.3 (38.5-48.6) | 35.1 (33.5-36.4) | -39.0 (-40.0 to -38.1) | -2.78 (-2.87 to -2.69) | -19.6 (-21.1 to -18.3) | -2.93 (-3.15 to -2.67) |
| Kurukshetra | 53.2 (48.0-58.3) | 41.8 (37.3-46.8) | 32.1 (31.1-33.4) | -40.7 (-41.7 to -39.8) | -2.94 (-3.03 to -2.85) | -24.1 (-25.9 to -23.2) | -3.70 (-4.02 to -3.52) |
| Mahendragarh | 54.1 (49.0-59.3) | 44.7 (40.2-49.1) | 33.8 (32.3-35.4) | -38.5 (-39.6 to -37.6) | -2.74 (-2.83 to -2.65) | -25.2 (-26.6 to -24.4) | -3.91 (-4.14 to -3.74) |
| Nuh | 58.8 (53.9-63.5) | 48.3 (43.9-52.5) | 38.7 (37.1-39.9) | -35.1 (-36.0 to -34.2) | -2.43 (-2.50 to -2.35) | -20.8 (-22.0 to -20.0) | -3.12 (-3.31 to -2.96) |
| Palwal | 58.5 (52.5-64.3) | 48.2 (43.4-53.2) | 37.0 (34.7-39.1) | -37.7 (-38.9 to -36.7) | -2.67 (-2.76 to -2.56) | -24.1 (-25.9 to -22.9) | -3.72 (-4.00 to -3.47) |
| Panchkula | 48.0 (40.8-55.0) | 39.1 (32.8-45.8) | 30.4 (28.2-33.4) | -37.3 (-39.4 to -35.9) | -2.65 (-2.81 to -2.50) | -22.6 (-25.6 to -20.8) | -3.50 (-3.95 to -3.09) |
| Panipat | 58.6 (51.5-66.0) | 44.4 (37.9-51.0) | 36.5 (33.1-39.9) | -38.4 (-39.6 to -37.4) | -2.74 (-2.83 to -2.63) | -18.3 (-20.8 to -16.7) | -2.74 (-3.10 to -2.41) |
| Rewari | 56.2 (49.6-63.3) | 45.5 (40.6-51.2) | 36.7 (34.1-39.5) | -35.5 (-37.2 to -34.5) | -2.48 (-2.61 to -2.37) | -20.0 (-22.1 to -18.8) | -3.01 (-3.33 to -2.75) |
| Rohtak | 58.2 (53.3-63.5) | 45.0 (39.7-50.5) | 37.1 (34.7-39.8) | -37.2 (-38.5 to -36.4) | -2.62 (-2.72 to -2.53) | -18.4 (-20.4 to -17.1) | -2.73 (-3.04 to -2.48) |
| Sirsa | 55.5 (48.6-62.5) | 42.0 (36.7-48.0) | 35.0 (33.3-36.5) | -37.6 (-39.0 to -36.6) | -2.66 (-2.77 to -2.56) | -17.2 (-19.2 to -15.9) | -2.53 (-2.83 to -2.28) |
| Sonipat | 59.6 (54.3-64.5) | 45.9 (41.0-50.7) | 36.7 (35.1-38.5) | -39.3 (-40.3 to -38.4) | -2.81 (-2.89 to -2.72) | -20.8 (-22.6 to -19.9) | -3.14 (-3.42 to -2.95) |
| Yamunanagar | 57.0 (51.5-62.7) | 45.1 (40.2-50.2) | 34.5 (32.9-36.5) | -40.3 (-41.3 to -39.5) | -2.91 (-2.99 to -2.82) | -24.3 (-25.9 to -23.1) | -3.75 (-4.00 to -3.51) |
| **Uttarakhand** | **58.4 (55.1-62.5)** | **42.4 (41.1-43.9)** | **32.4 (30.9-33.7)** | **-45.5 (-46.2 to -45.1)** | **-3.41 (-3.48 to -3.36)** | **-24.6 (-25.5 to -23.9)** | **-3.87 (-4.67 to -3.08)** |
| Almora | 56.3 (49.8-62.7) | 39.5 (35.6-44.1) | 29.7 (28.9-30.8) | -48.0 (-49.1 to -47.3) | -3.68 (-3.78 to -3.59) | -25.5 (-26.9 to -24.2) | -3.97 (-4.20 to -3.70) |
| Bageshwar | 60.9 (52.5-69.6) | 40.6 (34.0-48.2) | 27.5 (25.2-29.9) | -55.7 (-57.0 to -54.9) | -4.57 (-4.71 to -4.45) | -32.8 (-34.8 to -31.2) | -5.39 (-5.72 to -5.00) |
| Chamoli | 62.5 (53.2-71.4) | 43.2 (36.0-50.9) | 32.2 (29.5-35.6) | -49.1 (-50.8 to -48.3) | -3.81 (-3.97 to -3.70) | -25.8 (-27.8 to -24.2) | -4.05 (-4.37 to -3.71) |
| Champawat | 59.4 (53.6-65.1) | 38.7 (34.3-43.7) | 27.5 (26.0-29.3) | -54.6 (-55.6 to -53.9) | -4.42 (-4.53 to -4.33) | -29.7 (-31.4 to -28.5) | -4.75 (-5.04 to -4.49) |
| Dehradun | 55.5 (48.5-62.3) | 40.9 (35.5-46.0) | 29.6 (27.6-31.9) | -47.4 (-48.6 to -46.8) | -3.62 (-3.73 to -3.54) | -28.2 (-30.2 to -26.8) | -4.48 (-4.81 to -4.17) |
| Haridwar | 59.9 (54.1-66.3) | 44.7 (39.8-49.3) | 33.5 (32.4-34.6) | -45.0 (-45.9 to -44.4) | -3.36 (-3.44 to -3.29) | -25.8 (-27.0 to -24.9) | -4.02 (-4.22 to -3.82) |
| Nainital | 56.0 (49.6-62.8) | 41.3 (37.0-45.7) | 32.6 (31.3-33.9) | -42.7 (-44.0 to -42.1) | -3.13 (-3.25 to -3.07) | -21.9 (-23.4 to -20.9) | -3.32 (-3.55 to -3.12) |
| Pauri Garhwal | 58.2 (50.6-65.1) | 43.3 (37.5-47.9) | 33.1 (31.7-34.4) | -43.9 (-45.2 to -42.9) | -3.26 (-3.37 to -3.14) | -24.3 (-26.2 to -23.1) | -3.75 (-4.06 to -3.51) |
| Pithoragarh | 67.7 (60.6-75.4) | 42.4 (35.6-49.3) | 30.1 (27.1-33.3) | -56.4 (-57.6 to -55.3) | -4.66 (-4.80 to -4.51) | -29.5 (-31.5 to -27.7) | -4.74 (-5.07 to -4.34) |
| Rudraprayag | 62.4 (53.1-71.1) | 46.0 (39.1-52.8) | 34.6 (32.1-36.9) | -45.3 (-46.7 to -44.3) | -3.40 (-3.53 to -3.28) | -25.4 (-27.5 to -24.3) | -3.97 (-4.31 to -3.71) |
| Tehri Garhwal | 59.1 (50.7-66.0) | 43.6 (38.1-48.8) | 33.5 (32.0-34.6) | -44.1 (-45.4 to -43.2) | -3.28 (-3.39 to -3.18) | -23.9 (-25.9 to -22.9) | -3.67 (-4.02 to -3.47) |
| Udham Singh Nagar | 57.0 (51.2-62.7) | 42.2 (38.3-46.2) | 35.4 (34.4-36.4) | -38.7 (-39.7 to -38.1) | -2.76 (-2.84 to -2.69) | -17.0 (-18.3 to -16.1) | -2.48 (-2.68 to -2.32) |
| Uttarkashi | 59.4 (50.2-67.9) | 43.9 (37.6-49.8) | 34.4 (32.7-36.1) | -42.7 (-44.3 to -41.9) | -3.14 (-3.28 to -3.04) | -22.2 (-24.4 to -20.9) | -3.38 (-3.73 to -3.11) |
| **Tamil Nadu** | **38.8 (37.2-40.3)** | **32.4 (31.3-33.4)** | **25.7 (24.5-27.0)** | **-34.7 (-35.3 to -34.2)** | **-2.39 (-2.44 to -2.34)** | **-21.6 (-22.5 to -20.9)** | **-3.31 (-4.12 to -2.42)** |
| Ariyalur | 40.9 (35.1-46.8) | 32.7 (27.8-38.6) | 28.3 (26.8-29.8) | -31.5 (-33.3 to -30.4) | -2.14 (-2.27 to -2.03) | -13.9 (-16.3 to -12.7) | -2.01 (-2.35 to -1.76) |
| Chennai | 39.1 (30.2-48.8) | 24.1 (18.0-30.3) | 24.9 (22.0-27.9) | -36.4 (-39.4 to -34.5) | -2.60 (-2.81 to -2.37) | 4.5 (-1.0 to 8.6) | 0.58 (0.00 to 1.31) |
| Coimbatore | 39.4 (33.8-44.9) | 41.5 (35.8-47.2) | 27.3 (25.5-28.9) | -31.4 (-33.0 to -30.1) | -2.13 (-2.24 to -2.00) | -34.9 (-36.4 to -33.9) | -5.78 (-6.05 to -5.53) |
| Cuddalore | 41.1 (35.3-46.7) | 28.0 (24.0-33.5) | 24.9 (23.8-25.9) | -40.2 (-42.0 to -38.9) | -2.90 (-3.05 to -2.76) | -11.5 (-14.0 to -10.3) | -1.62 (-1.98 to -1.38) |
| Dharmapuri | 39.8 (35.0-44.8) | 38.8 (33.6-44.6) | 25.9 (24.6-27.3) | -35.8 (-37.3 to -34.7) | -2.50 (-2.62 to -2.39) | -33.9 (-35.4 to -32.6) | -5.58 (-5.84 to -5.29) |
| Dindigul | 38.8 (33.6-44.5) | 37.6 (32.1-43.2) | 26.4 (25.1-27.7) | -32.7 (-34.6 to -31.5) | -2.24 (-2.38 to -2.12) | -30.4 (-32.2 to -29.1) | -4.90 (-5.21 to -4.60) |
| Erode | 39.8 (35.0-44.7) | 42.8 (37.8-48.3) | 28.3 (27.1-29.4) | -29.6 (-31.4 to -28.2) | -1.98 (-2.11 to -1.85) | -34.7 (-36.0 to -33.4) | -5.73 (-5.97 to -5.44) |
| Kanchipuram | 41.2 (35.1-47.9) | 24.5 (20.7-29.3) | 25.0 (23.3-26.9) | -40.0 (-41.6 to -38.6) | -2.89 (-3.01 to -2.74) | 1.9 (-1.2 to 4.3) | 0.34 (-0.03 to 0.74) |
| Kanniyakumari | 34.3 (26.6-43.0) | 27.5 (21.4-34.4) | 21.2 (18.3-24.3) | -38.2 (-41.7 to -36.9) | -2.77 (-3.03 to -2.59) | -23.0 (-26.9 to -21.0) | -3.62 (-4.20 to -3.14) |
| Karur | 37.8 (32.7-42.5) | 38.0 (32.9-43.5) | 27.0 (26.2-27.9) | -29.3 (-30.9 to -27.9) | -1.95 (-2.07 to -1.83) | -29.6 (-31.0 to -28.2) | -4.73 (-4.97 to -4.44) |
| Krishnagiri | 42.1 (37.8-46.5) | 39.3 (34.7-43.8) | 25.3 (24.5-26.1) | -40.7 (-41.6 to -39.7) | -2.94 (-3.02 to -2.84) | -36.4 (-37.9 to -35.3) | -6.08 (-6.37 to -5.82) |
| Madurai | 36.2 (31.5-41.3) | 33.3 (28.8-38.5) | 24.8 (23.5-25.9) | -32.1 (-34.1 to -30.7) | -2.19 (-2.34 to -2.05) | -26.1 (-28.0 to -24.9) | -4.09 (-4.40 to -3.83) |
| Nagapattinam | 38.6 (32.0-46.3) | 25.1 (20.5-30.2) | 23.2 (21.8-24.6) | -40.5 (-42.6 to -39.1) | -2.95 (-3.11 to -2.78) | -7.6 (-10.9 to -5.4) | -1.05 (-1.48 to -0.64) |
| Namakkal | 36.9 (32.0-42.0) | 38.3 (32.9-43.9) | 26.1 (25.1-27.3) | -29.9 (-31.3 to -28.6) | -2.00 (-2.10 to -1.88) | -32.4 (-34.0 to -31.2) | -5.28 (-5.56 to -5.01) |
| Perambalur | 39.2 (32.8-46.1) | 34.2 (29.3-39.9) | 27.4 (25.9-28.9) | -30.6 (-32.6 to -29.4) | -2.07 (-2.21 to -1.94) | -20.5 (-22.8 to -18.9) | -3.09 (-3.44 to -2.78) |
| Pudukkottai | 39.0 (32.9-45.4) | 33.4 (28.3-38.7) | 26.9 (25.4-28.5) | -31.7 (-33.8 to -30.3) | -2.16 (-2.31 to -2.02) | -20.0 (-22.0 to -18.5) | -3.02 (-3.31 to -2.72) |
| Ramanathapuram | 34.8 (28.9-40.5) | 28.6 (23.9-33.7) | 22.4 (21.1-23.8) | -36.3 (-38.5 to -35.2) | -2.55 (-2.73 to -2.43) | -22.3 (-24.9 to -21.0) | -3.43 (-3.82 to -3.14) |
| Salem | 38.2 (33.8-42.8) | 37.5 (32.8-42.8) | 25.3 (24.3-26.4) | -34.5 (-35.9 to -33.5) | -2.39 (-2.49 to -2.28) | -33.1 (-34.1 to -31.9) | -5.41 (-5.59 to -5.14) |
| Sivaganga | 37.1 (31.4-42.3) | 31.9 (27.5-36.8) | 24.8 (23.5-26.3) | -33.8 (-35.5 to -32.5) | -2.33 (-2.46 to -2.20) | -22.9 (-25.1 to -21.6) | -3.52 (-3.85 to -3.25) |
| Thanjavur | 38.7 (33.2-44.4) | 29.8 (25.1-34.5) | 25.9 (24.4-27.2) | -33.8 (-35.8 to -32.4) | -2.33 (-2.49 to -2.19) | -13.7 (-16.1 to -11.9) | -1.97 (-2.31 to -1.64) |
| The Nilgiris | 42.1 (35.9-48.4) | 43.7 (38.5-49.0) | 29.5 (28.0-31.4) | -30.5 (-32.9 to -29.3) | -2.06 (-2.23 to -1.93) | -33.3 (-34.8 to -32.1) | -5.45 (-5.71 to -5.19) |
| Theni | 36.8 (32.7-41.5) | 35.2 (30.7-40.2) | 24.4 (23.4-25.8) | -34.5 (-36.0 to -33.3) | -2.38 (-2.50 to -2.27) | -31.3 (-32.9 to -30.2) | -5.07 (-5.33 to -4.81) |
| Thiruvallur | 40.1 (33.8-46.9) | 27.2 (22.5-32.5) | 27.2 (25.2-29.7) | -32.8 (-35.4 to -31.3) | -2.26 (-2.45 to -2.10) | -0.4 (-3.9 to 1.8) | 0.01 (-0.42 to 0.40) |
| Thiruvarur | 38.0 (31.7-44.2) | 26.4 (21.7-31.0) | 24.0 (23.0-25.2) | -37.3 (-39.3 to -36.0) | -2.65 (-2.80 to -2.50) | -9.2 (-12.2 to -7.3) | -1.28 (-1.68 to -0.93) |
| Thoothukudi | 34.6 (29.0-40.6) | 31.2 (26.1-36.6) | 25.0 (23.4-26.4) | -28.3 (-30.4 to -26.8) | -1.89 (-2.02 to -1.74) | -20.4 (-22.6 to -18.6) | -3.10 (-3.42 to -2.72) |
| Tiruchirappalli | 38.1 (33.1-43.9) | 35.3 (31.1-40.8) | 26.8 (25.6-28.3) | -30.2 (-31.5 to -28.8) | -2.03 (-2.12 to -1.90) | -24.7 (-26.3 to -23.7) | -3.83 (-4.07 to -3.60) |
| Tirunelveli | 36.7 (31.5-42.3) | 33.2 (28.6-38.2) | 26.6 (25.0-28.3) | -28.0 (-29.9 to -26.6) | -1.86 (-1.99 to -1.72) | -20.2 (-22.4 to -18.3) | -3.05 (-3.38 to -2.67) |
| Tiruppur | 39.0 (34.6-43.7) | 40.7 (35.4-46.1) | 27.3 (26.3-28.5) | -30.8 (-32.3 to -30.0) | -2.08 (-2.18 to -1.99) | -33.7 (-35.0 to -32.3) | -5.53 (-5.77 to -5.23) |
| Tiruvannamalai | 40.4 (35.8-45.5) | 29.0 (25.1-33.1) | 25.7 (24.7-27.1) | -37.3 (-38.7 to -36.3) | -2.63 (-2.74 to -2.53) | -12.0 (-14.1 to -10.2) | -1.69 (-1.99 to -1.38) |
| Vellore | 39.7 (34.7-44.8) | 29.7 (25.8-34.1) | 26.2 (24.9-27.6) | -34.8 (-36.2 to -33.5) | -2.41 (-2.52 to -2.29) | -12.3 (-14.7 to -11.2) | -1.75 (-2.08 to -1.53) |
| Viluppuram | 41.2 (36.5-45.8) | 28.7 (24.7-33.3) | 24.9 (24.2-25.6) | -40.4 (-41.5 to -39.5) | -2.92 (-3.01 to -2.82) | -13.6 (-15.8 to -12.3) | -1.95 (-2.26 to -1.69) |
| Virudhunagar | 35.6 (30.4-40.6) | 32.5 (28.1-37.5) | 25.4 (24.1-27.0) | -29.3 (-31.3 to -28.1) | -1.96 (-2.10 to -1.84) | -22.5 (-24.4 to -20.8) | -3.44 (-3.73 to -3.10) |
| **Mizoram** | **45.9 (41.6-52.5)** | **36.3 (34.9-38.0)** | **29.7 (28.4-31.1)** | **-36.2 (-37.0 to -35.6)** | **-2.53 (-2.59 to -2.47)** | **-19.1 (-20.2 to -18.2)** | **-2.88 (-3.79 to -2.04)** |
| Aizawl | 45.9 (41.5-50.3) | 35.9 (32.2-39.7) | 29.3 (28.7-30.1) | -36.9 (-38.0 to -36.1) | -2.59 (-2.68 to -2.51) | -18.9 (-20.1 to -17.8) | -2.81 (-2.98 to -2.60) |
| Champhai | 46.2 (41.0-51.5) | 37.8 (33.4-42.0) | 29.5 (28.5-30.7) | -36.9 (-38.3 to -35.9) | -2.59 (-2.71 to -2.49) | -22.7 (-24.0 to -21.7) | -3.46 (-3.67 to -3.27) |
| Kolasib | 45.8 (40.7-50.9) | 34.7 (30.8-38.9) | 29.2 (28.2-30.4) | -37.0 (-38.0 to -36.0) | -2.60 (-2.68 to -2.50) | -16.4 (-18.0 to -15.1) | -2.39 (-2.63 to -2.15) |
| Lawangtlai | 47.6 (41.8-54.3) | 38.5 (34.6-42.3) | 32.3 (31.0-33.6) | -32.8 (-34.7 to -31.5) | -2.24 (-2.39 to -2.12) | -16.9 (-18.7 to -15.9) | -2.47 (-2.74 to -2.27) |
| Lunglei | 45.2 (39.6-50.7) | 36.4 (33.0-39.9) | 29.2 (28.7-30.0) | -36.0 (-37.4 to -35.0) | -2.52 (-2.62 to -2.42) | -20.5 (-21.8 to -19.6) | -3.07 (-3.28 to -2.89) |
| Mamit | 44.5 (39.2-50.2) | 33.7 (30.2-37.4) | 28.2 (27.2-29.1) | -37.4 (-38.7 to -36.3) | -2.64 (-2.75 to -2.53) | -16.9 (-18.7 to -15.7) | -2.47 (-2.74 to -2.25) |
| Saiha | 48.6 (41.8-56.4) | 40.7 (35.6-45.6) | 34.5 (32.7-36.2) | -29.7 (-31.5 to -28.8) | -1.99 (-2.12 to -1.90) | -16.1 (-18.0 to -14.9) | -2.34 (-2.63 to -2.11) |
| Serchhip | 43.8 (38.4-49.8) | 35.1 (30.8-39.1) | 27.1 (25.5-28.9) | -38.8 (-40.4 to -37.6) | -2.77 (-2.91 to -2.64) | -23.4 (-25.2 to -22.3) | -3.59 (-3.88 to -3.37) |
| **Maharashtra** | **57.4 (53.3-61.6)** | **41.6 (40.3-43.1)** | **33.1 (31.6-34.5)** | **-43.3 (-43.8 to -42.9)** | **-3.19 (-3.23 to -3.15)** | **-21.4 (-22.0 to -20.8)** | **-3.26 (-4.03 to -2.50)** |
| Ahmednagar | 54.8 (48.8-61.1) | 42.7 (38.8-46.7) | 34.6 (33.3-36.0) | -37.6 (-38.8 to -36.5) | -2.65 (-2.76 to -2.55) | -19.6 (-21.0 to -19.1) | -2.92 (-3.14 to -2.81) |
| Akola | 58.8 (50.8-67.4) | 50.4 (45.1-55.2) | 35.9 (34.4-37.1) | -39.7 (-41.0 to -38.8) | -2.85 (-2.96 to -2.75) | -29.6 (-31.0 to -28.7) | -4.71 (-4.96 to -4.53) |
| Amravati | 60.5 (52.4-68.1) | 45.1 (41.0-49.3) | 32.3 (31.2-33.4) | -47.5 (-48.6 to -46.7) | -3.62 (-3.73 to -3.53) | -29.2 (-30.4 to -28.4) | -4.65 (-4.85 to -4.47) |
| Aurangabad | 56.9 (50.1-63.9) | 47.9 (43.5-52.9) | 36.4 (35.3-37.6) | -36.8 (-38.2 to -35.8) | -2.58 (-2.70 to -2.49) | -24.9 (-26.3 to -24.0) | -3.85 (-4.08 to -3.67) |
| Beed | 57.2 (50.6-63.6) | 47.8 (43.6-51.7) | 36.1 (35.0-37.3) | -37.7 (-39.1 to -36.9) | -2.66 (-2.79 to -2.58) | -25.3 (-26.4 to -24.7) | -3.91 (-4.10 to -3.78) |
| Bhandara | 63.4 (54.6-72.1) | 38.6 (35.0-42.4) | 32.4 (31.2-33.9) | -49.6 (-50.9 to -48.8) | -3.86 (-3.98 to -3.75) | -16.8 (-18.3 to -15.9) | -2.46 (-2.67 to -2.28) |
| Buldhana | 57.7 (50.6-65.0) | 52.1 (47.4-56.9) | 36.9 (35.8-38.1) | -36.8 (-38.1 to -35.9) | -2.58 (-2.69 to -2.49) | -30.0 (-31.0 to -29.0) | -4.78 (-4.96 to -4.59) |
| Chandrapur | 56.9 (48.5-66.7) | 42.3 (37.5-46.6) | 34.9 (33.3-36.5) | -39.3 (-40.7 to -37.9) | -2.82 (-2.93 to -2.67) | -18.3 (-20.0 to -17.0) | -2.70 (-2.97 to -2.45) |
| Dhule | 64.3 (56.7-71.5) | 49.4 (45.0-53.9) | 38.0 (36.4-39.6) | -41.8 (-43.0 to -40.8) | -3.05 (-3.15 to -2.95) | -23.9 (-25.5 to -22.7) | -3.67 (-3.94 to -3.44) |
| Gadchiroli | 62.5 (53.0-71.4) | 42.9 (38.3-47.6) | 36.5 (35.5-37.5) | -42.3 (-44.0 to -41.2) | -3.10 (-3.25 to -2.98) | -15.7 (-17.6 to -14.7) | -2.27 (-2.55 to -2.08) |
| Gondia | 66.6 (57.6-75.5) | 38.1 (34.4-41.9) | 32.4 (31.0-33.8) | -52.1 (-53.2 to -51.5) | -4.13 (-4.25 to -4.05) | -15.6 (-17.0 to -14.2) | -2.25 (-2.46 to -2.01) |
| Hingoli | 58.7 (48.4-68.0) | 50.9 (46.7-55.5) | 37.8 (36.2-39.5) | -36.2 (-38.0 to -35.2) | -2.54 (-2.68 to -2.43) | -26.7 (-27.8 to -26.0) | -4.17 (-4.36 to -4.03) |
| Jalgaon | 60.1 (53.1-67.5) | 48.7 (43.9-54.2) | 36.4 (35.0-37.8) | -40.3 (-41.7 to -39.8) | -2.91 (-3.03 to -2.85) | -26.2 (-27.3 to -25.2) | -4.09 (-4.26 to -3.89) |
| Jalna | 58.7 (51.1-66.8) | 53.2 (48.2-58.8) | 39.5 (38.1-40.8) | -33.5 (-34.9 to -32.6) | -2.30 (-2.40 to -2.21) | -26.7 (-28.0 to -25.9) | -4.17 (-4.39 to -4.00) |
| Kolhapur | 58.4 (50.0-66.2) | 35.5 (31.6-39.9) | 30.0 (28.3-31.6) | -49.5 (-50.7 to -48.7) | -3.84 (-3.97 to -3.74) | -16.2 (-18.1 to -14.9) | -2.37 (-2.64 to -2.11) |
| Latur | 59.1 (50.8-67.4) | 44.7 (40.3-48.7) | 35.4 (34.0-36.8) | -40.8 (-42.4 to -39.8) | -2.96 (-3.09 to -2.85) | -21.6 (-23.2 to -21.0) | -3.26 (-3.52 to -3.13) |
| Mumbai City | 50.9 (37.6-66.7) | 30.2 (21.7-39.5) | 26.7 (23.3-30.0) | -47.3 (-51.3 to -46.3) | -3.72 (-4.03 to -3.49) | -10.3 (-17.5 to -9.2) | -1.67 (-2.54 to -1.22) |
| Mumbai Suburban | 53.2 (42.8-65.2) | 31.6 (24.8-38.7) | 27.5 (24.2-30.7) | -48.7 (-51.3 to -47.7) | -3.79 (-4.03 to -3.63) | -13.1 (-17.0 to -11.6) | -1.93 (-2.46 to -1.59) |
| Nagpur | 60.5 (52.1-69.5) | 40.2 (36.0-43.9) | 31.6 (30.1-33.7) | -48.5 (-49.7 to -47.7) | -3.73 (-3.85 to -3.64) | -22.1 (-23.7 to -20.7) | -3.35 (-3.60 to -3.09) |
| Nanded | 61.1 (52.5-69.6) | 47.3 (43.5-51.8) | 37.1 (36.0-38.3) | -40.0 (-41.6 to -39.1) | -2.88 (-3.01 to -2.78) | -22.5 (-23.5 to -21.8) | -3.42 (-3.58 to -3.28) |
| Nandurbar | 67.2 (60.3-74.2) | 48.8 (45.0-52.8) | 39.5 (38.6-40.6) | -42.1 (-43.0 to -41.5) | -3.07 (-3.15 to -3.01) | -20.0 (-21.3 to -19.1) | -2.98 (-3.19 to -2.81) |
| Nashik | 63.0 (56.0-69.7) | 48.4 (44.1-52.6) | 38.1 (36.8-39.5) | -40.3 (-41.4 to -39.8) | -2.91 (-3.00 to -2.84) | -22.2 (-23.6 to -21.3) | -3.36 (-3.60 to -3.19) |
| Osmanabad | 56.9 (50.2-64.3) | 43.0 (39.4-47.0) | 34.6 (33.6-35.6) | -39.9 (-41.0 to -39.0) | -2.87 (-2.96 to -2.77) | -20.3 (-21.4 to -19.3) | -3.04 (-3.21 to -2.84) |
| Palghar | 61.6 (54.0-69.6) | 39.3 (34.3-44.3) | 32.9 (31.4-34.7) | -47.3 (-48.5 to -46.4) | -3.60 (-3.72 to -3.50) | -16.8 (-18.7 to -15.4) | -2.46 (-2.74 to -2.20) |
| Parbhani | 59.8 (51.2-67.6) | 51.8 (47.6-56.0) | 38.1 (36.9-40.0) | -36.9 (-38.5 to -36.0) | -2.60 (-2.73 to -2.50) | -27.2 (-28.2 to -26.4) | -4.26 (-4.44 to -4.11) |
| Pune | 50.7 (44.8-56.9) | 37.4 (33.3-41.5) | 29.1 (27.6-30.6) | -43.4 (-44.6 to -42.4) | -3.21 (-3.31 to -3.09) | -23.0 (-24.6 to -21.9) | -3.52 (-3.77 to -3.29) |
| Raigad | 52.7 (44.9-61.0) | 33.9 (29.7-38.6) | 29.5 (27.7-31.3) | -44.8 (-46.2 to -43.9) | -3.36 (-3.47 to -3.25) | -13.6 (-15.6 to -12.1) | -1.95 (-2.23 to -1.66) |
| Ratnagiri | 52.6 (43.4-62.7) | 33.6 (28.6-39.1) | 29.0 (27.0-30.8) | -45.5 (-47.1 to -44.2) | -3.43 (-3.57 to -3.27) | -14.4 (-16.7 to -13.2) | -2.07 (-2.41 to -1.85) |
| Sangli | 58.1 (50.4-65.3) | 36.4 (32.3-40.5) | 29.3 (28.0-30.7) | -50.4 (-51.6 to -49.6) | -3.94 (-4.06 to -3.84) | -20.3 (-21.9 to -19.1) | -3.04 (-3.30 to -2.81) |
| Satara | 50.6 (43.2-57.6) | 35.5 (31.2-40.0) | 26.3 (24.9-27.8) | -48.7 (-50.0 to -47.9) | -3.76 (-3.88 to -3.65) | -26.5 (-28.5 to -25.4) | -4.15 (-4.49 to -3.92) |
| Sindhudurg | 56.0 (46.7-65.4) | 33.7 (28.7-38.9) | 32.0 (30.1-33.9) | -43.3 (-45.3 to -42.1) | -3.22 (-3.38 to -3.07) | -5.5 (-7.9 to -3.7) | -0.71 (-1.01 to -0.39) |
| Solapur | 55.9 (49.0-63.4) | 39.8 (35.8-43.8) | 33.1 (31.9-34.3) | -41.6 (-43.1 to -40.7) | -3.03 (-3.16 to -2.93) | -17.6 (-18.7 to -16.6) | -2.57 (-2.75 to -2.39) |
| Thane | 55.6 (47.4-64.6) | 34.9 (29.7-40.6) | 30.4 (27.9-32.6) | -45.9 (-47.7 to -44.8) | -3.48 (-3.63 to -3.34) | -13.3 (-15.4 to -11.6) | -1.91 (-2.19 to -1.58) |
| Wardha | 57.9 (50.0-66.5) | 42.0 (37.8-46.4) | 32.5 (31.4-33.8) | -44.5 (-45.9 to -43.8) | -3.32 (-3.45 to -3.23) | -23.2 (-24.6 to -22.1) | -3.55 (-3.77 to -3.33) |
| Washim | 58.3 (48.5-67.7) | 51.4 (46.5-56.2) | 37.1 (35.5-38.6) | -37.0 (-38.8 to -36.1) | -2.61 (-2.75 to -2.51) | -28.7 (-29.9 to -27.7) | -4.55 (-4.76 to -4.33) |
| Yavatmal | 57.5 (49.5-65.3) | 46.3 (41.4-50.8) | 36.2 (35.1-37.6) | -37.7 (-39.3 to -36.7) | -2.67 (-2.80 to -2.57) | -22.6 (-23.9 to -21.7) | -3.44 (-3.65 to -3.26) |
| **Punjab** | **46.7 (44.3-49.8)** | **38.3 (37.1-39.5)** | **30.2 (28.8-31.7)** | **-36.2 (-37.1 to -35.7)** | **-2.53 (-2.60 to -2.47)** | **-21.9 (-22.9 to -21.0)** | **-3.37 (-4.13 to -2.60)** |
| Amritsar | 47.6 (41.3-54.1) | 36.6 (32.4-41.4) | 30.0 (27.6-32.7) | -37.6 (-39.5 to -36.3) | -2.67 (-2.82 to -2.53) | -18.6 (-20.6 to -17.0) | -2.77 (-3.07 to -2.47) |
| Barnala | 47.4 (41.8-52.9) | 40.5 (35.3-45.3) | 32.9 (31.5-34.7) | -31.4 (-33.0 to -29.8) | -2.12 (-2.24 to -1.98) | -19.6 (-21.6 to -18.4) | -2.92 (-3.25 to -2.69) |
| Bathinda | 48.8 (43.3-54.4) | 40.5 (36.1-45.4) | 32.9 (31.4-34.3) | -33.5 (-34.9 to -32.3) | -2.30 (-2.40 to -2.19) | -19.7 (-21.5 to -18.0) | -2.94 (-3.22 to -2.63) |
| Faridkot | 48.4 (42.1-55.3) | 37.1 (32.5-41.9) | 31.6 (29.9-33.8) | -35.3 (-37.1 to -34.3) | -2.46 (-2.60 to -2.36) | -15.6 (-17.6 to -14.6) | -2.26 (-2.56 to -2.06) |
| Fatehgarh Sahib | 43.5 (38.4-47.9) | 38.5 (34.1-43.0) | 27.1 (25.6-28.5) | -38.4 (-39.8 to -37.5) | -2.73 (-2.84 to -2.64) | -30.3 (-31.9 to -29.2) | -4.87 (-5.14 to -4.62) |
| Fazilka | 50.6 (43.5-57.4) | 40.1 (35.4-44.8) | 33.7 (31.6-35.8) | -34.2 (-35.9 to -32.8) | -2.37 (-2.49 to -2.23) | -16.8 (-18.8 to -15.5) | -2.46 (-2.77 to -2.21) |
| Ferozepur | 50.4 (44.1-56.4) | 38.4 (33.7-43.1) | 33.1 (31.2-35.1) | -35.0 (-36.4 to -33.7) | -2.43 (-2.54 to -2.31) | -14.5 (-16.4 to -13.3) | -2.09 (-2.36 to -1.85) |
| Gurdaspur | 45.2 (40.5-50.2) | 35.5 (31.1-39.8) | 27.8 (26.6-29.2) | -39.4 (-40.7 to -38.6) | -2.82 (-2.94 to -2.73) | -22.4 (-24.3 to -21.0) | -3.41 (-3.72 to -3.14) |
| Hoshiarpur | 46.9 (42.0-51.4) | 38.0 (33.6-43.1) | 29.4 (28.2-30.5) | -38.2 (-39.4 to -37.5) | -2.71 (-2.81 to -2.63) | -23.4 (-25.0 to -22.2) | -3.59 (-3.85 to -3.34) |
| Jalandhar | 46.3 (41.2-51.5) | 36.3 (32.1-41.0) | 29.4 (27.9-30.9) | -37.4 (-38.6 to -36.3) | -2.64 (-2.74 to -2.53) | -19.6 (-22.0 to -18.4) | -2.94 (-3.32 to -2.70) |
| Kapurthala | 47.4 (42.2-51.8) | 36.8 (32.4-41.5) | 30.0 (28.7-31.6) | -37.5 (-39.0 to -36.5) | -2.65 (-2.78 to -2.55) | -19.2 (-21.2 to -18.0) | -2.86 (-3.18 to -2.62) |
| Ludhiana | 44.8 (39.8-49.6) | 37.3 (33.3-41.5) | 29.2 (27.7-30.6) | -35.6 (-36.9 to -34.5) | -2.48 (-2.58 to -2.37) | -22.4 (-24.1 to -21.1) | -3.41 (-3.68 to -3.16) |
| Mansa | 48.3 (43.0-53.7) | 42.9 (37.9-48.1) | 32.8 (31.7-34.1) | -32.8 (-34.2 to -31.9) | -2.24 (-2.34 to -2.15) | -24.2 (-26.2 to -23.1) | -3.74 (-4.06 to -3.50) |
| Moga | 47.6 (41.7-53.6) | 37.0 (32.4-41.8) | 31.3 (29.8-32.7) | -35.0 (-36.7 to -33.6) | -2.43 (-2.56 to -2.30) | -15.9 (-17.9 to -14.5) | -2.31 (-2.62 to -2.06) |
| Pathankot | 46.0 (40.3-52.3) | 36.9 (31.2-42.7) | 27.9 (25.8-30.3) | -39.9 (-41.4 to -38.5) | -2.88 (-2.99 to -2.72) | -25.0 (-27.4 to -23.5) | -3.89 (-4.28 to -3.58) |
| Patiala | 43.6 (38.5-48.8) | 39.5 (34.6-44.9) | 27.7 (26.4-29.2) | -37.2 (-38.6 to -36.7) | -2.63 (-2.74 to -2.56) | -30.5 (-32.1 to -29.3) | -4.91 (-5.17 to -4.64) |
| Rupnagar | 43.5 (38.8-48.1) | 38.4 (33.5-43.1) | 27.1 (25.8-28.5) | -38.4 (-39.9 to -37.5) | -2.73 (-2.85 to -2.64) | -30.0 (-31.6 to -29.2) | -4.82 (-5.09 to -4.62) |
| Sahibzada Ajit Singh Nagar | 42.4 (37.4-47.5) | 38.4 (34.0-42.8) | 26.6 (25.1-28.2) | -38.0 (-39.5 to -36.8) | -2.70 (-2.82 to -2.58) | -31.4 (-32.8 to -30.2) | -5.08 (-5.33 to -4.81) |
| Sangrur | 46.9 (41.3-52.5) | 42.1 (36.9-47.6) | 32.6 (31.3-33.9) | -31.2 (-32.8 to -30.2) | -2.11 (-2.23 to -2.01) | -23.1 (-24.9 to -22.0) | -3.54 (-3.83 to -3.32) |
| Shahid Bhagat Singh Nagar | 45.9 (40.8-51.2) | 39.4 (34.6-44.5) | 29.7 (28.1-31.5) | -36.1 (-37.4 to -34.9) | -2.53 (-2.62 to -2.41) | -25.4 (-26.9 to -24.3) | -3.96 (-4.19 to -3.71) |
| Sri Mukhtar Sahib | 50.2 (42.6-57.3) | 39.3 (34.0-44.1) | 32.7 (31.2-34.6) | -35.6 (-37.3 to -34.8) | -2.49 (-2.62 to -2.40) | -17.4 (-19.5 to -16.1) | -2.56 (-2.89 to -2.31) |
| Tarn Taran | 50.1 (44.7-55.5) | 38.7 (34.5-42.9) | 32.9 (31.5-34.5) | -35.0 (-36.7 to -33.9) | -2.43 (-2.56 to -2.32) | -15.6 (-17.7 to -14.6) | -2.26 (-2.58 to -2.06) |
| **Sikkim** | **45.8 (39.5-55.2)** | **35.6 (33.9-37.6)** | **28.5 (27.0-30.0)** | **-38.5 (-40.2 to -37.6)** | **-2.74 (-2.88 to -2.65)** | **-20.4 (-22.1 to -19.3)** | **-3.15 (-4.21 to -2.23)** |
| East Sikkim | 39.8 (32.5-46.4) | 32.2 (27.3-37.6) | 24.9 (23.1-27.2) | -37.9 (-40.0 to -36.6) | -2.70 (-2.87 to -2.55) | -22.9 (-25.6 to -21.7) | -3.54 (-3.95 to -3.26) |
| North Sikkim | 47.0 (40.1-53.9) | 36.7 (30.5-42.5) | 30.1 (27.2-33.2) | -36.7 (-39.2 to -35.8) | -2.59 (-2.79 to -2.48) | -18.7 (-21.3 to -17.2) | -2.81 (-3.19 to -2.49) |
| South Sikkim | 47.7 (42.0-53.5) | 36.9 (32.3-41.9) | 29.5 (27.4-31.6) | -39.0 (-40.5 to -37.9) | -2.79 (-2.91 to -2.67) | -20.8 (-23.1 to -19.5) | -3.14 (-3.51 to -2.88) |
| West Sikkim | 53.3 (46.8-59.0) | 39.5 (34.2-45.4) | 32.9 (30.1-35.6) | -39.1 (-40.6 to -38.4) | -2.80 (-2.93 to -2.72) | -17.3 (-19.5 to -16.1) | -2.56 (-2.88 to -2.31) |
| **Nagaland** | **44.3 (40.0-51.8)** | **37.9 (36.3-39.5)** | **31.4 (30.1-33.0)** | **-29.8 (-30.9 to -29.1)** | **-1.99 (-2.07 to -1.92)** | **-17.5 (-20.0 to -16.1)** | **-2.68 (-3.52 to -1.88)** |
| Dimapur | 44.7 (37.6-52.2) | 36.5 (28.6-44.5) | 26.4 (24.3-28.7) | -41.5 (-43.6 to -40.3) | -3.04 (-3.21 to -2.89) | -27.7 (-30.2 to -26.2) | -4.44 (-4.81 to -4.06) |
| Kiphire | 42.5 (37.2-47.9) | 39.4 (31.8-46.7) | 36.9 (34.9-39.3) | -13.8 (-16.0 to -12.4) | -0.82 (-0.95 to -0.71) | -6.7 (-9.9 to -6.0) | -0.89 (-1.32 to -0.72) |
| Kohima | 43.0 (37.7-48.5) | 37.4 (30.4-44.6) | 29.1 (27.9-30.4) | -33.1 (-34.7 to -32.1) | -2.26 (-2.39 to -2.17) | -22.4 (-25.3 to -20.9) | -3.45 (-3.90 to -3.13) |
| Longleng | 44.2 (37.2-50.5) | 37.1 (29.3-44.9) | 32.7 (30.4-35.2) | -26.7 (-28.9 to -25.3) | -1.76 (-1.90 to -1.63) | -11.7 (-15.6 to -9.5) | -1.70 (-2.23 to -1.27) |
| Mokokchung | 44.4 (38.2-50.2) | 36.9 (28.0-44.9) | 30.6 (29.0-32.2) | -31.8 (-33.6 to -30.7) | -2.16 (-2.29 to -2.05) | -17.1 (-20.6 to -15.4) | -2.56 (-3.06 to -2.20) |
| Mon | 43.7 (38.3-48.7) | 37.0 (30.3-44.3) | 34.7 (33.5-36.2) | -21.3 (-23.0 to -20.3) | -1.34 (-1.45 to -1.25) | -6.5 (-9.3 to -4.7) | -0.87 (-1.22 to -0.54) |
| Peren | 47.7 (41.2-53.4) | 40.6 (33.6-47.9) | 31.5 (30.2-33.1) | -34.7 (-35.9 to -34.1) | -2.40 (-2.49 to -2.34) | -22.8 (-25.1 to -21.5) | -3.50 (-3.86 to -3.23) |
| Phek | 42.1 (36.8-48.3) | 39.2 (31.7-46.4) | 33.9 (32.1-35.7) | -20.1 (-21.8 to -18.3) | -1.26 (-1.36 to -1.11) | -13.6 (-16.7 to -11.7) | -1.97 (-2.41 to -1.61) |
| Tuensang | 44.3 (39.1-49.9) | 39.6 (32.1-47.1) | 37.0 (35.1-38.8) | -17.3 (-19.4 to -16.1) | -1.06 (-1.19 to -0.96) | -6.8 (-10.4 to -5.2) | -0.91 (-1.40 to -0.60) |
| Wokha | 45.0 (40.0-50.1) | 37.3 (30.2-44.3) | 28.7 (27.6-29.9) | -37.0 (-38.3 to -36.4) | -2.61 (-2.71 to -2.54) | -23.3 (-26.0 to -21.6) | -3.60 (-4.02 to -3.25) |
| Zunheboto | 43.8 (39.0-48.9) | 38.4 (30.9-46.1) | 32.1 (30.8-33.5) | -27.5 (-29.3 to -26.4) | -1.81 (-1.94 to -1.71) | -16.5 (-19.1 to -15.2) | -2.44 (-2.81 to -2.17) |
| **Himachal Pradesh** | **49.9 (47.7-52.7)** | **40.1 (38.6-41.7)** | **32.1 (30.2-34.2)** | **-36.6 (-37.6 to -36.0)** | **-2.57 (-2.64 to -2.50)** | **-20.8 (-22.0 to -20.0)** | **-3.18 (-4.26 to -2.16)** |
| Bilaspur | 46.3 (41.0-51.8) | 36.6 (31.7-41.9) | 29.3 (27.6-31.6) | -37.4 (-38.9 to -36.3) | -2.64 (-2.76 to -2.53) | -20.4 (-22.3 to -18.7) | -3.09 (-3.37 to -2.75) |
| Chamba | 53.0 (46.5-59.4) | 42.6 (37.3-48.5) | 34.7 (32.2-36.9) | -35.3 (-36.8 to -34.3) | -2.46 (-2.57 to -2.35) | -19.3 (-21.2 to -18.0) | -2.88 (-3.17 to -2.62) |
| Hamirpur | 47.9 (42.5-53.7) | 37.3 (32.7-42.4) | 29.4 (27.5-31.2) | -39.4 (-40.8 to -38.4) | -2.83 (-2.94 to -2.72) | -21.9 (-23.6 to -20.2) | -3.34 (-3.60 to -3.01) |
| Kangra | 51.4 (46.1-57.0) | 40.6 (36.0-45.3) | 32.0 (30.2-34.1) | -38.5 (-40.0 to -37.7) | -2.74 (-2.86 to -2.66) | -21.9 (-23.8 to -21.0) | -3.33 (-3.63 to -3.14) |
| Kinnaur | 51.7 (41.2-61.9) | 40.5 (32.9-48.1) | 32.6 (30.2-35.5) | -37.3 (-39.9 to -36.2) | -2.66 (-2.85 to -2.52) | -19.7 (-22.7 to -18.2) | -3.00 (-3.43 to -2.65) |
| Kullu | 51.5 (45.0-58.3) | 41.8 (36.7-47.4) | 32.8 (30.6-34.9) | -37.1 (-38.7 to -35.7) | -2.62 (-2.75 to -2.48) | -22.2 (-24.1 to -20.4) | -3.39 (-3.68 to -3.04) |
| Lahul and Spiti | 49.4 (41.6-57.6) | 39.6 (32.7-46.6) | 31.7 (28.3-34.9) | -36.4 (-38.4 to -35.1) | -2.57 (-2.72 to -2.42) | -20.4 (-22.9 to -19.4) | -3.09 (-3.47 to -2.86) |
| Mandi | 46.9 (40.6-52.2) | 37.8 (33.5-42.7) | 29.7 (28.0-31.4) | -37.5 (-39.0 to -36.7) | -2.65 (-2.77 to -2.57) | -22.3 (-24.2 to -20.9) | -3.40 (-3.70 to -3.12) |
| Shimla | 51.4 (45.9-56.7) | 42.2 (37.5-47.1) | 34.1 (32.6-35.6) | -34.5 (-35.9 to -33.4) | -2.38 (-2.50 to -2.28) | -20.0 (-21.9 to -19.1) | -3.00 (-3.29 to -2.81) |
| Sirmaur | 51.5 (45.7-57.2) | 43.7 (39.1-48.7) | 35.4 (33.4-37.6) | -32.1 (-33.6 to -30.8) | -2.18 (-2.29 to -2.06) | -19.8 (-21.0 to -18.4) | -2.96 (-3.15 to -2.70) |
| Solan | 48.0 (43.3-53.5) | 39.4 (34.9-43.9) | 31.9 (29.9-33.8) | -34.4 (-35.6 to -33.3) | -2.37 (-2.47 to -2.27) | -19.8 (-22.0 to -18.5) | -2.96 (-3.32 to -2.71) |
| Una | 49.7 (44.1-56.0) | 37.4 (32.5-42.7) | 31.9 (30.4-33.9) | -36.6 (-37.8 to -35.7) | -2.57 (-2.66 to -2.48) | -15.3 (-17.3 to -14.1) | -2.21 (-2.50 to -1.99) |
| **UTs other than Delhi** | **46.0 (40.6-53.5)** | **34.9 (31.1-40.8)** | **29.3 (26.1-33.3)** | **-36.9 (-38.8 to -35.7)** | **-2.61 (-2.76 to -2.48)** | **-16.6 (-18.5 to -15.3)** | **-2.48 (-3.95 to -1.01)** |
| Chandigarh | 48.9 (39.1-58.8) | 40.5 (32.0-49.6) | 29.0 (26.4-33.1) | -41.0 (-44.3 to -39.1) | -3.02 (-3.29 to -2.79) | -28.5 (-31.4 to -26.1) | -4.63 (-5.04 to -4.05) |
| Dadra & Nagar Haveli | 58.2 (48.5-67.7) | 58.2 (49.1-67.3) | 45.8 (43.2-48.2) | -21.8 (-24.7 to -20.0) | -1.39 (-1.58 to -1.23) | -21.9 (-24.5 to -20.4) | -3.36 (-3.76 to -3.03) |
| Daman | 49.0 (34.6-65.0) | 47.1 (34.3-60.8) | 34.2 (32.0-38.5) | -29.2 (-35.8 to -26.9) | -2.09 (-2.49 to -1.75) | -26.8 (-33.8 to -24.1) | -4.49 (-5.53 to -3.67) |
| Diu | 52.8 (31.1-74.3) | 51.0 (31.9-73.1) | 34.8 (32.0-38.8) | -31.3 (-40.6 to -30.0) | -2.43 (-2.92 to -2.00) | -29.3 (-38.4 to -27.6) | -5.28 (-6.48 to -4.33) |
| Karaikal | 39.6 (29.0-51.4) | 22.1 (16.3-28.8) | 26.7 (24.5-31.1) | -31.9 (-35.9 to -29.9) | -2.26 (-2.49 to -1.99) | 23.0 (13.6 to 27.9) | 2.85 (1.97 to 3.69) |
| Lakshwadeep | 46.6 (24.2-69.4) | 40.7 (21.2-62.8) | 41.0 (39.8-46.3) | -6.4 (-20.5 to -6.0) | -0.74 (-1.43 to -0.34) | 5.5 (-6.4 to 7.4) | 0.56 (-1.11 to 1.62) |
| Mahe | 34.2 (22.4-47.6) | 26.8 (18.1-38.2) | 26.7 (25.3-31.5) | -19.4 (-28.7 to -16.4) | -1.42 (-1.89 to -0.98) | 2.5 (-8.5 to 5.2) | 0.03 (-1.11 to 0.86) |
| Nicobars | 42.8 (24.1-64.7) | 40.0 (26.4-56.5) | 36.2 (34.1-39.3) | -10.4 (-21.6 to -10.0) | -0.80 (-1.35 to -0.55) | -7.2 (-14.4 to -6.0) | -1.17 (-2.03 to -0.72) |
| North & Middle Andaman | 42.2 (23.3-63.8) | 39.1 (26.5-55.1) | 35.4 (32.9-38.0) | -11.3 (-22.0 to -8.7) | -0.84 (-1.38 to -0.47) | -7.3 (-14.0 to -4.3) | -1.17 (-1.97 to -0.48) |
| Puducherry | 40.9 (29.6-55.6) | 19.4 (13.1-28.1) | 22.1 (20.0-25.5) | -45.7 (-50.2 to -43.5) | -3.57 (-3.90 to -3.20) | 17.0 (4.9 to 21.5) | 2.00 (0.82 to 2.94) |
| South Andaman | 36.2 (19.0-56.2) | 31.7 (20.3-46.7) | 27.1 (24.8-30.1) | -19.8 (-30.2 to -19.8) | -1.48 (-2.01 to -1.22) | -12.0 (-18.9 to -9.4) | -1.97 (-2.78 to -1.25) |
| Yanam | 43.9 (26.0-68.3) | 36.9 (21.4-55.6) | 31.3 (30.1-35.3) | -24.7 (-35.4 to -23.5) | -1.91 (-2.45 to -1.49) | -11.0 (-22.1 to -9.5) | -2.19 (-3.33 to -1.26) |
| **Kerala** | **38.5 (36.6-40.4)** | **28.5 (27.2-29.9)** | **21.6 (20.2-23.0)** | **-44.6 (-45.7 to -43.7)** | **-3.32 (-3.42 to -3.23)** | **-24.9 (-26.1 to -24.0)** | **-3.94 (-4.92 to -2.95)** |
| Alappuzha | 35.2 (28.6-42.5) | 25.2 (21.1-29.7) | 16.4 (15.0-18.3) | -53.8 (-55.6 to -52.6) | -4.36 (-4.54 to -4.18) | -35.4 (-37.6 to -34.0) | -5.92 (-6.30 to -5.56) |
| Ernakulam | 36.4 (30.2-42.4) | 27.2 (23.5-31.1) | 18.4 (16.7-20.1) | -50.1 (-51.6 to -48.8) | -3.93 (-4.07 to -3.75) | -33.2 (-35.8 to -32.1) | -5.45 (-5.93 to -5.18) |
| Idukki | 38.7 (33.3-44.5) | 29.9 (26.1-33.8) | 21.3 (19.9-22.8) | -45.6 (-47.2 to -44.6) | -3.43 (-3.58 to -3.31) | -29.4 (-31.3 to -28.1) | -4.71 (-5.03 to -4.42) |
| Kannur | 39.9 (33.5-46.8) | 29.2 (25.0-33.8) | 24.7 (23.6-26.1) | -38.6 (-40.2 to -37.5) | -2.76 (-2.89 to -2.64) | -15.9 (-17.9 to -14.1) | -2.32 (-2.62 to -1.99) |
| Kasaragod | 38.9 (31.6-46.7) | 27.1 (22.7-32.7) | 22.3 (20.8-24.0) | -43.0 (-45.4 to -41.8) | -3.19 (-3.39 to -3.04) | -17.9 (-20.3 to -15.2) | -2.68 (-3.02 to -2.17) |
| Kollam | 34.5 (26.4-43.9) | 24.0 (19.5-29.2) | 17.2 (14.8-20.5) | -50.2 (-53.0 to -48.5) | -3.98 (-4.22 to -3.72) | -28.6 (-32.0 to -26.7) | -4.64 (-5.17 to -4.16) |
| Kottayam | 34.6 (28.5-41.1) | 25.3 (21.8-29.8) | 16.4 (15.2-17.8) | -53.2 (-54.9 to -52.2) | -4.29 (-4.46 to -4.13) | -35.8 (-37.9 to -34.2) | -5.99 (-6.37 to -5.60) |
| Kozhikode | 41.1 (34.0-49.0) | 31.2 (26.5-36.6) | 25.9 (23.6-28.5) | -37.4 (-39.6 to -36.0) | -2.66 (-2.83 to -2.50) | -17.2 (-20.1 to -15.2) | -2.56 (-2.99 to -2.16) |
| Malappuram | 41.8 (36.5-46.9) | 32.3 (28.7-36.1) | 25.7 (24.7-26.8) | -39.2 (-40.8 to -38.1) | -2.81 (-2.94 to -2.69) | -21.1 (-22.8 to -19.7) | -3.18 (-3.45 to -2.91) |
| Palakkad | 42.7 (37.8-48.2) | 34.2 (30.1-38.6) | 25.8 (24.5-27.2) | -40.3 (-41.8 to -39.4) | -2.91 (-3.04 to -2.81) | -25.2 (-27.0 to -23.7) | -3.92 (-4.21 to -3.62) |
| Pathanamthitta | 35.8 (29.7-42.2) | 26.0 (22.3-30.5) | 18.5 (17.5-19.8) | -49.0 (-50.7 to -47.8) | -3.80 (-3.96 to -3.65) | -29.5 (-31.2 to -28.2) | -4.72 (-5.01 to -4.44) |
| Thiruvananthapuram | 34.6 (26.1-44.3) | 22.8 (17.1-30.1) | 17.7 (14.8-21.1) | -48.8 (-52.5 to -47.1) | -3.85 (-4.17 to -3.57) | -21.5 (-25.9 to -18.3) | -3.44 (-4.00 to -2.68) |
| Thrissur | 38.8 (32.0-45.7) | 29.2 (24.7-34.5) | 22.3 (20.3-24.8) | -43.1 (-45.3 to -42.1) | -3.20 (-3.38 to -3.07) | -24.3 (-27.1 to -22.9) | -3.79 (-4.23 to -3.47) |
| Wayanad | 45.7 (39.5-52.5) | 35.7 (31.5-40.6) | 28.4 (26.4-30.6) | -38.4 (-40.1 to -37.1) | -2.74 (-2.88 to -2.60) | -21.1 (-22.7 to -20.1) | -3.19 (-3.43 to -2.98) |
| **Delhi** | **52.2 (49.2-55.9)** | **40.6 (39.1-42.2)** | **32.3 (30.4-34.2)** | **-39.0 (-40.0 to -38.1)** | **-2.79 (-2.86 to -2.69)** | **-21.3 (-22.8 to -20.2)** | **-3.29 (-4.29 to -2.37)** |
| Central | 51.4 (37.9-63.5) | 40.0 (28.6-51.4) | 31.2 (29.2-35.8) | -39.1 (-44.4 to -37.7) | -2.92 (-3.29 to -2.65) | -21.0 (-28.5 to -20.1) | -3.47 (-4.49 to -2.98) |
| East | 52.3 (39.4-64.6) | 40.8 (30.4-51.6) | 31.0 (28.6-34.7) | -40.8 (-45.2 to -38.8) | -3.05 (-3.37 to -2.76) | -23.5 (-29.3 to -21.6) | -3.84 (-4.64 to -3.24) |
| New Delhi | 52.5 (39.6-64.7) | 41.2 (30.1-52.7) | 32.1 (29.8-36.1) | -38.9 (-44.8 to -37.8) | -2.89 (-3.33 to -2.67) | -21.4 (-28.5 to -19.4) | -3.54 (-4.49 to -2.87) |
| North | 51.7 (41.3-61.8) | 40.0 (30.3-50.6) | 31.6 (29.1-35.8) | -39.3 (-42.9 to -37.9) | -2.87 (-3.15 to -2.67) | -20.9 (-26.0 to -18.7) | -3.31 (-4.03 to -2.75) |
| North East | 51.9 (39.4-64.9) | 40.1 (29.3-51.0) | 30.6 (28.1-34.8) | -41.1 (-45.5 to -38.8) | -3.09 (-3.40 to -2.75) | -23.0 (-28.5 to -19.2) | -3.77 (-4.49 to -2.83) |
| North West | 52.6 (45.2-59.3) | 40.6 (34.5-47.7) | 33.1 (30.7-36.1) | -37.8 (-39.7 to -36.8) | -2.69 (-2.84 to -2.58) | -19.0 (-21.4 to -17.0) | -2.88 (-3.21 to -2.46) |
| Shahdara | 52.4 (34.2-69.9) | 40.5 (25.6-56.5) | 31.2 (29.1-36.1) | -39.5 (-46.1 to -36.6) | -3.08 (-3.47 to -2.56) | -20.6 (-30.4 to -18.9) | -3.70 (-4.86 to -2.77) |
| South | 52.7 (43.4-61.0) | 41.7 (34.5-48.3) | 32.8 (29.7-35.8) | -38.2 (-41.1 to -36.4) | -2.76 (-2.97 to -2.54) | -21.7 (-24.8 to -19.6) | -3.38 (-3.82 to -2.90) |
| South East | 51.5 (40.9-60.9) | 40.6 (32.0-49.8) | 31.5 (29.4-34.9) | -39.2 (-42.8 to -37.5) | -2.86 (-3.13 to -2.64) | -22.3 (-27.0 to -20.0) | -3.54 (-4.22 to -2.97) |
| South West | 52.6 (44.4-60.0) | 40.8 (34.6-46.6) | 33.4 (30.5-36.7) | -37.2 (-39.5 to -35.7) | -2.64 (-2.82 to -2.48) | -18.6 (-22.3 to -16.2) | -2.82 (-3.37 to -2.33) |
| West | 51.7 (42.7-59.7) | 40.1 (32.5-48.2) | 32.2 (29.3-34.8) | -38.3 (-40.9 to -36.5) | -2.76 (-2.95 to -2.54) | -20.0 (-24.3 to -18.1) | -3.09 (-3.72 to -2.64) |
| **Goa** | **33.8 (30.2-40.6)** | **25.3 (23.6-27.0)** | **21.3 (19.6-23.1)** | **-37.2 (-39.6 to -35.8)** | **-2.65 (-2.83 to -2.49)** | **-15.6 (-19.3 to -14.1)** | **-2.41 (-3.80 to -1.17)** |
| North Goa | 32.8 (26.3-39.9) | 25.0 (19.6-30.2) | 20.5 (19.0-22.0) | -37.8 (-40.5 to -36.0) | -2.71 (-2.91 to -2.50) | -17.9 (-21.4 to -15.7) | -2.69 (-3.21 to -2.25) |
| South Goa | 34.6 (28.2-41.4) | 25.5 (20.4-31.0) | 22.0 (20.1-24.2) | -36.6 (-39.0 to -35.1) | -2.60 (-2.78 to -2.42) | -13.5 (-17.2 to -11.9) | -2.00 (-2.50 to -1.63) |

UTs=Union territories.

^*^The states are listed in increasing order of Socio-demographic Index in 2017.

^†^The state of Jammu and Kashmir was divided into two union territories in August 2019; as we are reporting findings up to 2017, we report findings for the undivided state of Jammu and Kashmir.

# **5. Prevalence of wasting in the districts of India in 2000, 2010 and 2017**

| **States*/Districts** | **Wasting percent prevalence (95% uncertainty interval)** | | | | | | |
| --- | --- | --- | --- | --- | --- | --- | --- |
|  | **2000** | **2010** | **2017** | **Percentage change, 2000-2017** | **Annual rate of change, 2000-2017 (%)** | **Percentage change, 2010-2017** | **Annual rate of change 2010-2017 (%)** |
| **India** | **19.2 (18.9-19.6)** | **17.1 (16.9-17.3)** | **15.7 (15.6-15.9)** | **-19.2 (-19.5 to -18.9)** | **-1.18 (-1.20 to -1.15)** | **-9.2 (-9.5 to -8.9)** | **-1.23 (-1.47 to -0.97)** |
| **Bihar** | **22.7 (20.9-24.5)** | **16.3 (15.5-17.0)** | **14.5 (13.8-15.1)** | **-36.9 (-38.4 to -35.9)** | **-2.60 (-2.72 to -2.50)** | **-11.7 (-13.6 to -10.3)** | **-1.69 (-2.76 to -0.68)** |
| Araria | 20.4 (16.7-24.4) | 15.6 (11.8-20.4) | 15.0 (14.1-16.0) | -26.4 (-29.2 to -25.2) | -1.75 (-1.93 to -1.62) | -2.7 (-7.7 to 1.0) | -0.37 (-0.98 to 0.28) |
| Arwal | 22.5 (15.7-29.8) | 17.5 (13.3-22.3) | 18.4 (17.2-19.4) | -16.9 (-21.7 to -14.8) | -1.10 (-1.36 to -0.87) | 5.8 (0.9 to 8.5) | 0.83 (0.27 to 1.30) |
| Aurangabad | 24.1 (16.7-33.6) | 19.7 (15.0-25.5) | 19.4 (18.2-20.4) | -18.1 (-24.0 to -16.6) | -1.19 (-1.52 to -0.99) | -0.6 (-4.9 to 2.5) | -0.07 (-0.57 to 0.49) |
| Banka | 27.8 (19.2-37.3) | 20.0 (15.1-24.6) | 19.6 (18.7-20.5) | -28.4 (-33.2 to -26.6) | -1.95 (-2.26 to -1.72) | -1.4 (-6.2 to 0.4) | -0.15 (-0.77 to 0.19) |
| Begusarai | 21.4 (15.4-28.1) | 14.7 (11.3-19.0) | 14.0 (13.5-14.5) | -34.3 (-38.1 to -32.8) | -2.42 (-2.69 to -2.22) | -4.5 (-8.9 to -2.2) | -0.63 (-1.16 to -0.17) |
| Bhagalpur | 23.6 (17.5-31.5) | 16.0 (12.4-19.5) | 15.9 (15.3-16.7) | -32.1 (-35.2 to -29.9) | -2.23 (-2.44 to -1.99) | -0.2 (-4.2 to 1.5) | 0.02 (-0.46 to 0.36) |
| Bhojpur | 21.8 (15.5-29.3) | 16.0 (11.9-20.6) | 16.8 (16.0-17.6) | -21.7 (-25.9 to -20.1) | -1.43 (-1.67 to -1.24) | 6.2 (1.3 to 9.2) | 0.87 (0.33 to 1.40) |
| Buxar | 20.2 (14.5-27.2) | 14.4 (11.0-18.1) | 14.1 (13.5-14.8) | -29.6 (-34.0 to -27.1) | -2.03 (-2.32 to -1.76) | -1.8 (-5.6 to 0.7) | -0.22 (-0.68 to 0.25) |
| Darbhanga | 23.5 (19.5-28.5) | 15.0 (12.1-18.3) | 12.6 (12.1-13.1) | -46.9 (-48.5 to -46.0) | -3.57 (-3.72 to -3.46) | -15.7 (-19.4 to -14.1) | -2.33 (-2.87 to -1.99) |
| East Champaran | 24.7 (20.9-28.7) | 19.1 (15.6-23.2) | 12.8 (12.3-13.4) | -48.9 (-50.2 to -47.9) | -3.79 (-3.90 to -3.66) | -33.5 (-35.5 to -31.7) | -5.53 (-5.86 to -5.11) |
| Gaya | 24.1 (16.5-33.5) | 18.0 (13.6-22.6) | 18.0 (17.3-18.8) | -24.0 (-28.7 to -21.8) | -1.61 (-1.89 to -1.36) | 0.5 (-4.2 to 2.1) | 0.11 (-0.46 to 0.44) |
| Gopalganj | 23.9 (19.0-28.9) | 18.9 (15.4-23.4) | 12.8 (12.2-13.6) | -46.7 (-49.0 to -45.3) | -3.57 (-3.77 to -3.38) | -32.4 (-34.4 to -30.7) | -5.32 (-5.64 to -4.91) |
| Jamui | 25.3 (16.6-35.3) | 18.9 (14.1-24.0) | 18.0 (17.3-18.8) | -27.3 (-32.8 to -25.0) | -1.88 (-2.23 to -1.60) | -4.3 (-8.8 to -2.5) | -0.59 (-1.15 to -0.22) |
| Jehanabad | 22.4 (15.3-30.8) | 16.6 (12.6-21.2) | 17.8 (16.6-18.8) | -19.2 (-25.5 to -17.1) | -1.27 (-1.64 to -1.03) | 7.9 (2.4 to 9.8) | 1.09 (0.48 to 1.48) |
| Kaimur | 20.3 (14.2-27.9) | 13.7 (10.4-17.6) | 12.5 (11.9-13.4) | -37.3 (-41.5 to -34.7) | -2.71 (-3.01 to -2.39) | -7.9 (-12.7 to -5.8) | -1.15 (-1.76 to -0.70) |
| Katihar | 21.6 (16.3-27.8) | 13.6 (10.9-16.5) | 13.8 (13.2-14.6) | -35.8 (-38.7 to -33.9) | -2.54 (-2.74 to -2.32) | 2.0 (-2.2 to 4.5) | 0.34 (-0.18 to 0.77) |
| Khagaria | 21.8 (15.6-28.7) | 14.4 (10.7-19.1) | 14.4 (13.8-15.1) | -33.2 (-37.4 to -31.2) | -2.33 (-2.63 to -2.09) | 1.2 (-3.4 to 3.7) | 0.16 (-0.35 to 0.65) |
| Kishanganj | 21.0 (16.8-25.7) | 15.4 (12.0-19.0) | 14.4 (13.5-15.4) | -31.5 (-34.2 to -30.3) | -2.15 (-2.34 to -2.02) | -6.2 (-9.7 to -3.8) | -0.85 (-1.29 to -0.41) |
| Lakhisarai | 22.6 (14.3-32.3) | 16.5 (11.9-21.5) | 15.9 (14.9-16.9) | -27.8 (-33.0 to -26.2) | -1.93 (-2.24 to -1.69) | -2.3 (-7.1 to 0.3) | -0.35 (-0.90 to 0.18) |
| Madhepura | 21.2 (16.6-25.8) | 14.4 (11.3-18.0) | 14.2 (13.7-14.7) | -33.0 (-35.9 to -31.6) | -2.28 (-2.49 to -2.12) | -1.0 (-4.8 to 2.1) | -0.10 (-0.55 to 0.44) |
| Madhubani | 25.6 (22.2-29.6) | 16.6 (13.2-20.5) | 13.3 (12.5-14.2) | -48.6 (-49.9 to -47.4) | -3.75 (-3.88 to -3.60) | -19.4 (-22.7 to -17.8) | -2.96 (-3.43 to -2.59) |
| Munger | 22.7 (16.0-30.1) | 15.8 (12.0-20.1) | 15.8 (15.0-16.8) | -29.5 (-34.6 to -28.7) | -2.03 (-2.38 to -1.89) | 0.6 (-4.1 to 2.7) | 0.11 (-0.45 to 0.53) |
| Muzaffarpur | 22.1 (17.3-27.6) | 14.9 (11.4-18.7) | 11.3 (10.8-11.8) | -49.3 (-51.5 to -47.9) | -3.85 (-4.06 to -3.65) | -24.1 (-27.8 to -21.9) | -3.79 (-4.36 to -3.30) |
| Nalanda | 21.8 (15.5-29.2) | 16.0 (12.4-19.9) | 15.8 (15.1-16.3) | -26.5 (-30.8 to -24.0) | -1.79 (-2.06 to -1.53) | -1.0 (-5.2 to 0.6) | -0.11 (-0.61 to 0.23) |
| Nawada | 23.0 (15.8-32.7) | 16.8 (12.6-21.2) | 16.6 (15.8-17.3) | -26.4 (-31.2 to -23.9) | -1.81 (-2.09 to -1.52) | -0.9 (-5.5 to 0.5) | -0.10 (-0.66 to 0.21) |
| Patna | 21.2 (16.3-26.9) | 15.1 (12.0-18.6) | 14.8 (14.4-15.2) | -30.0 (-33.3 to -28.4) | -2.05 (-2.27 to -1.87) | -2.3 (-6.0 to -0.1) | -0.27 (-0.73 to 0.13) |
| Purnia | 21.0 (16.4-26.0) | 14.1 (11.2-17.4) | 14.1 (13.5-14.6) | -33.1 (-35.4 to -31.4) | -2.29 (-2.45 to -2.11) | 0.3 (-3.8 to 2.6) | 0.08 (-0.41 to 0.50) |
| Rohtas | 21.6 (15.2-29.6) | 16.7 (12.7-21.2) | 16.3 (15.7-17.0) | -23.3 (-28.1 to -22.1) | -1.55 (-1.84 to -1.38) | -1.9 (-6.7 to 1.1) | -0.24 (-0.83 to 0.29) |
| Saharsa | 22.1 (16.6-28.5) | 14.5 (10.7-18.9) | 14.1 (13.5-14.8) | -35.9 (-39.0 to -34.6) | -2.55 (-2.77 to -2.38) | -1.2 (-5.8 to 1.6) | -0.17 (-0.70 to 0.37) |
| Samastipur | 21.6 (16.8-27.3) | 14.4 (11.2-18.1) | 12.8 (12.6-13.2) | -40.6 (-42.8 to -39.4) | -2.97 (-3.13 to -2.81) | -10.5 (-14.2 to -8.5) | -1.51 (-2.00 to -1.11) |
| Saran | 21.9 (17.0-27.6) | 15.6 (12.4-19.5) | 13.7 (13.2-14.2) | -37.7 (-40.4 to -36.3) | -2.70 (-2.90 to -2.53) | -12.3 (-14.9 to -9.9) | -1.79 (-2.12 to -1.33) |
| Sheikhpura | 22.4 (14.4-31.7) | 16.8 (12.5-21.6) | 15.9 (14.9-16.9) | -27.0 (-33.3 to -24.7) | -1.87 (-2.27 to -1.58) | -4.5 (-10.0 to -2.1) | -0.65 (-1.33 to -0.16) |
| Sheohar | 23.2 (19.0-28.1) | 16.5 (12.8-20.5) | 11.6 (10.7-12.7) | -50.3 (-52.4 to -49.1) | -3.95 (-4.15 to -3.79) | -29.5 (-33.0 to -27.6) | -4.80 (-5.36 to -4.33) |
| Sitamarhi | 24.8 (21.4-28.3) | 17.4 (14.0-20.8) | 12.5 (11.9-13.2) | -50.5 (-51.8 to -49.4) | -3.96 (-4.09 to -3.82) | -28.6 (-30.7 to -26.9) | -4.58 (-4.91 to -4.19) |
| Siwan | 23.3 (18.1-28.4) | 17.7 (14.1-22.1) | 13.4 (12.8-14.0) | -42.9 (-45.3 to -41.5) | -3.18 (-3.39 to -3.01) | -24.7 (-27.3 to -22.9) | -3.87 (-4.27 to -3.47) |
| Supaul | 23.0 (19.6-26.6) | 15.8 (12.5-19.6) | 14.3 (13.8-15.0) | -38.2 (-39.9 to -37.0) | -2.72 (-2.86 to -2.59) | -8.8 (-11.7 to -6.6) | -1.24 (-1.60 to -0.83) |
| Vaishali | 21.3 (16.6-27.3) | 14.6 (11.4-18.2) | 12.5 (12.0-13.0) | -41.4 (-44.1 to -39.5) | -3.05 (-3.26 to -2.82) | -14.0 (-18.1 to -11.8) | -2.08 (-2.64 to -1.62) |
| West Champaran | 22.8 (18.7-27.0) | 19.6 (16.0-23.4) | 12.6 (12.1-13.3) | -44.9 (-47.0 to -43.9) | -3.38 (-3.56 to -3.25) | -35.8 (-38.4 to -34.3) | -6.00 (-6.48 to -5.63) |
| **Madhya Pradesh** | **22.3 (21.2-23.4)** | **21.1 (20.4-21.9)** | **18.8 (18.1-19.4)** | **-16.8 (-17.5 to -16.0)** | **-1.01 (-1.05 to -0.95)** | **-12.2 (-13.2 to -11.7)** | **-1.71 (-2.53 to -0.94)** |
| Agar Malwa | 24.6 (19.0-30.1) | 22.6 (17.8-27.8) | 21.9 (20.4-23.4) | -10.8 (-15.4 to -9.1) | -0.65 (-0.91 to -0.50) | -3.0 (-7.8 to -0.7) | -0.38 (-1.00 to 0.05) |
| Alirajpur | 24.6 (20.1-30.2) | 29.6 (25.4-33.8) | 25.6 (24.3-27.1) | 4.5 (0.9 to 7.3) | 0.28 (0.11 to 0.47) | -13.9 (-16.2 to -12.6) | -1.99 (-2.34 to -1.75) |
| Anuppur | 23.0 (17.8-28.8) | 17.6 (13.8-22.2) | 19.7 (18.4-21.1) | -14.1 (-18.1 to -13.0) | -0.87 (-1.10 to -0.75) | 12.7 (7.5 to 14.4) | 1.74 (1.17 to 2.07) |
| Ashoknagar | 24.2 (19.5-29.4) | 19.5 (15.4-24.5) | 18.8 (17.6-20.3) | -22.7 (-25.9 to -21.4) | -1.47 (-1.67 to -1.33) | -3.3 (-7.5 to -1.5) | -0.44 (-0.95 to -0.08) |
| Balaghat | 23.7 (19.3-28.9) | 21.6 (18.0-26.1) | 21.1 (20.0-22.3) | -11.0 (-13.4 to -8.9) | -0.65 (-0.78 to -0.48) | -2.8 (-5.2 to -0.9) | -0.31 (-0.62 to 0.01) |
| Barwani | 26.5 (22.5-30.9) | 25.5 (21.0-29.7) | 23.4 (22.6-24.5) | -12.2 (-14.3 to -11.1) | -0.72 (-0.84 to -0.63) | -8.4 (-11.2 to -7.2) | -1.15 (-1.52 to -0.91) |
| Betul | 20.3 (16.6-24.2) | 20.2 (16.1-24.4) | 20.0 (18.6-21.5) | -1.4 (-4.9 to 0.2) | -0.05 (-0.23 to 0.07) | -0.7 (-4.8 to 0.8) | -0.05 (-0.56 to 0.26) |
| Bhind | 19.1 (15.5-22.7) | 14.3 (11.4-17.7) | 16.1 (15.5-16.7) | -15.9 (-18.8 to -14.4) | -0.98 (-1.14 to -0.84) | 13.0 (8.5 to 15.6) | 1.80 (1.30 to 2.22) |
| Bhopal | 19.9 (15.5-25.1) | 18.5 (13.3-25.0) | 15.5 (14.2-16.9) | -21.5 (-25.3 to -19.1) | -1.40 (-1.62 to -1.17) | -14.9 (-19.8 to -12.5) | -2.31 (-2.94 to -1.73) |
| Burhanpur | 26.3 (20.9-31.8) | 18.4 (14.2-23.9) | 15.5 (14.4-16.8) | -41.3 (-43.7 to -40.0) | -3.03 (-3.22 to -2.87) | -15.3 (-18.7 to -12.8) | -2.29 (-2.74 to -1.77) |
| Chhatarpur | 21.9 (17.8-26.4) | 21.4 (18.4-24.9) | 17.1 (16.0-18.1) | -22.2 (-24.5 to -20.7) | -1.43 (-1.56 to -1.28) | -20.7 (-22.9 to -19.0) | -3.12 (-3.47 to -2.80) |
| Chhindwara | 21.3 (17.3-25.1) | 19.8 (16.0-24.2) | 19.7 (18.6-21.0) | -7.3 (-10.6 to -5.4) | -0.41 (-0.59 to -0.27) | -0.1 (-3.4 to 2.9) | 0.05 (-0.35 to 0.55) |
| Damoh | 20.5 (16.6-24.8) | 20.8 (17.1-24.5) | 16.1 (15.0-17.2) | -21.5 (-23.8 to -19.8) | -1.38 (-1.51 to -1.22) | -22.6 (-25.2 to -20.8) | -3.49 (-3.89 to -3.10) |
| Datia | 22.0 (17.8-26.1) | 19.4 (16.0-23.4) | 20.4 (19.3-21.5) | -7.7 (-10.8 to -6.1) | -0.44 (-0.60 to -0.31) | 5.1 (2.1 to 7.6) | 0.79 (0.44 to 1.18) |
| Dewas | 23.4 (19.5-27.6) | 21.3 (17.6-25.5) | 17.4 (16.6-18.1) | -26.2 (-28.5 to -24.9) | -1.72 (-1.88 to -1.59) | -18.8 (-21.2 to -17.2) | -2.83 (-3.17 to -2.49) |
| Dhar | 23.4 (19.9-27.8) | 26.1 (22.8-29.4) | 19.0 (18.4-19.7) | -19.3 (-21.2 to -17.5) | -1.20 (-1.32 to -1.06) | -27.8 (-29.4 to -27.2) | -4.39 (-4.66 to -4.24) |
| Dindori | 22.2 (17.3-27.8) | 21.0 (16.6-25.8) | 21.4 (20.3-22.6) | -2.9 (-7.0 to -0.3) | -0.16 (-0.37 to 0.04) | 2.2 (-1.2 to 4.7) | 0.37 (-0.03 to 0.80) |
| Guna | 24.7 (19.5-30.2) | 17.6 (14.2-22.0) | 19.2 (18.0-20.3) | -22.1 (-25.0 to -20.5) | -1.43 (-1.60 to -1.27) | 9.2 (5.3 to 11.3) | 1.30 (0.88 to 1.67) |
| Gwalior | 21.9 (17.8-26.3) | 17.6 (13.5-22.2) | 19.9 (18.6-21.3) | -9.1 (-11.9 to -6.7) | -0.53 (-0.68 to -0.34) | 13.8 (8.9 to 16.3) | 1.89 (1.36 to 2.31) |
| Harda | 21.4 (16.5-27.6) | 21.1 (16.5-26.6) | 14.9 (13.9-16.2) | -29.8 (-32.9 to -27.8) | -2.04 (-2.23 to -1.82) | -28.9 (-31.9 to -26.9) | -4.68 (-5.14 to -4.19) |
| Hoshangabad | 20.2 (16.2-24.3) | 22.3 (17.7-27.6) | 19.6 (18.5-20.8) | -3.0 (-6.7 to -0.7) | -0.15 (-0.35 to 0.02) | -12.1 (-15.2 to -10.4) | -1.75 (-2.16 to -1.41) |
| Indore | 21.4 (16.9-26.4) | 20.1 (16.1-24.6) | 15.3 (13.9-16.7) | -28.8 (-31.9 to -27.1) | -1.94 (-2.15 to -1.76) | -24.0 (-27.1 to -22.0) | -3.77 (-4.22 to -3.31) |
| Jabalpur | 21.3 (17.4-25.9) | 25.8 (21.6-29.7) | 20.6 (19.3-21.9) | -3.0 (-5.7 to -0.1) | -0.15 (-0.28 to 0.06) | -20.5 (-22.9 to -19.4) | -3.11 (-3.47 to -2.87) |
| Jhabua | 23.4 (19.4-28.4) | 29.4 (25.0-33.7) | 22.0 (21.0-23.2) | -6.0 (-9.0 to -4.0) | -0.33 (-0.49 to -0.18) | -25.5 (-27.5 to -24.2) | -3.98 (-4.31 to -3.70) |
| Katni | 22.1 (17.9-26.6) | 23.7 (19.6-27.7) | 20.3 (19.4-21.4) | -8.0 (-11.2 to -6.1) | -0.46 (-0.63 to -0.31) | -14.7 (-17.0 to -13.3) | -2.13 (-2.45 to -1.86) |
| Khandwa | 24.3 (20.4-28.9) | 21.4 (17.8-25.6) | 15.5 (14.9-16.2) | -36.6 (-38.7 to -35.0) | -2.58 (-2.74 to -2.41) | -27.8 (-29.4 to -26.1) | -4.41 (-4.66 to -4.05) |
| Khargone | 26.7 (22.6-30.9) | 23.6 (19.2-27.8) | 17.5 (16.4-18.6) | -35.1 (-37.3 to -34.3) | -2.45 (-2.62 to -2.35) | -26.3 (-28.8 to -24.9) | -4.15 (-4.55 to -3.82) |
| Mandla | 22.3 (18.0-27.6) | 22.9 (18.6-27.8) | 22.3 (21.1-23.6) | 0.4 (-2.6 to 2.7) | 0.05 (-0.09 to 0.21) | -2.6 (-5.8 to -0.9) | -0.30 (-0.71 to 0.01) |
| Mandsaur | 22.5 (18.0-27.4) | 26.3 (22.1-30.3) | 19.9 (18.6-21.2) | -11.5 (-15.1 to -9.1) | -0.69 (-0.89 to -0.49) | -24.7 (-27.1 to -23.6) | -3.85 (-4.23 to -3.59) |
| Morena | 19.4 (16.2-22.8) | 14.4 (11.4-17.5) | 18.2 (17.6-18.9) | -6.4 (-8.9 to -4.6) | -0.35 (-0.48 to -0.22) | 27.1 (22.7 to 29.6) | 3.52 (3.09 to 3.89) |
| Narsinghpur | 21.5 (17.5-26.5) | 21.7 (17.1-27.0) | 17.6 (16.3-19.0) | -18.1 (-20.8 to -15.7) | -1.14 (-1.29 to -0.93) | -18.7 (-22.7 to -16.7) | -2.85 (-3.43 to -2.41) |
| Neemuch | 20.7 (16.7-25.5) | 21.4 (17.6-25.1) | 20.2 (18.8-21.6) | -2.5 (-6.5 to -0.2) | -0.13 (-0.33 to 0.05) | -5.7 (-8.8 to -4.3) | -0.75 (-1.16 to -0.48) |
| Niwari | 23.6 (18.7-28.6) | 23.6 (19.5-27.9) | 21.1 (19.7-22.7) | -10.5 (-14.0 to -8.3) | -0.63 (-0.82 to -0.44) | -11.0 (-14.2 to -9.5) | -1.56 (-2.00 to -1.26) |
| Panna | 21.6 (17.1-26.4) | 22.6 (18.6-26.6) | 18.7 (17.7-20.1) | -13.3 (-16.6 to -11.3) | -0.81 (-0.99 to -0.63) | -17.7 (-20.3 to -16.5) | -2.64 (-3.02 to -2.37) |
| Raisen | 20.5 (17.3-24.0) | 22.1 (17.4-27.6) | 18.3 (17.3-19.4) | -11.2 (-13.4 to -10.0) | -0.66 (-0.78 to -0.55) | -17.2 (-20.4 to -15.1) | -2.58 (-3.03 to -2.15) |
| Rajgarh | 25.4 (21.0-30.5) | 21.0 (17.4-25.0) | 20.8 (19.7-21.7) | -18.5 (-20.7 to -17.4) | -1.15 (-1.28 to -1.05) | -1.1 (-4.5 to 1.3) | -0.08 (-0.51 to 0.33) |
| Ratlam | 22.1 (17.9-26.9) | 26.6 (21.4-31.9) | 20.3 (19.1-21.6) | -8.0 (-11.6 to -5.6) | -0.47 (-0.66 to -0.27) | -23.7 (-26.3 to -22.1) | -3.68 (-4.08 to -3.33) |
| Rewa | 21.0 (17.5-25.1) | 18.8 (15.6-22.2) | 16.3 (15.5-17.4) | -22.3 (-25.0 to -20.7) | -1.43 (-1.60 to -1.28) | -13.5 (-15.8 to -11.3) | -1.95 (-2.27 to -1.55) |
| Sagar | 22.1 (18.3-26.4) | 22.4 (18.0-26.9) | 18.0 (17.0-19.0) | -19.1 (-21.3 to -17.4) | -1.20 (-1.33 to -1.05) | -19.9 (-23.6 to -18.5) | -3.04 (-3.59 to -2.71) |
| Satna | 21.7 (18.0-26.3) | 19.9 (16.6-23.5) | 17.0 (16.1-17.8) | -21.9 (-24.5 to -20.0) | -1.40 (-1.57 to -1.23) | -14.8 (-17.2 to -13.5) | -2.15 (-2.49 to -1.89) |
| Sehore | 22.0 (18.5-25.9) | 20.6 (16.4-25.8) | 17.5 (16.9-18.3) | -20.9 (-23.0 to -19.5) | -1.32 (-1.45 to -1.20) | -15.0 (-18.9 to -13.5) | -2.22 (-2.77 to -1.89) |
| Seoni | 23.0 (18.4-27.9) | 21.8 (17.4-26.9) | 20.2 (19.1-21.5) | -12.1 (-15.5 to -10.1) | -0.73 (-0.92 to -0.56) | -7.0 (-10.5 to -5.4) | -0.97 (-1.42 to -0.64) |
| Shahdol | 21.9 (17.3-27.1) | 16.2 (12.3-20.3) | 16.1 (15.2-17.3) | -26.3 (-30.0 to -24.0) | -1.75 (-2.00 to -1.52) | 0.2 (-4.0 to 1.7) | 0.06 (-0.44 to 0.39) |
| Shajapur | 24.8 (20.0-29.9) | 21.6 (17.2-26.3) | 20.2 (18.9-21.4) | -19.0 (-22.1 to -17.7) | -1.19 (-1.38 to -1.07) | -6.6 (-10.4 to -4.7) | -0.91 (-1.40 to -0.54) |
| Sheopur | 19.5 (15.6-24.6) | 12.7 (9.4-16.6) | 18.7 (17.6-19.8) | -3.9 (-6.8 to -1.5) | -0.21 (-0.35 to -0.03) | 48.6 (40.8 to 52.2) | 5.77 (5.12 to 6.28) |
| Shivpuri | 23.1 (19.3-27.2) | 17.1 (13.4-20.8) | 18.1 (17.2-19.2) | -22.2 (-24.8 to -20.8) | -1.41 (-1.59 to -1.29) | 6.4 (2.7 to 9.2) | 0.94 (0.53 to 1.40) |
| Sidhi | 21.5 (18.1-26.0) | 19.0 (15.4-22.9) | 18.2 (17.3-19.2) | -15.6 (-18.1 to -13.6) | -0.95 (-1.10 to -0.79) | -4.3 (-7.9 to -1.7) | -0.55 (-1.01 to -0.09) |
| Singrauli | 22.7 (18.1-27.7) | 21.2 (16.7-25.9) | 22.5 (21.2-23.9) | -0.7 (-4.6 to 1.8) | -0.02 (-0.22 to 0.16) | 6.6 (2.2 to 9.1) | 0.96 (0.46 to 1.38) |
| Tikamgarh | 23.0 (18.5-27.9) | 23.4 (19.6-27.3) | 18.6 (17.6-19.7) | -19.1 (-21.3 to -17.7) | -1.20 (-1.33 to -1.07) | -20.7 (-23.1 to -19.1) | -3.14 (-3.51 to -2.81) |
| Ujjain | 22.8 (19.2-26.8) | 22.9 (19.6-26.8) | 18.9 (18.1-19.7) | -17.5 (-19.9 to -15.9) | -1.08 (-1.23 to -0.95) | -18.1 (-20.5 to -16.8) | -2.69 (-3.05 to -2.42) |
| Umaria | 21.6 (17.0-26.5) | 19.7 (15.7-24.2) | 18.2 (17.3-19.4) | -15.3 (-19.1 to -13.3) | -0.95 (-1.17 to -0.77) | -7.2 (-10.2 to -5.1) | -1.00 (-1.37 to -0.60) |
| Vidisha | 23.1 (19.1-27.2) | 22.7 (17.7-28.4) | 20.2 (19.0-21.6) | -12.9 (-15.3 to -11.3) | -0.77 (-0.91 to -0.64) | -10.6 (-14.3 to -9.0) | -1.53 (-2.02 to -1.19) |
| **Jharkhand** | **27.3 (23.8-30.4)** | **19.4 (18.5-20.3)** | **19.0 (18.3-19.7)** | **-30.7 (-33.1 to -29.4)** | **-2.08 (-2.25 to -1.95)** | **-2.7 (-4.4 to -1.4)** | **-0.30 (-1.13 to 0.55)** |
| Bokaro | 26.3 (18.0-36.3) | 20.9 (17.5-25.5) | 18.5 (17.5-19.5) | -28.7 (-33.7 to -27.2) | -1.98 (-2.30 to -1.77) | -11.7 (-14.4 to -9.9) | -1.68 (-2.04 to -1.32) |
| Chatra | 25.7 (16.9-36.3) | 16.0 (11.6-20.7) | 15.8 (15.1-16.5) | -37.2 (-41.5 to -36.3) | -2.72 (-3.01 to -2.53) | -0.5 (-5.1 to 2.1) | -0.07 (-0.60 to 0.43) |
| Deoghar | 26.8 (19.5-35.0) | 18.8 (15.3-22.5) | 19.6 (18.8-20.5) | -26.0 (-29.8 to -24.8) | -1.74 (-1.98 to -1.58) | 4.4 (0.7 to 6.7) | 0.68 (0.25 to 1.06) |
| Dhanbad | 26.9 (19.2-35.8) | 20.7 (17.3-24.7) | 19.1 (18.1-20.1) | -28.2 (-32.7 to -26.8) | -1.93 (-2.22 to -1.74) | -8.2 (-10.8 to -6.1) | -1.13 (-1.46 to -0.74) |
| Dumka | 28.5 (22.0-36.5) | 19.7 (16.5-23.3) | 21.4 (20.4-22.4) | -24.6 (-27.2 to -22.6) | -1.62 (-1.77 to -1.42) | 8.3 (4.3 to 11.0) | 1.22 (0.75 to 1.63) |
| East Singhbhum | 29.4 (23.4-36.5) | 22.7 (18.3-27.6) | 19.8 (18.3-21.4) | -32.7 (-36.0 to -30.6) | -2.26 (-2.51 to -2.04) | -12.6 (-15.7 to -10.9) | -1.82 (-2.25 to -1.48) |
| Garhwa | 26.6 (19.7-35.6) | 15.2 (11.2-19.7) | 17.8 (16.9-18.8) | -32.2 (-35.1 to -30.5) | -2.25 (-2.42 to -2.04) | 19.1 (12.7 to 23.1) | 2.50 (1.85 to 3.13) |
| Giridih | 26.0 (17.8-34.5) | 18.7 (15.2-22.3) | 18.2 (17.5-18.9) | -29.0 (-34.1 to -28.2) | -2.00 (-2.33 to -1.85) | -2.8 (-5.8 to -1.5) | -0.32 (-0.69 to -0.07) |
| Godda | 24.7 (17.5-33.1) | 16.5 (12.6-20.2) | 18.4 (17.4-19.4) | -24.2 (-28.4 to -21.1) | -1.62 (-1.87 to -1.31) | 12.7 (7.9 to 15.6) | 1.75 (1.22 to 2.22) |
| Gumla | 29.4 (20.8-38.5) | 20.5 (15.8-25.7) | 21.7 (20.5-22.7) | -25.5 (-30.4 to -23.4) | -1.71 (-2.03 to -1.48) | 6.5 (1.8 to 8.6) | 0.93 (0.40 to 1.32) |
| Hazaribagh | 25.9 (17.0-37.1) | 16.8 (12.3-21.7) | 15.5 (14.9-16.2) | -38.7 (-43.2 to -37.6) | -2.85 (-3.17 to -2.64) | -7.0 (-11.5 to -4.9) | -1.01 (-1.57 to -0.57) |
| Jamtara | 28.8 (22.2-36.8) | 20.8 (17.5-24.7) | 20.7 (19.6-21.9) | -27.7 (-30.7 to -26.0) | -1.86 (-2.05 to -1.68) | -0.4 (-3.4 to 1.6) | 0.03 (-0.35 to 0.37) |
| Khunti | 30.1 (21.2-39.9) | 22.8 (18.1-28.4) | 21.2 (20.1-22.3) | -28.6 (-33.1 to -27.5) | -1.96 (-2.25 to -1.79) | -6.9 (-10.1 to -4.5) | -0.95 (-1.36 to -0.51) |
| Koderma | 25.6 (17.4-35.4) | 16.9 (12.9-21.3) | 16.8 (16.0-17.7) | -33.1 (-37.1 to -31.6) | -2.34 (-2.60 to -2.12) | 0.2 (-4.4 to 1.9) | 0.05 (-0.50 to 0.41) |
| Latehar | 26.8 (18.8-36.0) | 16.4 (12.2-21.1) | 17.9 (17.2-18.6) | -32.5 (-36.5 to -30.8) | -2.28 (-2.54 to -2.06) | 9.6 (6.0 to 12.0) | 1.33 (0.97 to 1.76) |
| Lohardaga | 26.3 (17.9-35.2) | 17.8 (13.5-22.4) | 18.6 (17.5-19.6) | -28.5 (-33.2 to -26.5) | -1.95 (-2.26 to -1.72) | 5.2 (0.9 to 6.7) | 0.75 (0.26 to 1.06) |
| Pakur | 24.8 (19.8-30.3) | 18.7 (15.8-21.8) | 20.5 (19.4-21.5) | -17.7 (-20.9 to -15.7) | -1.11 (-1.30 to -0.93) | 9.4 (6.7 to 11.2) | 1.37 (1.06 to 1.66) |
| Palamu | 25.7 (17.8-34.8) | 15.2 (11.5-19.4) | 17.3 (16.3-18.3) | -31.5 (-36.5 to -29.0) | -2.20 (-2.55 to -1.92) | 15.2 (9.5 to 18.7) | 2.04 (1.44 to 2.60) |
| Ramgarh | 27.4 (19.1-37.6) | 19.8 (15.9-24.9) | 17.8 (17.1-18.8) | -34.3 (-38.4 to -32.7) | -2.44 (-2.72 to -2.22) | -9.9 (-13.5 to -8.5) | -1.42 (-1.90 to -1.11) |
| Ranchi | 28.6 (20.3-38.4) | 21.0 (16.7-26.0) | 19.4 (18.6-20.4) | -31.2 (-35.2 to -29.8) | -2.17 (-2.43 to -1.98) | -7.2 (-10.9 to -5.4) | -0.99 (-1.47 to -0.64) |
| Sahibganj | 22.5 (17.2-28.7) | 16.4 (13.6-19.6) | 18.9 (17.7-19.9) | -15.7 (-18.9 to -14.1) | -0.98 (-1.15 to -0.82) | 15.1 (12.1 to 17.1) | 2.09 (1.77 to 2.40) |
| Saraikela Kharsawan | 30.2 (23.4-38.3) | 23.9 (19.8-28.1) | 21.1 (20.0-22.3) | -29.8 (-32.4 to -28.3) | -2.03 (-2.19 to -1.86) | -11.5 (-13.9 to -9.9) | -1.64 (-1.95 to -1.32) |
| Simdega | 29.5 (22.5-36.6) | 19.4 (15.8-23.7) | 18.7 (17.8-19.7) | -36.8 (-39.4 to -35.0) | -2.62 (-2.81 to -2.42) | -3.7 (-7.4 to -1.0) | -0.47 (-0.94 to -0.01) |
| West Singhbhum | 30.6 (23.8-37.6) | 22.4 (18.9-26.4) | 20.1 (19.1-21.0) | -34.4 (-37.0 to -33.4) | -2.40 (-2.59 to -2.28) | -10.6 (-13.3 to -8.7) | -1.48 (-1.86 to -1.14) |
| **Uttar Pradesh** | **16.3 (15.3-17.2)** | **12.5 (12.0-13.1)** | **13.0 (12.5-13.5)** | **-20.8 (-21.8 to -19.9)** | **-1.29 (-1.36 to -1.22)** | **3.1 (2.1 to 4.3)** | **0.55 (-0.37 to 1.44)** |
| Agra | 15.8 (12.3-19.7) | 8.6 (6.4-11.2) | 12.3 (11.8-13.1) | -21.8 (-25.1 to -19.9) | -1.41 (-1.61 to -1.23) | 45.1 (37.7 to 49.2) | 5.42 (4.78 to 5.99) |
| Aligarh | 14.4 (10.9-18.3) | 5.9 (4.4-7.7) | 10.9 (10.4-11.4) | -24.1 (-26.8 to -21.8) | -1.58 (-1.74 to -1.36) | 87.6 (77.9 to 91.7) | 9.33 (8.66 to 9.83) |
| Allahabad | 20.3 (16.0-24.4) | 15.1 (13.0-17.4) | 14.0 (13.4-14.8) | -30.8 (-33.7 to -29.9) | -2.10 (-2.30 to -1.98) | -7.4 (-9.8 to -6.0) | -0.98 (-1.31 to -0.74) |
| Ambedkar Nagar | 16.5 (13.1-20.8) | 14.7 (12.4-17.4) | 12.0 (11.4-12.6) | -27.3 (-30.2 to -25.5) | -1.82 (-2.01 to -1.64) | -18.9 (-21.3 to -17.1) | -2.83 (-3.19 to -2.48) |
| Amethi | 21.0 (17.0-24.9) | 19.9 (17.1-22.9) | 17.3 (16.5-18.0) | -17.7 (-20.5 to -16.1) | -1.10 (-1.26 to -0.96) | -13.5 (-14.9 to -11.7) | -1.93 (-2.11 to -1.61) |
| Amroha | 11.5 (8.8-15.0) | 6.5 (5.1-8.1) | 12.3 (11.9-12.9) | 8.5 (3.5 to 11.4) | 0.47 (0.26 to 0.69) | 92.1 (85.7 to 95.3) | 9.75 (9.33 to 10.11) |
| Auraiya | 18.1 (13.6-23.3) | 11.6 (9.0-15.0) | 12.4 (11.9-12.9) | -31.4 (-34.2 to -30.2) | -2.16 (-2.34 to -2.01) | 7.7 (3.0 to 10.2) | 1.09 (0.57 to 1.52) |
| Azamgarh | 15.7 (12.0-20.2) | 12.3 (9.8-15.1) | 11.4 (11.0-12.0) | -27.3 (-30.7 to -25.8) | -1.83 (-2.05 to -1.66) | -7.3 (-11.5 to -5.6) | -1.01 (-1.57 to -0.67) |
| Baghpat | 8.4 (6.2-11.1) | 6.4 (4.9-8.5) | 12.0 (11.4-12.8) | 45.7 (37.8 to 50.0) | 2.21 (1.95 to 2.45) | 91.0 (83.7 to 96.4) | 9.63 (9.16 to 10.20) |
| Bahraich | 15.9 (13.3-18.9) | 16.0 (13.8-18.4) | 13.4 (12.9-14.0) | -16.1 (-18.8 to -14.7) | -0.99 (-1.15 to -0.86) | -16.8 (-18.8 to -15.3) | -2.47 (-2.77 to -2.18) |
| Ballia | 16.6 (12.7-20.4) | 11.7 (9.4-14.7) | 13.4 (13.1-13.9) | -18.8 (-22.4 to -17.1) | -1.19 (-1.40 to -1.02) | 14.8 (11.8 to 17.0) | 2.03 (1.73 to 2.39) |
| Balrampur | 15.3 (12.5-18.3) | 14.2 (11.6-16.9) | 11.3 (10.9-11.8) | -26.0 (-28.2 to -24.4) | -1.71 (-1.85 to -1.56) | -20.4 (-23.6 to -19.1) | -3.10 (-3.59 to -2.81) |
| Banda | 23.0 (18.1-28.6) | 16.6 (13.3-19.6) | 16.1 (15.3-17.1) | -29.7 (-32.7 to -28.9) | -2.01 (-2.22 to -1.90) | -2.6 (-6.3 to -1.1) | -0.30 (-0.77 to -0.01) |
| Barabanki | 20.1 (16.0-24.1) | 20.7 (17.7-23.5) | 15.7 (15.1-16.4) | -22.0 (-24.8 to -20.5) | -1.41 (-1.59 to -1.27) | -24.4 (-26.1 to -23.1) | -3.78 (-4.04 to -3.50) |
| Bareilly | 12.3 (8.9-16.3) | 10.1 (8.3-12.6) | 11.9 (11.4-12.4) | -2.4 (-7.9 to -0.9) | -0.16 (-0.42 to 0.00) | 17.6 (13.5 to 20.1) | 2.39 (1.95 to 2.77) |
| Basti | 17.0 (14.0-20.6) | 15.6 (13.1-18.4) | 12.0 (11.6-12.5) | -29.5 (-31.8 to -27.6) | -1.99 (-2.14 to -1.81) | -23.3 (-25.7 to -21.6) | -3.59 (-3.98 to -3.25) |
| Bhadohi | 18.2 (14.0-22.7) | 13.3 (10.8-16.5) | 14.4 (13.3-15.4) | -20.8 (-24.4 to -19.0) | -1.34 (-1.55 to -1.16) | 8.6 (4.4 to 11.1) | 1.23 (0.76 to 1.64) |
| Bijnor | 10.3 (7.3-14.2) | 7.3 (5.6-9.3) | 12.3 (11.7-13.1) | 21.4 (15.2 to 24.3) | 1.11 (0.89 to 1.34) | 70.2 (62.8 to 74.1) | 7.86 (7.30 to 8.33) |
| Budaun | 14.0 (10.2-18.1) | 9.1 (7.2-11.4) | 12.3 (12.0-12.7) | -10.9 (-15.2 to -8.7) | -0.67 (-0.90 to -0.47) | 35.8 (29.7 to 38.9) | 4.48 (3.90 to 4.92) |
| Bulandshahr | 12.8 (9.5-16.5) | 5.3 (3.9-7.0) | 11.4 (10.9-12.1) | -10.1 (-14.6 to -8.7) | -0.62 (-0.86 to -0.47) | 121.6 (110.8 to 127.8) | 11.95 (11.31 to 12.55) |
| Chandauli | 15.7 (11.9-20.3) | 10.4 (8.2-12.9) | 12.2 (11.6-12.9) | -22.1 (-26.1 to -20.1) | -1.44 (-1.68 to -1.24) | 17.9 (13.1 to 20.3) | 2.40 (1.90 to 2.79) |
| Chitrakoot | 23.1 (18.8-28.0) | 17.3 (14.8-20.0) | 16.2 (15.5-16.9) | -30.0 (-32.7 to -28.6) | -2.03 (-2.22 to -1.89) | -7.1 (-9.3 to -5.1) | -0.94 (-1.23 to -0.59) |
| Deoria | 16.5 (13.3-20.0) | 13.0 (10.7-15.5) | 11.7 (11.3-12.0) | -29.6 (-31.9 to -27.9) | -2.00 (-2.15 to -1.82) | -10.3 (-13.3 to -9.0) | -1.45 (-1.85 to -1.18) |
| Etah | 17.0 (12.9-22.1) | 8.3 (5.8-11.2) | 10.7 (10.3-11.2) | -36.5 (-40.1 to -34.4) | -2.62 (-2.88 to -2.36) | 32.1 (24.2 to 36.0) | 3.99 (3.27 to 4.60) |
| Etawah | 16.8 (12.0-22.4) | 9.4 (6.6-12.8) | 10.8 (10.1-11.5) | -35.6 (-39.2 to -34.2) | -2.54 (-2.79 to -2.35) | 17.0 (11.3 to 20.1) | 2.20 (1.67 to 2.77) |
| Faizabad | 17.9 (14.4-21.6) | 18.6 (16.2-21.5) | 13.6 (12.9-14.3) | -24.5 (-27.6 to -22.9) | -1.60 (-1.81 to -1.44) | -27.9 (-29.4 to -26.5) | -4.42 (-4.65 to -4.13) |
| Farrukhabad | 15.9 (11.4-21.0) | 10.4 (7.9-13.4) | 11.1 (10.4-11.9) | -29.3 (-33.5 to -28.1) | -2.02 (-2.29 to -1.84) | 8.0 (3.2 to 11.4) | 1.12 (0.59 to 1.68) |
| Fatehpur | 21.9 (17.6-26.7) | 17.8 (15.1-20.9) | 16.1 (15.4-16.8) | -26.9 (-30.1 to -25.0) | -1.78 (-2.00 to -1.60) | -10.4 (-13.1 to -8.7) | -1.44 (-1.82 to -1.14) |
| Firozabad | 16.9 (13.2-21.0) | 8.6 (6.3-11.6) | 11.2 (10.7-11.8) | -33.7 (-36.7 to -32.2) | -2.35 (-2.56 to -2.18) | 31.4 (23.4 to 33.9) | 3.92 (3.16 to 4.37) |
| Gautam Buddha Nagar | 10.9 (8.3-13.8) | 6.2 (4.7-8.0) | 12.4 (11.0-14.0) | 14.8 (9.3 to 17.1) | 0.80 (0.58 to 0.98) | 104.4 (94.1 to 111.2) | 10.66 (10.02 to 11.35) |
| Ghaziabad | 10.0 (7.7-12.7) | 5.9 (4.5-7.6) | 11.8 (11.1-12.8) | 19.9 (14.3 to 23.0) | 1.07 (0.84 to 1.27) | 103.1 (93.6 to 105.9) | 10.59 (9.98 to 10.94) |
| Ghazipur | 15.3 (11.5-19.5) | 10.6 (8.4-13.0) | 11.6 (11.2-12.0) | -24.0 (-28.5 to -22.4) | -1.58 (-1.88 to -1.40) | 9.9 (5.2 to 12.6) | 1.40 (0.86 to 1.84) |
| Gonda | 17.1 (13.6-21.2) | 18.2 (15.7-21.3) | 12.6 (12.0-13.3) | -26.2 (-29.3 to -24.3) | -1.74 (-1.94 to -1.55) | -31.4 (-33.0 to -30.0) | -5.09 (-5.35 to -4.78) |
| Gorakhpur | 16.0 (13.1-19.4) | 13.1 (10.8-15.9) | 11.1 (10.8-11.5) | -30.5 (-32.9 to -29.1) | -2.07 (-2.24 to -1.92) | -15.2 (-18.0 to -13.3) | -2.23 (-2.62 to -1.85) |
| Hamirpur | 21.4 (17.3-25.9) | 15.3 (12.7-18.1) | 15.6 (14.8-16.2) | -27.4 (-29.9 to -25.9) | -1.82 (-1.99 to -1.67) | 1.5 (-2.2 to 3.5) | 0.29 (-0.17 to 0.63) |
| Hapur | 11.3 (8.6-14.4) | 5.4 (4.1-7.1) | 11.6 (11.0-12.2) | 3.7 (-1.2 to 5.5) | 0.21 (-0.01 to 0.37) | 116.3 (107.0 to 122.2) | 11.58 (11.03 to 12.15) |
| Hardoi | 18.4 (14.4-23.1) | 13.9 (11.8-16.2) | 12.2 (11.8-12.6) | -33.9 (-36.5 to -32.0) | -2.36 (-2.55 to -2.16) | -12.9 (-15.2 to -11.0) | -1.84 (-2.17 to -1.49) |
| Hathras | 15.2 (11.5-20.0) | 6.6 (4.8-8.9) | 10.7 (10.3-11.2) | -29.1 (-32.4 to -26.8) | -1.98 (-2.19 to -1.74) | 64.8 (54.6 to 67.8) | 7.31 (6.52 to 7.76) |
| Jalaun | 19.9 (15.6-24.9) | 14.2 (11.5-17.6) | 15.3 (14.5-16.4) | -22.9 (-26.0 to -21.3) | -1.49 (-1.68 to -1.33) | 8.1 (4.0 to 10.2) | 1.17 (0.70 to 1.52) |
| Jaunpur | 17.4 (14.1-20.9) | 13.8 (11.7-16.3) | 13.4 (13.0-13.8) | -23.1 (-25.9 to -21.6) | -1.49 (-1.67 to -1.35) | -3.3 (-6.0 to -1.5) | -0.38 (-0.73 to -0.07) |
| Jhansi | 22.5 (18.4-26.8) | 17.7 (15.1-20.9) | 17.2 (16.4-18.0) | -23.8 (-26.7 to -22.5) | -1.54 (-1.73 to -1.41) | -2.9 (-4.9 to -1.4) | -0.32 (-0.56 to -0.05) |
| Kannauj | 17.1 (12.5-21.7) | 11.6 (9.1-14.8) | 11.8 (11.2-12.3) | -31.0 (-34.2 to -29.0) | -2.13 (-2.35 to -1.92) | 2.3 (-2.4 to 5.0) | 0.36 (-0.20 to 0.83) |
| Kanpur Dehat | 19.4 (14.1-25.1) | 13.8 (10.6-17.5) | 13.8 (13.1-14.8) | -28.1 (-32.0 to -26.1) | -1.90 (-2.16 to -1.69) | 1.1 (-2.9 to 3.2) | 0.18 (-0.28 to 0.59) |
| Kanpur Nagar | 19.0 (14.4-25.1) | 14.9 (11.8-18.2) | 13.2 (12.2-14.4) | -30.3 (-33.1 to -28.7) | -2.08 (-2.25 to -1.89) | -11.1 (-14.6 to -9.4) | -1.62 (-2.06 to -1.24) |
| Kasganj | 16.5 (12.1-21.7) | 8.6 (6.1-11.2) | 11.2 (10.7-11.8) | -31.3 (-35.9 to -29.5) | -2.17 (-2.49 to -1.96) | 33.6 (26.3 to 36.4) | 4.16 (3.51 to 4.65) |
| Kaushambi | 21.7 (16.9-26.8) | 17.4 (15.0-20.1) | 16.0 (15.0-17.0) | -26.2 (-29.5 to -24.6) | -1.74 (-1.96 to -1.57) | -8.8 (-10.9 to -6.8) | -1.20 (-1.48 to -0.85) |
| Khushinagar | 16.7 (12.7-20.7) | 14.2 (11.3-17.3) | 12.1 (11.5-12.9) | -27.5 (-30.1 to -25.6) | -1.83 (-2.01 to -1.65) | -14.3 (-17.2 to -12.7) | -2.10 (-2.49 to -1.76) |
| Lakhimpur Kheri | 15.7 (12.8-18.7) | 15.8 (13.7-18.2) | 15.3 (14.6-16.1) | -2.1 (-5.6 to 0.0) | -0.10 (-0.27 to 0.06) | -3.3 (-5.7 to -1.5) | -0.38 (-0.69 to -0.07) |
| Lalitpur | 23.6 (18.5-28.6) | 19.3 (15.3-23.4) | 17.0 (16.0-18.2) | -27.8 (-31.0 to -25.8) | -1.86 (-2.07 to -1.66) | -11.9 (-15.6 to -10.4) | -1.72 (-2.23 to -1.39) |
| Lucknow | 21.1 (17.0-26.1) | 19.7 (16.4-23.2) | 16.1 (15.1-17.2) | -23.8 (-26.6 to -22.4) | -1.55 (-1.73 to -1.40) | -18.5 (-20.8 to -16.5) | -2.78 (-3.11 to -2.37) |
| Maharajganj | 15.3 (12.5-18.5) | 14.0 (11.5-16.7) | 11.8 (11.2-12.3) | -23.1 (-25.7 to -21.5) | -1.49 (-1.65 to -1.34) | -16.3 (-19.4 to -15.2) | -2.42 (-2.86 to -2.16) |
| Mahoba | 22.1 (17.9-26.3) | 16.9 (14.4-19.8) | 15.4 (14.6-16.3) | -30.5 (-33.1 to -29.0) | -2.07 (-2.25 to -1.91) | -9.2 (-11.4 to -7.2) | -1.26 (-1.55 to -0.91) |
| Mainpuri | 16.8 (12.8-21.2) | 9.0 (6.6-11.9) | 10.5 (10.2-11.0) | -37.2 (-40.5 to -35.5) | -2.66 (-2.91 to -2.46) | 18.7 (13.2 to 21.8) | 2.47 (1.91 to 2.97) |
| Mathura | 12.8 (9.3-16.7) | 6.1 (4.5-8.0) | 11.6 (10.9-12.4) | -8.6 (-13.3 to -6.3) | -0.53 (-0.77 to -0.32) | 92.2 (82.3 to 96.4) | 9.70 (9.04 to 10.20) |
| Mau | 15.6 (11.5-20.3) | 11.4 (8.7-14.6) | 11.2 (10.6-11.9) | -27.8 (-31.8 to -25.7) | -1.88 (-2.15 to -1.65) | -0.9 (-6.7 to 2.0) | -0.11 (-0.84 to 0.42) |
| Meerut | 9.7 (7.5-12.5) | 6.1 (4.8-7.7) | 11.9 (11.4-12.3) | 23.7 (17.7 to 27.2) | 1.25 (1.01 to 1.47) | 95.4 (88.2 to 99.4) | 10.02 (9.54 to 10.44) |
| Mirzapur | 18.6 (14.6-22.6) | 13.5 (11.1-16.7) | 15.5 (14.9-16.2) | -16.7 (-20.0 to -14.8) | -1.04 (-1.23 to -0.87) | 14.5 (10.2 to 16.8) | 2.01 (1.53 to 2.37) |
| Moradabad | 11.1 (8.0-14.6) | 7.3 (5.8-9.0) | 11.7 (11.2-12.2) | 6.8 (0.3 to 9.4) | 0.37 (0.08 to 0.58) | 60.6 (54.9 to 65.1) | 7.00 (6.55 to 7.51) |
| Muzaffarnagar | 8.7 (6.6-11.3) | 6.6 (5.2-8.5) | 11.8 (11.4-12.3) | 37.8 (30.2 to 41.3) | 1.89 (1.61 to 2.10) | 82.0 (74.8 to 88.0) | 8.90 (8.39 to 9.52) |
| Pilibhit | 13.2 (10.3-16.6) | 13.0 (10.8-15.5) | 14.5 (13.8-15.2) | 10.8 (5.7 to 13.1) | 0.61 (0.38 to 0.78) | 11.7 (8.6 to 14.1) | 1.66 (1.31 to 2.03) |
| Pratapgarh | 20.6 (16.4-25.0) | 17.2 (15.1-19.7) | 15.4 (14.8-16.0) | -25.4 (-27.8 to -24.5) | -1.67 (-1.82 to -1.56) | -11.3 (-13.4 to -9.9) | -1.58 (-1.87 to -1.32) |
| Rae Bareli | 22.2 (17.4-27.4) | 20.6 (17.2-24.1) | 18.6 (17.7-19.5) | -16.3 (-19.7 to -15.3) | -1.01 (-1.21 to -0.91) | -10.2 (-12.5 to -8.2) | -1.42 (-1.73 to -1.07) |
| Rampur | 11.5 (8.5-15.2) | 8.0 (6.4-9.7) | 10.7 (10.4-11.1) | -5.7 (-11.0 to -3.6) | -0.35 (-0.62 to -0.15) | 35.2 (30.8 to 37.8) | 4.43 (4.02 to 4.79) |
| Saharanpur | 9.0 (6.4-12.5) | 7.2 (5.5-9.6) | 12.9 (12.3-13.5) | 45.7 (37.9 to 50.4) | 2.20 (1.95 to 2.47) | 80.8 (73.3 to 87.8) | 8.76 (8.26 to 9.50) |
| Sambhal | 12.1 (8.7-16.4) | 6.9 (5.3-8.9) | 12.7 (12.0-13.4) | 6.1 (0.0 to 8.8) | 0.33 (0.06 to 0.55) | 86.6 (78.7 to 91.2) | 9.27 (8.74 to 9.78) |
| Sant Kabir Nagar | 16.3 (12.6-20.3) | 13.5 (10.7-16.6) | 11.1 (10.6-11.6) | -32.1 (-34.7 to -30.3) | -2.22 (-2.39 to -2.02) | -18.2 (-21.9 to -15.8) | -2.75 (-3.29 to -2.27) |
| Shahjahanpur | 14.4 (10.8-18.5) | 12.6 (10.4-15.4) | 13.0 (12.6-13.5) | -9.0 (-13.2 to -6.6) | -0.55 (-0.77 to -0.34) | 2.8 (-0.1 to 5.0) | 0.46 (0.13 to 0.84) |
| Shamli | 7.9 (5.9-10.5) | 6.6 (5.0-8.7) | 12.6 (12.0-13.2) | 61.8 (52.5 to 66.3) | 2.84 (2.55 to 3.07) | 93.3 (84.0 to 99.9) | 9.82 (9.19 to 10.48) |
| Shravasti | 15.3 (12.2-19.0) | 15.0 (12.1-18.3) | 12.3 (11.7-13.0) | -19.8 (-22.6 to -18.1) | -1.26 (-1.42 to -1.10) | -18.3 (-21.6 to -17.0) | -2.76 (-3.24 to -2.46) |
| Siddharth Nagar | 15.9 (12.7-19.4) | 13.8 (11.1-16.8) | 10.9 (10.6-11.3) | -31.9 (-34.3 to -30.4) | -2.19 (-2.35 to -2.03) | -21.4 (-24.1 to -19.2) | -3.28 (-3.69 to -2.83) |
| Sitapur | 19.7 (15.3-25.0) | 16.9 (14.4-19.5) | 13.1 (12.4-13.8) | -33.3 (-36.3 to -31.2) | -2.32 (-2.53 to -2.10) | -22.9 (-24.8 to -21.3) | -3.53 (-3.80 to -3.20) |
| Sonbhadra | 19.3 (14.1-24.9) | 13.1 (9.9-16.9) | 16.3 (15.3-17.3) | -14.4 (-19.6 to -12.3) | -0.91 (-1.20 to -0.70) | 26.2 (19.5 to 30.0) | 3.35 (2.70 to 3.94) |
| Sultanpur | 18.2 (14.1-22.8) | 16.9 (14.3-20.0) | 14.1 (13.3-15.0) | -22.3 (-25.3 to -20.5) | -1.45 (-1.62 to -1.27) | -16.7 (-19.5 to -15.2) | -2.48 (-2.88 to -2.16) |
| Unnao | 20.6 (16.4-25.2) | 16.7 (14.0-19.5) | 14.1 (13.6-14.6) | -31.5 (-33.8 to -29.5) | -2.15 (-2.31 to -1.95) | -16.0 (-18.7 to -14.5) | -2.35 (-2.74 to -2.05) |
| Varanasi | 16.5 (12.8-20.9) | 11.6 (9.4-14.5) | 13.5 (12.5-14.5) | -18.0 (-21.9 to -16.3) | -1.15 (-1.37 to -0.97) | 16.5 (12.6 to 20.1) | 2.24 (1.84 to 2.77) |
| **Rajasthan** | **20.0 (18.8-21.1)** | **16.5 (15.8-17.2)** | **17.5 (16.9-18.1)** | **-13.4 (-14.3 to -12.5)** | **-0.78 (-0.84 to -0.71)** | **5.4 (3.5 to 6.6)** | **0.84 (-0.02 to 1.71)** |
| Ajmer | 22.1 (17.3-27.3) | 16.3 (12.2-20.6) | 20.4 (19.2-21.7) | -7.3 (-11.3 to -5.3) | -0.42 (-0.63 to -0.26) | 27.0 (21.2 to 30.1) | 3.46 (2.91 to 3.95) |
| Alwar | 12.4 (10.0-15.3) | 12.4 (9.7-15.2) | 15.7 (15.2-16.5) | 27.1 (22.5 to 30.2) | 1.43 (1.25 to 1.61) | 28.1 (22.6 to 30.0) | 3.62 (3.07 to 3.94) |
| Banswara | 25.3 (21.0-29.9) | 29.4 (24.5-34.7) | 22.4 (21.5-23.6) | -11.5 (-14.5 to -9.7) | -0.68 (-0.85 to -0.54) | -24.1 (-26.8 to -23.5) | -3.73 (-4.16 to -3.58) |
| Baran | 24.2 (19.4-29.7) | 16.2 (13.0-20.6) | 18.2 (17.1-19.4) | -25.0 (-27.6 to -23.1) | -1.64 (-1.80 to -1.46) | 12.7 (8.5 to 15.6) | 1.75 (1.31 to 2.22) |
| Barmer | 22.7 (17.7-28.2) | 20.8 (15.7-27.3) | 20.1 (18.6-21.4) | -10.7 (-15.1 to -9.1) | -0.65 (-0.89 to -0.50) | -2.3 (-8.1 to 0.2) | -0.31 (-1.05 to 0.17) |
| Bharatpur | 14.6 (11.0-18.8) | 11.3 (8.7-14.4) | 15.9 (15.1-17.0) | 9.9 (4.7 to 12.2) | 0.56 (0.33 to 0.73) | 41.9 (35.4 to 45.0) | 5.11 (4.54 to 5.55) |
| Bhilwara | 21.8 (17.3-26.6) | 17.0 (13.5-21.5) | 20.0 (18.7-21.4) | -7.9 (-11.3 to -6.1) | -0.46 (-0.64 to -0.31) | 18.1 (13.2 to 21.3) | 2.43 (1.91 to 2.91) |
| Bikaner | 21.3 (15.9-27.1) | 14.1 (10.5-18.1) | 16.8 (15.9-18.1) | -20.6 (-24.0 to -19.4) | -1.33 (-1.52 to -1.19) | 21.1 (14.4 to 23.0) | 2.75 (2.06 to 3.12) |
| Bundi | 22.8 (18.8-27.7) | 16.0 (11.9-20.6) | 18.8 (17.5-20.0) | -17.7 (-20.2 to -15.5) | -1.10 (-1.25 to -0.92) | 18.9 (12.5 to 22.4) | 2.49 (1.83 to 3.05) |
| Chittorgarh | 22.2 (17.6-26.9) | 18.9 (14.9-23.0) | 18.9 (17.7-20.2) | -14.7 (-17.9 to -12.7) | -0.90 (-1.08 to -0.73) | 0.1 (-3.7 to 1.7) | 0.08 (-0.39 to 0.38) |
| Churu | 16.0 (12.5-20.2) | 16.4 (12.9-20.6) | 17.8 (16.6-18.8) | 11.9 (7.7 to 15.3) | 0.67 (0.49 to 0.89) | 9.2 (4.6 to 11.6) | 1.29 (0.78 to 1.71) |
| Dausa | 15.2 (11.9-19.7) | 9.8 (6.9-13.4) | 13.2 (12.3-14.1) | -12.8 (-17.2 to -10.6) | -0.79 (-1.04 to -0.59) | 37.4 (29.8 to 41.8) | 4.56 (3.91 to 5.22) |
| Dholpur | 20.6 (16.7-25.2) | 14.8 (11.3-18.7) | 16.9 (16.1-17.6) | -18.0 (-21.0 to -16.3) | -1.13 (-1.30 to -0.97) | 15.0 (9.9 to 17.2) | 2.02 (1.49 to 2.41) |
| Dungarpur | 24.3 (19.3-29.8) | 26.6 (21.4-31.8) | 23.6 (22.1-25.1) | -2.8 (-6.7 to -0.2) | -0.15 (-0.35 to 0.05) | -11.3 (-15.1 to -10.0) | -1.61 (-2.15 to -1.34) |
| Hanumangarh | 13.2 (9.5-17.7) | 19.5 (14.9-24.6) | 18.5 (17.5-19.7) | 42.7 (34.2 to 46.2) | 2.08 (1.79 to 2.30) | -4.4 (-8.7 to -2.7) | -0.60 (-1.14 to -0.25) |
| Jaipur | 17.2 (13.6-20.8) | 11.3 (8.7-14.7) | 12.6 (11.8-13.3) | -27.1 (-29.8 to -25.5) | -1.80 (-1.98 to -1.64) | 12.1 (7.0 to 16.3) | 1.63 (1.10 to 2.30) |
| Jaisalmer | 21.1 (14.1-30.0) | 17.3 (12.6-22.8) | 17.7 (16.0-19.4) | -14.1 (-20.6 to -12.1) | -0.93 (-1.28 to -0.69) | 4.0 (-1.8 to 7.4) | 0.52 (-0.12 to 1.16) |
| Jalore | 24.9 (20.0-30.5) | 23.7 (18.8-29.0) | 18.9 (17.6-20.2) | -24.3 (-27.3 to -22.5) | -1.58 (-1.78 to -1.42) | -20.4 (-23.7 to -18.1) | -3.11 (-3.60 to -2.64) |
| Jhalawar | 26.5 (21.8-31.4) | 24.0 (20.0-28.1) | 20.2 (19.0-21.5) | -24.1 (-27.1 to -22.9) | -1.57 (-1.76 to -1.45) | -16.1 (-19.0 to -14.2) | -2.36 (-2.79 to -2.00) |
| Jhunjhunu | 12.7 (9.9-16.1) | 14.3 (11.0-18.2) | 15.3 (14.3-16.3) | 21.2 (15.6 to 23.5) | 1.14 (0.91 to 1.30) | 7.9 (4.1 to 10.9) | 1.11 (0.72 to 1.61) |
| Jodhpur | 23.0 (17.4-29.4) | 14.2 (11.0-18.0) | 17.0 (16.0-18.1) | -25.6 (-28.9 to -23.8) | -1.70 (-1.91 to -1.51) | 20.6 (16.0 to 22.6) | 2.72 (2.27 to 3.07) |
| Karauli | 18.1 (14.4-22.9) | 12.2 (9.2-15.8) | 16.2 (15.3-17.1) | -10.0 (-13.2 to -8.0) | -0.59 (-0.76 to -0.43) | 34.7 (28.5 to 38.2) | 4.33 (3.76 to 4.84) |
| Kota | 22.5 (18.3-27.3) | 16.6 (13.1-20.9) | 18.6 (17.4-19.9) | -17.5 (-20.4 to -15.5) | -1.09 (-1.26 to -0.92) | 12.5 (7.0 to 15.1) | 1.71 (1.10 to 2.16) |
| Nagaur | 22.6 (18.4-27.6) | 13.6 (10.6-17.4) | 16.1 (15.1-17.1) | -29.2 (-31.7 to -27.5) | -1.96 (-2.13 to -1.80) | 19.3 (14.0 to 22.6) | 2.55 (2.01 to 3.07) |
| Pali | 25.2 (20.1-30.3) | 19.1 (15.1-23.7) | 18.7 (17.4-20.0) | -25.6 (-29.0 to -24.1) | -1.69 (-1.91 to -1.53) | -1.7 (-5.6 to 0.0) | -0.19 (-0.66 to 0.15) |
| Pratapgarh | 24.4 (19.8-29.3) | 27.0 (21.8-32.3) | 21.5 (20.1-23.1) | -11.6 (-15.2 to -9.4) | -0.69 (-0.90 to -0.52) | -20.5 (-23.6 to -18.6) | -3.12 (-3.60 to -2.73) |
| Rajsamand | 22.8 (18.3-27.5) | 18.1 (14.5-22.5) | 18.7 (17.5-19.9) | -18.0 (-21.7 to -16.3) | -1.13 (-1.35 to -0.97) | 3.3 (-0.9 to 6.2) | 0.51 (0.01 to 1.00) |
| Sawai Madhopur | 18.7 (14.9-23.3) | 11.1 (8.0-14.6) | 15.3 (14.5-16.2) | -18.1 (-20.9 to -16.9) | -1.13 (-1.30 to -1.01) | 40.0 (32.4 to 43.9) | 4.86 (4.21 to 5.44) |
| Sikar | 16.2 (12.8-19.5) | 14.5 (11.1-19.1) | 15.2 (14.4-16.1) | -5.9 (-9.4 to -3.6) | -0.34 (-0.52 to -0.16) | 6.0 (1.8 to 8.5) | 0.84 (0.40 to 1.31) |
| Sirohi | 25.8 (19.6-32.6) | 27.1 (21.7-32.9) | 23.2 (21.8-24.9) | -9.6 (-14.3 to -7.4) | -0.58 (-0.83 to -0.39) | -14.2 (-17.5 to -12.7) | -2.09 (-2.54 to -1.76) |
| Sri Ganganagar | 16.4 (11.4-22.4) | 17.3 (13.7-20.8) | 15.3 (14.0-16.8) | -5.0 (-11.5 to -2.5) | -0.33 (-0.65 to -0.09) | -11.8 (-15.6 to -9.5) | -1.71 (-2.22 to -1.26) |
| Tonk | 21.1 (17.3-25.7) | 13.3 (10.0-17.6) | 17.0 (15.9-18.2) | -19.3 (-21.8 to -16.8) | -1.22 (-1.36 to -1.00) | 29.9 (22.7 to 34.8) | 3.77 (3.09 to 4.46) |
| Udaipur | 22.7 (18.8-26.7) | 22.8 (18.5-27.2) | 22.0 (20.8-23.5) | -3.0 (-7.5 to -1.1) | -0.15 (-0.39 to -0.01) | -3.2 (-6.6 to -1.6) | -0.40 (-0.81 to -0.09) |
| **Chhattisgarh** | **19.8 (17.7-21.8)** | **15.6 (14.8-16.4)** | **15.8 (15.2-16.5)** | **-20.5 (-22.7 to -20.0)** | **-1.29 (-1.43 to -1.23)** | **1.1 (-0.7 to 2.9)** | **0.23 (-0.73 to 1.24)** |
| Balod | 19.9 (13.9-26.4) | 16.5 (12.8-20.4) | 14.4 (13.8-15.1) | -26.7 (-31.1 to -25.3) | -1.81 (-2.09 to -1.63) | -12.5 (-16.1 to -10.7) | -1.82 (-2.31 to -1.44) |
| Baloda Bazar | 19.2 (14.8-24.3) | 15.0 (11.8-18.5) | 15.5 (14.8-16.1) | -19.0 (-22.6 to -17.3) | -1.21 (-1.42 to -1.04) | 3.6 (-0.7 to 5.7) | 0.56 (0.04 to 0.94) |
| Balrampur | 20.9 (15.4-26.7) | 15.2 (11.9-19.3) | 18.1 (17.4-19.0) | -12.7 (-17.2 to -10.7) | -0.78 (-1.03 to -0.60) | 20.3 (15.5 to 23.7) | 2.69 (2.20 to 3.20) |
| Bastar | 19.4 (15.0-24.9) | 17.3 (13.0-22.2) | 19.6 (18.5-20.5) | 1.6 (-3.1 to 4.5) | 0.10 (-0.12 to 0.32) | 14.6 (9.3 to 17.3) | 1.96 (1.41 to 2.42) |
| Bemetara | 19.2 (14.7-24.1) | 15.0 (11.9-18.5) | 14.5 (13.9-15.1) | -24.2 (-28.1 to -23.0) | -1.59 (-1.84 to -1.45) | -2.7 (-6.9 to -0.3) | -0.34 (-0.87 to 0.11) |
| Bijapur | 15.7 (10.8-22.8) | 18.3 (13.6-23.9) | 16.5 (15.5-17.7) | 7.9 (-0.1 to 12.2) | 0.40 (0.05 to 0.73) | -8.8 (-13.3 to -6.2) | -1.30 (-1.86 to -0.76) |
| Bilaspur | 20.7 (16.3-25.8) | 17.3 (13.6-21.9) | 18.2 (17.2-19.2) | -11.8 (-15.3 to -9.2) | -0.71 (-0.91 to -0.50) | 5.7 (2.1 to 9.1) | 0.83 (0.43 to 1.38) |
| Dantewada | 18.3 (12.9-24.7) | 18.4 (13.6-24.5) | 18.8 (17.6-19.7) | 4.3 (-1.6 to 6.4) | 0.22 (-0.04 to 0.42) | 3.6 (-1.8 to 6.5) | 0.48 (-0.12 to 1.03) |
| Dhamtari | 17.9 (12.5-23.6) | 13.6 (10.4-16.9) | 13.9 (13.2-14.6) | -21.2 (-25.9 to -18.8) | -1.39 (-1.67 to -1.14) | 3.1 (-1.3 to 5.5) | 0.46 (-0.04 to 0.90) |
| Durg | 18.5 (13.5-24.0) | 14.1 (11.0-17.4) | 12.5 (11.8-13.2) | -32.0 (-36.2 to -29.7) | -2.23 (-2.52 to -1.97) | -11.3 (-14.4 to -8.9) | -1.64 (-2.03 to -1.17) |
| Gariaband | 18.6 (14.6-23.1) | 13.2 (10.6-16.1) | 14.2 (13.7-14.8) | -23.6 (-26.8 to -22.2) | -1.54 (-1.74 to -1.39) | 7.9 (3.2 to 9.9) | 1.13 (0.60 to 1.49) |
| Janjgir-Champa | 19.7 (14.8-25.0) | 15.1 (11.5-19.5) | 15.9 (15.1-16.7) | -18.5 (-22.6 to -16.6) | -1.19 (-1.42 to -0.99) | 6.2 (1.8 to 9.3) | 0.89 (0.40 to 1.41) |
| Jashpur | 21.4 (16.9-26.6) | 15.5 (12.3-19.3) | 16.7 (16.0-17.4) | -21.9 (-25.3 to -20.9) | -1.41 (-1.62 to -1.29) | 7.9 (4.0 to 10.7) | 1.14 (0.70 to 1.59) |
| Kabirdham | 19.9 (14.4-25.6) | 16.5 (12.4-21.0) | 16.1 (15.1-17.1) | -18.2 (-22.8 to -16.3) | -1.18 (-1.44 to -0.97) | -1.6 (-6.6 to 1.1) | -0.21 (-0.82 to 0.29) |
| Kondagaon | 19.2 (13.9-25.2) | 17.7 (13.3-22.5) | 18.0 (17.1-18.8) | -5.1 (-10.0 to -1.8) | -0.31 (-0.55 to -0.05) | 2.1 (-3.2 to 4.2) | 0.31 (-0.31 to 0.73) |
| Korba | 21.1 (16.1-26.0) | 15.6 (12.1-19.9) | 16.6 (15.9-17.3) | -21.2 (-25.6 to -19.9) | -1.37 (-1.65 to -1.22) | 6.8 (1.5 to 9.3) | 0.97 (0.35 to 1.41) |
| Korea | 22.1 (16.7-28.1) | 16.0 (12.4-20.1) | 16.6 (15.8-17.5) | -24.7 (-28.1 to -22.9) | -1.63 (-1.84 to -1.44) | 4.5 (0.4 to 7.3) | 0.66 (0.20 to 1.14) |
| Mahasamund | 19.1 (14.8-24.0) | 13.9 (10.8-17.3) | 14.6 (13.9-15.3) | -23.8 (-27.4 to -22.5) | -1.56 (-1.79 to -1.41) | 5.3 (1.1 to 8.1) | 0.78 (0.30 to 1.25) |
| Mungeli | 19.7 (15.0-24.7) | 16.7 (13.2-21.6) | 17.3 (16.4-18.3) | -11.5 (-15.7 to -9.4) | -0.70 (-0.93 to -0.51) | 4.0 (-0.5 to 7.2) | 0.59 (0.07 to 1.13) |
| Narayanpur | 18.7 (12.5-26.3) | 19.9 (14.4-25.8) | 18.2 (16.9-19.6) | 0.5 (-6.8 to 3.2) | -0.03 (-0.35 to 0.24) | -7.1 (-13.4 to -5.0) | -1.07 (-1.88 to -0.58) |
| North Bastar Kanker | 19.8 (13.5-26.5) | 17.2 (12.8-21.6) | 16.5 (15.6-17.5) | -14.8 (-20.8 to -13.9) | -0.96 (-1.29 to -0.81) | -3.2 (-8.5 to -1.6) | -0.45 (-1.11 to -0.08) |
| Raigarh | 19.9 (15.5-24.7) | 14.1 (11.2-17.8) | 14.5 (14.0-15.1) | -27.2 (-29.8 to -25.8) | -1.82 (-1.98 to -1.66) | 3.0 (-1.2 to 5.9) | 0.46 (-0.03 to 0.95) |
| Raipur | 17.8 (12.5-24.0) | 12.6 (9.3-16.3) | 12.3 (11.5-13.1) | -30.1 (-34.9 to -28.5) | -2.09 (-2.41 to -1.87) | -1.9 (-6.3 to 0.4) | -0.26 (-0.78 to 0.20) |
| Rajnandgaon | 21.1 (15.3-27.4) | 18.0 (14.5-21.6) | 14.9 (14.2-15.8) | -28.5 (-32.9 to -27.4) | -1.94 (-2.23 to -1.79) | -17.2 (-19.9 to -14.7) | -2.56 (-2.96 to -2.09) |
| Sukma | 17.6 (12.6-23.4) | 17.2 (12.9-22.4) | 19.5 (18.2-20.5) | 12.8 (7.1 to 16.7) | 0.69 (0.46 to 0.96) | 14.8 (9.5 to 18.3) | 1.97 (1.44 to 2.55) |
| Surajpur | 22.5 (17.2-28.1) | 16.2 (12.7-20.2) | 18.7 (18.0-19.5) | -16.7 (-20.5 to -15.8) | -1.05 (-1.27 to -0.94) | 16.2 (11.6 to 19.0) | 2.19 (1.70 to 2.64) |
| Surguja | 22.6 (16.4-29.9) | 15.3 (11.5-19.9) | 18.1 (17.0-19.2) | -19.5 (-23.9 to -17.9) | -1.26 (-1.52 to -1.09) | 19.4 (14.4 to 22.0) | 2.56 (2.07 to 3.00) |
| **Odisha** | **19.6 (18.7-20.6)** | **18.8 (18.1-19.5)** | **17.3 (16.7-18.0)** | **-12.6 (-13.6 to -11.4)** | **-0.73 (-0.79 to -0.64)** | **-8.5 (-9.8 to -7.6)** | **-1.14 (-1.98 to -0.26)** |
| Angul | 21.2 (17.0-25.5) | 20.5 (16.0-25.1) | 18.3 (17.1-19.5) | -13.8 (-17.1 to -11.8) | -0.84 (-1.02 to -0.67) | -10.6 (-15.0 to -8.9) | -1.52 (-2.12 to -1.16) |
| Balangir | 21.1 (17.2-24.9) | 21.2 (17.7-25.2) | 19.9 (18.7-21.1) | -6.2 (-9.3 to -4.3) | -0.34 (-0.51 to -0.20) | -6.7 (-9.3 to -4.9) | -0.90 (-1.23 to -0.57) |
| Balasore | 20.3 (17.0-23.8) | 23.0 (19.4-27.2) | 15.7 (14.7-16.7) | -23.2 (-25.7 to -22.2) | -1.50 (-1.65 to -1.39) | -32.2 (-34.7 to -31.0) | -5.27 (-5.70 to -4.97) |
| Bargarh | 20.5 (17.1-24.3) | 21.3 (17.7-25.7) | 19.8 (19.0-20.7) | -3.5 (-6.2 to -2.0) | -0.18 (-0.32 to -0.06) | -7.0 (-10.0 to -5.8) | -0.94 (-1.33 to -0.70) |
| Bhadrak | 18.8 (15.2-23.0) | 17.7 (14.4-21.6) | 13.0 (12.2-13.8) | -31.2 (-33.4 to -29.8) | -2.13 (-2.28 to -1.98) | -27.0 (-29.8 to -25.5) | -4.29 (-4.74 to -3.94) |
| Boudh | 19.8 (16.0-23.7) | 20.4 (16.6-24.6) | 18.4 (17.4-19.6) | -6.7 (-10.0 to -4.9) | -0.38 (-0.55 to -0.23) | -9.8 (-12.3 to -8.4) | -1.37 (-1.71 to -1.09) |
| Cuttack | 18.1 (15.3-21.7) | 14.7 (11.5-18.6) | 13.4 (12.4-14.3) | -26.2 (-28.7 to -24.4) | -1.73 (-1.89 to -1.56) | -7.9 (-12.0 to -5.4) | -1.12 (-1.66 to -0.64) |
| Deogarh | 21.8 (17.8-25.9) | 21.9 (17.4-27.2) | 18.9 (17.7-20.1) | -13.4 (-16.7 to -12.0) | -0.81 (-1.00 to -0.68) | -13.6 (-17.2 to -11.6) | -1.99 (-2.50 to -1.59) |
| Dhenkanal | 20.7 (17.4-24.0) | 17.3 (13.8-21.0) | 15.6 (14.8-16.5) | -24.9 (-27.8 to -23.4) | -1.62 (-1.82 to -1.48) | -9.5 (-13.6 to -7.7) | -1.35 (-1.90 to -0.98) |
| Gajapati | 19.0 (14.3-23.7) | 14.0 (10.0-19.2) | 17.5 (15.9-19.4) | -7.1 (-11.2 to -5.5) | -0.43 (-0.63 to -0.27) | 28.0 (20.3 to 30.7) | 3.51 (2.80 to 4.01) |
| Ganjam | 16.2 (13.0-19.7) | 12.6 (9.4-15.7) | 14.8 (13.5-16.2) | -8.7 (-12.5 to -6.4) | -0.51 (-0.72 to -0.33) | 18.6 (13.2 to 21.6) | 2.45 (1.91 to 2.96) |
| Jagatsinghapur | 17.1 (13.5-21.1) | 15.4 (11.8-19.8) | 13.7 (12.7-14.8) | -20.3 (-23.2 to -18.7) | -1.30 (-1.47 to -1.14) | -10.9 (-14.5 to -8.9) | -1.59 (-2.04 to -1.17) |
| Jajapur | 19.1 (15.6-23.3) | 15.1 (12.0-18.4) | 12.6 (11.8-13.6) | -34.4 (-37.3 to -33.1) | -2.40 (-2.62 to -2.25) | -16.7 (-20.5 to -15.5) | -2.51 (-3.04 to -2.22) |
| Jharsuguda | 20.4 (16.4-25.0) | 21.4 (17.1-26.4) | 18.8 (17.6-20.3) | -7.5 (-11.0 to -5.8) | -0.43 (-0.62 to -0.29) | -12.0 (-15.9 to -9.7) | -1.74 (-2.28 to -1.30) |
| Kalahandi | 21.2 (17.7-25.0) | 20.9 (17.3-25.1) | 21.0 (19.6-22.2) | -1.5 (-4.7 to 0.9) | -0.05 (-0.22 to 0.11) | 0.5 (-2.5 to 3.2) | 0.14 (-0.21 to 0.59) |
| Kandhamal | 19.4 (15.3-23.4) | 19.6 (15.7-23.7) | 19.4 (18.2-20.8) | 0.1 (-3.2 to 2.3) | 0.03 (-0.13 to 0.19) | -0.9 (-4.3 to 1.6) | -0.07 (-0.47 to 0.36) |
| Kendrapara | 17.7 (13.5-22.9) | 15.7 (11.8-20.3) | 12.7 (11.5-13.9) | -28.1 (-31.9 to -26.3) | -1.91 (-2.15 to -1.70) | -18.9 (-22.9 to -17.5) | -2.93 (-3.46 to -2.55) |
| Kendujhar | 22.4 (18.9-26.1) | 22.5 (18.7-26.4) | 18.8 (17.6-20.0) | -16.5 (-19.2 to -14.8) | -1.01 (-1.18 to -0.87) | -16.9 (-19.4 to -15.7) | -2.50 (-2.86 to -2.25) |
| Khordha | 17.1 (13.8-20.8) | 13.5 (10.0-17.6) | 13.1 (11.9-14.6) | -23.5 (-26.6 to -21.4) | -1.53 (-1.73 to -1.33) | -2.3 (-6.9 to 1.1) | -0.34 (-0.87 to 0.29) |
| Koraput | 21.7 (18.3-25.9) | 17.0 (12.4-22.6) | 21.9 (20.7-23.3) | 1.0 (-1.9 to 3.2) | 0.09 (-0.05 to 0.24) | 31.0 (25.2 to 34.3) | 3.90 (3.37 to 4.42) |
| Malkangiri | 20.6 (16.1-26.0) | 20.8 (15.3-27.4) | 27.4 (25.8-29.0) | 34.4 (29.4 to 38.8) | 1.75 (1.57 to 1.99) | 34.0 (26.9 to 37.4) | 4.23 (3.57 to 4.75) |
| Mayurbhanj | 22.6 (19.7-25.8) | 27.3 (23.3-31.6) | 20.8 (20.1-21.9) | -8.3 (-10.2 to -7.0) | -0.47 (-0.56 to -0.36) | -24.3 (-26.2 to -23.1) | -3.76 (-4.07 to -3.51) |
| Nabarangpur | 21.0 (16.8-25.4) | 22.0 (16.5-28.6) | 24.0 (22.8-25.3) | 14.7 (10.9 to 16.8) | 0.83 (0.66 to 0.97) | 10.1 (5.3 to 12.9) | 1.39 (0.87 to 1.88) |
| Nayagarh | 17.7 (14.4-22.1) | 15.9 (12.4-19.5) | 15.6 (14.5-16.7) | -12.0 (-15.5 to -9.9) | -0.72 (-0.92 to -0.54) | -1.9 (-6.1 to 0.1) | -0.24 (-0.75 to 0.16) |
| Nuapada | 20.7 (15.5-26.1) | 20.4 (15.7-25.3) | 21.2 (19.6-23.0) | 3.1 (-2.3 to 5.4) | 0.18 (-0.08 to 0.37) | 4.6 (-0.5 to 8.1) | 0.67 (0.07 to 1.26) |
| Puri | 16.1 (12.9-19.7) | 14.2 (10.5-18.3) | 14.3 (13.1-15.7) | -10.7 (-14.1 to -8.8) | -0.64 (-0.82 to -0.48) | 2.1 (-2.9 to 3.9) | 0.30 (-0.27 to 0.69) |
| Rayagada | 21.1 (17.0-25.3) | 18.5 (14.3-23.6) | 21.7 (20.5-22.9) | 3.0 (-0.8 to 5.2) | 0.20 (0.01 to 0.36) | 18.5 (14.1 to 22.2) | 2.47 (2.03 to 3.03) |
| Sambalpur | 20.5 (16.8-24.3) | 22.0 (17.4-26.9) | 19.1 (18.0-20.5) | -6.9 (-10.6 to -5.7) | -0.39 (-0.60 to -0.28) | -13.3 (-16.1 to -10.9) | -1.94 (-2.31 to -1.48) |
| Sonepur | 20.2 (16.8-24.2) | 21.3 (17.4-25.7) | 18.5 (17.4-19.8) | -8.6 (-11.4 to -6.9) | -0.50 (-0.64 to -0.35) | -13.2 (-16.3 to -10.8) | -1.90 (-2.35 to -1.47) |
| Sundargarh | 21.2 (17.0-25.5) | 23.4 (19.4-28.4) | 19.8 (18.8-21.0) | -6.4 (-9.6 to -4.7) | -0.36 (-0.53 to -0.22) | -15.6 (-18.4 to -13.9) | -2.29 (-2.70 to -1.95) |
| **Assam** | **17.5 (16.0-19.2)** | **14.5 (13.7-15.4)** | **13.5 (12.8-14.2)** | **-23.9 (-25.3 to -23.0)** | **-1.53 (-1.62 to -1.45)** | **-7.3 (-10.1 to -5.6)** | **-1.06 (-2.24 to 0.08)** |
| Baksa | 17.4 (12.8-23.1) | 11.8 (8.4-15.4) | 12.2 (11.4-13.1) | -29.8 (-33.2 to -28.2) | -2.05 (-2.26 to -1.85) | 4.7 (-2.3 to 7.2) | 0.61 (-0.18 to 1.13) |
| Barpeta | 22.1 (16.9-28.7) | 13.7 (10.0-18.0) | 13.8 (13.0-14.9) | -37.4 (-40.2 to -35.3) | -2.68 (-2.89 to -2.44) | 2.5 (-3.7 to 5.3) | 0.32 (-0.39 to 0.88) |
| Biswanath | 10.4 (7.2-14.9) | 13.4 (8.6-19.8) | 13.0 (12.2-13.7) | 28.2 (19.9 to 32.3) | 1.41 (1.12 to 1.70) | 0.0 (-8.9 to 2.9) | -0.16 (-1.16 to 0.54) |
| Bongaigaon | 24.6 (18.9-31.7) | 13.9 (10.0-17.8) | 14.2 (13.0-15.4) | -42.5 (-45.2 to -40.6) | -3.16 (-3.37 to -2.93) | 3.5 (-2.6 to 6.6) | 0.46 (-0.22 to 1.05) |
| Cachar | 20.6 (16.5-25.4) | 22.3 (17.1-28.4) | 16.6 (15.6-17.6) | -19.3 (-22.4 to -17.4) | -1.22 (-1.40 to -1.05) | -25.1 (-28.2 to -22.9) | -3.99 (-4.43 to -3.47) |
| Charaideo | 10.5 (7.3-15.0) | 9.0 (5.5-13.6) | 10.7 (9.9-11.6) | 4.4 (-2.4 to 9.0) | 0.20 (-0.08 to 0.56) | 24.8 (12.9 to 29.9) | 2.97 (1.88 to 3.93) |
| Chirang | 21.2 (16.0-27.7) | 12.3 (8.8-15.9) | 11.9 (11.1-12.9) | -43.8 (-46.4 to -42.2) | -3.29 (-3.50 to -3.07) | -1.9 (-7.5 to 1.4) | -0.29 (-0.96 to 0.33) |
| Darrang | 17.2 (12.8-22.3) | 13.4 (9.8-17.7) | 13.0 (12.2-13.9) | -23.5 (-27.3 to -21.4) | -1.55 (-1.78 to -1.33) | -1.5 (-7.7 to 1.3) | -0.23 (-0.98 to 0.33) |
| Dhemaji | 10.6 (7.6-14.4) | 10.8 (6.9-16.6) | 12.6 (12.0-13.3) | 21.7 (14.2 to 26.3) | 1.13 (0.83 to 1.43) | 21.4 (11.7 to 25.5) | 2.61 (1.72 to 3.41) |
| Dhubri | 32.2 (26.3-38.3) | 15.6 (12.7-19.0) | 15.6 (14.5-16.7) | -52.0 (-53.6 to -50.9) | -4.14 (-4.30 to -3.99) | 0.2 (-3.0 to 2.2) | 0.09 (-0.28 to 0.45) |
| Dibrugarh | 11.5 (8.3-15.4) | 10.5 (6.7-15.9) | 12.8 (12.1-13.6) | 13.5 (6.5 to 18.2) | 0.72 (0.43 to 1.04) | 26.7 (17.0 to 32.0) | 3.24 (2.39 to 4.16) |
| Dima Hasao | 15.9 (11.9-20.7) | 19.9 (14.2-26.6) | 13.7 (12.9-14.5) | -13.1 (-16.9 to -10.8) | -0.82 (-1.01 to -0.60) | -30.1 (-33.4 to -27.9) | -4.96 (-5.45 to -4.38) |
| Goalpara | 25.5 (21.1-30.9) | 15.0 (11.9-18.4) | 15.5 (14.8-16.2) | -39.6 (-41.5 to -38.8) | -2.86 (-3.01 to -2.75) | 3.2 (-0.9 to 5.5) | 0.51 (0.01 to 0.91) |
| Golaghat | 11.9 (8.6-15.9) | 13.1 (8.5-18.9) | 12.3 (11.6-13.1) | 5.0 (-1.2 to 8.5) | 0.26 (-0.01 to 0.54) | -3.7 (-10.7 to -1.1) | -0.66 (-1.45 to -0.02) |
| Hailakandi | 23.6 (18.6-28.9) | 21.9 (16.7-27.4) | 17.0 (15.9-18.3) | -27.9 (-30.9 to -26.9) | -1.87 (-2.07 to -1.75) | -22.1 (-26.1 to -19.8) | -3.43 (-4.04 to -2.94) |
| Hojai | 14.9 (9.9-21.6) | 18.4 (11.9-26.1) | 13.3 (11.8-15.2) | -8.3 (-15.0 to -5.6) | -0.57 (-0.89 to -0.28) | -26.0 (-32.1 to -24.8) | -4.27 (-5.19 to -3.82) |
| Jorhat | 11.2 (7.9-15.0) | 11.2 (7.1-16.4) | 11.6 (10.9-12.2) | 5.8 (-0.6 to 10.2) | 0.30 (0.02 to 0.63) | 7.5 (-1.5 to 10.1) | 0.88 (-0.06 to 1.51) |
| Kamrup | 19.8 (15.2-25.0) | 13.7 (10.4-17.4) | 11.7 (11.2-12.3) | -41.1 (-43.0 to -40.0) | -3.01 (-3.15 to -2.86) | -13.9 (-17.9 to -12.0) | -2.07 (-2.61 to -1.64) |
| Kamrup Metropolitan | 17.7 (12.9-23.1) | 14.0 (10.3-18.8) | 11.0 (9.8-12.4) | -37.4 (-40.8 to -36.0) | -2.70 (-2.94 to -2.50) | -20.3 (-24.7 to -17.6) | -3.19 (-3.78 to -2.56) |
| Karbi Anglong | 13.2 (9.9-17.5) | 16.2 (11.2-22.5) | 12.9 (12.3-13.5) | -1.5 (-6.0 to 0.4) | -0.10 (-0.30 to 0.08) | -19.0 (-25.4 to -16.8) | -3.00 (-3.91 to -2.42) |
| Karimganj | 23.5 (18.4-29.5) | 22.4 (17.4-27.7) | 17.6 (16.4-19.0) | -25.1 (-28.3 to -23.4) | -1.66 (-1.86 to -1.48) | -21.5 (-25.4 to -19.9) | -3.32 (-3.91 to -2.95) |
| Kokrajhar | 27.2 (21.9-33.1) | 13.6 (10.8-16.6) | 13.3 (12.5-14.1) | -51.7 (-53.3 to -50.6) | -4.10 (-4.26 to -3.95) | -2.2 (-6.2 to 0.0) | -0.26 (-0.75 to 0.14) |
| Lakhimpur | 9.5 (6.3-13.7) | 11.0 (6.9-16.3) | 11.5 (11.0-12.1) | 24.0 (16.3 to 28.4) | 1.21 (0.94 to 1.53) | 8.4 (-0.8 to 12.2) | 0.97 (0.03 to 1.78) |
| Majuli | 10.6 (7.2-15.2) | 11.2 (7.2-16.2) | 11.6 (11.0-12.2) | 12.0 (5.3 to 16.7) | 0.62 (0.36 to 0.96) | 7.9 (-0.7 to 11.7) | 0.93 (0.05 to 1.72) |
| Morigaon | 16.5 (12.8-21.2) | 14.9 (11.0-19.5) | 12.5 (11.9-13.1) | -24.0 (-27.6 to -22.4) | -1.58 (-1.80 to -1.41) | -15.1 (-18.8 to -12.6) | -2.28 (-2.76 to -1.75) |
| Nagaon | 14.0 (10.0-18.7) | 16.0 (11.1-21.7) | 13.1 (12.4-13.8) | -5.3 (-11.6 to -2.1) | -0.33 (-0.66 to -0.06) | -16.7 (-22.0 to -14.5) | -2.60 (-3.32 to -2.06) |
| Nalbari | 20.3 (15.5-25.8) | 13.0 (9.5-17.0) | 12.6 (11.9-13.4) | -37.9 (-40.5 to -36.3) | -2.72 (-2.91 to -2.53) | -2.0 (-6.9 to 0.5) | -0.29 (-0.87 to 0.21) |
| Sivasagar | 10.3 (6.9-14.9) | 9.5 (5.7-14.3) | 10.6 (10.0-11.4) | 6.7 (-1.1 to 11.3) | 0.31 (0.00 to 0.69) | 18.0 (6.4 to 21.7) | 2.14 (1.02 to 2.97) |
| Sonitpur | 12.4 (8.6-17.4) | 14.3 (9.6-20.5) | 13.7 (12.6-14.8) | 13.2 (6.4 to 16.6) | 0.68 (0.42 to 0.96) | -1.1 (-8.6 to 1.9) | -0.26 (-1.12 to 0.41) |
| South Salmara Mancachar | 29.9 (25.2-34.8) | 16.5 (14.1-19.3) | 16.1 (14.6-17.5) | -46.7 (-48.4 to -45.4) | -3.56 (-3.71 to -3.39) | -2.5 (-5.3 to -0.1) | -0.28 (-0.62 to 0.13) |
| Tinsukia | 15.1 (10.7-19.8) | 12.3 (7.7-18.5) | 16.3 (15.4-17.4) | 10.7 (3.7 to 14.1) | 0.56 (0.27 to 0.83) | 37.9 (26.2 to 42.4) | 4.48 (3.49 to 5.29) |
| Udalguri | 14.7 (10.3-19.6) | 12.1 (8.7-16.1) | 12.2 (11.2-13.1) | -15.7 (-20.7 to -13.5) | -1.01 (-1.28 to -0.78) | 2.8 (-3.6 to 6.5) | 0.35 (-0.37 to 1.04) |
| West Karbi Anglong | 14.7 (10.4-20.6) | 17.6 (12.3-24.3) | 11.9 (10.9-12.8) | -17.4 (-22.9 to -15.5) | -1.14 (-1.44 to -0.92) | -31.3 (-35.8 to -30.0) | -5.22 (-5.93 to -4.77) |
| **Andhra Pradesh** | **18.7 (17.7-19.8)** | **19.3 (18.5-20.0)** | **17.6 (16.9-18.3)** | **-7.1 (-8.4 to -6.0)** | **-0.38 (-0.45 to -0.30)** | **-9.5 (-11.3 to -8.3)** | **-1.34 (-2.20 to -0.44)** |
| Anantapur | 18.5 (15.6-22.0) | 24.0 (19.5-28.9) | 18.4 (17.2-19.7) | -1.1 (-4.5 to 0.7) | -0.03 (-0.21 to 0.10) | -23.8 (-27.2 to -22.3) | -3.70 (-4.24 to -3.36) |
| Chittoor | 16.5 (13.5-19.4) | 27.4 (22.7-32.9) | 19.6 (18.3-21.0) | 19.3 (15.1 to 21.1) | 1.06 (0.88 to 1.18) | -28.7 (-31.2 to -27.1) | -4.59 (-5.00 to -4.22) |
| East Godavari | 19.4 (15.7-23.4) | 16.3 (11.4-23.3) | 17.8 (16.2-19.3) | -8.1 (-11.5 to -5.6) | -0.47 (-0.65 to -0.28) | 12.2 (6.4 to 15.7) | 1.56 (1.02 to 2.23) |
| Guntur | 18.5 (15.5-22.0) | 16.9 (13.4-21.2) | 16.2 (14.7-18.1) | -12.7 (-15.7 to -10.8) | -0.76 (-0.93 to -0.60) | -3.5 (-7.3 to -1.4) | -0.48 (-0.93 to -0.06) |
| Krishna | 18.2 (15.2-21.5) | 18.4 (14.0-24.2) | 17.2 (16.0-18.7) | -6.0 (-10.0 to -4.6) | -0.33 (-0.55 to -0.21) | -5.9 (-10.5 to -3.1) | -0.86 (-1.41 to -0.30) |
| Kurnool | 18.9 (15.7-22.9) | 25.2 (19.9-31.3) | 18.6 (17.1-20.0) | -1.9 (-4.7 to 0.5) | -0.08 (-0.22 to 0.09) | -26.2 (-28.8 to -24.3) | -4.16 (-4.55 to -3.71) |
| Prakasam | 19.8 (16.1-23.7) | 16.8 (12.4-22.9) | 15.1 (13.6-16.7) | -23.9 (-26.6 to -22.7) | -1.56 (-1.73 to -1.43) | -9.3 (-14.2 to -6.4) | -1.39 (-1.99 to -0.79) |
| Sri Potti Sriramulu Nellore | 17.8 (14.2-22.4) | 24.7 (17.9-32.9) | 15.0 (13.7-16.6) | -15.7 (-19.1 to -13.2) | -0.98 (-1.17 to -0.76) | -38.6 (-42.3 to -37.2) | -6.68 (-7.33 to -6.22) |
| Srikakulam | 18.4 (14.4-22.5) | 13.3 (9.7-18.4) | 14.0 (12.9-15.2) | -23.8 (-26.8 to -22.3) | -1.56 (-1.74 to -1.40) | 7.1 (1.7 to 10.2) | 0.93 (0.37 to 1.53) |
| Visakhapatnam | 19.3 (15.4-23.8) | 12.4 (9.2-16.1) | 19.4 (18.0-20.9) | 1.1 (-2.8 to 3.6) | 0.08 (-0.10 to 0.26) | 59.3 (49.3 to 63.9) | 6.80 (6.00 to 7.41) |
| Vizianagaram | 20.5 (16.4-24.5) | 13.4 (9.8-17.8) | 16.4 (15.0-18.0) | -20.2 (-23.3 to -18.6) | -1.28 (-1.47 to -1.13) | 24.1 (17.9 to 28.0) | 3.09 (2.51 to 3.71) |
| West Godavari | 19.3 (15.7-23.1) | 19.0 (13.4-26.0) | 19.6 (17.8-21.5) | 1.3 (-3.3 to 3.3) | 0.09 (-0.14 to 0.25) | 4.7 (-1.7 to 7.7) | 0.59 (-0.10 to 1.20) |
| YSR | 19.1 (15.5-23.2) | 21.2 (16.2-27.1) | 18.7 (17.1-20.3) | -1.7 (-5.3 to 0.9) | -0.08 (-0.26 to 0.11) | -11.0 (-15.3 to -8.3) | -1.61 (-2.18 to -1.08) |
| **West Bengal** | **18.9 (17.7-20.0)** | **17.5 (16.9-18.1)** | **13.7 (13.2-14.2)** | **-28.5 (-29.2 to -27.8)** | **-1.88 (-1.93 to -1.82)** | **-22.6 (-23.8 to -21.8)** | **-3.51 (-4.37 to -2.56)** |
| Alipurduar | 20.7 (16.9-24.7) | 16.0 (13.2-19.4) | 13.0 (12.1-14.1) | -37.4 (-40.0 to -36.2) | -2.66 (-2.87 to -2.52) | -18.6 (-21.4 to -16.7) | -2.80 (-3.21 to -2.42) |
| Bankura | 21.8 (18.1-25.8) | 21.9 (18.4-25.8) | 17.6 (16.6-18.6) | -19.9 (-22.2 to -18.7) | -1.25 (-1.39 to -1.14) | -20.3 (-22.9 to -18.8) | -3.07 (-3.47 to -2.76) |
| Birbhum | 20.1 (16.8-24.0) | 21.8 (18.7-25.2) | 16.6 (15.9-17.3) | -17.9 (-20.2 to -15.9) | -1.11 (-1.25 to -0.95) | -24.6 (-26.6 to -23.7) | -3.80 (-4.14 to -3.61) |
| Cooch Behar | 23.9 (20.1-27.9) | 17.8 (14.9-20.8) | 14.5 (13.6-15.5) | -39.7 (-41.9 to -38.5) | -2.87 (-3.05 to -2.72) | -18.6 (-20.8 to -16.8) | -2.79 (-3.10 to -2.43) |
| Dakshin Dinajpur | 18.6 (15.4-22.4) | 18.2 (15.4-21.1) | 15.3 (13.9-16.6) | -18.1 (-20.9 to -16.6) | -1.13 (-1.30 to -0.99) | -16.3 (-19.1 to -14.5) | -2.41 (-2.81 to -2.05) |
| Darjeeling | 12.5 (10.0-15.2) | 15.2 (12.3-18.5) | 11.6 (10.8-12.5) | -7.2 (-10.8 to -5.5) | -0.42 (-0.60 to -0.27) | -23.6 (-26.3 to -22.3) | -3.68 (-4.08 to -3.36) |
| Hooghly | 18.7 (16.0-21.5) | 14.8 (12.6-17.5) | 12.9 (12.2-13.6) | -31.6 (-33.5 to -30.2) | -2.15 (-2.28 to -2.01) | -13.3 (-15.8 to -11.4) | -1.92 (-2.27 to -1.56) |
| Howrah | 17.6 (14.3-21.1) | 13.1 (10.7-15.9) | 11.0 (9.9-12.2) | -37.6 (-40.0 to -36.1) | -2.68 (-2.87 to -2.51) | -15.9 (-19.2 to -13.3) | -2.37 (-2.82 to -1.85) |
| Jalpaiguri | 17.7 (13.9-21.5) | 16.8 (13.8-20.3) | 14.5 (13.3-15.8) | -18.0 (-21.3 to -16.1) | -1.14 (-1.33 to -0.96) | -14.0 (-17.1 to -12.1) | -2.05 (-2.48 to -1.67) |
| Jhargram | 21.9 (17.8-26.4) | 23.5 (19.5-28.5) | 15.8 (14.8-16.6) | -28.2 (-30.5 to -27.0) | -1.88 (-2.03 to -1.76) | -33.2 (-35.1 to -31.7) | -5.47 (-5.78 to -5.11) |
| Kalimpong | 10.2 (7.8-12.6) | 11.0 (8.5-13.5) | 10.1 (9.4-11.0) | -0.3 (-4.9 to 2.1) | -0.01 (-0.23 to 0.18) | -7.7 (-11.6 to -5.3) | -1.10 (-1.59 to -0.63) |
| Kolkata | 16.3 (12.6-20.6) | 11.2 (8.6-14.1) | 9.7 (8.2-11.3) | -40.6 (-44.1 to -38.4) | -3.00 (-3.27 to -2.71) | -12.9 (-18.7 to -10.1) | -1.99 (-2.74 to -1.36) |
| Maldah | 17.4 (14.3-20.6) | 18.6 (16.0-21.4) | 16.3 (15.6-17.1) | -6.3 (-9.3 to -4.6) | -0.35 (-0.51 to -0.22) | -12.6 (-14.9 to -11.1) | -1.79 (-2.12 to -1.50) |
| Murshidabad | 17.4 (14.6-20.4) | 19.7 (17.0-23.2) | 14.1 (13.4-15.0) | -19.1 (-21.4 to -17.5) | -1.20 (-1.33 to -1.05) | -29.1 (-30.5 to -27.4) | -4.64 (-4.87 to -4.28) |
| Nadia | 16.3 (14.1-18.4) | 16.3 (14.4-18.6) | 11.9 (11.1-12.8) | -27.5 (-29.5 to -26.3) | -1.81 (-1.96 to -1.70) | -27.5 (-29.4 to -26.0) | -4.35 (-4.66 to -4.02) |
| North 24 Parganas | 16.8 (14.1-19.3) | 14.1 (11.8-16.2) | 10.9 (10.1-11.8) | -36.0 (-38.3 to -35.0) | -2.53 (-2.71 to -2.41) | -23.5 (-25.8 to -22.2) | -3.62 (-3.98 to -3.34) |
| Paschim Burdwan | 20.1 (15.8-25.3) | 21.7 (17.9-25.8) | 16.5 (15.4-17.8) | -17.6 (-20.8 to -15.2) | -1.10 (-1.29 to -0.90) | -24.0 (-26.2 to -22.9) | -3.73 (-4.07 to -3.47) |
| Pashchim Medinipur | 20.7 (17.9-23.5) | 19.3 (16.5-22.5) | 14.3 (13.5-15.1) | -31.8 (-33.4 to -30.8) | -2.16 (-2.28 to -2.06) | -26.5 (-28.5 to -25.2) | -4.18 (-4.48 to -3.88) |
| Purba Burdwan | 20.3 (17.1-23.4) | 17.3 (14.5-20.6) | 15.1 (14.3-16.1) | -25.9 (-28.0 to -24.6) | -1.70 (-1.83 to -1.57) | -12.6 (-15.4 to -11.2) | -1.80 (-2.20 to -1.52) |
| Purba Medinipur | 21.8 (18.6-25.1) | 20.6 (17.0-24.4) | 13.6 (12.8-14.5) | -38.2 (-40.1 to -37.2) | -2.72 (-2.88 to -2.61) | -34.3 (-36.6 to -32.4) | -5.69 (-6.09 to -5.25) |
| Puruliya | 22.3 (17.9-27.4) | 26.2 (22.6-30.3) | 18.9 (18.2-19.5) | -15.6 (-18.6 to -14.1) | -0.96 (-1.13 to -0.82) | -28.5 (-30.2 to -27.2) | -4.52 (-4.82 to -4.24) |
| South 24 Parganas | 20.2 (16.8-23.7) | 17.0 (13.8-20.3) | 12.9 (12.0-14.1) | -36.7 (-39.1 to -35.5) | -2.60 (-2.78 to -2.46) | -24.2 (-27.1 to -22.6) | -3.78 (-4.23 to -3.42) |
| Uttar Dinajpur | 17.0 (14.3-20.0) | 15.8 (13.1-18.5) | 13.6 (13.1-14.2) | -20.3 (-22.6 to -18.5) | -1.28 (-1.42 to -1.13) | -14.5 (-16.6 to -13.0) | -2.10 (-2.40 to -1.81) |
| **Tripura** | **20.7 (18.7-22.7)** | **18.9 (18.0-19.7)** | **16.5 (15.8-17.2)** | **-20.8 (-22.7 to -19.5)** | **-1.31 (-1.43 to -1.19)** | **-13.2 (-15.5 to -11.4)** | **-1.94 (-2.98 to -0.92)** |
| Dhalai | 20.1 (16.4-24.7) | 16.5 (13.1-20.6) | 14.0 (12.9-15.0) | -30.5 (-33.4 to -28.7) | -2.08 (-2.28 to -1.89) | -15.3 (-18.8 to -12.9) | -2.28 (-2.76 to -1.80) |
| Gomati | 20.2 (16.6-24.8) | 18.0 (14.8-21.7) | 16.7 (15.1-18.4) | -17.5 (-21.0 to -16.2) | -1.10 (-1.30 to -0.96) | -7.6 (-11.0 to -5.2) | -1.05 (-1.49 to -0.61) |
| Khowai | 21.8 (18.2-25.9) | 19.7 (16.8-23.2) | 16.1 (14.7-17.6) | -26.6 (-28.8 to -24.8) | -1.75 (-1.90 to -1.59) | -18.7 (-21.6 to -17.4) | -2.81 (-3.24 to -2.53) |
| North Tripura | 20.0 (15.9-23.9) | 18.8 (15.0-23.4) | 15.6 (14.5-16.8) | -22.1 (-25.0 to -20.6) | -1.42 (-1.60 to -1.27) | -17.0 (-20.3 to -15.4) | -2.56 (-3.02 to -2.20) |
| Sepahijala | 20.9 (17.1-25.3) | 19.9 (16.8-23.3) | 18.3 (16.8-19.8) | -12.5 (-16.2 to -10.8) | -0.75 (-0.97 to -0.60) | -8.2 (-11.4 to -6.7) | -1.14 (-1.56 to -0.84) |
| South Tripura | 20.4 (16.4-25.4) | 18.0 (14.9-21.7) | 17.1 (15.8-18.6) | -16.1 (-19.4 to -13.6) | -1.01 (-1.19 to -0.79) | -4.8 (-8.4 to -1.4) | -0.65 (-1.10 to -0.06) |
| Unakoti | 20.6 (16.7-24.8) | 18.4 (14.8-22.8) | 15.0 (13.8-16.6) | -27.4 (-30.6 to -26.1) | -1.83 (-2.05 to -1.69) | -18.6 (-21.6 to -16.5) | -2.82 (-3.24 to -2.38) |
| West Tripura | 21.0 (17.2-25.8) | 20.1 (16.3-24.1) | 17.0 (15.4-18.8) | -19.5 (-22.6 to -17.4) | -1.25 (-1.42 to -1.04) | -15.7 (-19.1 to -14.0) | -2.36 (-2.82 to -1.97) |
| **Arunachal Pradesh** | **14.5 (13.0-16.0)** | **17.8 (17.0-18.7)** | **16.9 (16.2-17.7)** | **18.3 (12.8 to 21.1)** | **0.99 (0.76 to 1.18)** | **-2.9 (-8.6 to 0.6)** | **-0.75 (-1.76 to 0.29)** |
| Anjaw | 15.9 (10.0-23.5) | 16.0 (9.6-24.2) | 14.9 (13.4-16.4) | -3.0 (-12.9 to 1.2) | -0.29 (-0.74 to 0.13) | -2.7 (-11.8 to -0.5) | -0.64 (-1.62 to 0.06) |
| Changlang | 16.3 (11.7-22.0) | 18.8 (12.1-26.7) | 17.9 (16.5-19.8) | 12.3 (5.8 to 14.9) | 0.65 (0.39 to 0.87) | -1.7 (-9.2 to 2.0) | -0.39 (-1.21 to 0.42) |
| Dibang Valley | 12.7 (7.3-20.0) | 13.4 (7.7-21.9) | 11.3 (9.7-13.1) | -5.5 (-17.3 to -1.6) | -0.52 (-1.04 to -0.03) | -11.1 (-19.6 to -9.9) | -1.99 (-2.89 to -1.32) |
| East Kameng | 8.4 (5.2-12.6) | 17.2 (11.1-25.5) | 15.4 (14.2-16.9) | 91.1 (73.2 to 96.6) | 3.74 (3.32 to 4.09) | -7.3 (-13.7 to -3.8) | -1.23 (-1.92 to -0.40) |
| East Siang | 13.3 (8.8-19.4) | 17.8 (10.5-28.4) | 16.1 (14.6-18.0) | 24.7 (15.1 to 29.3) | 1.22 (0.88 to 1.57) | -4.6 (-12.5 to -1.8) | -0.96 (-1.73 to -0.12) |
| Kamle | 7.9 (4.5-13.4) | 14.2 (7.7-23.3) | 11.2 (10.3-13.2) | 53.0 (36.4 to 56.6) | 2.29 (1.89 to 2.71) | -14.9 (-27.6 to -12.9) | -2.78 (-4.33 to -1.79) |
| Kra Daddi | 7.5 (4.1-12.6) | 16.1 (9.2-26.9) | 15.8 (14.6-17.7) | 127.3 (98.3 to 132.0) | 4.70 (4.14 to 5.10) | 4.3 (-7.0 to 7.2) | 0.21 (-0.87 to 1.14) |
| Kurung Kumey | 8.1 (4.8-12.7) | 18.3 (11.0-27.6) | 18.2 (16.4-19.9) | 138.8 (116.1 to 145.5) | 5.07 (4.67 to 5.45) | 4.2 (-4.8 to 9.4) | 0.33 (-0.55 to 1.43) |
| Lohit | 15.8 (10.6-22.7) | 18.5 (11.4-28.6) | 17.8 (16.0-19.4) | 16.1 (8.0 to 20.0) | 0.81 (0.51 to 1.13) | 0.9 (-8.0 to 4.3) | -0.10 (-1.04 to 0.74) |
| Longding | 14.4 (9.9-20.4) | 16.3 (10.4-23.7) | 16.7 (15.4-18.1) | 18.1 (10.5 to 21.2) | 0.93 (0.64 to 1.19) | 5.8 (-3.9 to 8.9) | 0.63 (-0.42 to 1.35) |
| Lower Dibang Valley | 14.9 (10.0-21.2) | 18.5 (11.2-30.2) | 17.3 (15.8-19.0) | 20.2 (11.9 to 22.6) | 1.02 (0.71 to 1.26) | -1.4 (-9.5 to 2.1) | -0.44 (-1.25 to 0.43) |
| Lower Siang | 10.9 (7.5-15.9) | 16.8 (9.7-26.9) | 13.8 (12.6-15.3) | 30.2 (20.8 to 34.6) | 1.49 (1.16 to 1.81) | -13.9 (-21.2 to -11.4) | -2.34 (-3.17 to -1.55) |
| Lower Subansiri | 7.3 (4.6-11.0) | 15.3 (9.1-24.6) | 11.6 (10.8-12.7) | 66.0 (52.1 to 70.9) | 2.90 (2.54 to 3.24) | -20.1 (-27.3 to -16.8) | -3.37 (-4.27 to -2.43) |
| Namsai | 17.1 (11.8-23.7) | 20.4 (13.0-31.9) | 19.7 (18.4-21.2) | 18.2 (9.4 to 21.3) | 0.93 (0.58 to 1.19) | 0.5 (-8.1 to 4.3) | -0.15 (-1.05 to 0.74) |
| Pakke Kessang | 8.4 (5.9-11.5) | 17.9 (12.1-25.8) | 12.8 (12.0-13.7) | 56.6 (46.8 to 62.6) | 2.61 (2.32 to 2.94) | -26.5 (-31.4 to -23.5) | -4.37 (-5.05 to -3.58) |
| Papum Pare | 8.0 (4.9-12.6) | 16.8 (9.8-26.1) | 11.6 (10.3-13.2) | 52.4 (38.1 to 57.1) | 2.37 (1.96 to 2.73) | -28.0 (-34.9 to -25.5) | -4.79 (-5.74 to -3.94) |
| Shi Yomi | 9.2 (5.8-14.0) | 14.8 (9.0-23.8) | 12.8 (11.7-14.1) | 45.5 (33.7 to 51.0) | 2.12 (1.77 to 2.50) | -8.1 (-15.6 to -4.6) | -1.47 (-2.22 to -0.53) |
| Siang | 9.3 (5.2-15.8) | 14.2 (7.9-23.0) | 13.2 (11.9-14.8) | 51.4 (34.9 to 54.0) | 2.28 (1.82 to 2.61) | -1.2 (-11.2 to 1.8) | -0.51 (-1.53 to 0.39) |
| Tawang | 8.4 (5.2-12.6) | 14.0 (9.3-20.8) | 12.3 (10.9-13.8) | 53.0 (37.6 to 56.0) | 2.39 (1.94 to 2.69) | -9.5 (-15.2 to -5.0) | -1.58 (-2.17 to -0.57) |
| Tirap | 16.5 (11.3-23.0) | 18.3 (11.1-27.3) | 18.0 (16.6-19.7) | 12.1 (3.9 to 15.4) | 0.61 (0.28 to 0.90) | 2.0 (-7.0 to 6.0) | 0.10 (-0.88 to 0.97) |
| Upper Siang | 10.7 (5.5-18.9) | 14.6 (8.1-23.3) | 14.0 (12.7-15.6) | 42.5 (23.3 to 46.0) | 1.87 (1.29 to 2.29) | 2.2 (-10.1 to 5.7) | -0.06 (-1.36 to 0.94) |
| Upper Subansiri | 7.7 (4.4-12.4) | 16.4 (9.5-27.0) | 16.7 (15.3-18.1) | 131.0 (104.9 to 133.7) | 4.83 (4.34 to 5.15) | 8.2 (-3.0 to 12.4) | 0.79 (-0.29 to 1.82) |
| West Kameng | 8.9 (5.7-13.1) | 14.0 (9.4-20.4) | 10.9 (9.6-12.3) | 27.5 (14.6 to 30.3) | 1.32 (0.86 to 1.61) | -20.3 (-25.4 to -17.6) | -3.31 (-3.92 to -2.57) |
| West Siang | 7.9 (4.2-13.0) | 13.3 (7.4-23.0) | 12.2 (11.0-13.8) | 65.9 (45.0 to 69.0) | 2.78 (2.25 to 3.17) | -2.2 (-12.0 to 0.2) | -0.71 (-1.66 to 0.18) |
| **Meghalaya** | **20.8 (18.8-22.7)** | **17.1 (16.2-18.1)** | **12.9 (12.2-13.6)** | **-38.5 (-40.2 to -37.2)** | **-2.75 (-2.88 to -2.61)** | **-25.1 (-27.1 to -23.5)** | **-4.04 (-5.25 to -2.87)** |
| East Garo Hills | 22.8 (17.8-28.7) | 16.2 (13.0-19.9) | 16.3 (15.3-17.5) | -28.3 (-31.0 to -26.7) | -1.90 (-2.07 to -1.73) | 0.9 (-2.7 to 3.7) | 0.18 (-0.25 to 0.66) |
| East Jaintia Hills | 17.1 (13.1-21.4) | 20.7 (15.8-25.7) | 12.8 (12.1-13.8) | -24.7 (-28.1 to -22.8) | -1.63 (-1.84 to -1.43) | -38.0 (-40.7 to -36.3) | -6.49 (-6.98 to -6.04) |
| East Khasi Hills | 17.7 (13.5-22.6) | 16.5 (12.6-21.1) | 9.6 (8.7-11.0) | -45.4 (-49.0 to -43.9) | -3.47 (-3.77 to -3.24) | -41.3 (-44.1 to -39.2) | -7.25 (-7.75 to -6.64) |
| North Garo Hills | 24.2 (19.6-29.9) | 15.8 (12.6-19.8) | 16.0 (14.9-17.3) | -34.1 (-36.8 to -32.6) | -2.38 (-2.57 to -2.21) | 1.3 (-2.2 to 3.5) | 0.22 (-0.17 to 0.63) |
| Ri Bhoi | 16.3 (12.4-20.8) | 15.0 (11.0-19.5) | 9.6 (8.7-10.6) | -40.9 (-44.6 to -39.4) | -3.03 (-3.31 to -2.81) | -35.9 (-39.3 to -33.9) | -6.09 (-6.67 to -5.54) |
| South Garo Hills | 24.2 (18.8-30.3) | 18.7 (15.2-22.8) | 18.4 (16.9-20.2) | -23.8 (-27.6 to -21.4) | -1.56 (-1.80 to -1.34) | -1.3 (-4.8 to 1.4) | -0.13 (-0.55 to 0.34) |
| South West Garo Hills | 27.2 (22.3-32.7) | 18.7 (15.8-21.9) | 16.3 (15.1-18.0) | -40.4 (-42.7 to -39.4) | -2.95 (-3.13 to -2.81) | -12.9 (-15.6 to -10.8) | -1.88 (-2.23 to -1.45) |
| South West Khasi Hills | 22.0 (18.0-26.3) | 17.4 (14.4-20.5) | 13.7 (12.4-15.0) | -38.3 (-40.8 to -37.1) | -2.75 (-2.94 to -2.60) | -21.9 (-24.9 to -19.9) | -3.37 (-3.83 to -2.95) |
| West Garo Hills | 27.4 (23.0-32.1) | 17.7 (15.0-21.1) | 16.8 (15.7-17.8) | -39.3 (-41.1 to -38.2) | -2.82 (-2.97 to -2.70) | -5.5 (-7.7 to -3.2) | -0.71 (-0.99 to -0.32) |
| West Jaintia Hills | 16.3 (12.3-20.8) | 18.8 (14.4-23.6) | 10.7 (9.9-11.8) | -34.1 (-37.2 to -32.5) | -2.40 (-2.61 to -2.20) | -43.1 (-45.1 to -41.3) | -7.61 (-7.98 to -7.10) |
| West Khasi Hills | 20.1 (15.9-24.5) | 15.9 (12.7-19.7) | 12.2 (11.6-12.8) | -39.6 (-41.9 to -38.3) | -2.87 (-3.05 to -2.71) | -23.6 (-26.7 to -22.0) | -3.67 (-4.16 to -3.31) |
| **Karnataka** | **17.2 (16.3-18.2)** | **20.2 (19.6-20.9)** | **17.5 (16.8-18.1)** | **0.4 (-0.8 to 1.4)** | **0.07 (0.01 to 0.14)** | **-14.5 (-15.2 to -13.5)** | **-2.09 (-2.95 to -1.23)** |
| Bagalkot | 18.2 (14.4-22.6) | 28.4 (25.6-31.5) | 17.6 (16.1-18.9) | -2.9 (-7.6 to -1.0) | -0.16 (-0.40 to 0.00) | -39.1 (-40.2 to -38.1) | -6.65 (-6.87 to -6.41) |
| Ballari | 19.5 (16.1-23.6) | 24.1 (20.5-27.6) | 17.3 (16.3-18.3) | -11.5 (-14.0 to -9.5) | -0.68 (-0.81 to -0.52) | -28.7 (-31.2 to -27.5) | -4.58 (-5.01 to -4.29) |
| Belagavi | 18.8 (15.5-22.3) | 26.3 (23.7-28.9) | 18.8 (17.6-20.1) | -0.4 (-4.8 to 1.5) | 0.01 (-0.23 to 0.15) | -29.5 (-31.2 to -28.5) | -4.71 (-5.01 to -4.50) |
| Bengaluru Rural | 16.3 (12.8-20.2) | 14.0 (11.0-17.6) | 15.2 (14.3-16.2) | -6.4 (-10.2 to -3.9) | -0.38 (-0.57 to -0.18) | 9.1 (4.0 to 11.1) | 1.28 (0.69 to 1.65) |
| Bengaluru Urban | 14.9 (11.7-18.4) | 13.0 (10.1-16.1) | 14.1 (12.9-15.4) | -5.2 (-9.6 to -3.5) | -0.30 (-0.53 to -0.15) | 8.6 (3.4 to 12.4) | 1.20 (0.62 to 1.82) |
| Bidar | 25.1 (20.7-30.0) | 17.4 (14.5-20.4) | 18.8 (17.5-20.0) | -25.5 (-28.5 to -24.0) | -1.67 (-1.87 to -1.53) | 7.7 (4.0 to 9.5) | 1.14 (0.70 to 1.43) |
| Chamarajanagar | 17.2 (13.5-21.0) | 17.1 (13.9-20.6) | 16.8 (15.7-18.0) | -1.8 (-6.2 to 0.4) | -0.09 (-0.31 to 0.08) | -1.5 (-5.0 to 0.4) | -0.14 (-0.59 to 0.20) |
| Chikballapur | 17.8 (14.5-21.6) | 13.0 (10.5-16.1) | 14.6 (13.7-15.5) | -18.2 (-21.3 to -16.3) | -1.14 (-1.32 to -0.97) | 12.5 (6.9 to 14.0) | 1.73 (1.10 to 2.01) |
| Chikkamagaluru | 15.5 (12.0-20.3) | 18.4 (14.1-23.3) | 18.5 (17.0-20.2) | 20.3 (14.3 to 23.3) | 1.08 (0.84 to 1.29) | 1.4 (-3.0 to 3.9) | 0.23 (-0.29 to 0.69) |
| Chitradurga | 18.2 (14.5-22.1) | 19.2 (15.4-23.4) | 18.3 (17.1-19.6) | 0.5 (-4.1 to 3.1) | 0.05 (-0.18 to 0.23) | -4.6 (-8.5 to -2.3) | -0.61 (-1.11 to -0.19) |
| Dakshina Kannada | 11.3 (7.9-15.6) | 14.8 (11.3-19.4) | 15.9 (14.4-17.6) | 43.6 (34.1 to 46.0) | 2.10 (1.79 to 2.29) | 8.7 (3.6 to 11.0) | 1.19 (0.65 to 1.63) |
| Davanagere | 16.1 (11.8-20.4) | 22.2 (18.3-26.4) | 15.6 (14.4-16.9) | -2.7 (-7.8 to 0.2) | -0.15 (-0.41 to 0.07) | -30.3 (-32.4 to -29.0) | -4.90 (-5.25 to -4.58) |
| Dharwad | 16.3 (12.7-20.3) | 29.9 (26.5-32.9) | 21.8 (20.3-23.2) | 34.7 (28.9 to 37.7) | 1.76 (1.55 to 1.94) | -27.9 (-29.5 to -26.6) | -4.41 (-4.67 to -4.14) |
| Gadag | 16.7 (12.5-21.3) | 29.8 (27.0-32.4) | 20.1 (18.6-21.7) | 21.6 (15.7 to 24.6) | 1.15 (0.91 to 1.35) | -33.4 (-34.8 to -32.6) | -5.47 (-5.73 to -5.28) |
| Hassan | 15.0 (11.4-18.7) | 18.0 (14.4-22.2) | 18.3 (16.9-19.5) | 22.9 (18.3 to 25.6) | 1.22 (1.04 to 1.40) | 2.2 (-1.3 to 5.4) | 0.36 (-0.05 to 0.88) |
| Haveri | 15.5 (11.8-19.3) | 25.8 (22.4-28.9) | 16.9 (15.7-18.2) | 9.8 (4.8 to 12.1) | 0.55 (0.33 to 0.73) | -35.1 (-36.5 to -34.1) | -5.82 (-6.07 to -5.57) |
| Kalaburgi | 24.4 (19.7-29.3) | 21.7 (18.5-25.2) | 20.7 (19.5-21.8) | -15.3 (-19.0 to -13.6) | -0.94 (-1.16 to -0.79) | -5.0 (-7.3 to -3.0) | -0.62 (-0.92 to -0.29) |
| Kodagu | 12.7 (10.0-15.7) | 15.2 (12.3-18.8) | 14.1 (13.2-15.1) | 11.3 (6.3 to 13.2) | 0.64 (0.41 to 0.78) | -7.4 (-10.4 to -5.5) | -1.02 (-1.40 to -0.65) |
| Kolar | 17.9 (14.6-21.7) | 15.1 (12.2-18.4) | 16.7 (15.5-17.7) | -7.1 (-10.4 to -4.8) | -0.41 (-0.58 to -0.23) | 10.4 (5.5 to 13.7) | 1.46 (0.90 to 1.97) |
| Koppal | 18.4 (14.3-22.7) | 28.9 (26.3-31.4) | 20.8 (19.4-22.1) | 13.8 (9.0 to 15.9) | 0.77 (0.56 to 0.92) | -28.9 (-30.0 to -27.9) | -4.58 (-4.78 to -4.38) |
| Mandya | 15.5 (12.1-19.3) | 16.6 (13.2-20.3) | 17.3 (16.1-18.5) | 12.0 (7.6 to 14.8) | 0.68 (0.49 to 0.86) | 3.9 (-0.1 to 6.6) | 0.59 (0.13 to 1.05) |
| Mysuru | 15.4 (12.4-18.9) | 16.9 (13.7-20.6) | 16.6 (15.3-18.0) | 8.2 (3.8 to 11.2) | 0.48 (0.28 to 0.68) | -1.8 (-4.7 to 0.5) | -0.19 (-0.54 to 0.21) |
| Raichur | 20.5 (16.5-25.0) | 26.9 (23.1-31.0) | 22.1 (20.7-23.5) | 7.8 (3.4 to 10.3) | 0.46 (0.25 to 0.63) | -18.6 (-20.9 to -16.9) | -2.77 (-3.12 to -2.45) |
| Ramanagara | 16.4 (13.2-19.8) | 16.2 (13.0-19.9) | 17.4 (16.4-18.6) | 6.0 (2.3 to 8.2) | 0.37 (0.19 to 0.52) | 8.0 (5.0 to 11.0) | 1.15 (0.83 to 1.64) |
| Shivamogga | 14.0 (10.6-17.7) | 21.0 (17.5-24.7) | 17.3 (16.1-18.7) | 25.0 (19.0 to 28.5) | 1.31 (1.08 to 1.53) | -17.8 (-20.8 to -15.6) | -2.65 (-3.11 to -2.23) |
| Tumakuru | 17.3 (14.2-20.6) | 16.0 (13.0-19.7) | 17.8 (17.0-19.0) | 2.8 (-0.1 to 5.1) | 0.19 (0.05 to 0.35) | 11.7 (8.5 to 14.5) | 1.64 (1.31 to 2.08) |
| Udupi | 12.0 (8.1-17.3) | 17.7 (13.1-22.8) | 18.0 (16.6-19.4) | 54.0 (43.6 to 58.0) | 2.50 (2.19 to 2.76) | 2.7 (-2.9 to 4.9) | 0.36 (-0.27 to 0.83) |
| Uttara Kannada | 13.4 (10.2-17.1) | 23.8 (20.4-27.4) | 17.2 (15.8-18.9) | 29.2 (23.5 to 33.0) | 1.50 (1.30 to 1.74) | -28.2 (-30.3 to -26.7) | -4.49 (-4.83 to -4.16) |
| Vijaypura | 23.5 (19.0-28.6) | 28.9 (25.9-32.1) | 18.0 (16.5-19.5) | -23.5 (-26.2 to -22.0) | -1.53 (-1.70 to -1.38) | -38.4 (-39.9 to -37.6) | -6.51 (-6.80 to -6.30) |
| Yadgir | 22.0 (17.7-26.4) | 24.9 (21.4-28.5) | 21.6 (20.4-22.7) | -1.8 (-5.6 to 0.2) | -0.08 (-0.28 to 0.07) | -13.7 (-15.6 to -12.4) | -1.97 (-2.23 to -1.72) |
| **Telangana** | **25.4 (23.3-27.4)** | **23.7 (22.4-25.2)** | **19.1 (17.8-20.5)** | **-25.1 (-27.0 to -23.5)** | **-1.63 (-1.76 to -1.49)** | **-20.0 (-21.4 to -19.0)** | **-3.07 (-4.05 to -2.17)** |
| Adilabad | 39.5 (29.8-50.2) | 26.3 (21.0-33.1) | 25.8 (24.3-27.3) | -34.7 (-37.6 to -33.7) | -2.44 (-2.64 to -2.30) | -1.8 (-5.0 to 0.3) | -0.22 (-0.58 to 0.19) |
| Bhadradri Kothagudem | 28.1 (19.8-38.0) | 26.4 (19.1-34.9) | 21.3 (19.7-22.7) | -23.0 (-28.1 to -21.3) | -1.54 (-1.84 to -1.33) | -18.3 (-22.9 to -15.3) | -2.85 (-3.46 to -2.19) |
| Hyderabad | 19.4 (14.0-26.2) | 19.2 (14.8-23.8) | 15.4 (13.8-17.2) | -19.7 (-24.2 to -18.2) | -1.30 (-1.54 to -1.11) | -19.4 (-23.3 to -17.9) | -3.00 (-3.54 to -2.62) |
| Jagitial | 25.0 (17.2-33.4) | 23.7 (18.4-29.8) | 19.5 (18.1-20.9) | -20.7 (-25.5 to -18.0) | -1.37 (-1.64 to -1.09) | -17.4 (-21.1 to -14.9) | -2.64 (-3.16 to -2.11) |
| Jangoan | 20.1 (14.5-26.3) | 26.1 (20.7-32.2) | 20.5 (19.2-21.9) | 3.5 (-2.3 to 6.2) | 0.19 (-0.08 to 0.41) | -21.3 (-24.9 to -19.9) | -3.29 (-3.83 to -2.94) |
| Jayashankar Bhupalpally | 23.5 (15.9-32.8) | 30.1 (21.6-39.5) | 21.9 (20.7-23.5) | -4.5 (-11.3 to -1.2) | -0.31 (-0.64 to -0.01) | -26.7 (-30.0 to -25.4) | -4.29 (-4.78 to -3.92) |
| Jogulamba Gadwal | 34.3 (26.9-42.4) | 27.5 (20.4-35.4) | 19.7 (17.8-21.3) | -42.7 (-45.1 to -41.5) | -3.17 (-3.37 to -3.01) | -28.0 (-31.4 to -26.3) | -4.52 (-5.05 to -4.08) |
| Kamareddy | 30.4 (22.6-39.1) | 26.9 (22.0-31.6) | 22.3 (21.1-23.8) | -26.0 (-29.8 to -24.3) | -1.74 (-1.98 to -1.55) | -17.4 (-19.9 to -15.6) | -2.60 (-2.95 to -2.23) |
| Karimnagar | 20.9 (15.0-27.7) | 26.7 (20.2-33.8) | 20.1 (18.7-21.6) | -2.4 (-7.4 to 0.6) | -0.16 (-0.39 to 0.09) | -24.2 (-27.8 to -22.0) | -3.82 (-4.36 to -3.31) |
| Khammam | 28.0 (22.1-34.9) | 21.0 (16.3-26.8) | 18.0 (16.4-19.6) | -35.8 (-38.3 to -34.1) | -2.53 (-2.71 to -2.34) | -13.7 (-17.8 to -11.1) | -2.05 (-2.59 to -1.50) |
| Kumuram Bheem Asifabad | 36.5 (27.5-48.6) | 32.2 (24.1-40.7) | 24.6 (23.2-26.1) | -32.0 (-35.7 to -29.6) | -2.23 (-2.47 to -1.96) | -23.2 (-26.7 to -21.6) | -3.63 (-4.16 to -3.25) |
| Mahabubnagar | 31.8 (25.1-40.2) | 26.2 (20.4-33.1) | 19.4 (17.7-21.1) | -39.2 (-41.7 to -38.1) | -2.84 (-3.03 to -2.69) | -26.1 (-29.2 to -24.8) | -4.15 (-4.62 to -3.80) |
| Mahuababad | 21.6 (15.0-30.3) | 25.0 (18.4-33.0) | 19.7 (18.1-21.7) | -6.9 (-13.2 to -4.7) | -0.46 (-0.76 to -0.22) | -20.2 (-24.8 to -18.6) | -3.17 (-3.82 to -2.73) |
| Mancherial | 27.8 (19.8-38.3) | 29.4 (22.3-37.4) | 21.5 (20.1-23.0) | -21.5 (-25.9 to -18.6) | -1.42 (-1.67 to -1.13) | -26.5 (-30.0 to -24.6) | -4.24 (-4.78 to -3.76) |
| Medak | 25.3 (18.5-34.0) | 27.3 (22.2-32.6) | 22.4 (20.5-24.1) | -10.4 (-15.5 to -7.5) | -0.65 (-0.91 to -0.40) | -18.1 (-20.8 to -16.4) | -2.72 (-3.10 to -2.36) |
| Medchal Malkajgiri | 20.9 (15.1-28.0) | 21.4 (16.9-26.2) | 17.7 (16.3-19.2) | -14.6 (-19.3 to -12.2) | -0.93 (-1.18 to -0.69) | -17.4 (-20.6 to -15.2) | -2.63 (-3.07 to -2.17) |
| Nagarkurnool | 30.4 (23.4-39.3) | 20.8 (15.3-27.2) | 17.1 (15.9-18.7) | -43.8 (-46.5 to -42.4) | -3.29 (-3.50 to -3.09) | -17.1 (-22.2 to -15.0) | -2.63 (-3.35 to -2.13) |
| Nalgonda | 25.1 (19.2-32.6) | 18.6 (14.6-23.4) | 16.9 (15.3-18.4) | -32.3 (-35.0 to -29.8) | -2.24 (-2.42 to -1.98) | -8.6 (-12.0 to -5.5) | -1.23 (-1.65 to -0.66) |
| Nirmal | 35.4 (25.6-45.2) | 22.7 (17.9-28.5) | 21.5 (20.2-23.0) | -39.0 (-42.6 to -37.5) | -2.84 (-3.12 to -2.64) | -5.0 (-9.3 to -2.8) | -0.69 (-1.24 to -0.26) |
| Nizamabad | 30.5 (22.9-39.2) | 23.4 (19.0-28.1) | 21.0 (19.4-22.4) | -30.9 (-34.3 to -28.6) | -2.13 (-2.36 to -1.88) | -10.5 (-14.0 to -9.1) | -1.50 (-1.96 to -1.19) |
| Peddapalli | 21.9 (15.0-30.8) | 26.5 (19.2-34.4) | 18.9 (17.6-20.5) | -11.8 (-18.3 to -8.4) | -0.77 (-1.11 to -0.45) | -27.9 (-31.8 to -25.9) | -4.54 (-5.13 to -4.01) |
| Rajanna Sircilla | 23.1 (16.5-30.7) | 26.9 (21.4-33.0) | 22.3 (20.9-23.8) | -1.6 (-7.5 to 1.7) | -0.12 (-0.39 to 0.16) | -17.0 (-20.3 to -15.3) | -2.55 (-3.02 to -2.19) |
| Rangareddy | 22.8 (17.1-30.2) | 20.7 (16.7-24.7) | 16.4 (15.2-17.8) | -27.6 (-31.3 to -26.2) | -1.86 (-2.10 to -1.69) | -20.7 (-23.7 to -18.9) | -3.17 (-3.61 to -2.78) |
| Sangareddy | 28.4 (22.1-35.7) | 24.6 (20.6-28.7) | 19.4 (17.9-21.0) | -31.3 (-34.5 to -29.6) | -2.16 (-2.38 to -1.96) | -21.5 (-23.8 to -20.3) | -3.30 (-3.63 to -3.02) |
| Siddipet | 21.3 (15.5-29.0) | 27.9 (22.6-34.3) | 23.1 (21.6-24.5) | 10.2 (2.9 to 14.1) | 0.54 (0.22 to 0.83) | -17.3 (-20.0 to -15.7) | -2.59 (-2.97 to -2.24) |
| Suryapet | 25.3 (19.0-33.5) | 19.3 (14.8-24.9) | 17.6 (16.2-19.1) | -30.0 (-33.9 to -28.8) | -2.07 (-2.32 to -1.90) | -8.5 (-12.3 to -6.9) | -1.25 (-1.70 to -0.86) |
| Vikarabad | 28.6 (21.3-38.0) | 24.2 (20.1-29.3) | 18.2 (16.7-19.6) | -35.7 (-39.5 to -33.9) | -2.56 (-2.82 to -2.32) | -24.7 (-27.8 to -22.9) | -3.89 (-4.35 to -3.47) |
| Wanaparthy | 33.3 (24.8-43.9) | 25.0 (16.8-33.5) | 18.2 (16.7-19.8) | -45.0 (-48.2 to -42.9) | -3.43 (-3.68 to -3.15) | -25.9 (-31.6 to -23.7) | -4.23 (-5.08 to -3.61) |
| Warangal Rural | 19.1 (12.3-27.8) | 27.3 (19.1-37.6) | 20.3 (19.0-21.8) | 9.8 (1.3 to 12.7) | 0.46 (0.13 to 0.76) | -24.6 (-29.9 to -22.4) | -3.98 (-4.76 to -3.38) |
| Warangal Urban | 18.8 (12.8-26.0) | 27.6 (20.0-36.1) | 20.8 (19.7-22.3) | 13.3 (5.3 to 16.0) | 0.67 (0.36 to 0.93) | -24.0 (-28.2 to -21.8) | -3.83 (-4.44 to -3.27) |
| Yadadri Bhuvanagiri | 20.6 (15.3-28.1) | 22.4 (17.6-27.5) | 18.4 (16.7-19.9) | -9.6 (-14.5 to -7.5) | -0.60 (-0.85 to -0.39) | -18.0 (-21.5 to -16.1) | -2.72 (-3.23 to -2.32) |
| **Gujarat** | **21.9 (20.8-23.0)** | **19.9 (19.3-20.5)** | **19.3 (18.7-19.8)** | **-12.9 (-13.9 to -12.1)** | **-0.75 (-0.81 to -0.69)** | **-4.0 (-5.4 to -3.0)** | **-0.47 (-1.15 to 0.23)** |
| Ahmedabad | 19.9 (16.9-23.1) | 16.1 (13.2-19.6) | 15.1 (13.9-16.6) | -24.6 (-27.3 to -23.7) | -1.60 (-1.78 to -1.51) | -5.9 (-9.1 to -3.3) | -0.81 (-1.20 to -0.33) |
| Amreli | 22.6 (18.4-27.7) | 13.7 (10.4-17.1) | 17.7 (16.3-19.2) | -22.0 (-24.7 to -20.3) | -1.42 (-1.58 to -1.26) | 29.7 (22.8 to 32.5) | 3.76 (3.09 to 4.22) |
| Anand | 20.7 (17.3-24.4) | 17.9 (14.2-22.1) | 16.3 (14.7-17.8) | -21.6 (-24.1 to -19.7) | -1.38 (-1.53 to -1.21) | -8.9 (-12.5 to -5.8) | -1.27 (-1.73 to -0.70) |
| Arvalli | 22.8 (18.9-26.9) | 25.7 (22.2-29.5) | 22.9 (21.2-24.6) | 0.3 (-2.8 to 1.8) | 0.05 (-0.11 to 0.17) | -11.5 (-14.0 to -10.2) | -1.62 (-1.97 to -1.37) |
| Banaskantha | 23.6 (19.6-28.0) | 23.3 (19.6-27.3) | 19.0 (17.8-20.4) | -19.7 (-22.5 to -17.7) | -1.24 (-1.41 to -1.07) | -18.6 (-20.8 to -16.8) | -2.79 (-3.10 to -2.42) |
| Bharuch | 22.0 (18.7-26.3) | 25.2 (21.3-30.0) | 19.3 (17.8-21.2) | -12.4 (-14.9 to -10.1) | -0.74 (-0.88 to -0.56) | -23.6 (-26.1 to -21.8) | -3.66 (-4.04 to -3.28) |
| Bhavnagar | 22.6 (18.3-27.1) | 17.1 (13.2-21.3) | 18.1 (16.7-19.7) | -20.1 (-24.1 to -18.2) | -1.29 (-1.53 to -1.11) | 6.6 (2.2 to 9.6) | 0.93 (0.46 to 1.45) |
| Botad | 21.9 (17.4-27.0) | 15.2 (11.3-19.9) | 17.2 (15.7-19.2) | -21.3 (-26.5 to -18.4) | -1.40 (-1.72 to -1.12) | 14.3 (8.4 to 18.3) | 1.87 (1.30 to 2.56) |
| Chhotaudepur | 24.7 (20.2-29.2) | 27.7 (23.7-32.0) | 28.2 (26.7-29.9) | 14.2 (10.4 to 16.4) | 0.80 (0.64 to 0.95) | 1.4 (-1.9 to 3.3) | 0.30 (-0.13 to 0.61) |
| Dahod | 23.5 (19.8-28.2) | 28.1 (24.6-32.0) | 24.1 (22.9-25.8) | 2.2 (-0.6 to 4.7) | 0.16 (0.02 to 0.32) | -14.9 (-16.7 to -13.7) | -2.14 (-2.41 to -1.93) |
| Dang | 25.4 (19.7-31.5) | 28.5 (24.1-33.5) | 30.0 (28.2-31.8) | 19.1 (14.1 to 22.3) | 1.03 (0.83 to 1.24) | 5.0 (2.5 to 7.5) | 0.79 (0.49 to 1.17) |
| Devbhumi Dwarka | 19.0 (13.4-25.5) | 13.9 (9.2-20.5) | 17.3 (15.8-19.4) | -7.3 (-13.5 to -4.0) | -0.48 (-0.78 to -0.18) | 29.1 (19.0 to 32.4) | 3.52 (2.64 to 4.20) |
| Gandhinagar | 21.0 (17.6-24.8) | 18.7 (15.5-22.3) | 16.6 (15.2-18.1) | -21.5 (-24.4 to -20.1) | -1.38 (-1.56 to -1.24) | -11.4 (-14.6 to -9.2) | -1.63 (-2.07 to -1.21) |
| Gir Somnath | 22.9 (18.1-28.9) | 13.5 (9.9-18.3) | 18.2 (16.6-20.0) | -20.2 (-24.0 to -18.2) | -1.30 (-1.53 to -1.11) | 37.2 (30.4 to 42.0) | 4.54 (3.98 to 5.25) |
| Jamnagar | 20.1 (15.7-24.6) | 14.5 (10.7-19.5) | 19.4 (18.0-20.8) | -3.1 (-7.7 to -0.1) | -0.18 (-0.41 to 0.05) | 36.4 (28.6 to 42.6) | 4.44 (3.78 to 5.31) |
| Junagadh | 22.4 (17.5-27.0) | 14.2 (10.0-19.6) | 19.9 (18.5-21.5) | -11.2 (-15.1 to -8.7) | -0.68 (-0.89 to -0.47) | 43.1 (34.2 to 47.3) | 5.13 (4.41 to 5.79) |
| Kachchh | 19.6 (15.0-24.7) | 16.1 (11.5-21.8) | 20.6 (19.0-22.6) | 6.1 (0.8 to 8.7) | 0.34 (0.11 to 0.55) | 30.2 (24.1 to 33.7) | 3.77 (3.25 to 4.34) |
| Kheda | 21.4 (17.8-25.5) | 19.0 (15.6-23.4) | 17.6 (16.2-19.0) | -18.0 (-20.4 to -16.5) | -1.12 (-1.26 to -0.99) | -7.2 (-10.1 to -4.7) | -0.99 (-1.36 to -0.54) |
| Mahesana | 22.2 (18.8-25.9) | 20.4 (16.8-24.0) | 17.5 (16.2-19.1) | -21.6 (-24.2 to -20.0) | -1.37 (-1.54 to -1.23) | -14.3 (-17.5 to -12.2) | -2.08 (-2.54 to -1.68) |
| Mahisagar | 22.9 (19.0-27.2) | 25.3 (21.5-29.3) | 25.0 (23.5-26.7) | 9.1 (6.0 to 11.6) | 0.54 (0.40 to 0.70) | -1.4 (-4.1 to -0.2) | -0.11 (-0.45 to 0.11) |
| Morbi | 20.6 (16.6-25.1) | 14.1 (10.8-17.9) | 17.4 (15.9-19.1) | -15.6 (-19.9 to -14.0) | -0.97 (-1.22 to -0.81) | 24.2 (16.9 to 26.9) | 3.13 (2.38 to 3.58) |
| Narmada | 24.6 (20.1-29.7) | 28.1 (23.8-32.7) | 27.6 (26.2-29.0) | 12.6 (8.3 to 15.1) | 0.72 (0.52 to 0.88) | -2.0 (-4.6 to -0.3) | -0.20 (-0.53 to 0.10) |
| Navsari | 23.9 (19.5-28.3) | 25.0 (20.2-30.6) | 21.3 (19.8-22.9) | -11.3 (-14.5 to -9.5) | -0.67 (-0.85 to -0.52) | -14.9 (-18.9 to -12.6) | -2.20 (-2.79 to -1.75) |
| Panchmahal | 23.3 (19.5-27.2) | 24.0 (20.0-28.2) | 25.3 (24.1-27.1) | 8.8 (5.9 to 11.8) | 0.52 (0.39 to 0.71) | 5.5 (3.2 to 7.4) | 0.84 (0.58 to 1.16) |
| Patan | 22.2 (18.7-26.8) | 19.0 (15.8-22.8) | 17.7 (16.3-19.5) | -20.5 (-23.9 to -19.1) | -1.31 (-1.52 to -1.17) | -7.1 (-10.3 to -5.0) | -0.97 (-1.39 to -0.58) |
| Porbandar | 20.1 (14.7-26.5) | 13.6 (8.9-19.3) | 18.0 (16.3-19.9) | -9.2 (-14.5 to -6.6) | -0.58 (-0.85 to -0.34) | 37.1 (26.8 to 40.8) | 4.42 (3.57 to 5.12) |
| Rajkot | 20.6 (16.6-24.8) | 13.1 (9.5-17.3) | 16.7 (15.5-18.2) | -19.1 (-22.0 to -17.0) | -1.21 (-1.38 to -1.02) | 29.4 (21.5 to 33.0) | 3.68 (2.94 to 4.27) |
| Sabar Kantha | 22.0 (18.0-26.7) | 24.7 (21.2-28.9) | 20.5 (18.9-22.3) | -7.1 (-10.9 to -4.9) | -0.41 (-0.61 to -0.23) | -17.8 (-20.3 to -16.0) | -2.65 (-3.02 to -2.30) |
| Surat | 21.8 (18.2-26.4) | 24.9 (20.6-29.7) | 19.2 (17.8-21.1) | -12.0 (-14.8 to -9.7) | -0.72 (-0.87 to -0.53) | -23.1 (-25.8 to -21.7) | -3.58 (-4.00 to -3.25) |
| Surendranagar | 21.5 (17.8-25.9) | 15.2 (12.2-18.6) | 16.9 (15.3-18.9) | -21.3 (-24.6 to -19.6) | -1.37 (-1.57 to -1.21) | 11.8 (7.3 to 14.8) | 1.63 (1.14 to 2.12) |
| Tapi | 24.0 (19.9-28.2) | 28.0 (24.5-32.0) | 26.7 (25.3-28.5) | 11.1 (7.7 to 13.3) | 0.65 (0.49 to 0.79) | -5.2 (-7.2 to -3.4) | -0.65 (-0.91 to -0.34) |
| Vadodara | 21.8 (18.6-25.8) | 21.5 (18.4-25.3) | 20.0 (18.6-21.7) | -9.0 (-11.5 to -7.2) | -0.51 (-0.65 to -0.37) | -7.5 (-10.2 to -5.7) | -1.01 (-1.36 to -0.69) |
| Valsad | 25.5 (20.2-31.1) | 22.4 (16.4-29.4) | 21.3 (20.0-22.8) | -16.3 (-20.2 to -15.0) | -1.02 (-1.24 to -0.88) | -3.5 (-9.2 to -1.6) | -0.53 (-1.22 to -0.09) |
| **Manipur** | **9.0 (7.9-10.1)** | **8.8 (8.3-9.4)** | **7.7 (7.2-8.2)** | **-14.0 (-17.1 to -11.8)** | **-0.85 (-1.03 to -0.67)** | **-10.9 (-16.8 to -8.0)** | **-1.93 (-3.35 to -0.60)** |
| Bishnupur | 9.1 (6.8-12.0) | 8.4 (5.6-12.3) | 7.0 (6.4-7.8) | -22.0 (-25.8 to -19.6) | -1.45 (-1.66 to -1.21) | -14.0 (-20.2 to -10.9) | -2.26 (-3.00 to -1.48) |
| Chandel | 11.2 (8.2-14.5) | 10.1 (7.0-14.0) | 8.8 (7.9-9.9) | -21.4 (-25.8 to -19.5) | -1.41 (-1.67 to -1.19) | -11.5 (-17.4 to -9.1) | -1.82 (-2.53 to -1.20) |
| Churachandpur | 10.3 (7.9-13.2) | 8.9 (6.0-12.8) | 7.3 (7.0-7.7) | -29.0 (-32.7 to -26.7) | -1.97 (-2.22 to -1.73) | -16.5 (-22.0 to -14.5) | -2.62 (-3.32 to -2.04) |
| Imphal East | 8.2 (6.3-10.6) | 8.3 (5.5-12.0) | 7.3 (6.7-7.9) | -10.6 (-15.3 to -7.6) | -0.65 (-0.90 to -0.40) | -9.2 (-14.6 to -6.0) | -1.48 (-2.07 to -0.72) |
| Imphal West | 8.4 (6.3-11.3) | 8.3 (5.3-12.5) | 7.1 (6.1-8.4) | -14.2 (-20.2 to -11.0) | -0.93 (-1.25 to -0.61) | -10.7 (-18.1 to -8.8) | -1.80 (-2.64 to -1.16) |
| Jiribam | 13.3 (9.9-17.4) | 17.2 (12.2-23.9) | 15.8 (14.4-17.4) | 21.0 (13.1 to 24.4) | 1.09 (0.78 to 1.34) | -6.0 (-12.7 to -2.0) | -0.97 (-1.76 to -0.15) |
| Kakching | 9.0 (6.9-11.8) | 8.5 (5.7-12.1) | 7.4 (6.8-8.1) | -17.6 (-21.7 to -14.3) | -1.13 (-1.35 to -0.83) | -10.3 (-17.3 to -6.7) | -1.69 (-2.51 to -0.83) |
| Kamjong | 8.6 (6.1-11.9) | 9.2 (5.8-13.5) | 8.9 (8.1-9.9) | 5.7 (-2.4 to 9.0) | 0.28 (-0.08 to 0.56) | 0.3 (-7.8 to 4.5) | -0.14 (-1.01 to 0.76) |
| Kangpokpi | 8.3 (6.3-10.9) | 8.5 (5.5-12.4) | 7.5 (7.0-8.1) | -9.1 (-13.6 to -6.6) | -0.56 (-0.79 to -0.34) | -8.5 (-14.7 to -5.4) | -1.39 (-2.08 to -0.64) |
| Noney | 10.7 (8.5-13.4) | 11.2 (7.7-15.6) | 9.2 (8.7-9.8) | -13.8 (-17.1 to -11.7) | -0.85 (-1.03 to -0.66) | -16.1 (-21.2 to -13.9) | -2.51 (-3.18 to -1.95) |
| Pherzawl | 11.6 (9.0-14.6) | 11.1 (7.7-15.1) | 9.5 (8.8-10.2) | -18.2 (-22.3 to -15.6) | -1.16 (-1.40 to -0.92) | -13.3 (-19.3 to -10.7) | -2.07 (-2.85 to -1.45) |
| Senapati | 8.6 (6.7-10.8) | 8.9 (6.0-12.4) | 7.8 (7.4-8.1) | -9.1 (-12.8 to -6.7) | -0.54 (-0.73 to -0.34) | -10.7 (-17.0 to -8.2) | -1.69 (-2.46 to -1.07) |
| Tamenglong | 9.8 (7.8-12.1) | 10.7 (7.5-15.0) | 8.7 (8.3-9.1) | -11.6 (-14.5 to -9.4) | -0.70 (-0.85 to -0.52) | -17.7 (-22.8 to -15.5) | -2.77 (-3.45 to -2.22) |
| Tengnoupal | 9.3 (7.0-12.4) | 9.4 (6.3-13.6) | 8.5 (7.7-9.5) | -8.5 (-14.3 to -5.8) | -0.54 (-0.84 to -0.29) | -7.1 (-14.6 to -4.9) | -1.19 (-2.07 to -0.57) |
| Thoubal | 8.5 (6.4-11.2) | 8.3 (5.5-12.0) | 7.4 (7.0-8.1) | -11.4 (-16.5 to -9.4) | -0.71 (-0.98 to -0.51) | -7.5 (-14.5 to -4.0) | -1.23 (-2.05 to -0.44) |
| Ukhrul | 8.0 (5.9-10.9) | 8.3 (5.2-12.2) | 8.8 (8.1-9.6) | 11.4 (5.0 to 15.4) | 0.61 (0.34 to 0.90) | 9.3 (2.1 to 12.1) | 1.11 (0.44 to 1.77) |
| **Jammu and Kashmir**^†^ | **11.0 (10.0-12.1)** | **8.4 (7.9-8.9)** | **8.1 (7.7-8.6)** | **-26.2 (-28.3 to -24.8)** | **-1.72 (-1.86 to -1.58)** | **-2.3 (-6.1 to -0.3)** | **-0.41 (-1.67 to 0.86)** |
| Anantnag | 9.9 (7.2-13.2) | 7.9 (5.4-11.1) | 7.3 (6.9-7.8) | -25.6 (-30.0 to -23.4) | -1.73 (-2.00 to -1.48) | -5.1 (-12.0 to -2.6) | -0.83 (-1.66 to -0.23) |
| Badgam | 9.5 (6.7-12.8) | 8.2 (5.4-12.3) | 7.3 (6.6-8.0) | -22.2 (-26.7 to -19.3) | -1.48 (-1.73 to -1.18) | -8.7 (-14.9 to -6.2) | -1.44 (-2.11 to -0.75) |
| Bandipore | 8.5 (6.1-11.8) | 7.7 (5.3-10.8) | 5.5 (5.1-6.1) | -34.8 (-38.3 to -33.1) | -2.49 (-2.71 to -2.26) | -27.6 (-32.8 to -25.5) | -4.55 (-5.31 to -3.93) |
| Baramulla | 9.5 (6.7-12.9) | 8.6 (5.9-11.9) | 7.0 (6.6-7.4) | -25.6 (-29.8 to -23.7) | -1.74 (-1.97 to -1.50) | -17.4 (-23.4 to -14.8) | -2.76 (-3.56 to -2.10) |
| Doda | 13.1 (9.8-17.3) | 8.5 (6.2-11.2) | 9.3 (8.8-10.0) | -28.4 (-31.7 to -26.8) | -1.94 (-2.14 to -1.74) | 10.9 (4.8 to 14.6) | 1.44 (0.80 to 2.09) |
| Ganderbal | 8.4 (6.1-11.3) | 7.5 (5.1-10.8) | 5.9 (5.5-6.4) | -28.9 (-33.5 to -27.0) | -1.99 (-2.28 to -1.76) | -19.5 (-25.0 to -17.5) | -3.13 (-3.84 to -2.54) |
| Jammu | 14.6 (11.4-18.4) | 9.6 (7.8-11.6) | 10.6 (9.7-11.5) | -27.2 (-30.7 to -25.4) | -1.83 (-2.05 to -1.63) | 10.6 (7.5 to 13.7) | 1.49 (1.17 to 1.97) |
| Kargil | 8.8 (5.7-13.4) | 6.9 (4.4-10.2) | 5.8 (5.3-6.5) | -31.4 (-37.4 to -30.0) | -2.27 (-2.63 to -1.99) | -12.5 (-19.8 to -8.4) | -2.04 (-2.93 to -1.10) |
| Kathua | 14.4 (11.4-17.9) | 9.0 (7.1-11.0) | 9.6 (9.0-10.3) | -33.5 (-36.2 to -32.1) | -2.33 (-2.52 to -2.17) | 6.7 (2.9 to 9.2) | 0.97 (0.54 to 1.39) |
| Kishtwar | 11.1 (7.9-15.1) | 8.3 (5.8-11.3) | 8.5 (7.9-9.3) | -22.3 (-27.4 to -19.4) | -1.49 (-1.79 to -1.19) | 5.6 (-1.3 to 9.0) | 0.69 (-0.04 to 1.37) |
| Kulgam | 10.4 (7.8-13.4) | 8.3 (5.6-11.9) | 8.4 (7.8-9.0) | -18.7 (-23.0 to -16.9) | -1.21 (-1.45 to -1.02) | 3.8 (-2.6 to 7.2) | 0.43 (-0.23 to 1.13) |
| Kupwara | 9.2 (5.7-13.5) | 8.8 (5.8-12.5) | 6.0 (5.3-6.8) | -32.8 (-39.0 to -30.8) | -2.37 (-2.77 to -2.06) | -30.1 (-35.1 to -27.6) | -5.09 (-5.79 to -4.32) |
| Leh | 10.2 (6.0-17.1) | 7.8 (4.8-12.1) | 8.0 (6.8-9.5) | -16.8 (-26.9 to -14.6) | -1.25 (-1.74 to -0.85) | 7.7 (-2.6 to 10.4) | 0.78 (-0.23 to 1.56) |
| PoJK | 7.3 (4.4-11.3) | 10.0 (7.7-12.6) | 8.6 (6.6-11.1) | 14.6 (2.5 to 18.2) | 0.19 (-0.29 to 0.59) | -19.8 (-24.3 to -17.4) | -3.94 (-4.58 to -3.90) |
| Poonch | 10.5 (7.1-14.6) | 9.0 (6.8-11.6) | 11.1 (10.2-12.2) | 8.7 (1.1 to 12.1) | 0.44 (0.12 to 0.73) | 25.0 (19.2 to 29.1) | 3.20 (2.66 to 3.83) |
| Pulwama | 9.7 (7.3-12.8) | 8.0 (5.6-11.5) | 7.2 (6.8-7.7) | -25.5 (-29.6 to -23.0) | -1.71 (-1.97 to -1.45) | -8.3 (-13.9 to -5.6) | -1.31 (-1.95 to -0.67) |
| Rajouri | 11.5 (7.6-15.8) | 8.4 (6.4-11.0) | 12.1 (10.9-13.6) | 8.4 (1.3 to 11.1) | 0.41 (0.14 to 0.67) | 47.2 (40.8 to 51.1) | 5.61 (5.12 to 6.18) |
| Ramban | 12.5 (9.1-16.7) | 8.8 (6.1-12.2) | 9.7 (8.9-10.7) | -21.0 (-25.8 to -18.8) | -1.40 (-1.66 to -1.15) | 12.9 (5.2 to 16.5) | 1.66 (0.86 to 2.33) |
| Reasi | 11.9 (8.9-15.3) | 8.1 (6.1-10.7) | 10.2 (9.6-10.9) | -13.3 (-17.1 to -11.1) | -0.83 (-1.03 to -0.62) | 27.1 (21.1 to 30.8) | 3.46 (2.89 to 4.03) |
| Samba | 15.7 (12.2-19.8) | 9.9 (7.8-12.3) | 9.4 (8.4-10.4) | -40.4 (-43.6 to -38.7) | -2.97 (-3.22 to -2.74) | -5.2 (-9.6 to -2.4) | -0.73 (-1.27 to -0.21) |
| Shopian | 10.0 (7.4-13.3) | 8.2 (5.6-11.8) | 7.9 (7.4-8.7) | -20.2 (-24.0 to -18.4) | -1.33 (-1.52 to -1.12) | -0.5 (-7.9 to 2.6) | -0.20 (-1.01 to 0.51) |
| Srinagar | 9.3 (6.8-12.6) | 8.0 (5.2-11.5) | 6.6 (5.9-7.4) | -28.4 (-33.5 to -26.2) | -1.97 (-2.28 to -1.70) | -15.4 (-21.2 to -14.1) | -2.51 (-3.18 to -1.99) |
| Udhampur | 13.6 (10.7-16.9) | 8.4 (6.4-10.8) | 8.6 (8.2-9.2) | -36.5 (-40.0 to -35.1) | -2.59 (-2.86 to -2.42) | 3.0 (-1.6 to 5.3) | 0.43 (-0.09 to 0.87) |
| **Haryana** | **12.5 (11.3-13.8)** | **16.2 (15.4-17.0)** | **12.5 (11.8-13.2)** | **-0.1 (-2.4 to 1.7)** | **0.04 (-0.08 to 0.16)** | **-23.1 (-24.8 to -21.8)** | **-3.66 (-4.90 to -2.53)** |
| Ambala | 14.2 (11.2-17.6) | 14.2 (11.2-17.6) | 12.1 (11.8-12.6) | -14.1 (-17.4 to -12.0) | -0.87 (-1.05 to -0.69) | -14.3 (-17.5 to -12.6) | -2.11 (-2.54 to -1.75) |
| Bhiwani | 11.3 (8.6-14.3) | 17.5 (13.3-22.4) | 12.2 (11.7-12.7) | 8.9 (3.3 to 11.4) | 0.50 (0.25 to 0.69) | -30.1 (-33.6 to -28.0) | -4.91 (-5.47 to -4.40) |
| Charkhi Dadri | 12.0 (9.6-14.7) | 16.7 (13.4-20.5) | 11.6 (11.2-12.0) | -3.2 (-7.4 to -0.7) | -0.17 (-0.39 to 0.02) | -30.9 (-34.0 to -29.3) | -5.04 (-5.56 to -4.64) |
| Faridabad | 14.9 (11.7-19.0) | 13.0 (10.0-16.2) | 11.2 (10.3-12.3) | -24.5 (-27.7 to -22.2) | -1.62 (-1.81 to -1.39) | -13.0 (-16.7 to -10.2) | -1.95 (-2.41 to -1.37) |
| Fatehabad | 10.8 (8.0-14.0) | 19.2 (14.7-24.6) | 12.8 (12.3-13.4) | 20.9 (13.8 to 25.5) | 1.10 (0.81 to 1.39) | -32.9 (-36.2 to -31.8) | -5.45 (-6.01 to -5.12) |
| Gurugram | 13.2 (9.9-16.9) | 15.2 (11.7-19.4) | 11.8 (10.9-12.8) | -10.6 (-14.9 to -8.5) | -0.65 (-0.87 to -0.46) | -22.4 (-25.6 to -19.6) | -3.49 (-3.95 to -2.90) |
| Hisar | 10.3 (7.6-13.6) | 17.9 (13.4-23.3) | 13.0 (12.4-13.7) | 28.5 (20.4 to 32.0) | 1.46 (1.15 to 1.69) | -27.0 (-30.4 to -24.8) | -4.33 (-4.85 to -3.81) |
| Jhajjar | 11.9 (8.9-15.5) | 15.5 (11.9-19.5) | 11.5 (10.9-12.0) | -2.5 (-7.7 to -0.1) | -0.15 (-0.41 to 0.05) | -25.5 (-28.6 to -24.0) | -4.04 (-4.51 to -3.66) |
| Jind | 10.1 (7.5-13.5) | 16.8 (12.7-21.2) | 14.4 (13.8-15.0) | 45.0 (38.0 to 50.6) | 2.18 (1.95 to 2.48) | -13.8 (-17.0 to -12.0) | -2.04 (-2.46 to -1.65) |
| Kaithal | 11.2 (8.6-14.7) | 16.3 (12.6-20.6) | 14.8 (14.1-15.5) | 33.3 (27.0 to 37.8) | 1.69 (1.46 to 1.95) | -8.7 (-12.6 to -6.9) | -1.25 (-1.75 to -0.86) |
| Karnal | 11.8 (9.2-15.2) | 15.8 (12.4-19.5) | 13.7 (13.1-14.3) | 16.5 (10.7 to 19.2) | 0.90 (0.65 to 1.09) | -13.3 (-16.6 to -10.8) | -1.95 (-2.40 to -1.47) |
| Kurukshetra | 13.4 (10.8-16.6) | 15.6 (12.5-18.9) | 13.8 (13.3-14.3) | 3.3 (-0.1 to 5.9) | 0.21 (0.05 to 0.39) | -11.4 (-14.6 to -9.6) | -1.65 (-2.07 to -1.28) |
| Mahendragarh | 13.4 (10.4-16.6) | 16.8 (12.9-21.3) | 10.9 (10.1-11.5) | -18.5 (-22.5 to -16.5) | -1.17 (-1.41 to -0.99) | -35.4 (-38.1 to -33.8) | -5.95 (-6.42 to -5.53) |
| Nuh | 14.5 (11.1-18.7) | 17.7 (13.8-22.6) | 13.5 (12.9-14.1) | -6.3 (-10.4 to -4.1) | -0.38 (-0.58 to -0.19) | -23.5 (-26.7 to -21.2) | -3.68 (-4.16 to -3.17) |
| Palwal | 16.2 (12.5-20.6) | 13.8 (10.8-17.6) | 11.8 (11.2-12.6) | -26.8 (-30.5 to -24.8) | -1.79 (-2.04 to -1.58) | -14.1 (-18.1 to -11.8) | -2.10 (-2.65 to -1.62) |
| Panchkula | 15.1 (11.0-19.8) | 13.3 (9.9-17.6) | 11.6 (10.4-12.8) | -22.7 (-27.0 to -20.2) | -1.51 (-1.76 to -1.24) | -12.2 (-17.3 to -9.6) | -1.85 (-2.51 to -1.27) |
| Panipat | 11.4 (8.5-15.2) | 14.9 (11.3-19.2) | 12.7 (11.6-14.0) | 12.8 (6.5 to 16.3) | 0.69 (0.43 to 0.95) | -13.9 (-18.0 to -11.1) | -2.09 (-2.62 to -1.51) |
| Rewari | 13.0 (10.3-16.4) | 16.8 (13.2-20.9) | 11.8 (11.0-12.6) | -9.1 (-13.0 to -6.8) | -0.55 (-0.75 to -0.35) | -29.9 (-32.7 to -28.2) | -4.85 (-5.30 to -4.42) |
| Rohtak | 10.8 (8.4-13.9) | 15.9 (12.4-20.4) | 12.5 (11.7-13.5) | 17.0 (11.3 to 20.5) | 0.92 (0.69 to 1.15) | -20.7 (-24.2 to -18.9) | -3.20 (-3.71 to -2.78) |
| Sirsa | 13.2 (9.4-17.9) | 25.2 (18.8-32.2) | 14.1 (13.3-14.9) | 8.7 (0.3 to 12.2) | 0.46 (0.07 to 0.73) | -44.0 (-46.6 to -42.7) | -7.84 (-8.34 to -7.42) |
| Sonipat | 11.6 (9.1-14.6) | 15.1 (11.9-18.6) | 12.3 (11.9-12.8) | 7.2 (2.6 to 9.8) | 0.42 (0.21 to 0.60) | -18.3 (-21.3 to -16.6) | -2.76 (-3.18 to -2.39) |
| Yamunanagar | 13.5 (10.2-17.2) | 13.8 (10.7-17.7) | 10.9 (10.4-11.4) | -18.7 (-22.6 to -16.2) | -1.20 (-1.42 to -0.97) | -20.7 (-23.8 to -17.8) | -3.21 (-3.62 to -2.59) |
| **Uttarakhand** | **16.4 (14.6-18.5)** | **12.4 (11.7-13.1)** | **11.1 (10.5-11.7)** | **-32.9 (-35.1 to -31.4)** | **-2.26 (-2.42 to -2.11)** | **-10.7 (-13.4 to -8.6)** | **-1.60 (-2.84 to -0.38)** |
| Almora | 16.7 (12.0-22.3) | 12.6 (9.4-16.3) | 9.3 (8.9-9.8) | -44.2 (-47.3 to -42.7) | -3.34 (-3.59 to -3.12) | -25.7 (-30.3 to -24.0) | -4.11 (-4.83 to -3.66) |
| Bageshwar | 19.5 (13.7-27.1) | 14.5 (9.9-19.9) | 11.2 (10.2-12.1) | -42.0 (-46.0 to -40.2) | -3.15 (-3.45 to -2.88) | -21.6 (-27.0 to -19.7) | -3.43 (-4.21 to -2.91) |
| Chamoli | 19.0 (12.1-27.8) | 11.8 (7.5-17.2) | 9.7 (8.5-11.1) | -47.3 (-52.0 to -46.0) | -3.75 (-4.11 to -3.46) | -15.3 (-21.0 to -11.6) | -2.45 (-3.14 to -1.58) |
| Champawat | 18.0 (13.5-22.9) | 16.7 (12.5-21.8) | 10.4 (9.7-11.2) | -42.2 (-45.1 to -41.1) | -3.13 (-3.36 to -2.97) | -37.5 (-40.6 to -36.0) | -6.41 (-6.94 to -5.97) |
| Dehradun | 14.3 (10.5-18.9) | 11.0 (8.1-14.6) | 13.4 (12.4-14.6) | -5.2 (-10.7 to -3.7) | -0.32 (-0.60 to -0.16) | 24.1 (16.2 to 27.6) | 3.07 (2.29 to 3.66) |
| Haridwar | 14.2 (10.4-18.8) | 11.1 (8.5-14.5) | 11.3 (10.9-11.8) | -19.9 (-24.1 to -18.1) | -1.29 (-1.53 to -1.10) | 2.9 (-0.5 to 6.1) | 0.43 (0.07 to 0.99) |
| Nainital | 16.3 (11.9-21.6) | 12.7 (10.0-16.1) | 8.5 (8.2-8.8) | -47.7 (-50.5 to -46.4) | -3.70 (-3.94 to -3.50) | -32.9 (-36.2 to -30.8) | -5.44 (-6.01 to -4.93) |
| Pauri Garhwal | 15.9 (11.0-21.7) | 11.4 (8.2-15.1) | 11.6 (10.9-12.4) | -25.8 (-30.2 to -23.7) | -1.75 (-2.01 to -1.50) | 3.6 (-2.5 to 5.9) | 0.48 (-0.21 to 0.95) |
| Pithoragarh | 24.7 (17.8-31.9) | 16.2 (10.7-22.2) | 12.7 (11.6-14.3) | -48.5 (-52.0 to -47.1) | -3.80 (-4.11 to -3.57) | -20.0 (-26.4 to -16.7) | -3.21 (-4.10 to -2.41) |
| Rudraprayag | 17.1 (10.7-25.9) | 11.2 (6.9-16.5) | 10.1 (9.1-11.2) | -38.2 (-43.9 to -36.6) | -2.87 (-3.25 to -2.56) | -6.4 (-13.3 to -3.9) | -1.10 (-1.85 to -0.42) |
| Tehri Garhwal | 15.8 (10.8-21.7) | 11.2 (7.7-15.6) | 13.7 (13.1-14.4) | -11.3 (-16.6 to -8.2) | -0.73 (-0.99 to -0.44) | 25.4 (17.0 to 28.8) | 3.19 (2.39 to 3.79) |
| Udham Singh Nagar | 18.0 (14.2-22.6) | 14.5 (11.9-17.6) | 9.6 (9.3-9.9) | -47.0 (-49.2 to -46.2) | -3.61 (-3.79 to -3.48) | -34.2 (-36.8 to -32.9) | -5.66 (-6.13 to -5.33) |
| Uttarkashi | 16.9 (10.9-24.0) | 11.3 (7.2-15.8) | 12.3 (11.4-13.1) | -24.9 (-30.0 to -23.3) | -1.72 (-2.00 to -1.48) | 12.6 (4.5 to 15.2) | 1.57 (0.77 to 2.17) |
| **Tamil Nadu** | **20.5 (19.7-21.5)** | **21.1 (20.5-21.8)** | **18.4 (17.8-19.0)** | **-11.4 (-12.2 to -10.6)** | **-0.65 (-0.70 to -0.59)** | **-13.7 (-14.7 to -12.8)** | **-1.97 (-2.71 to -1.23)** |
| Ariyalur | 21.3 (18.4-25.0) | 21.0 (17.6-24.8) | 19.2 (18.3-20.1) | -10.7 (-13.0 to -8.8) | -0.62 (-0.75 to -0.47) | -9.0 (-12.2 to -7.2) | -1.25 (-1.68 to -0.91) |
| Chennai | 16.9 (12.4-22.1) | 23.4 (18.2-28.6) | 16.4 (15.2-18.2) | -2.0 (-7.4 to 0.4) | -0.14 (-0.39 to 0.08) | -29.8 (-33.5 to -28.0) | -4.88 (-5.47 to -4.39) |
| Coimbatore | 20.0 (17.1-23.5) | 18.7 (15.3-22.7) | 17.4 (16.2-18.7) | -13.6 (-16.5 to -11.5) | -0.81 (-0.99 to -0.65) | -7.5 (-10.3 to -5.3) | -1.02 (-1.39 to -0.63) |
| Cuddalore | 20.7 (17.2-25.2) | 20.4 (16.2-24.9) | 17.6 (16.6-18.7) | -14.9 (-17.1 to -13.6) | -0.91 (-1.03 to -0.79) | -13.4 (-16.6 to -11.3) | -1.95 (-2.40 to -1.54) |
| Dharmapuri | 19.7 (15.9-23.8) | 18.9 (15.3-23.0) | 20.6 (19.2-22.0) | 4.7 (1.6 to 7.2) | 0.29 (0.15 to 0.46) | 9.0 (4.9 to 11.7) | 1.29 (0.83 to 1.73) |
| Dindigul | 22.9 (19.2-27.1) | 22.8 (18.8-27.3) | 18.9 (17.7-20.4) | -17.8 (-20.7 to -16.4) | -1.11 (-1.28 to -0.98) | -17.2 (-20.2 to -15.5) | -2.57 (-3.00 to -2.22) |
| Erode | 22.6 (18.9-26.7) | 20.5 (16.8-24.4) | 19.1 (18.0-20.2) | -15.8 (-18.4 to -14.4) | -0.97 (-1.12 to -0.84) | -6.9 (-10.3 to -4.4) | -0.93 (-1.38 to -0.50) |
| Kanchipuram | 17.7 (14.3-22.2) | 22.2 (18.2-26.7) | 17.1 (15.5-18.7) | -2.9 (-6.4 to -1.0) | -0.16 (-0.33 to 0.00) | -23.0 (-25.9 to -21.0) | -3.58 (-4.01 to -3.13) |
| Kanniyakumari | 15.7 (11.1-21.1) | 13.8 (10.4-17.4) | 14.4 (13.2-16.2) | -6.9 (-13.6 to -5.2) | -0.45 (-0.79 to -0.25) | 5.7 (0.1 to 8.9) | 0.78 (0.15 to 1.35) |
| Karur | 22.5 (19.1-26.5) | 21.9 (18.2-25.7) | 19.1 (18.0-20.3) | -15.9 (-18.3 to -14.5) | -0.97 (-1.11 to -0.85) | -13.4 (-16.4 to -11.4) | -1.93 (-2.37 to -1.55) |
| Krishnagiri | 18.5 (15.6-22.0) | 16.9 (13.7-20.4) | 19.6 (18.6-20.6) | 5.6 (2.3 to 7.9) | 0.35 (0.19 to 0.50) | 15.9 (12.7 to 18.2) | 2.19 (1.85 to 2.54) |
| Madurai | 23.1 (19.6-27.2) | 24.8 (21.2-29.5) | 17.8 (16.6-19.2) | -23.6 (-26.2 to -22.0) | -1.52 (-1.69 to -1.37) | -28.8 (-30.7 to -27.4) | -4.60 (-4.91 to -4.29) |
| Nagapattinam | 20.9 (16.1-26.6) | 17.1 (13.1-22.2) | 17.4 (16.0-18.9) | -16.4 (-20.7 to -14.2) | -1.03 (-1.28 to -0.83) | 2.7 (-2.4 to 5.2) | 0.38 (-0.20 to 0.86) |
| Namakkal | 22.9 (18.7-27.5) | 20.8 (16.6-25.3) | 18.4 (17.1-19.9) | -19.8 (-22.1 to -18.5) | -1.25 (-1.39 to -1.12) | -11.5 (-14.5 to -9.4) | -1.67 (-2.05 to -1.25) |
| Perambalur | 21.7 (18.2-25.4) | 23.2 (19.4-27.1) | 19.8 (18.7-21.1) | -9.0 (-11.4 to -7.5) | -0.52 (-0.65 to -0.39) | -15.0 (-18.1 to -13.6) | -2.20 (-2.64 to -1.90) |
| Pudukkottai | 23.1 (19.0-27.6) | 24.1 (20.0-28.4) | 20.4 (19.2-21.9) | -11.8 (-14.7 to -10.1) | -0.70 (-0.86 to -0.56) | -15.5 (-18.2 to -14.1) | -2.27 (-2.67 to -1.99) |
| Ramanathapuram | 24.9 (20.0-30.8) | 24.4 (20.0-30.0) | 19.4 (17.8-20.9) | -22.2 (-25.1 to -20.2) | -1.43 (-1.61 to -1.25) | -20.7 (-23.3 to -19.1) | -3.16 (-3.54 to -2.81) |
| Salem | 20.6 (17.2-23.8) | 19.5 (16.1-22.9) | 18.5 (17.5-19.5) | -10.3 (-12.7 to -8.7) | -0.60 (-0.73 to -0.47) | -5.4 (-8.5 to -3.5) | -0.70 (-1.11 to -0.36) |
| Sivaganga | 25.1 (20.7-30.2) | 26.5 (22.4-30.7) | 19.6 (18.1-21.0) | -22.3 (-25.0 to -21.0) | -1.43 (-1.60 to -1.30) | -26.6 (-29.0 to -25.3) | -4.20 (-4.59 to -3.90) |
| Thanjavur | 21.0 (17.3-25.3) | 19.7 (16.4-23.9) | 18.5 (17.5-19.9) | -11.9 (-14.5 to -10.6) | -0.71 (-0.85 to -0.60) | -6.0 (-8.9 to -3.6) | -0.80 (-1.17 to -0.38) |
| The Nilgiris | 16.5 (13.1-20.7) | 15.8 (12.4-19.8) | 16.3 (15.1-17.8) | -0.7 (-4.6 to 2.4) | -0.03 (-0.22 to 0.20) | 4.0 (-0.7 to 6.1) | 0.59 (0.04 to 0.98) |
| Theni | 19.9 (16.3-24.1) | 20.4 (16.5-24.7) | 16.5 (15.3-17.7) | -17.4 (-20.4 to -15.8) | -1.09 (-1.26 to -0.93) | -19.2 (-22.0 to -17.6) | -2.91 (-3.31 to -2.56) |
| Thiruvallur | 18.7 (15.1-23.3) | 25.2 (20.8-30.1) | 18.2 (16.7-19.9) | -2.8 (-6.4 to -0.6) | -0.15 (-0.33 to 0.02) | -28.3 (-31.1 to -26.7) | -4.53 (-4.98 to -4.16) |
| Thiruvarur | 21.4 (17.0-27.0) | 17.9 (13.9-22.4) | 17.8 (16.7-19.1) | -16.4 (-20.7 to -13.7) | -1.02 (-1.28 to -0.79) | 0.0 (-4.4 to 2.7) | 0.03 (-0.49 to 0.52) |
| Thoothukudi | 22.0 (17.3-26.9) | 19.2 (15.3-23.8) | 16.9 (15.3-18.4) | -23.4 (-27.0 to -21.6) | -1.53 (-1.76 to -1.35) | -12.0 (-15.4 to -9.6) | -1.76 (-2.19 to -1.27) |
| Tiruchirappalli | 21.3 (17.6-25.2) | 22.6 (18.9-26.9) | 19.6 (18.4-20.8) | -8.1 (-10.9 to -5.8) | -0.46 (-0.61 to -0.29) | -13.6 (-16.3 to -11.9) | -1.97 (-2.34 to -1.63) |
| Tirunelveli | 19.0 (15.5-23.2) | 17.7 (14.6-21.2) | 15.9 (14.9-17.1) | -16.3 (-19.1 to -14.3) | -1.01 (-1.17 to -0.84) | -10.4 (-12.7 to -7.9) | -1.47 (-1.76 to -1.02) |
| Tiruppur | 23.3 (19.8-27.5) | 21.6 (17.4-26.0) | 18.9 (17.7-20.3) | -19.1 (-21.7 to -17.9) | -1.20 (-1.35 to -1.08) | -12.4 (-16.4 to -10.4) | -1.80 (-2.36 to -1.41) |
| Tiruvannamalai | 20.2 (17.2-23.3) | 21.9 (18.7-25.6) | 21.3 (20.1-22.5) | 5.2 (2.1 to 7.3) | 0.33 (0.18 to 0.47) | -2.9 (-5.6 to -1.1) | -0.33 (-0.67 to -0.02) |
| Vellore | 19.5 (15.8-23.2) | 20.5 (17.1-24.6) | 22.6 (21.2-24.2) | 15.6 (12.3 to 18.6) | 0.88 (0.74 to 1.06) | 9.8 (6.3 to 11.9) | 1.42 (1.01 to 1.75) |
| Viluppuram | 20.5 (17.3-24.6) | 22.4 (18.2-26.9) | 18.8 (17.7-20.1) | -8.8 (-10.9 to -6.3) | -0.50 (-0.61 to -0.32) | -16.3 (-19.5 to -14.6) | -2.43 (-2.88 to -2.07) |
| Virudhunagar | 22.8 (18.6-27.2) | 23.5 (19.5-28.3) | 17.5 (16.2-18.9) | -23.5 (-26.4 to -21.7) | -1.53 (-1.71 to -1.36) | -25.9 (-28.3 to -24.4) | -4.08 (-4.45 to -3.73) |
| **Mizoram** | **11.1 (9.9-12.3)** | **14.1 (13.5-14.8)** | **13.0 (12.5-13.6)** | **16.8 (13.3 to 19.3)** | **0.94 (0.79 to 1.09)** | **-7.6 (-11.7 to -6.0)** | **-1.18 (-2.18 to -0.11)** |
| Aizawl | 9.8 (8.0-11.9) | 12.8 (9.1-16.7) | 10.7 (10.2-11.3) | 9.2 (5.0 to 12.5) | 0.54 (0.35 to 0.75) | -15.9 (-21.0 to -14.5) | -2.44 (-3.14 to -2.06) |
| Champhai | 10.4 (8.2-13.4) | 12.3 (9.0-16.5) | 11.0 (10.3-11.8) | 6.4 (2.7 to 9.5) | 0.37 (0.21 to 0.59) | -9.5 (-14.3 to -7.2) | -1.44 (-2.02 to -0.91) |
| Kolasib | 10.8 (8.8-13.0) | 16.6 (12.3-21.1) | 13.5 (13.0-14.3) | 25.3 (21.4 to 28.0) | 1.35 (1.19 to 1.51) | -17.9 (-22.1 to -16.8) | -2.74 (-3.33 to -2.43) |
| Lawngtlai | 13.9 (10.5-18.0) | 16.7 (12.8-22.1) | 18.0 (16.2-19.6) | 30.8 (24.1 to 34.2) | 1.57 (1.33 to 1.79) | 8.5 (3.8 to 12.2) | 1.17 (0.67 to 1.79) |
| Lunglei | 12.3 (9.5-15.7) | 13.6 (10.1-17.3) | 14.3 (13.2-15.4) | 17.2 (11.4 to 19.6) | 0.93 (0.69 to 1.11) | 6.4 (0.5 to 9.6) | 0.88 (0.22 to 1.45) |
| Mamit | 11.7 (9.5-14.1) | 16.0 (12.4-20.2) | 14.7 (13.7-15.9) | 25.6 (20.6 to 28.1) | 1.36 (1.15 to 1.51) | -7.8 (-12.5 to -5.9) | -1.12 (-1.72 to -0.72) |
| Saiha | 11.6 (8.2-16.2) | 14.0 (9.8-18.9) | 14.5 (13.1-16.1) | 28.4 (20.0 to 31.7) | 1.43 (1.13 to 1.68) | 6.2 (-0.3 to 9.2) | 0.80 (0.10 to 1.39) |
| Serchhip | 10.6 (7.9-13.5) | 12.0 (8.8-15.5) | 11.3 (10.3-12.3) | 8.3 (2.7 to 11.0) | 0.46 (0.21 to 0.67) | -4.1 (-9.6 to -1.0) | -0.63 (-1.27 to 0.00) |
| **Maharashtra** | **21.9 (21.0-22.9)** | **22.5 (21.9-23.2)** | **18.7 (18.0-19.4)** | **-15.6 (-16.7 to -14.8)** | **-0.93 (-1.00 to -0.87)** | **-17.9 (-18.9 to -16.8)** | **-2.67 (-3.44 to -1.92)** |
| Ahmednagar | 23.0 (19.7-27.1) | 17.9 (14.9-21.2) | 18.4 (17.2-19.7) | -20.5 (-22.6 to -19.3) | -1.29 (-1.42 to -1.18) | 2.6 (-1.3 to 4.5) | 0.44 (-0.05 to 0.77) |
| Akola | 23.8 (19.5-28.2) | 24.0 (19.0-29.9) | 18.7 (17.6-19.8) | -21.7 (-23.9 to -20.2) | -1.38 (-1.52 to -1.25) | -21.9 (-25.0 to -19.8) | -3.39 (-3.84 to -2.93) |
| Amravati | 21.2 (17.1-25.2) | 26.2 (20.8-32.4) | 19.7 (18.4-21.0) | -6.9 (-10.4 to -5.1) | -0.39 (-0.58 to -0.25) | -24.8 (-27.9 to -23.4) | -3.89 (-4.38 to -3.56) |
| Aurangabad | 23.1 (19.3-27.0) | 17.5 (13.2-22.4) | 17.7 (16.8-18.9) | -23.7 (-26.4 to -22.6) | -1.53 (-1.71 to -1.42) | 2.3 (-3.2 to 4.4) | 0.34 (-0.31 to 0.76) |
| Beed | 22.7 (18.9-26.6) | 16.8 (13.9-20.4) | 19.1 (18.0-20.4) | -16.3 (-18.8 to -14.5) | -1.00 (-1.15 to -0.85) | 14.0 (9.7 to 17.0) | 1.93 (1.46 to 2.39) |
| Bhandara | 22.5 (18.3-27.3) | 30.6 (25.8-35.6) | 19.9 (18.9-21.0) | -11.7 (-15.0 to -9.8) | -0.69 (-0.88 to -0.54) | -35.5 (-37.3 to -34.6) | -5.92 (-6.24 to -5.68) |
| Buldhana | 24.9 (20.9-29.5) | 21.6 (16.7-26.8) | 17.9 (16.8-18.9) | -28.6 (-30.7 to -27.3) | -1.91 (-2.05 to -1.78) | -17.3 (-20.6 to -14.7) | -2.60 (-3.07 to -2.08) |
| Chandrapur | 21.7 (17.5-27.1) | 29.6 (23.9-36.1) | 22.4 (21.1-23.9) | 3.6 (-0.2 to 6.6) | 0.22 (0.04 to 0.43) | -24.6 (-27.4 to -23.8) | -3.84 (-4.29 to -3.63) |
| Dhule | 22.6 (18.4-27.2) | 29.3 (22.8-36.6) | 24.9 (23.6-26.5) | 10.3 (6.1 to 13.2) | 0.60 (0.40 to 0.78) | -14.8 (-18.4 to -13.3) | -2.19 (-2.70 to -1.86) |
| Gadchiroli | 20.0 (14.8-26.1) | 31.0 (23.9-37.9) | 21.0 (19.6-22.2) | 6.0 (1.1 to 9.1) | 0.33 (0.12 to 0.57) | -32.4 (-35.4 to -31.4) | -5.32 (-5.84 to -5.05) |
| Gondia | 22.8 (18.2-27.9) | 32.7 (27.2-38.4) | 20.5 (19.1-21.8) | -10.2 (-14.0 to -8.6) | -0.61 (-0.81 to -0.46) | -38.0 (-40.0 to -36.5) | -6.44 (-6.82 to -6.07) |
| Hingoli | 25.1 (20.1-30.5) | 19.3 (15.6-24.1) | 20.0 (18.8-21.3) | -20.3 (-23.3 to -18.7) | -1.29 (-1.47 to -1.14) | 3.8 (0.3 to 6.5) | 0.59 (0.19 to 1.04) |
| Jalgaon | 24.4 (20.5-28.3) | 24.8 (19.2-30.9) | 20.0 (18.8-21.3) | -18.3 (-21.5 to -16.3) | -1.14 (-1.34 to -0.97) | -19.1 (-21.7 to -17.3) | -2.91 (-3.27 to -2.52) |
| Jalna | 23.6 (19.7-27.7) | 17.1 (13.2-21.8) | 18.2 (17.4-19.3) | -23.4 (-25.7 to -22.2) | -1.51 (-1.65 to -1.39) | 6.7 (1.5 to 9.1) | 0.96 (0.36 to 1.39) |
| Kolhapur | 16.3 (13.2-19.9) | 23.6 (20.1-27.9) | 16.0 (14.9-17.2) | -2.1 (-5.8 to 0.2) | -0.10 (-0.29 to 0.07) | -32.9 (-34.9 to -31.1) | -5.40 (-5.75 to -4.99) |
| Latur | 23.1 (19.4-27.1) | 19.2 (15.9-22.9) | 17.8 (16.9-18.8) | -23.4 (-25.8 to -22.0) | -1.51 (-1.67 to -1.38) | -7.7 (-10.4 to -6.0) | -1.06 (-1.40 to -0.74) |
| Mumbai City | 17.7 (12.8-23.7) | 17.0 (11.2-23.7) | 14.3 (13.2-16.4) | -17.7 (-23.9 to -14.5) | -1.20 (-1.52 to -0.85) | -13.2 (-22.2 to -10.0) | -2.20 (-3.34 to -1.35) |
| Mumbai Suburban | 18.8 (14.7-23.1) | 19.0 (14.0-24.7) | 16.0 (14.7-17.4) | -14.5 (-18.1 to -12.5) | -0.90 (-1.10 to -0.72) | -15.1 (-20.2 to -13.3) | -2.31 (-3.01 to -1.85) |
| Nagpur | 20.8 (16.1-26.2) | 25.5 (21.1-30.5) | 17.6 (16.3-19.2) | -14.9 (-17.8 to -12.7) | -0.93 (-1.07 to -0.73) | -31.3 (-33.9 to -29.6) | -5.09 (-5.54 to -4.71) |
| Nanded | 22.8 (18.8-27.5) | 21.9 (18.1-26.4) | 18.9 (17.8-20.0) | -17.6 (-20.5 to -15.9) | -1.09 (-1.27 to -0.94) | -14.0 (-16.8 to -12.1) | -2.03 (-2.43 to -1.66) |
| Nandurbar | 21.6 (17.9-25.7) | 35.5 (29.2-42.0) | 27.3 (26.0-28.6) | 26.2 (21.4 to 29.0) | 1.40 (1.20 to 1.55) | -23.5 (-26.1 to -22.5) | -3.63 (-4.04 to -3.40) |
| Nashik | 22.2 (18.7-25.9) | 24.3 (20.2-28.6) | 21.0 (19.7-22.3) | -5.8 (-8.3 to -4.2) | -0.31 (-0.45 to -0.19) | -13.8 (-17.2 to -12.5) | -2.00 (-2.50 to -1.72) |
| Osmanabad | 23.2 (19.1-27.8) | 19.3 (15.7-22.8) | 16.8 (15.9-17.9) | -27.9 (-30.6 to -26.1) | -1.85 (-2.04 to -1.68) | -12.9 (-16.1 to -11.3) | -1.86 (-2.30 to -1.55) |
| Palghar | 22.3 (18.6-26.5) | 26.2 (20.2-32.4) | 20.2 (18.7-21.8) | -9.8 (-12.8 to -7.9) | -0.57 (-0.74 to -0.42) | -22.5 (-26.2 to -20.8) | -3.51 (-4.07 to -3.10) |
| Parbhani | 24.2 (19.8-28.4) | 18.1 (14.6-22.0) | 19.4 (18.2-20.6) | -20.0 (-23.0 to -18.5) | -1.27 (-1.45 to -1.12) | 7.1 (2.7 to 9.7) | 1.04 (0.52 to 1.46) |
| Pune | 23.4 (20.0-27.0) | 19.5 (15.6-24.2) | 18.1 (16.6-19.7) | -23.1 (-25.3 to -21.8) | -1.49 (-1.62 to -1.37) | -6.8 (-11.0 to -4.7) | -0.95 (-1.49 to -0.53) |
| Raigad | 20.6 (17.4-24.1) | 18.8 (14.7-23.5) | 17.4 (16.2-18.6) | -15.9 (-18.9 to -14.3) | -0.97 (-1.15 to -0.84) | -7.1 (-10.4 to -4.7) | -1.00 (-1.40 to -0.54) |
| Ratnagiri | 20.3 (14.7-25.7) | 18.6 (13.9-23.8) | 17.7 (16.1-19.3) | -12.3 (-16.6 to -10.3) | -0.76 (-0.99 to -0.57) | -4.2 (-8.9 to -1.3) | -0.60 (-1.17 to -0.04) |
| Sangli | 19.6 (16.2-23.6) | 28.9 (24.5-33.8) | 17.3 (16.2-18.5) | -12.1 (-15.1 to -10.6) | -0.72 (-0.89 to -0.59) | -40.7 (-42.2 to -39.7) | -7.01 (-7.32 to -6.75) |
| Satara | 22.1 (18.4-26.4) | 19.9 (15.7-24.9) | 17.3 (16.0-18.9) | -22.2 (-25.1 to -20.6) | -1.43 (-1.61 to -1.27) | -13.0 (-16.8 to -10.7) | -1.92 (-2.43 to -1.45) |
| Sindhudurg | 14.7 (10.6-19.6) | 20.3 (15.0-26.6) | 14.2 (12.9-15.9) | -2.4 (-6.9 to 0.7) | -0.16 (-0.36 to 0.10) | -29.8 (-33.4 to -28.0) | -4.88 (-5.44 to -4.39) |
| Solapur | 22.9 (19.6-27.1) | 24.6 (20.6-29.2) | 15.8 (14.8-17.0) | -31.5 (-33.9 to -29.8) | -2.15 (-2.32 to -1.98) | -36.4 (-38.3 to -35.1) | -6.11 (-6.46 to -5.78) |
| Thane | 20.8 (16.9-24.9) | 21.6 (16.7-26.8) | 18.0 (16.5-19.8) | -13.3 (-16.5 to -11.2) | -0.81 (-0.99 to -0.63) | -15.9 (-20.2 to -14.5) | -2.41 (-3.00 to -2.04) |
| Wardha | 21.2 (17.4-25.3) | 25.1 (20.7-30.1) | 20.3 (19.1-21.6) | -4.3 (-7.5 to -2.6) | -0.23 (-0.40 to -0.09) | -19.3 (-22.3 to -17.5) | -2.92 (-3.36 to -2.54) |
| Washim | 25.0 (20.5-30.5) | 21.8 (16.9-27.3) | 20.5 (19.0-21.8) | -18.3 (-21.0 to -16.7) | -1.15 (-1.31 to -1.00) | -5.7 (-9.5 to -3.7) | -0.79 (-1.26 to -0.39) |
| Yavatmal | 23.2 (19.1-27.5) | 24.5 (19.7-30.2) | 22.7 (21.5-23.9) | -2.6 (-5.5 to -0.6) | -0.12 (-0.27 to 0.02) | -7.4 (-10.9 to -5.0) | -1.02 (-1.48 to -0.58) |
| **Punjab** | **10.7 (10.0-11.4)** | **12.1 (11.5-12.6)** | **10.6 (10.1-11.1)** | **-1.6 (-4.0 to -0.2)** | **-0.05 (-0.18 to 0.05)** | **-12.9 (-15.1 to -11.6)** | **-1.90 (-3.00 to -0.80)** |
| Amritsar | 11.9 (8.4-16.1) | 13.2 (11.0-16.1) | 9.5 (8.7-10.5) | -18.8 (-23.8 to -17.5) | -1.24 (-1.51 to -1.05) | -28.4 (-30.8 to -26.3) | -4.55 (-4.94 to -4.07) |
| Barnala | 7.7 (5.7-10.1) | 10.9 (8.1-14.2) | 9.6 (9.3-10.0) | 27.0 (19.8 to 30.7) | 1.39 (1.12 to 1.63) | -10.8 (-14.9 to -8.9) | -1.60 (-2.12 to -1.17) |
| Bathinda | 8.9 (6.7-11.5) | 13.6 (10.7-16.9) | 10.7 (10.3-11.2) | 21.5 (15.6 to 25.0) | 1.13 (0.91 to 1.37) | -21.3 (-24.6 to -19.5) | -3.29 (-3.77 to -2.89) |
| Faridkot | 9.2 (6.4-12.6) | 15.2 (12.2-18.3) | 10.8 (10.0-11.6) | 19.1 (11.8 to 21.2) | 0.99 (0.71 to 1.19) | -29.1 (-31.6 to -27.7) | -4.68 (-5.08 to -4.33) |
| Fatehgarh Sahib | 11.2 (8.7-14.2) | 9.9 (7.5-12.4) | 11.2 (10.8-11.8) | 0.9 (-3.8 to 3.4) | 0.06 (-0.16 to 0.25) | 14.0 (8.1 to 16.1) | 1.90 (1.25 to 2.28) |
| Fazilka | 12.4 (8.5-17.4) | 17.6 (14.1-21.5) | 11.9 (11.1-12.8) | -1.5 (-6.8 to 1.4) | -0.14 (-0.35 to 0.14) | -32.7 (-35.6 to -31.1) | -5.37 (-5.89 to -4.99) |
| Ferozepur | 9.7 (6.6-13.5) | 15.6 (12.8-18.9) | 10.8 (10.1-11.7) | 13.5 (6.1 to 16.0) | 0.69 (0.40 to 0.93) | -31.3 (-33.9 to -29.9) | -5.12 (-5.53 to -4.76) |
| Gurdaspur | 13.8 (10.6-17.4) | 12.4 (10.4-15.0) | 9.9 (9.4-10.6) | -28.1 (-31.3 to -26.2) | -1.89 (-2.10 to -1.69) | -20.1 (-22.4 to -18.3) | -3.05 (-3.37 to -2.68) |
| Hoshiarpur | 13.2 (10.4-16.3) | 11.2 (8.5-14.2) | 10.9 (10.3-11.5) | -17.5 (-20.9 to -15.7) | -1.10 (-1.29 to -0.93) | -2.7 (-7.2 to 0.4) | -0.36 (-0.91 to 0.20) |
| Jalandhar | 10.1 (7.7-12.9) | 11.3 (8.7-14.2) | 10.7 (10.1-11.4) | 6.8 (1.6 to 9.6) | 0.38 (0.15 to 0.60) | -4.9 (-9.3 to -2.9) | -0.68 (-1.23 to -0.27) |
| Kapurthala | 10.6 (8.1-13.5) | 12.0 (9.5-14.9) | 10.8 (10.2-11.3) | 3.0 (-2.1 to 5.1) | 0.18 (-0.07 to 0.35) | -10.1 (-14.2 to -7.8) | -1.46 (-2.00 to -1.00) |
| Ludhiana | 9.5 (7.3-12.0) | 10.0 (7.6-12.8) | 10.1 (9.6-10.6) | 7.5 (2.2 to 9.8) | 0.43 (0.18 to 0.61) | 1.9 (-3.3 to 3.9) | 0.28 (-0.34 to 0.69) |
| Mansa | 8.3 (5.6-11.3) | 12.4 (8.8-16.3) | 10.7 (10.2-11.2) | 33.2 (23.6 to 37.7) | 1.64 (1.30 to 1.94) | -12.7 (-18.6 to -10.3) | -1.94 (-2.73 to -1.39) |
| Moga | 8.2 (6.0-11.0) | 12.5 (10.0-15.8) | 10.3 (9.8-10.8) | 27.1 (19.6 to 31.1) | 1.39 (1.11 to 1.65) | -17.7 (-20.8 to -15.5) | -2.67 (-3.10 to -2.22) |
| Pathankot | 15.8 (12.3-19.5) | 11.0 (8.5-13.5) | 8.5 (7.9-9.2) | -46.5 (-49.1 to -45.4) | -3.55 (-3.79 to -3.40) | -22.5 (-26.2 to -21.2) | -3.49 (-4.06 to -3.17) |
| Patiala | 10.4 (8.2-13.0) | 10.2 (7.9-13.0) | 11.9 (11.5-12.4) | 15.4 (11.1 to 18.8) | 0.86 (0.67 to 1.07) | 17.2 (11.6 to 18.7) | 2.30 (1.71 to 2.60) |
| Rupnagar | 12.0 (9.1-15.2) | 9.3 (7.1-11.6) | 10.5 (10.1-11.0) | -11.9 (-15.6 to -9.0) | -0.72 (-0.93 to -0.49) | 13.5 (8.5 to 16.0) | 1.83 (1.31 to 2.26) |
| Sahibzada Ajit Singh Nagar | 12.3 (8.9-16.5) | 9.2 (6.4-12.1) | 11.3 (10.6-11.9) | -7.2 (-11.5 to -4.9) | -0.45 (-0.65 to -0.23) | 24.1 (16.6 to 26.8) | 3.07 (2.34 to 3.56) |
| Sangrur | 9.0 (6.8-11.7) | 11.1 (8.0-14.1) | 10.9 (10.6-11.3) | 22.1 (15.6 to 25.7) | 1.17 (0.91 to 1.40) | -1.3 (-6.9 to 0.9) | -0.16 (-0.87 to 0.26) |
| Shahid Bhagat Singh Nagar | 12.2 (9.2-15.5) | 10.6 (7.8-13.5) | 11.1 (10.4-11.8) | -8.2 (-12.9 to -5.2) | -0.50 (-0.74 to -0.25) | 5.9 (-0.3 to 7.8) | 0.81 (0.10 to 1.21) |
| Sri Mukhtar Sahib | 10.7 (7.4-14.4) | 16.1 (12.9-19.4) | 11.5 (10.8-12.1) | 9.1 (3.9 to 12.5) | 0.48 (0.28 to 0.75) | -28.8 (-31.5 to -27.1) | -4.64 (-5.07 to -4.22) |
| Tarn Taran | 10.0 (7.3-13.3) | 13.7 (11.4-16.2) | 9.7 (9.2-10.3) | -1.6 (-6.7 to 0.7) | -0.11 (-0.34 to 0.10) | -29.6 (-32.3 to -28.0) | -4.76 (-5.22 to -4.40) |
| **Sikkim** | **7.7 (6.5-9.2)** | **7.5 (6.9-8.1)** | **6.3 (5.9-6.8)** | **-17.1 (-20.2 to -14.9)** | **-1.08 (-1.24 to -0.88)** | **-14.2 (-19.1 to -12.3)** | **-2.35 (-3.88 to -0.82)** |
| East Sikkim | 8.9 (6.7-11.5) | 7.8 (5.6-10.3) | 6.9 (6.4-7.4) | -22.1 (-26.1 to -20.3) | -1.45 (-1.68 to -1.26) | -10.7 (-16.1 to -7.4) | -1.64 (-2.31 to -0.94) |
| North Sikkim | 6.6 (4.7-9.2) | 6.5 (4.4-9.1) | 5.6 (5.1-6.3) | -13.7 (-18.6 to -10.3) | -0.90 (-1.13 to -0.57) | -11.7 (-18.9 to -9.7) | -1.88 (-2.78 to -1.29) |
| South Sikkim | 7.9 (6.2-9.8) | 8.0 (6.0-10.3) | 6.7 (6.3-7.2) | -15.3 (-18.7 to -12.5) | -0.96 (-1.14 to -0.71) | -16.3 (-21.1 to -14.6) | -2.50 (-3.15 to -2.07) |
| West Sikkim | 6.1 (4.7-7.8) | 7.0 (5.0-9.4) | 5.6 (5.3-6.1) | -7.5 (-11.9 to -3.9) | -0.46 (-0.68 to -0.17) | -18.5 (-22.9 to -16.6) | -2.91 (-3.46 to -2.39) |
| **Nagaland** | **12.5 (11.3-13.9)** | **12.8 (12.2-13.4)** | **11.6 (11.1-12.1)** | **-7.1 (-11.1 to -5.0)** | **-0.41 (-0.62 to -0.24)** | **-6.7 (-13.9 to -4.4)** | **-1.34 (-2.48 to -0.30)** |
| Dimapur | 13.4 (10.0-17.7) | 16.7 (10.5-23.4) | 10.9 (9.9-11.9) | -18.3 (-23.2 to -16.4) | -1.18 (-1.47 to -0.98) | -32.9 (-38.5 to -31.9) | -5.65 (-6.49 to -5.13) |
| Kiphire | 12.6 (8.9-16.8) | 10.1 (6.2-15.0) | 11.2 (10.1-12.6) | -9.1 (-14.3 to -6.8) | -0.59 (-0.84 to -0.35) | 15.9 (5.6 to 18.3) | 1.92 (0.92 to 2.55) |
| Kohima | 12.6 (9.8-16.2) | 12.1 (7.7-17.5) | 8.9 (8.4-9.5) | -29.0 (-32.4 to -26.9) | -1.97 (-2.19 to -1.74) | -23.7 (-30.3 to -22.4) | -3.90 (-4.83 to -3.38) |
| Longleng | 11.4 (8.2-15.6) | 11.0 (6.8-16.6) | 13.5 (12.4-14.8) | 20.9 (14.6 to 23.7) | 1.08 (0.86 to 1.31) | 28.4 (15.3 to 32.3) | 3.40 (2.18 to 4.20) |
| Mokokchung | 11.1 (7.9-14.8) | 11.1 (7.1-17.0) | 11.5 (10.9-12.3) | 5.7 (0.6 to 8.4) | 0.31 (0.10 to 0.53) | 7.7 (-2.3 to 11.3) | 0.89 (-0.18 to 1.67) |
| Mon | 11.9 (8.3-16.6) | 11.3 (7.2-16.9) | 15.7 (14.6-17.0) | 35.2 (28.8 to 39.1) | 1.75 (1.55 to 2.00) | 44.5 (33.1 to 48.6) | 5.17 (4.28 to 5.92) |
| Peren | 13.9 (10.6-18.5) | 16.2 (10.7-23.1) | 9.4 (8.7-10.0) | -32.1 (-35.0 to -29.5) | -2.24 (-2.42 to -1.95) | -41.1 (-46.5 to -39.4) | -7.30 (-8.31 to -6.68) |
| Phek | 12.0 (9.1-15.7) | 10.6 (6.6-15.1) | 9.6 (9.1-10.3) | -19.3 (-23.5 to -17.2) | -1.24 (-1.48 to -1.03) | -5.7 (-12.9 to -3.5) | -0.97 (-1.79 to -0.36) |
| Tuensang | 13.4 (9.4-18.2) | 11.2 (6.8-16.7) | 14.6 (13.4-15.8) | 11.2 (4.9 to 14.9) | 0.60 (0.34 to 0.87) | 35.9 (24.3 to 38.4) | 4.24 (3.28 to 4.86) |
| Wokha | 12.1 (8.8-15.8) | 13.0 (8.3-19.0) | 12.3 (11.4-13.2) | 3.0 (-2.6 to 5.5) | 0.16 (-0.10 to 0.37) | -2.0 (-9.9 to 1.6) | -0.45 (-1.32 to 0.37) |
| Zunheboto | 12.2 (8.5-16.4) | 10.3 (6.1-15.8) | 10.2 (9.3-11.2) | -15.2 (-20.7 to -12.9) | -0.99 (-1.28 to -0.75) | 2.9 (-6.9 to 5.4) | 0.18 (-0.86 to 0.90) |
| **Himachal Pradesh** | **11.3 (10.6-12.1)** | **12.1 (11.4-12.9)** | **10.6 (9.9-11.2)** | **-6.8 (-8.6 to -5.0)** | **-0.37 (-0.47 to -0.24)** | **-12.9 (-15.7 to -11.0)** | **-1.95 (-3.32 to -0.56)** |
| Bilaspur | 11.3 (9.0-13.9) | 11.9 (9.3-14.7) | 11.5 (10.8-12.3) | 2.4 (-0.9 to 4.3) | 0.16 (0.01 to 0.30) | -3.0 (-6.6 to -1.0) | -0.39 (-0.82 to 0.01) |
| Chamba | 11.8 (8.9-14.7) | 12.5 (9.1-15.9) | 9.6 (9.0-10.4) | -17.9 (-22.4 to -16.2) | -1.14 (-1.41 to -0.97) | -22.1 (-26.7 to -20.0) | -3.48 (-4.15 to -2.97) |
| Hamirpur | 11.7 (9.1-14.7) | 12.4 (9.5-15.7) | 10.6 (9.7-11.6) | -9.1 (-12.7 to -6.8) | -0.54 (-0.73 to -0.35) | -13.9 (-17.9 to -11.8) | -2.08 (-2.60 to -1.62) |
| Kangra | 11.9 (9.6-14.5) | 13.0 (9.9-16.5) | 10.0 (9.2-10.8) | -16.0 (-20.0 to -14.5) | -1.00 (-1.23 to -0.85) | -22.5 (-26.5 to -20.3) | -3.51 (-4.12 to -3.01) |
| Kinnaur | 11.5 (7.6-16.9) | 11.9 (7.7-17.3) | 9.4 (8.3-10.5) | -15.6 (-22.9 to -13.9) | -1.06 (-1.44 to -0.81) | -18.8 (-25.8 to -16.7) | -3.05 (-3.99 to -2.41) |
| Kullu | 11.4 (8.1-15.5) | 12.5 (9.2-16.4) | 9.3 (8.1-10.7) | -17.5 (-23.1 to -15.9) | -1.15 (-1.46 to -0.94) | -25.5 (-29.9 to -23.2) | -4.10 (-4.75 to -3.53) |
| Lahul and Spiti | 10.9 (7.9-14.6) | 11.6 (8.0-15.6) | 8.7 (7.5-9.9) | -19.2 (-24.7 to -16.3) | -1.27 (-1.58 to -0.97) | -23.8 (-28.5 to -21.7) | -3.82 (-4.49 to -3.26) |
| Mandi | 11.7 (9.2-14.2) | 11.7 (9.0-14.8) | 10.0 (9.5-10.7) | -14.0 (-17.5 to -12.2) | -0.86 (-1.05 to -0.69) | -14.3 (-17.5 to -11.8) | -2.12 (-2.54 to -1.62) |
| Shimla | 10.9 (8.1-14.1) | 11.1 (8.0-14.6) | 10.7 (10.0-11.6) | -0.1 (-5.3 to 2.6) | -0.02 (-0.26 to 0.21) | -2.6 (-8.3 to 0.1) | -0.39 (-1.07 to 0.15) |
| Sirmaur | 9.9 (7.8-12.5) | 11.3 (8.8-14.5) | 12.0 (11.3-12.7) | 22.2 (16.4 to 24.9) | 1.18 (0.95 to 1.37) | 6.2 (2.3 to 8.7) | 0.89 (0.46 to 1.33) |
| Solan | 10.2 (7.9-13.0) | 10.7 (7.9-13.6) | 11.7 (11.1-12.3) | 16.2 (12.0 to 19.6) | 0.89 (0.72 to 1.11) | 10.5 (5.5 to 13.1) | 1.44 (0.90 to 1.90) |
| Una | 11.5 (9.4-14.1) | 13.7 (10.5-17.3) | 12.4 (11.7-13.1) | 7.6 (3.4 to 10.1) | 0.45 (0.25 to 0.62) | -9.4 (-13.3 to -7.2) | -1.35 (-1.86 to -0.92) |
| **UTs other than Delhi** | **16.3 (14.3-18.7)** | **14.1 (12.3-15.9)** | **13.5 (11.8-15.5)** | **-17.4 (-20.2 to -15.6)** | **-1.08 (-1.25 to -0.92)** | **-3.8 (-7.1 to -1.7)** | **-0.53 (-1.95 to 0.77)** |
| Chandigarh | 11.2 (7.8-15.7) | 8.1 (5.5-11.2) | 13.2 (12.1-14.8) | 21.2 (12.3 to 26.6) | 1.06 (0.74 to 1.44) | 68.9 (56.7 to 73.4) | 7.57 (6.73 to 8.27) |
| Dadra & Nagar Haveli | 26.0 (20.3-32.2) | 18.6 (13.1-24.4) | 17.5 (15.9-18.8) | -33.0 (-36.2 to -31.5) | -2.30 (-2.52 to -2.12) | -4.8 (-11.7 to -2.6) | -0.76 (-1.60 to -0.22) |
| Daman | 26.1 (17.7-36.0) | 19.9 (11.9-30.0) | 17.2 (16.0-19.6) | -32.7 (-39.8 to -30.1) | -2.38 (-2.84 to -2.01) | -9.5 (-20.4 to -6.9) | -1.79 (-3.03 to -0.86) |
| Diu | 21.1 (11.6-32.6) | 10.2 (5.3-17.2) | 12.5 (10.9-14.6) | -37.9 (-46.8 to -36.5) | -3.01 (-3.54 to -2.55) | 31.8 (8.4 to 32.7) | 3.20 (1.30 to 4.23) |
| Karaikal | 18.0 (11.9-25.7) | 13.7 (9.3-19.4) | 13.3 (11.7-15.3) | -24.5 (-31.5 to -22.6) | -1.69 (-2.12 to -1.42) | -0.2 (-8.0 to 3.3) | -0.21 (-1.03 to 0.60) |
| Lakshwadeep | 11.3 (4.6-22.9) | 10.9 (4.9-20.1) | 10.7 (9.9-13.2) | 9.1 (-10.6 to 10.5) | -0.04 (-0.67 to 0.73) | 8.7 (-11.6 to 6.8) | 0.48 (-2.21 to 1.52) |
| Mahe | 10.0 (6.4-14.9) | 10.2 (7.0-15.1) | 9.7 (8.7-11.4) | 0.1 (-10.6 to 5.4) | -0.14 (-0.59 to 0.37) | -1.9 (-11.5 to 1.9) | -0.54 (-1.58 to 0.40) |
| Nicobars | 15.3 (6.8-27.8) | 11.8 (5.9-20.1) | 6.7 (5.3-8.6) | -50.5 (-59.8 to -50.5) | -4.43 (-5.09 to -3.94) | -39.2 (-47.5 to -38.5) | -7.34 (-8.55 to -6.49) |
| North & Middle Andaman | 18.1 (7.7-32.8) | 21.6 (12.3-33.3) | 16.2 (15.3-18.0) | 3.9 (-17.8 to 5.7) | -0.21 (-1.08 to 0.38) | -20.6 (-28.2 to -18.2) | -3.54 (-4.44 to -2.67) |
| Puducherry | 16.1 (11.1-22.0) | 16.9 (11.7-23.3) | 12.3 (11.4-14.1) | -22.2 (-28.5 to -20.1) | -1.54 (-1.87 to -1.24) | -26.2 (-32.3 to -23.8) | -4.34 (-5.22 to -3.63) |
| South Andaman | 15.3 (5.9-29.8) | 14.8 (7.4-25.8) | 10.1 (8.5-12.0) | -22.2 (-38.0 to -23.6) | -1.94 (-2.68 to -1.49) | -26.0 (-37.0 to -24.5) | -4.70 (-6.18 to -3.76) |
| Yanam | 19.3 (11.4-30.1) | 15.3 (7.7-26.7) | 15.9 (14.9-18.6) | -13.2 (-24.1 to -11.0) | -1.07 (-1.53 to -0.62) | 14.2 (-2.5 to 17.7) | 1.11 (-0.22 to 2.48) |
| **Kerala** | **12.5 (11.7-13.4)** | **15.7 (15.0-16.3)** | **15.4 (14.8-16.0)** | **22.6 (19.4 to 24.5)** | **1.23 (1.10 to 1.35)** | **-2.5 (-4.2 to -1.1)** | **-0.27 (-1.22 to 0.66)** |
| Alappuzha | 10.9 (8.1-14.5) | 18.2 (15.0-22.1) | 17.4 (16.1-18.7) | 61.5 (52.5 to 66.9) | 2.82 (2.55 to 3.10) | -4.8 (-8.7 to -2.5) | -0.64 (-1.14 to -0.21) |
| Ernakulam | 11.2 (8.1-14.7) | 16.4 (13.4-20.2) | 15.0 (13.5-16.6) | 36.6 (29.1 to 39.0) | 1.82 (1.56 to 2.00) | -8.1 (-12.2 to -5.6) | -1.14 (-1.68 to -0.67) |
| Idukki | 15.4 (12.6-18.4) | 19.9 (17.0-23.2) | 16.4 (15.4-17.5) | 6.5 (3.2 to 9.1) | 0.39 (0.24 to 0.57) | -17.8 (-20.1 to -15.9) | -2.64 (-2.99 to -2.27) |
| Kannur | 12.2 (9.3-15.6) | 13.5 (10.6-16.5) | 13.5 (12.6-14.6) | 11.5 (7.1 to 14.4) | 0.64 (0.46 to 0.85) | 0.6 (-3.6 to 1.8) | 0.13 (-0.37 to 0.40) |
| Kasaragod | 11.9 (8.5-15.9) | 14.1 (10.4-18.6) | 16.5 (14.9-18.1) | 41.8 (33.1 to 44.0) | 2.03 (1.74 to 2.21) | 18.1 (12.1 to 21.6) | 2.38 (1.77 to 2.95) |
| Kollam | 12.5 (8.8-16.6) | 18.7 (15.0-23.4) | 17.7 (16.5-19.7) | 44.6 (36.2 to 48.6) | 2.13 (1.88 to 2.40) | -5.0 (-9.6 to -2.3) | -0.71 (-1.27 to -0.19) |
| Kottayam | 11.7 (9.0-15.1) | 18.4 (15.2-22.0) | 16.5 (15.1-18.0) | 42.4 (36.7 to 46.1) | 2.08 (1.90 to 2.29) | -10.6 (-13.9 to -8.5) | -1.50 (-1.95 to -1.10) |
| Kozhikode | 12.2 (9.1-16.0) | 12.5 (10.1-15.5) | 12.7 (11.3-14.3) | 5.4 (0.5 to 8.9) | 0.29 (0.09 to 0.56) | 1.4 (-3.5 to 3.3) | 0.21 (-0.36 to 0.60) |
| Malappuram | 13.1 (10.3-15.7) | 13.8 (11.1-16.8) | 15.6 (14.3-17.0) | 19.7 (14.4 to 21.8) | 1.07 (0.84 to 1.21) | 13.4 (9.4 to 15.5) | 1.84 (1.42 to 2.21) |
| Palakkad | 15.4 (12.3-18.8) | 16.6 (13.4-20.4) | 15.7 (14.1-17.3) | 2.6 (-1.7 to 4.8) | 0.16 (-0.04 to 0.33) | -5.5 (-8.9 to -3.4) | -0.75 (-1.17 to -0.35) |
| Pathanamthitta | 13.4 (10.1-17.4) | 20.8 (17.2-25.2) | 18.6 (17.0-20.4) | 39.8 (33.2 to 43.9) | 1.97 (1.75 to 2.21) | -11.1 (-14.3 to -9.1) | -1.59 (-2.02 to -1.20) |
| Thiruvananthapuram | 12.1 (8.4-16.9) | 14.9 (11.3-18.8) | 14.8 (13.6-16.8) | 25.0 (16.2 to 28.1) | 1.24 (0.94 to 1.51) | -0.1 (-6.6 to 3.0) | -0.07 (-0.82 to 0.57) |
| Thrissur | 11.6 (8.8-14.9) | 14.4 (11.6-17.7) | 14.6 (13.1-16.2) | 26.5 (19.6 to 30.1) | 1.37 (1.11 to 1.61) | 1.4 (-2.6 to 4.0) | 0.24 (-0.23 to 0.70) |
| Wayanad | 13.9 (10.9-17.3) | 14.5 (11.6-17.7) | 14.1 (13.0-15.3) | 1.6 (-2.4 to 3.8) | 0.10 (-0.08 to 0.28) | -2.6 (-7.0 to -1.1) | -0.33 (-0.88 to -0.01) |
| **Delhi** | **18.8 (17.7-19.9)** | **15.8 (15.0-16.7)** | **13.8 (13.0-14.5)** | **-26.6 (-29.6 to -25.1)** | **-1.77 (-1.96 to -1.61)** | **-12.8 (-16.8 to -10.9)** | **-1.99 (-3.31 to -0.77)** |
| Central | 19.1 (12.8-26.8) | 15.5 (10.0-22.1) | 13.7 (12.3-15.9) | -26.8 (-33.7 to -24.3) | -1.91 (-2.30 to -1.55) | -8.6 (-17.1 to -5.6) | -1.53 (-2.47 to -0.66) |
| East | 20.2 (13.8-28.7) | 15.0 (10.3-20.9) | 13.4 (12.3-15.3) | -31.9 (-37.2 to -30.0) | -2.30 (-2.61 to -1.99) | -8.3 (-15.4 to -5.2) | -1.39 (-2.19 to -0.62) |
| New Delhi | 18.6 (12.6-26.0) | 14.8 (9.6-21.1) | 13.2 (11.9-15.7) | -27.1 (-33.2 to -25.2) | -1.93 (-2.26 to -1.62) | -8.0 (-17.3 to -5.4) | -1.41 (-2.50 to -0.64) |
| North | 18.8 (13.5-25.4) | 15.4 (10.9-21.0) | 13.6 (12.0-15.7) | -26.8 (-32.0 to -25.3) | -1.85 (-2.16 to -1.62) | -10.4 (-15.4 to -7.2) | -1.64 (-2.20 to -0.90) |
| North East | 18.7 (12.2-27.0) | 15.2 (10.2-21.7) | 13.4 (12.1-15.6) | -26.2 (-34.0 to -25.1) | -1.88 (-2.32 to -1.61) | -9.3 (-18.6 to -5.0) | -1.60 (-2.73 to -0.58) |
| North West | 18.3 (13.8-24.0) | 16.5 (12.5-21.2) | 13.9 (12.4-15.7) | -23.3 (-27.7 to -21.2) | -1.55 (-1.81 to -1.32) | -15.0 (-19.7 to -12.1) | -2.29 (-2.91 to -1.67) |
| Shahdara | 20.2 (10.9-31.8) | 14.9 (8.6-25.5) | 13.4 (12.3-15.8) | -29.1 (-38.7 to -26.9) | -2.28 (-2.75 to -1.75) | -4.3 (-18.8 to -0.2) | -1.22 (-2.77 to 0.12) |
| South | 20.2 (14.8-27.2) | 15.9 (11.5-20.9) | 14.1 (12.7-15.6) | -29.4 (-33.4 to -27.2) | -2.03 (-2.28 to -1.77) | -10.3 (-15.7 to -7.6) | -1.59 (-2.25 to -0.97) |
| South East | 20.4 (14.5-27.3) | 15.5 (10.7-21.2) | 13.8 (12.8-15.5) | -31.3 (-36.5 to -29.5) | -2.22 (-2.55 to -1.96) | -8.8 (-15.9 to -6.6) | -1.45 (-2.28 to -0.81) |
| South West | 18.2 (13.6-24.4) | 16.5 (12.3-21.8) | 14.1 (12.5-15.7) | -21.9 (-26.1 to -19.4) | -1.44 (-1.69 to -1.19) | -13.5 (-18.2 to -11.3) | -2.05 (-2.66 to -1.55) |
| West | 17.1 (12.2-22.7) | 16.1 (11.8-21.5) | 13.7 (12.2-15.8) | -18.6 (-24.2 to -15.7) | -1.23 (-1.54 to -0.93) | -13.5 (-19.3 to -11.3) | -2.10 (-2.84 to -1.54) |
| **Goa** | **13.7 (12.6-14.8)** | **14.9 (14.1-15.6)** | **15.2 (14.5-15.9)** | **11.9 (6.2 to 14.6)** | **0.65 (0.41 to 0.86)** | **3.3 (-2.5 to 7.1)** | **0.32 (-0.73 to 1.36)** |
| North Goa | 14.3 (10.4-18.8) | 14.5 (10.9-18.6) | 15.2 (13.7-16.7) | 7.5 (1.4 to 10.9) | 0.41 (0.14 to 0.66) | 5.5 (-0.7 to 8.9) | 0.74 (0.05 to 1.36) |
| South Goa | 13.2 (9.8-16.7) | 15.1 (11.6-19.1) | 15.2 (13.6-16.8) | 16.3 (10.8 to 18.4) | 0.88 (0.66 to 1.05) | 1.6 (-4.0 to 5.5) | 0.22 (-0.43 to 0.90) |

UTs=Union territories.

^*^The states are listed in increasing order of Socio-demographic Index in 2017.

^†^The state of Jammu and Kashmir was divided into two union territories in August 2019; as we are reporting findings up to 2017, we report findings for the undivided state of Jammu and Kashmir.

# **6. Prevalence of underweight in the districts of India in 2000, 2010 and 2017**

| **States*/Districts** | **Underweight percent prevalence (95% uncertainty interval)** | | | | | | |
| --- | --- | --- | --- | --- | --- | --- | --- |
|  | **2000** | **2010** | **2017** | **Percentage change, 2000-2017** | **Annual rate of change, 2000-2017 (%)** | **Percentage change, 2010-2017** | **Annual rate of change 2010-2017 (%)** |
| **India** | **53.4 (52.3-54.6)** | **40.9 (40.3-41.5)** | **32.7 (32.3-33.1)** | **-39.9 (-40.0 to -39.8)** | **-2.85 (-2.86 to -2.84)** | **-21.1 (-21.4 to -20.9)** | **-3.22 (-3.44 to -2.98)** |
| **Bihar** | **60.5 (55.4-65.1)** | **49.7 (46.9-53.0)** | **39.1 (37.7-41.0)** | **-36.3 (-36.6 to -36.0)** | **-2.53 (-2.56 to -2.50)** | **-22.2 (-22.9 to -21.6)** | **-3.42 (-4.36 to -2.54)** |
| Araria | 58.0 (52.7-63.4) | 47.5 (41.4-54.0) | 39.4 (38.7-40.2) | -32.8 (-33.8 to -32.1) | -2.24 (-2.31 to -2.17) | -17.6 (-19.5 to -16.3) | -2.59 (-2.88 to -2.34) |
| Arwal | 65.9 (60.3-71.8) | 51.0 (42.7-58.6) | 45.0 (43.6-46.3) | -32.6 (-33.7 to -31.8) | -2.22 (-2.30 to -2.15) | -12.3 (-14.9 to -11.2) | -1.74 (-2.12 to -1.52) |
| Aurangabad | 64.8 (58.6-70.7) | 51.2 (44.2-58.3) | 45.7 (45.1-46.6) | -30.3 (-31.5 to -29.6) | -2.03 (-2.12 to -1.96) | -11.3 (-13.5 to -10.0) | -1.57 (-1.89 to -1.34) |
| Banka | 60.4 (52.8-68.1) | 58.3 (50.7-65.8) | 43.9 (43.0-44.9) | -28.0 (-29.6 to -27.0) | -1.85 (-1.96 to -1.76) | -25.3 (-27.1 to -24.5) | -3.93 (-4.22 to -3.76) |
| Begusarai | 55.5 (50.1-60.6) | 51.3 (43.6-59.4) | 38.7 (38.0-39.4) | -31.1 (-32.3 to -30.2) | -2.09 (-2.18 to -2.01) | -25.1 (-27.0 to -23.7) | -3.91 (-4.21 to -3.61) |
| Bhagalpur | 58.3 (50.9-66.8) | 51.9 (44.4-59.1) | 41.0 (40.2-41.8) | -30.3 (-31.8 to -29.4) | -2.03 (-2.15 to -1.95) | -21.5 (-23.7 to -20.6) | -3.26 (-3.62 to -3.06) |
| Bhojpur | 61.2 (53.8-67.9) | 47.8 (39.6-57.0) | 40.4 (39.3-41.3) | -34.7 (-36.1 to -34.1) | -2.40 (-2.51 to -2.33) | -15.7 (-18.4 to -14.5) | -2.30 (-2.69 to -2.05) |
| Buxar | 57.8 (51.9-63.3) | 44.1 (37.7-50.6) | 36.8 (36.0-37.6) | -37.1 (-38.4 to -36.4) | -2.61 (-2.71 to -2.54) | -17.1 (-19.2 to -15.8) | -2.52 (-2.84 to -2.26) |
| Darbhanga | 65.3 (60.7-69.3) | 51.7 (45.2-58.1) | 36.7 (35.8-37.7) | -44.7 (-45.4 to -44.0) | -3.33 (-3.40 to -3.25) | -29.7 (-31.1 to -28.9) | -4.75 (-4.98 to -4.57) |
| East Champaran | 61.4 (57.2-66.0) | 49.4 (43.5-55.7) | 35.9 (35.2-36.8) | -42.4 (-43.1 to -41.9) | -3.10 (-3.17 to -3.04) | -28.0 (-29.6 to -27.0) | -4.42 (-4.70 to -4.22) |
| Gaya | 69.4 (64.0-75.2) | 55.1 (46.8-62.9) | 47.7 (46.8-48.7) | -32.1 (-33.0 to -31.2) | -2.18 (-2.24 to -2.09) | -13.8 (-16.2 to -12.9) | -1.98 (-2.34 to -1.80) |
| Gopalganj | 58.2 (53.5-62.8) | 45.5 (39.1-51.2) | 32.1 (31.3-33.0) | -45.7 (-46.6 to -45.0) | -3.44 (-3.52 to -3.35) | -30.1 (-31.9 to -29.1) | -4.82 (-5.14 to -4.61) |
| Jamui | 58.0 (51.9-63.5) | 57.4 (49.7-64.4) | 42.8 (42.1-43.4) | -27.0 (-28.3 to -26.0) | -1.76 (-1.86 to -1.68) | -26.0 (-27.9 to -25.2) | -4.06 (-4.39 to -3.88) |
| Jehanabad | 67.0 (61.9-72.7) | 53.1 (45.4-61.2) | 45.2 (44.2-46.2) | -33.5 (-34.5 to -32.6) | -2.29 (-2.37 to -2.21) | -15.4 (-17.5 to -14.7) | -2.23 (-2.55 to -2.09) |
| Kaimur | 56.7 (48.6-64.5) | 42.4 (36.1-49.3) | 39.9 (38.9-40.9) | -30.3 (-31.8 to -29.1) | -2.04 (-2.14 to -1.92) | -6.2 (-9.3 to -4.7) | -0.81 (-1.23 to -0.54) |
| Katihar | 59.8 (54.4-65.5) | 44.7 (39.8-50.0) | 39.5 (38.7-40.1) | -34.8 (-35.9 to -34.0) | -2.40 (-2.50 to -2.33) | -12.3 (-13.9 to -11.0) | -1.72 (-1.95 to -1.50) |
| Khagaria | 56.7 (50.8-63.3) | 50.5 (42.3-58.5) | 39.5 (38.6-40.5) | -31.1 (-32.2 to -30.1) | -2.10 (-2.18 to -2.00) | -22.2 (-24.2 to -21.1) | -3.39 (-3.69 to -3.15) |
| Kishanganj | 53.2 (47.2-58.9) | 44.0 (37.0-51.5) | 39.4 (38.4-40.6) | -26.7 (-28.1 to -25.7) | -1.74 (-1.84 to -1.66) | -10.9 (-13.3 to -9.9) | -1.53 (-1.86 to -1.32) |
| Lakhisarai | 55.5 (48.3-62.5) | 55.0 (45.1-64.7) | 41.9 (40.5-43.1) | -25.2 (-26.8 to -24.2) | -1.63 (-1.74 to -1.54) | -24.2 (-27.2 to -23.3) | -3.75 (-4.24 to -3.54) |
| Madhepura | 59.8 (52.2-68.1) | 48.7 (40.6-57.3) | 39.1 (38.2-40.2) | -35.3 (-36.5 to -34.4) | -2.45 (-2.54 to -2.36) | -20.0 (-22.9 to -18.4) | -3.03 (-3.47 to -2.69) |
| Madhubani | 71.9 (67.1-76.0) | 53.0 (46.9-59.6) | 37.5 (36.6-38.5) | -48.7 (-49.3 to -48.2) | -3.75 (-3.80 to -3.69) | -29.9 (-31.3 to -29.0) | -4.78 (-5.03 to -4.58) |
| Munger | 55.9 (49.8-62.3) | 53.8 (45.7-62.0) | 41.4 (40.3-42.5) | -26.7 (-27.9 to -25.3) | -1.74 (-1.83 to -1.63) | -23.7 (-25.5 to -22.5) | -3.65 (-3.94 to -3.41) |
| Muzaffarpur | 56.7 (52.6-61.1) | 48.6 (43.0-54.5) | 34.5 (33.9-35.0) | -40.1 (-40.9 to -39.5) | -2.88 (-2.95 to -2.82) | -29.8 (-31.2 to -28.6) | -4.76 (-5.01 to -4.51) |
| Nalanda | 60.3 (56.5-64.6) | 51.9 (45.9-58.0) | 42.3 (41.7-42.9) | -30.8 (-31.8 to -30.2) | -2.07 (-2.14 to -2.01) | -19.3 (-21.0 to -18.1) | -2.86 (-3.14 to -2.64) |
| Nawada | 60.7 (55.5-65.3) | 53.8 (46.7-60.6) | 43.3 (42.5-43.9) | -29.6 (-30.6 to -28.9) | -1.96 (-2.05 to -1.91) | -20.2 (-22.6 to -19.3) | -3.04 (-3.41 to -2.85) |
| Patna | 57.4 (53.0-62.2) | 48.6 (42.4-54.9) | 39.0 (38.4-39.6) | -33.0 (-33.6 to -32.2) | -2.24 (-2.29 to -2.17) | -20.3 (-21.7 to -19.1) | -3.05 (-3.26 to -2.81) |
| Purnia | 57.8 (52.5-63.4) | 46.4 (40.3-53.0) | 40.0 (39.3-40.7) | -31.6 (-32.5 to -31.0) | -2.13 (-2.20 to -2.07) | -14.3 (-16.4 to -12.9) | -2.06 (-2.36 to -1.80) |
| Rohtas | 57.3 (48.9-64.9) | 44.2 (37.5-51.2) | 40.6 (39.6-41.6) | -29.7 (-31.5 to -29.1) | -1.99 (-2.12 to -1.92) | -8.5 (-10.8 to -7.1) | -1.15 (-1.46 to -0.89) |
| Saharsa | 60.2 (53.3-67.3) | 49.3 (41.4-57.1) | 38.2 (37.5-39.3) | -37.3 (-38.5 to -36.2) | -2.63 (-2.73 to -2.52) | -22.8 (-25.0 to -21.3) | -3.50 (-3.85 to -3.19) |
| Samastipur | 57.9 (54.0-61.5) | 50.9 (45.0-57.0) | 37.0 (36.5-37.5) | -37.0 (-37.8 to -36.3) | -2.59 (-2.67 to -2.53) | -27.9 (-29.3 to -26.9) | -4.41 (-4.65 to -4.20) |
| Saran | 57.1 (52.2-62.1) | 47.1 (40.4-54.7) | 36.1 (35.5-36.8) | -37.6 (-38.5 to -36.9) | -2.65 (-2.72 to -2.58) | -23.8 (-25.5 to -22.7) | -3.67 (-3.94 to -3.43) |
| Sheikhpura | 57.4 (50.9-63.4) | 54.4 (46.0-62.3) | 42.6 (41.5-44.1) | -26.5 (-28.1 to -25.7) | -1.73 (-1.84 to -1.65) | -22.2 (-24.7 to -21.4) | -3.39 (-3.79 to -3.20) |
| Sheohar | 64.7 (59.7-69.7) | 52.4 (46.1-58.5) | 37.2 (35.9-38.5) | -43.4 (-44.3 to -42.7) | -3.20 (-3.28 to -3.12) | -29.7 (-31.3 to -28.5) | -4.76 (-5.03 to -4.50) |
| Sitamarhi | 71.6 (67.3-75.4) | 55.2 (48.8-60.8) | 39.4 (38.1-40.4) | -46.0 (-46.6 to -45.4) | -3.46 (-3.51 to -3.40) | -29.4 (-30.9 to -28.6) | -4.68 (-4.95 to -4.51) |
| Siwan | 58.4 (54.3-63.5) | 45.8 (39.7-51.7) | 33.9 (33.2-34.7) | -42.9 (-43.5 to -42.2) | -3.15 (-3.21 to -3.08) | -26.7 (-28.5 to -25.8) | -4.19 (-4.50 to -3.98) |
| Supaul | 65.3 (60.4-70.1) | 50.6 (44.0-56.7) | 38.6 (37.6-39.6) | -41.7 (-42.4 to -41.1) | -3.04 (-3.09 to -2.97) | -24.3 (-26.0 to -23.3) | -3.75 (-4.03 to -3.53) |
| Vaishali | 53.5 (48.7-58.3) | 47.6 (40.8-55.1) | 35.4 (34.4-36.2) | -34.7 (-35.7 to -33.8) | -2.40 (-2.48 to -2.31) | -26.3 (-28.3 to -25.0) | -4.13 (-4.46 to -3.84) |
| West Champaran | 60.4 (55.0-65.2) | 46.5 (39.7-52.5) | 38.9 (37.5-40.1) | -36.5 (-37.7 to -35.9) | -2.56 (-2.65 to -2.49) | -17.1 (-19.0 to -16.5) | -2.52 (-2.80 to -2.38) |
| **Madhya Pradesh** | **64.7 (61.9-67.3)** | **51.8 (48.7-55.6)** | **37.1 (35.9-38.7)** | **-43.6 (-43.9 to -43.5)** | **-3.22 (-3.24 to -3.20)** | **-29.4 (-29.9 to -29.1)** | **-4.78 (-5.76 to -3.88)** |
| Agar Malwa | 62.4 (57.9-66.7) | 54.0 (48.2-59.8) | 34.7 (33.7-35.9) | -45.3 (-46.2 to -44.8) | -3.39 (-3.48 to -3.33) | -36.6 (-37.8 to -35.8) | -6.11 (-6.34 to -5.93) |
| Alirajpur | 73.5 (68.4-78.5) | 57.0 (51.2-62.5) | 47.9 (46.7-49.1) | -35.8 (-36.6 to -35.2) | -2.49 (-2.55 to -2.44) | -16.7 (-18.3 to -15.9) | -2.43 (-2.67 to -2.28) |
| Anuppur | 54.0 (47.5-61.2) | 46.8 (39.9-53.8) | 37.1 (35.8-38.3) | -32.1 (-33.6 to -31.1) | -2.18 (-2.29 to -2.08) | -21.3 (-23.2 to -20.0) | -3.23 (-3.52 to -2.96) |
| Ashoknagar | 73.9 (69.4-78.2) | 45.8 (39.0-53.2) | 38.0 (36.8-39.2) | -49.6 (-50.0 to -48.9) | -3.84 (-3.89 to -3.77) | -17.5 (-19.4 to -16.0) | -2.59 (-2.87 to -2.29) |
| Balaghat | 62.6 (58.1-67.6) | 48.3 (43.2-53.7) | 36.8 (35.9-37.7) | -42.1 (-42.9 to -41.6) | -3.08 (-3.15 to -3.01) | -24.6 (-26.0 to -23.7) | -3.80 (-4.02 to -3.61) |
| Barwani | 73.5 (68.2-78.4) | 55.5 (49.8-61.0) | 47.8 (47.0-48.6) | -35.8 (-36.4 to -35.4) | -2.49 (-2.54 to -2.45) | -14.5 (-15.8 to -13.1) | -2.07 (-2.25 to -1.82) |
| Betul | 57.4 (53.1-61.7) | 44.0 (37.8-50.5) | 33.0 (31.8-34.2) | -43.4 (-44.4 to -42.7) | -3.20 (-3.29 to -3.13) | -25.6 (-27.1 to -24.5) | -3.99 (-4.24 to -3.75) |
| Bhind | 61.1 (56.6-65.4) | 50.4 (43.3-57.4) | 40.9 (40.2-41.8) | -33.9 (-34.6 to -33.3) | -2.32 (-2.38 to -2.27) | -19.3 (-21.4 to -18.2) | -2.89 (-3.20 to -2.66) |
| Bhopal | 58.9 (53.0-66.2) | 54.4 (43.9-63.9) | 35.3 (34.1-36.7) | -41.0 (-41.9 to -39.8) | -2.97 (-3.05 to -2.85) | -35.5 (-37.9 to -34.6) | -5.93 (-6.37 to -5.68) |
| Burhanpur | 69.5 (64.2-74.5) | 46.8 (40.3-53.4) | 38.2 (37.1-39.5) | -46.0 (-46.9 to -45.2) | -3.46 (-3.55 to -3.38) | -18.9 (-21.0 to -17.3) | -2.82 (-3.13 to -2.52) |
| Chhatarpur | 70.0 (65.5-74.6) | 58.1 (53.3-62.9) | 36.6 (35.7-37.6) | -48.6 (-49.4 to -48.1) | -3.74 (-3.81 to -3.68) | -37.8 (-39.0 to -37.3) | -6.37 (-6.59 to -6.23) |
| Chhindwara | 60.0 (56.0-63.7) | 46.4 (39.7-52.4) | 33.2 (32.0-34.4) | -45.6 (-46.4 to -44.9) | -3.42 (-3.50 to -3.35) | -29.1 (-30.9 to -28.3) | -4.64 (-4.94 to -4.45) |
| Damoh | 69.2 (63.0-76.4) | 55.7 (50.0-61.3) | 35.1 (34.0-36.2) | -50.3 (-51.0 to -49.6) | -3.92 (-4.00 to -3.84) | -37.9 (-39.0 to -37.1) | -6.39 (-6.60 to -6.19) |
| Datia | 65.8 (61.2-70.3) | 51.6 (46.2-56.7) | 42.4 (41.5-43.3) | -36.6 (-37.3 to -35.8) | -2.56 (-2.62 to -2.49) | -18.6 (-20.2 to -17.8) | -2.75 (-3.00 to -2.59) |
| Dewas | 64.7 (60.8-68.3) | 53.6 (47.5-59.7) | 34.3 (33.6-35.0) | -47.9 (-48.6 to -47.5) | -3.66 (-3.73 to -3.61) | -36.8 (-38.0 to -35.9) | -6.16 (-6.38 to -5.96) |
| Dhar | 70.8 (67.2-74.9) | 54.9 (50.5-59.1) | 41.3 (40.3-42.2) | -42.6 (-43.1 to -42.1) | -3.12 (-3.17 to -3.07) | -25.6 (-26.6 to -24.6) | -3.97 (-4.13 to -3.77) |
| Dindori | 62.6 (55.0-70.2) | 58.5 (52.0-64.5) | 44.3 (43.3-45.3) | -29.9 (-31.4 to -29.0) | -2.00 (-2.11 to -1.92) | -25.0 (-26.5 to -24.4) | -3.87 (-4.12 to -3.74) |
| Guna | 71.2 (66.9-75.3) | 46.9 (41.0-53.5) | 40.4 (39.3-41.5) | -44.2 (-44.8 to -43.7) | -3.28 (-3.33 to -3.22) | -14.6 (-16.6 to -13.1) | -2.09 (-2.39 to -1.83) |
| Gwalior | 64.5 (58.9-69.9) | 47.2 (41.5-53.7) | 40.9 (40.2-41.8) | -37.4 (-38.4 to -36.8) | -2.64 (-2.72 to -2.58) | -13.9 (-16.1 to -12.9) | -1.99 (-2.31 to -1.80) |
| Harda | 64.6 (58.0-71.2) | 49.2 (42.5-56.2) | 29.3 (28.1-30.8) | -55.6 (-56.6 to -54.9) | -4.55 (-4.66 to -4.45) | -41.2 (-42.8 to -40.4) | -7.13 (-7.44 to -6.91) |
| Hoshangabad | 56.6 (52.4-60.9) | 49.7 (43.3-56.9) | 35.5 (34.7-36.5) | -38.3 (-39.1 to -37.7) | -2.71 (-2.78 to -2.66) | -29.3 (-30.9 to -28.3) | -4.67 (-4.94 to -4.46) |
| Indore | 60.3 (54.9-65.5) | 52.3 (45.8-58.5) | 32.1 (30.9-33.3) | -47.7 (-48.7 to -46.9) | -3.64 (-3.74 to -3.55) | -39.5 (-41.1 to -38.6) | -6.73 (-7.06 to -6.53) |
| Jabalpur | 61.8 (56.4-67.8) | 54.0 (49.4-58.9) | 37.4 (36.4-38.4) | -40.2 (-41.3 to -39.6) | -2.90 (-2.99 to -2.83) | -31.5 (-32.4 to -30.7) | -5.08 (-5.25 to -4.91) |
| Jhabua | 73.6 (68.3-78.7) | 49.8 (44.1-56.0) | 39.4 (38.2-40.7) | -47.4 (-48.1 to -46.9) | -3.61 (-3.68 to -3.55) | -21.6 (-23.3 to -20.4) | -3.28 (-3.54 to -3.03) |
| Katni | 60.8 (53.8-67.6) | 60.2 (54.1-65.5) | 39.8 (38.8-40.7) | -35.4 (-36.9 to -34.7) | -2.46 (-2.58 to -2.39) | -34.7 (-35.8 to -34.0) | -5.72 (-5.94 to -5.56) |
| Khandwa | 72.6 (68.0-77.1) | 52.6 (47.6-57.6) | 36.4 (35.8-37.2) | -50.8 (-51.5 to -50.5) | -3.97 (-4.05 to -3.93) | -31.6 (-32.7 to -30.7) | -5.10 (-5.29 to -4.92) |
| Khargone | 73.4 (68.0-78.8) | 56.9 (51.1-62.5) | 44.0 (43.1-44.9) | -41.0 (-41.7 to -40.5) | -2.97 (-3.03 to -2.92) | -23.5 (-24.9 to -22.6) | -3.60 (-3.82 to -3.41) |
| Mandla | 63.0 (57.3-68.7) | 56.2 (50.7-62.2) | 42.8 (41.9-43.8) | -32.9 (-33.8 to -32.2) | -2.24 (-2.31 to -2.18) | -24.6 (-26.1 to -23.6) | -3.79 (-4.04 to -3.59) |
| Mandsaur | 66.7 (61.8-71.5) | 50.6 (44.6-56.4) | 37.0 (36.1-38.0) | -45.4 (-46.3 to -44.8) | -3.40 (-3.48 to -3.34) | -27.6 (-29.1 to -26.5) | -4.34 (-4.60 to -4.12) |
| Morena | 60.3 (55.8-65.2) | 44.8 (38.8-51.0) | 39.0 (38.3-39.5) | -36.3 (-37.0 to -35.6) | -2.54 (-2.59 to -2.47) | -13.7 (-15.6 to -12.8) | -1.95 (-2.23 to -1.78) |
| Narsinghpur | 64.3 (59.3-69.2) | 48.3 (39.9-57.3) | 31.1 (29.8-32.6) | -52.6 (-53.4 to -52.1) | -4.18 (-4.27 to -4.12) | -36.1 (-38.3 to -35.4) | -6.04 (-6.44 to -5.84) |
| Neemuch | 70.3 (65.0-75.6) | 43.4 (37.8-49.1) | 36.1 (35.3-37.1) | -49.5 (-50.1 to -49.0) | -3.84 (-3.89 to -3.77) | -17.4 (-19.5 to -16.2) | -2.56 (-2.88 to -2.32) |
| Niwari | 70.8 (64.4-77.1) | 53.3 (47.0-59.3) | 41.9 (40.6-43.1) | -41.7 (-42.8 to -41.1) | -3.04 (-3.13 to -2.97) | -22.1 (-23.8 to -21.1) | -3.37 (-3.63 to -3.16) |
| Panna | 62.5 (56.4-68.5) | 59.5 (53.8-64.9) | 35.1 (34.0-36.3) | -44.7 (-45.9 to -44.0) | -3.33 (-3.45 to -3.25) | -41.8 (-42.9 to -41.4) | -7.24 (-7.46 to -7.12) |
| Raisen | 62.7 (59.1-66.5) | 57.0 (49.2-65.0) | 39.3 (38.4-40.3) | -38.3 (-38.9 to -37.6) | -2.71 (-2.76 to -2.65) | -31.7 (-33.0 to -30.9) | -5.13 (-5.36 to -4.94) |
| Rajgarh | 66.4 (61.3-70.7) | 53.7 (47.2-60.1) | 39.7 (38.7-40.5) | -41.2 (-41.9 to -40.4) | -2.98 (-3.05 to -2.90) | -26.8 (-28.7 to -25.8) | -4.21 (-4.53 to -3.99) |
| Ratlam | 67.0 (63.1-71.3) | 49.8 (44.9-54.2) | 35.5 (34.7-36.2) | -48.0 (-48.4 to -47.5) | -3.67 (-3.71 to -3.62) | -29.6 (-31.0 to -29.0) | -4.72 (-4.97 to -4.59) |
| Rewa | 61.1 (56.4-66.3) | 53.1 (47.9-58.5) | 32.2 (31.3-33.2) | -48.2 (-48.9 to -47.4) | -3.69 (-3.76 to -3.60) | -40.1 (-41.2 to -39.3) | -6.87 (-7.08 to -6.68) |
| Sagar | 71.1 (67.6-75.0) | 56.3 (48.9-63.8) | 36.5 (35.5-37.4) | -49.7 (-50.3 to -49.2) | -3.86 (-3.92 to -3.79) | -36.0 (-37.5 to -35.1) | -6.00 (-6.27 to -5.79) |
| Satna | 48.4 (43.4-53.4) | 51.0 (45.2-57.1) | 31.5 (30.6-32.5) | -35.7 (-36.9 to -34.7) | -2.49 (-2.59 to -2.39) | -39.0 (-40.0 to -38.1) | -6.63 (-6.82 to -6.41) |
| Sehore | 63.8 (60.1-67.6) | 53.7 (46.2-60.0) | 35.8 (35.3-36.4) | -44.7 (-45.2 to -44.2) | -3.33 (-3.38 to -3.28) | -34.0 (-35.8 to -33.3) | -5.59 (-5.93 to -5.43) |
| Seoni | 59.3 (55.0-64.1) | 49.9 (43.9-56.4) | 37.3 (36.2-38.3) | -38.0 (-38.9 to -37.2) | -2.68 (-2.77 to -2.61) | -25.9 (-27.5 to -24.9) | -4.04 (-4.31 to -3.82) |
| Shahdol | 56.7 (49.5-64.9) | 43.7 (36.4-50.5) | 31.3 (30.2-32.5) | -45.6 (-46.7 to -44.6) | -3.43 (-3.53 to -3.31) | -28.9 (-30.7 to -27.6) | -4.61 (-4.90 to -4.32) |
| Shajapur | 66.0 (60.1-71.5) | 53.8 (45.9-61.4) | 37.6 (36.2-38.8) | -43.9 (-44.9 to -43.3) | -3.25 (-3.34 to -3.18) | -30.8 (-32.7 to -30.0) | -4.97 (-5.31 to -4.77) |
| Sheopur | 70.7 (64.8-76.8) | 44.9 (38.1-51.3) | 41.8 (40.9-42.8) | -41.8 (-42.8 to -41.0) | -3.04 (-3.13 to -2.96) | -7.4 (-10.3 to -5.7) | -0.98 (-1.38 to -0.68) |
| Shivpuri | 71.8 (66.9-76.7) | 43.2 (37.2-49.2) | 38.1 (37.4-38.9) | -47.9 (-48.5 to -47.4) | -3.66 (-3.72 to -3.61) | -12.4 (-14.3 to -11.2) | -1.74 (-2.01 to -1.53) |
| Sidhi | 67.3 (62.4-72.4) | 51.4 (45.9-57.5) | 31.7 (30.8-32.7) | -53.9 (-54.5 to -53.3) | -4.33 (-4.40 to -4.26) | -39.1 (-40.2 to -38.1) | -6.66 (-6.85 to -6.41) |
| Singrauli | 73.5 (68.3-78.5) | 51.5 (44.2-59.3) | 36.7 (35.4-38.1) | -51.0 (-51.7 to -50.4) | -4.00 (-4.08 to -3.93) | -29.3 (-31.2 to -28.1) | -4.69 (-5.00 to -4.41) |
| Tikamgarh | 72.2 (67.6-76.7) | 56.1 (51.0-60.8) | 39.7 (38.6-40.5) | -46.0 (-46.6 to -45.6) | -3.46 (-3.52 to -3.41) | -30.2 (-31.3 to -29.5) | -4.83 (-5.02 to -4.69) |
| Ujjain | 61.9 (57.0-66.7) | 51.7 (45.3-57.8) | 31.7 (30.7-32.6) | -49.7 (-50.4 to -49.1) | -3.85 (-3.93 to -3.79) | -39.4 (-40.6 to -38.5) | -6.71 (-6.96 to -6.50) |
| Umaria | 60.9 (53.1-68.9) | 55.9 (49.6-62.4) | 38.5 (37.6-39.5) | -37.6 (-39.1 to -36.5) | -2.66 (-2.78 to -2.55) | -32.0 (-33.2 to -31.4) | -5.18 (-5.41 to -5.04) |
| Vidisha | 67.0 (63.4-70.7) | 56.6 (49.0-64.0) | 40.0 (39.0-41.3) | -41.2 (-41.8 to -40.4) | -2.98 (-3.04 to -2.91) | -29.8 (-31.3 to -28.7) | -4.78 (-5.02 to -4.53) |
| **Jharkhand** | **62.6 (58.0-66.5)** | **52.6 (49.7-56.4)** | **42.2 (40.5-44.8)** | **-33.5 (-34.1 to -33.1)** | **-2.29 (-2.33 to -2.25)** | **-20.7 (-21.5 to -20.1)** | **-3.15 (-4.20 to -2.19)** |
| Bokaro | 59.4 (51.7-67.0) | 52.8 (46.8-59.0) | 42.4 (41.4-43.4) | -29.3 (-30.9 to -28.4) | -1.95 (-2.07 to -1.87) | -20.4 (-22.1 to -19.5) | -3.05 (-3.34 to -2.88) |
| Chatra | 67.0 (58.2-75.2) | 51.4 (43.3-59.3) | 41.8 (41.1-42.8) | -38.4 (-39.7 to -37.7) | -2.73 (-2.84 to -2.65) | -19.1 (-21.5 to -17.9) | -2.86 (-3.22 to -2.62) |
| Deoghar | 63.7 (57.8-70.0) | 54.0 (46.9-60.7) | 41.6 (40.5-42.5) | -35.5 (-36.6 to -34.7) | -2.47 (-2.55 to -2.39) | -23.7 (-25.4 to -23.0) | -3.64 (-3.92 to -3.50) |
| Dhanbad | 58.9 (52.2-65.5) | 51.3 (44.9-57.2) | 40.7 (39.9-41.5) | -31.6 (-33.2 to -30.7) | -2.14 (-2.26 to -2.05) | -21.3 (-23.3 to -20.5) | -3.22 (-3.54 to -3.05) |
| Dumka | 67.6 (61.1-74.0) | 55.1 (48.5-60.4) | 42.9 (41.8-43.8) | -37.4 (-38.4 to -36.7) | -2.63 (-2.71 to -2.57) | -22.8 (-24.4 to -21.9) | -3.48 (-3.73 to -3.29) |
| East Singhbhum | 59.5 (51.4-68.1) | 51.7 (44.3-58.7) | 43.8 (42.6-45.0) | -26.9 (-28.8 to -25.7) | -1.77 (-1.90 to -1.65) | -15.8 (-18.2 to -14.0) | -2.30 (-2.67 to -1.97) |
| Garhwa | 64.8 (55.1-73.9) | 47.1 (38.7-55.8) | 39.9 (38.6-41.4) | -39.0 (-40.5 to -37.9) | -2.79 (-2.91 to -2.67) | -15.4 (-18.0 to -14.6) | -2.27 (-2.62 to -2.06) |
| Giridih | 62.3 (55.9-68.3) | 52.1 (45.8-58.4) | 40.1 (39.5-40.6) | -36.5 (-37.6 to -35.8) | -2.56 (-2.65 to -2.49) | -23.7 (-25.4 to -22.7) | -3.64 (-3.92 to -3.44) |
| Godda | 63.8 (54.2-72.9) | 50.9 (43.7-58.1) | 41.3 (40.1-42.4) | -35.9 (-37.6 to -35.0) | -2.52 (-2.65 to -2.42) | -19.5 (-21.8 to -18.4) | -2.92 (-3.28 to -2.69) |
| Gumla | 61.3 (53.6-69.8) | 56.8 (48.9-65.3) | 39.8 (39.1-40.8) | -35.7 (-37.4 to -34.7) | -2.49 (-2.63 to -2.39) | -30.5 (-32.3 to -29.5) | -4.90 (-5.23 to -4.68) |
| Hazaribagh | 64.2 (53.7-74.2) | 50.6 (41.8-59.0) | 39.6 (38.7-40.7) | -38.9 (-40.6 to -38.4) | -2.78 (-2.92 to -2.72) | -22.0 (-24.7 to -20.9) | -3.38 (-3.80 to -3.12) |
| Jamtara | 64.6 (59.3-70.3) | 52.7 (46.2-58.4) | 42.0 (41.0-42.9) | -35.9 (-36.8 to -35.2) | -2.50 (-2.57 to -2.43) | -21.1 (-23.0 to -20.4) | -3.18 (-3.49 to -3.03) |
| Khunti | 63.0 (56.5-69.6) | 59.6 (52.9-66.3) | 45.6 (44.7-46.7) | -28.4 (-29.6 to -27.7) | -1.88 (-1.96 to -1.81) | -24.3 (-25.7 to -23.2) | -3.73 (-3.98 to -3.52) |
| Koderma | 60.9 (53.3-68.7) | 49.3 (41.6-56.6) | 38.8 (37.6-39.7) | -37.1 (-38.7 to -36.5) | -2.61 (-2.75 to -2.55) | -21.8 (-24.6 to -20.4) | -3.33 (-3.78 to -3.04) |
| Latehar | 61.6 (53.1-69.9) | 52.6 (44.6-61.0) | 40.2 (39.4-41.1) | -35.5 (-37.2 to -34.8) | -2.47 (-2.61 to -2.40) | -24.1 (-26.4 to -22.9) | -3.73 (-4.10 to -3.46) |
| Lohardaga | 57.0 (46.9-67.8) | 54.8 (44.6-65.7) | 39.7 (38.3-41.0) | -30.5 (-33.4 to -29.2) | -2.07 (-2.28 to -1.93) | -27.7 (-30.3 to -26.3) | -4.41 (-4.83 to -4.08) |
| Pakur | 69.1 (62.4-76.4) | 51.2 (45.0-57.3) | 42.4 (41.1-43.5) | -39.5 (-40.5 to -38.7) | -2.83 (-2.91 to -2.75) | -17.9 (-19.6 to -16.6) | -2.64 (-2.89 to -2.40) |
| Palamu | 64.5 (55.3-74.1) | 48.9 (40.9-56.6) | 41.5 (40.6-42.8) | -36.2 (-37.9 to -35.0) | -2.54 (-2.67 to -2.42) | -15.5 (-17.9 to -14.3) | -2.26 (-2.61 to -2.02) |
| Ramgarh | 60.5 (52.0-69.4) | 52.1 (44.6-59.3) | 41.9 (40.9-42.9) | -31.3 (-33.1 to -30.2) | -2.12 (-2.25 to -2.01) | -20.2 (-22.1 to -19.3) | -3.03 (-3.33 to -2.85) |
| Ranchi | 59.0 (52.2-66.0) | 55.2 (48.5-62.1) | 43.7 (42.8-44.5) | -26.6 (-27.9 to -25.6) | -1.74 (-1.83 to -1.65) | -21.6 (-23.1 to -20.5) | -3.26 (-3.51 to -3.06) |
| Sahibganj | 65.7 (58.5-72.8) | 44.0 (38.9-49.1) | 39.4 (38.5-40.5) | -40.8 (-42.0 to -39.9) | -2.95 (-3.05 to -2.86) | -11.0 (-12.5 to -9.8) | -1.52 (-1.74 to -1.30) |
| Saraikela Kharsawan | 61.3 (54.1-69.0) | 57.2 (50.7-62.6) | 48.7 (47.1-49.8) | -21.3 (-22.8 to -20.1) | -1.34 (-1.43 to -1.24) | -15.7 (-17.4 to -14.8) | -2.27 (-2.52 to -2.09) |
| Simdega | 69.1 (61.3-76.4) | 58.5 (51.5-65.5) | 40.6 (39.5-42.0) | -42.0 (-43.2 to -41.4) | -3.07 (-3.18 to -3.00) | -31.3 (-33.0 to -30.7) | -5.06 (-5.36 to -4.92) |
| West Singhbhum | 65.2 (59.3-71.3) | 61.1 (55.5-67.0) | 51.0 (49.9-52.1) | -22.6 (-23.8 to -21.5) | -1.43 (-1.51 to -1.34) | -17.3 (-18.6 to -16.5) | -2.52 (-2.73 to -2.37) |
| **Uttar Pradesh** | **54.8 (51.2-59.1)** | **45.0 (43.4-47.3)** | **36.4 (35.2-37.8)** | **-34.6 (-34.8 to -34.4)** | **-2.38 (-2.40 to -2.36)** | **-20.1 (-20.4 to -19.7)** | **-3.03 (-3.76 to -2.36)** |
| Agra | 47.4 (42.8-52.1) | 39.5 (33.5-46.1) | 31.1 (30.2-32.2) | -35.2 (-36.2 to -34.3) | -2.44 (-2.52 to -2.35) | -21.7 (-23.8 to -20.4) | -3.31 (-3.63 to -3.04) |
| Aligarh | 51.4 (46.4-55.9) | 39.5 (33.3-45.9) | 35.0 (34.0-36.0) | -32.8 (-33.9 to -32.3) | -2.23 (-2.32 to -2.18) | -11.7 (-14.1 to -10.4) | -1.66 (-1.99 to -1.40) |
| Allahabad | 61.8 (57.6-66.6) | 50.9 (46.8-55.0) | 40.5 (39.8-41.2) | -35.5 (-36.3 to -34.7) | -2.46 (-2.53 to -2.39) | -21.4 (-22.5 to -20.8) | -3.22 (-3.40 to -3.10) |
| Ambedkar Nagar | 56.9 (51.5-62.2) | 47.2 (42.0-52.3) | 36.4 (35.6-37.1) | -37.0 (-37.9 to -36.2) | -2.60 (-2.67 to -2.52) | -23.8 (-24.9 to -22.6) | -3.65 (-3.83 to -3.41) |
| Amethi | 57.8 (53.2-62.3) | 51.5 (47.0-55.8) | 42.8 (41.7-43.8) | -26.9 (-27.8 to -26.3) | -1.75 (-1.82 to -1.70) | -17.7 (-18.9 to -16.9) | -2.60 (-2.77 to -2.44) |
| Amroha | 48.3 (42.2-54.5) | 37.8 (32.2-43.4) | 33.9 (33.1-34.9) | -30.4 (-31.7 to -29.2) | -2.04 (-2.13 to -1.93) | -10.9 (-13.1 to -9.7) | -1.51 (-1.83 to -1.29) |
| Auraiya | 57.4 (52.6-62.8) | 51.3 (44.2-58.5) | 37.6 (36.7-38.7) | -35.3 (-36.5 to -34.5) | -2.45 (-2.54 to -2.37) | -27.2 (-29.3 to -25.8) | -4.29 (-4.64 to -3.98) |
| Azamgarh | 55.2 (51.6-58.9) | 40.9 (36.3-45.1) | 34.2 (33.7-34.8) | -38.9 (-39.7 to -38.3) | -2.77 (-2.84 to -2.71) | -17.2 (-18.5 to -16.0) | -2.51 (-2.71 to -2.30) |
| Baghpat | 54.7 (49.2-60.4) | 35.1 (30.0-40.9) | 32.1 (31.0-33.0) | -42.2 (-43.3 to -41.4) | -3.08 (-3.18 to -3.00) | -8.9 (-11.2 to -7.4) | -1.21 (-1.52 to -0.94) |
| Bahraich | 53.1 (48.1-58.2) | 53.0 (48.5-57.7) | 42.4 (41.5-43.5) | -20.9 (-21.9 to -20.1) | -1.30 (-1.37 to -1.24) | -20.8 (-22.1 to -19.9) | -3.12 (-3.33 to -2.94) |
| Ballia | 53.6 (49.3-57.8) | 39.0 (34.0-44.2) | 32.4 (31.9-32.9) | -40.4 (-41.4 to -39.7) | -2.91 (-2.99 to -2.84) | -17.5 (-19.4 to -16.1) | -2.57 (-2.86 to -2.31) |
| Balrampur | 60.5 (54.1-67.3) | 51.7 (45.3-57.6) | 41.1 (40.0-42.4) | -32.8 (-34.1 to -31.9) | -2.24 (-2.34 to -2.15) | -21.1 (-22.9 to -20.4) | -3.18 (-3.46 to -3.03) |
| Banda | 62.0 (56.7-67.5) | 58.4 (53.3-63.6) | 38.5 (37.9-39.7) | -38.7 (-39.5 to -37.9) | -2.75 (-2.81 to -2.68) | -34.9 (-35.7 to -34.2) | -5.75 (-5.91 to -5.60) |
| Barabanki | 55.0 (51.8-58.6) | 52.1 (48.3-56.0) | 41.0 (40.1-41.9) | -26.3 (-27.2 to -25.5) | -1.71 (-1.77 to -1.64) | -22.1 (-23.2 to -21.4) | -3.34 (-3.52 to -3.20) |
| Bareilly | 46.6 (42.0-51.8) | 39.4 (35.2-44.0) | 37.2 (36.6-38.0) | -20.9 (-22.2 to -19.9) | -1.30 (-1.39 to -1.23) | -6.2 (-7.6 to -4.8) | -0.78 (-0.97 to -0.55) |
| Basti | 57.7 (52.2-63.3) | 48.8 (43.2-54.5) | 37.3 (36.6-38.0) | -36.2 (-37.5 to -35.4) | -2.53 (-2.64 to -2.45) | -24.3 (-25.4 to -23.3) | -3.74 (-3.92 to -3.55) |
| Bhadohi | 57.9 (52.9-63.8) | 43.9 (39.2-48.4) | 41.2 (40.0-42.5) | -29.6 (-30.6 to -28.6) | -1.97 (-2.04 to -1.88) | -6.8 (-8.6 to -5.9) | -0.88 (-1.12 to -0.72) |
| Bijnor | 44.0 (38.9-49.4) | 33.8 (28.7-39.5) | 31.6 (30.6-32.6) | -29.0 (-30.2 to -27.9) | -1.92 (-2.01 to -1.83) | -6.8 (-9.5 to -5.3) | -0.90 (-1.26 to -0.62) |
| Budaun | 50.5 (46.4-55.0) | 42.8 (37.9-48.4) | 38.0 (37.7-38.5) | -25.5 (-26.7 to -24.6) | -1.65 (-1.73 to -1.57) | -11.8 (-13.5 to -10.3) | -1.64 (-1.89 to -1.38) |
| Bulandshahr | 49.8 (45.1-54.5) | 38.0 (33.3-43.1) | 33.1 (32.5-33.7) | -34.4 (-35.5 to -33.6) | -2.37 (-2.46 to -2.29) | -13.5 (-15.6 to -12.2) | -1.92 (-2.23 to -1.68) |
| Chandauli | 53.5 (48.5-59.3) | 36.4 (31.3-41.8) | 37.8 (36.7-38.7) | -30.1 (-31.2 to -29.2) | -2.01 (-2.09 to -1.93) | 3.5 (1.0 to 5.2) | 0.60 (0.29 to 0.86) |
| Chitrakoot | 57.9 (53.0-62.9) | 54.7 (50.1-59.7) | 38.6 (37.5-39.6) | -34.2 (-34.9 to -33.5) | -2.35 (-2.40 to -2.28) | -30.3 (-31.5 to -29.5) | -4.86 (-5.07 to -4.67) |
| Deoria | 53.8 (48.1-59.3) | 38.3 (31.7-45.0) | 29.2 (28.3-30.3) | -46.5 (-47.6 to -45.7) | -3.52 (-3.62 to -3.43) | -24.2 (-26.7 to -22.9) | -3.75 (-4.15 to -3.46) |
| Etah | 55.6 (50.8-60.1) | 45.8 (39.2-52.7) | 33.6 (33.0-34.2) | -40.5 (-41.2 to -39.8) | -2.92 (-2.98 to -2.85) | -27.2 (-28.9 to -26.0) | -4.29 (-4.57 to -4.02) |
| Etawah | 60.8 (55.4-66.4) | 51.6 (43.9-59.0) | 35.2 (34.2-36.0) | -43.0 (-44.1 to -42.4) | -3.16 (-3.27 to -3.09) | -32.3 (-34.1 to -31.2) | -5.27 (-5.58 to -5.01) |
| Faizabad | 56.3 (51.6-60.6) | 49.3 (45.0-53.6) | 40.1 (39.2-41.0) | -29.6 (-30.8 to -28.9) | -1.97 (-2.06 to -1.90) | -19.4 (-20.8 to -18.2) | -2.88 (-3.10 to -2.66) |
| Farrukhabad | 56.7 (51.7-61.1) | 49.0 (42.7-55.4) | 35.3 (34.3-36.1) | -38.7 (-39.7 to -37.9) | -2.75 (-2.83 to -2.67) | -28.7 (-30.3 to -27.3) | -4.55 (-4.84 to -4.26) |
| Fatehpur | 64.0 (59.9-68.3) | 57.0 (52.6-61.2) | 40.4 (39.7-41.2) | -37.7 (-38.4 to -37.2) | -2.66 (-2.72 to -2.61) | -29.9 (-30.8 to -29.0) | -4.77 (-4.93 to -4.58) |
| Firozabad | 51.5 (47.2-57.0) | 43.5 (37.1-50.7) | 30.4 (29.5-31.5) | -41.8 (-42.8 to -40.9) | -3.05 (-3.14 to -2.95) | -30.5 (-32.3 to -29.3) | -4.92 (-5.21 to -4.64) |
| Gautam Buddha Nagar | 45.2 (39.9-50.3) | 30.6 (25.7-35.8) | 26.5 (25.4-28.0) | -42.0 (-43.4 to -41.3) | -3.08 (-3.20 to -2.98) | -13.6 (-16.4 to -11.4) | -1.96 (-2.37 to -1.56) |
| Ghaziabad | 49.5 (44.6-54.5) | 32.7 (28.3-37.5) | 28.4 (27.2-29.6) | -43.5 (-44.3 to -42.7) | -3.21 (-3.28 to -3.13) | -13.6 (-16.0 to -12.2) | -1.95 (-2.29 to -1.68) |
| Ghazipur | 52.1 (48.0-57.0) | 36.1 (31.6-40.9) | 32.3 (31.6-33.0) | -39.0 (-39.9 to -38.4) | -2.78 (-2.86 to -2.72) | -11.3 (-13.3 to -9.6) | -1.57 (-1.85 to -1.27) |
| Gonda | 55.9 (50.4-62.0) | 51.5 (46.3-56.4) | 41.6 (40.8-42.3) | -26.5 (-27.7 to -25.7) | -1.73 (-1.81 to -1.66) | -20.1 (-21.7 to -19.4) | -3.01 (-3.26 to -2.87) |
| Gorakhpur | 56.1 (52.0-60.3) | 41.3 (36.4-46.4) | 31.0 (30.3-31.5) | -45.7 (-46.3 to -45.2) | -3.43 (-3.48 to -3.37) | -25.8 (-27.5 to -24.8) | -4.01 (-4.31 to -3.81) |
| Hamirpur | 61.1 (55.5-66.1) | 57.9 (51.8-63.8) | 42.2 (41.1-43.3) | -31.8 (-33.0 to -31.2) | -2.15 (-2.24 to -2.09) | -27.9 (-29.0 to -26.8) | -4.39 (-4.58 to -4.17) |
| Hapur | 49.7 (43.6-56.2) | 35.9 (30.1-41.3) | 31.5 (30.4-32.6) | -37.4 (-38.6 to -36.0) | -2.64 (-2.73 to -2.50) | -12.9 (-15.4 to -11.3) | -1.84 (-2.20 to -1.54) |
| Hardoi | 54.0 (50.1-58.0) | 50.7 (45.1-56.6) | 37.4 (36.6-38.1) | -31.7 (-32.6 to -30.8) | -2.14 (-2.21 to -2.06) | -27.0 (-28.3 to -25.9) | -4.23 (-4.45 to -4.00) |
| Hathras | 50.6 (46.0-54.6) | 40.2 (33.8-47.0) | 34.4 (33.6-35.2) | -33.0 (-34.2 to -32.5) | -2.25 (-2.34 to -2.20) | -14.9 (-16.9 to -13.1) | -2.16 (-2.44 to -1.82) |
| Jalaun | 59.3 (54.7-64.0) | 53.6 (47.0-60.4) | 41.0 (40.2-42.0) | -31.7 (-32.7 to -30.9) | -2.14 (-2.22 to -2.07) | -24.1 (-25.7 to -23.2) | -3.72 (-3.97 to -3.51) |
| Jaunpur | 58.4 (54.8-62.4) | 45.0 (40.9-48.9) | 39.6 (39.1-40.2) | -33.0 (-33.8 to -32.5) | -2.25 (-2.31 to -2.20) | -12.8 (-13.9 to -12.0) | -1.79 (-1.95 to -1.65) |
| Jhansi | 64.5 (59.9-68.7) | 54.8 (49.4-59.6) | 43.0 (42.1-44.0) | -34.3 (-35.0 to -33.7) | -2.36 (-2.41 to -2.31) | -22.4 (-24.0 to -21.5) | -3.40 (-3.66 to -3.22) |
| Kannauj | 56.0 (52.1-60.5) | 50.3 (44.3-56.3) | 36.6 (35.8-37.3) | -35.5 (-36.4 to -34.8) | -2.46 (-2.54 to -2.40) | -27.8 (-29.5 to -26.4) | -4.39 (-4.67 to -4.09) |
| Kanpur Dehat | 59.3 (55.4-63.5) | 53.9 (47.6-60.4) | 37.6 (36.9-38.3) | -37.6 (-38.3 to -36.9) | -2.65 (-2.71 to -2.58) | -31.1 (-32.2 to -29.7) | -5.00 (-5.21 to -4.73) |
| Kanpur Nagar | 57.8 (52.4-63.5) | 53.7 (46.6-60.9) | 33.5 (32.3-34.7) | -42.9 (-43.8 to -42.0) | -3.15 (-3.24 to -3.06) | -38.3 (-40.0 to -37.2) | -6.48 (-6.81 to -6.23) |
| Kasganj | 55.6 (50.1-61.1) | 46.0 (39.1-53.8) | 37.0 (35.9-37.9) | -34.3 (-35.6 to -33.4) | -2.36 (-2.46 to -2.28) | -20.0 (-22.1 to -18.1) | -3.01 (-3.33 to -2.65) |
| Kaushambi | 62.7 (57.7-67.5) | 52.7 (48.2-57.1) | 42.1 (40.8-43.3) | -33.7 (-34.7 to -32.7) | -2.31 (-2.39 to -2.22) | -21.1 (-22.4 to -20.2) | -3.17 (-3.37 to -3.01) |
| Khushinagar | 55.5 (51.1-60.1) | 39.3 (34.3-44.5) | 32.4 (31.5-33.0) | -42.6 (-43.5 to -41.9) | -3.12 (-3.21 to -3.05) | -18.3 (-19.9 to -17.4) | -2.71 (-2.95 to -2.52) |
| Lakhimpur Kheri | 54.1 (50.3-58.0) | 54.2 (49.3-58.9) | 43.5 (42.3-44.7) | -20.4 (-21.5 to -19.7) | -1.26 (-1.34 to -1.21) | -20.5 (-21.9 to -19.5) | -3.07 (-3.30 to -2.89) |
| Lalitpur | 70.6 (66.2-75.0) | 52.2 (45.8-58.7) | 38.3 (37.4-39.4) | -46.7 (-47.3 to -46.1) | -3.53 (-3.59 to -3.47) | -27.3 (-28.8 to -26.4) | -4.30 (-4.54 to -4.10) |
| Lucknow | 54.8 (50.6-59.3) | 51.5 (46.4-56.3) | 36.5 (35.3-37.7) | -34.3 (-35.1 to -33.4) | -2.36 (-2.42 to -2.27) | -29.9 (-31.2 to -29.0) | -4.78 (-5.00 to -4.59) |
| Maharajganj | 61.6 (58.0-65.3) | 42.9 (38.0-47.7) | 35.1 (33.8-36.1) | -44.1 (-44.8 to -43.2) | -3.26 (-3.33 to -3.18) | -19.1 (-20.7 to -18.0) | -2.83 (-3.08 to -2.62) |
| Mahoba | 63.9 (59.4-68.7) | 58.1 (52.5-64.2) | 41.4 (40.2-42.1) | -36.2 (-37.0 to -35.4) | -2.52 (-2.59 to -2.45) | -29.6 (-30.9 to -29.0) | -4.72 (-4.95 to -4.59) |
| Mainpuri | 61.1 (54.0-67.7) | 50.7 (43.2-58.5) | 31.8 (30.8-32.9) | -48.9 (-49.9 to -47.9) | -3.77 (-3.87 to -3.65) | -38.0 (-40.0 to -37.2) | -6.43 (-6.82 to -6.21) |
| Mathura | 48.9 (44.1-53.2) | 35.5 (30.3-41.1) | 33.3 (32.2-34.1) | -32.7 (-33.6 to -32.0) | -2.23 (-2.30 to -2.16) | -6.6 (-9.1 to -5.1) | -0.87 (-1.19 to -0.59) |
| Mau | 53.5 (49.1-57.8) | 38.5 (33.5-43.5) | 30.5 (29.6-31.5) | -43.8 (-44.7 to -43.1) | -3.24 (-3.32 to -3.17) | -21.3 (-23.1 to -19.7) | -3.23 (-3.51 to -2.92) |
| Meerut | 55.0 (48.8-61.1) | 35.7 (30.4-40.9) | 31.0 (30.3-31.7) | -44.5 (-45.8 to -43.9) | -3.31 (-3.43 to -3.24) | -13.7 (-16.0 to -12.7) | -1.96 (-2.29 to -1.77) |
| Mirzapur | 62.3 (58.6-66.0) | 48.1 (42.4-53.6) | 42.1 (41.3-43.0) | -33.3 (-34.1 to -32.8) | -2.28 (-2.34 to -2.23) | -13.1 (-14.6 to -12.0) | -1.85 (-2.07 to -1.65) |
| Moradabad | 43.9 (39.9-48.4) | 35.9 (32.0-40.1) | 34.0 (33.4-34.6) | -23.5 (-24.6 to -22.4) | -1.49 (-1.57 to -1.40) | -6.0 (-7.7 to -4.6) | -0.75 (-0.99 to -0.52) |
| Muzaffarnagar | 53.5 (48.1-58.5) | 34.4 (29.3-39.5) | 30.8 (30.3-31.6) | -43.2 (-44.2 to -42.6) | -3.19 (-3.28 to -3.11) | -10.9 (-13.1 to -9.4) | -1.52 (-1.82 to -1.25) |
| Pilibhit | 50.2 (45.9-54.9) | 41.4 (36.0-46.9) | 37.8 (36.7-39.0) | -25.5 (-26.8 to -24.7) | -1.65 (-1.74 to -1.58) | -9.3 (-11.5 to -8.0) | -1.27 (-1.57 to -1.04) |
| Pratapgarh | 60.0 (56.5-63.7) | 50.9 (47.8-54.2) | 43.2 (42.7-43.8) | -28.8 (-29.5 to -28.2) | -1.90 (-1.95 to -1.85) | -16.0 (-16.7 to -15.2) | -2.31 (-2.41 to -2.17) |
| Rae Bareli | 60.5 (57.2-64.1) | 54.3 (50.1-58.6) | 40.4 (39.7-41.1) | -34.1 (-34.9 to -33.6) | -2.34 (-2.41 to -2.29) | -26.4 (-27.4 to -25.6) | -4.12 (-4.29 to -3.95) |
| Rampur | 43.8 (39.0-48.8) | 37.9 (33.6-42.5) | 36.0 (35.2-37.0) | -18.5 (-20.0 to -17.4) | -1.14 (-1.23 to -1.05) | -5.5 (-7.5 to -4.1) | -0.69 (-0.96 to -0.44) |
| Saharanpur | 47.0 (42.6-51.5) | 32.5 (26.8-38.4) | 31.8 (31.0-32.4) | -33.2 (-34.2 to -32.4) | -2.27 (-2.35 to -2.19) | -2.4 (-5.1 to -0.5) | -0.26 (-0.60 to 0.07) |
| Sambhal | 48.9 (43.9-54.2) | 39.1 (34.2-44.6) | 35.7 (35.0-36.3) | -27.8 (-28.8 to -26.5) | -1.82 (-1.90 to -1.72) | -9.3 (-10.9 to -8.0) | -1.26 (-1.48 to -1.04) |
| Sant Kabir Nagar | 57.8 (52.4-63.3) | 44.8 (39.2-50.4) | 33.3 (32.3-34.1) | -43.3 (-44.1 to -42.6) | -3.19 (-3.26 to -3.12) | -26.3 (-28.1 to -25.1) | -4.12 (-4.42 to -3.87) |
| Shahjahanpur | 50.3 (46.7-54.1) | 44.4 (39.3-50.0) | 37.6 (37.0-38.4) | -26.2 (-27.1 to -25.2) | -1.70 (-1.76 to -1.62) | -15.9 (-17.7 to -14.8) | -2.31 (-2.58 to -2.11) |
| Shamli | 51.1 (46.6-55.7) | 33.4 (28.0-38.9) | 32.9 (32.3-33.6) | -36.3 (-37.5 to -35.4) | -2.54 (-2.64 to -2.45) | -1.5 (-4.3 to 0.1) | -0.13 (-0.48 to 0.15) |
| Shravasti | 54.5 (48.6-60.2) | 52.8 (47.9-57.9) | 41.6 (40.7-42.6) | -24.5 (-25.6 to -23.6) | -1.58 (-1.65 to -1.49) | -22.1 (-23.4 to -21.3) | -3.35 (-3.56 to -3.19) |
| Siddharth Nagar | 62.7 (57.4-68.0) | 47.3 (42.1-52.6) | 37.6 (36.7-38.6) | -41.0 (-41.8 to -40.3) | -2.97 (-3.04 to -2.90) | -21.3 (-22.7 to -20.2) | -3.21 (-3.43 to -3.00) |
| Sitapur | 53.2 (49.8-56.7) | 54.6 (50.3-58.7) | 41.3 (40.3-42.1) | -23.3 (-24.2 to -22.5) | -1.48 (-1.54 to -1.41) | -25.3 (-26.4 to -24.3) | -3.90 (-4.09 to -3.73) |
| Sonbhadra | 63.9 (59.2-68.2) | 50.5 (42.9-57.8) | 42.7 (41.6-44.2) | -34.1 (-35.2 to -33.5) | -2.35 (-2.43 to -2.28) | -15.9 (-18.2 to -14.7) | -2.32 (-2.65 to -2.09) |
| Sultanpur | 57.7 (53.6-61.2) | 49.5 (46.1-53.2) | 40.9 (40.2-41.6) | -29.9 (-30.8 to -29.3) | -1.99 (-2.06 to -1.94) | -18.2 (-19.2 to -17.5) | -2.66 (-2.83 to -2.54) |
| Unnao | 60.5 (56.3-65.5) | 55.6 (49.2-62.0) | 36.7 (35.7-37.6) | -40.3 (-40.9 to -39.4) | -2.90 (-2.95 to -2.81) | -34.8 (-36.0 to -33.9) | -5.75 (-5.97 to -5.54) |
| Varanasi | 54.0 (47.7-61.1) | 36.0 (30.0-42.5) | 38.1 (37.0-39.3) | -30.2 (-31.6 to -29.0) | -2.02 (-2.12 to -1.91) | 5.8 (3.0 to 7.1) | 0.89 (0.56 to 1.12) |
| **Rajasthan** | **55.1 (51.7-59.0)** | **41.9 (40.6-43.6)** | **33.2 (32.2-34.4)** | **-40.7 (-40.9 to -40.4)** | **-2.93 (-2.95 to -2.91)** | **-21.6 (-22.3 to -20.9)** | **-3.31 (-3.86 to -2.77)** |
| Ajmer | 58.0 (54.1-61.6) | 36.5 (29.7-44.5) | 35.5 (34.4-36.8) | -39.8 (-40.6 to -39.1) | -2.85 (-2.93 to -2.79) | -2.6 (-6.5 to -0.8) | -0.30 (-0.81 to 0.03) |
| Alwar | 42.0 (38.5-45.6) | 37.0 (31.9-42.5) | 29.6 (28.9-30.4) | -30.4 (-31.5 to -29.5) | -2.04 (-2.12 to -1.95) | -20.6 (-22.8 to -19.6) | -3.11 (-3.46 to -2.90) |
| Banswara | 58.5 (55.1-62.2) | 54.2 (48.0-60.0) | 42.9 (42.2-44.1) | -27.6 (-28.4 to -26.9) | -1.81 (-1.87 to -1.75) | -21.6 (-23.1 to -20.6) | -3.27 (-3.50 to -3.07) |
| Baran | 58.5 (55.0-62.2) | 51.4 (44.7-57.3) | 41.6 (40.6-42.3) | -29.8 (-30.7 to -29.2) | -1.99 (-2.05 to -1.93) | -19.7 (-21.7 to -18.7) | -2.94 (-3.26 to -2.74) |
| Barmer | 67.6 (64.2-70.9) | 45.2 (36.9-54.5) | 38.3 (36.9-39.6) | -44.3 (-44.9 to -43.6) | -3.29 (-3.35 to -3.21) | -15.4 (-18.5 to -13.7) | -2.26 (-2.71 to -1.92) |
| Bharatpur | 43.6 (39.5-47.6) | 39.1 (33.5-45.3) | 34.3 (33.3-35.1) | -22.2 (-23.6 to -21.2) | -1.40 (-1.50 to -1.32) | -12.7 (-15.2 to -11.1) | -1.81 (-2.17 to -1.52) |
| Bhilwara | 56.7 (53.4-59.8) | 39.1 (32.9-45.4) | 36.3 (35.1-37.7) | -36.9 (-37.9 to -36.4) | -2.59 (-2.67 to -2.54) | -7.7 (-10.4 to -6.2) | -1.03 (-1.40 to -0.76) |
| Bikaner | 64.9 (61.4-68.0) | 29.8 (23.4-36.6) | 28.3 (27.5-29.3) | -57.3 (-57.8 to -56.9) | -4.76 (-4.82 to -4.70) | -4.9 (-8.6 to -2.9) | -0.64 (-1.12 to -0.27) |
| Bundi | 60.3 (56.8-64.1) | 42.5 (35.2-50.2) | 36.8 (35.8-37.8) | -40.0 (-40.7 to -39.3) | -2.87 (-2.93 to -2.81) | -13.7 (-16.5 to -12.5) | -1.98 (-2.38 to -1.73) |
| Chittorgarh | 56.6 (52.9-60.1) | 43.6 (37.4-49.6) | 36.1 (35.0-37.4) | -37.2 (-38.1 to -36.5) | -2.61 (-2.69 to -2.54) | -17.8 (-19.9 to -16.5) | -2.63 (-2.94 to -2.38) |
| Churu | 59.5 (56.0-62.7) | 35.3 (29.4-41.4) | 26.3 (25.4-27.3) | -56.8 (-57.3 to -56.1) | -4.69 (-4.75 to -4.61) | -26.0 (-28.0 to -24.8) | -4.08 (-4.39 to -3.80) |
| Dausa | 46.8 (40.4-53.2) | 35.6 (28.3-43.0) | 26.4 (25.4-27.3) | -44.3 (-45.9 to -43.7) | -3.30 (-3.44 to -3.22) | -26.0 (-29.3 to -24.3) | -4.10 (-4.64 to -3.73) |
| Dholpur | 47.3 (42.8-51.8) | 47.8 (41.9-54.5) | 38.2 (37.2-39.0) | -20.0 (-21.1 to -19.2) | -1.24 (-1.31 to -1.17) | -20.8 (-22.7 to -19.3) | -3.13 (-3.44 to -2.84) |
| Dungarpur | 55.2 (50.7-59.6) | 58.3 (51.3-65.6) | 46.7 (45.5-47.8) | -16.2 (-17.6 to -15.4) | -0.97 (-1.06 to -0.91) | -20.5 (-22.3 to -19.2) | -3.09 (-3.36 to -2.83) |
| Hanumangarh | 49.7 (44.8-54.2) | 42.4 (35.0-51.0) | 30.1 (29.0-31.2) | -40.3 (-41.5 to -39.4) | -2.91 (-3.01 to -2.81) | -29.5 (-31.8 to -27.9) | -4.73 (-5.11 to -4.38) |
| Jaipur | 47.1 (43.0-50.9) | 33.0 (27.0-40.0) | 24.4 (23.7-25.3) | -49.1 (-50.0 to -48.4) | -3.79 (-3.89 to -3.71) | -26.3 (-28.2 to -24.8) | -4.15 (-4.44 to -3.81) |
| Jaisalmer | 68.8 (65.3-72.2) | 34.0 (26.0-43.4) | 30.3 (29.0-31.7) | -56.9 (-57.5 to -56.2) | -4.71 (-4.78 to -4.62) | -10.5 (-14.8 to -8.2) | -1.53 (-2.10 to -1.07) |
| Jalore | 66.3 (62.7-70.1) | 59.0 (51.8-66.4) | 42.4 (41.3-43.4) | -37.0 (-37.6 to -36.4) | -2.59 (-2.65 to -2.54) | -28.9 (-30.4 to -28.2) | -4.59 (-4.86 to -4.44) |
| Jhalawar | 53.2 (49.3-57.1) | 58.6 (51.7-65.5) | 38.6 (37.2-39.8) | -28.2 (-29.3 to -27.3) | -1.86 (-1.94 to -1.78) | -34.8 (-36.5 to -33.8) | -5.76 (-6.08 to -5.52) |
| Jhunjhunu | 48.4 (44.7-52.3) | 35.7 (29.3-42.6) | 23.1 (22.0-24.1) | -53.2 (-54.1 to -52.5) | -4.26 (-4.36 to -4.17) | -35.7 (-37.9 to -34.9) | -5.98 (-6.37 to -5.74) |
| Jodhpur | 62.1 (57.4-67.5) | 36.2 (30.4-42.6) | 32.3 (31.1-33.5) | -48.8 (-49.5 to -48.0) | -3.76 (-3.83 to -3.66) | -11.0 (-13.6 to -9.4) | -1.55 (-1.91 to -1.24) |
| Karauli | 51.5 (45.8-56.7) | 44.7 (37.8-51.3) | 35.9 (34.9-36.8) | -31.1 (-32.5 to -30.2) | -2.10 (-2.20 to -2.01) | -20.3 (-22.5 to -19.3) | -3.06 (-3.40 to -2.85) |
| Kota | 59.0 (54.8-63.4) | 47.8 (40.0-55.5) | 37.6 (36.3-38.8) | -37.2 (-38.0 to -36.4) | -2.61 (-2.68 to -2.53) | -21.8 (-23.9 to -20.4) | -3.32 (-3.65 to -3.03) |
| Nagaur | 61.9 (58.7-64.9) | 31.3 (24.8-38.0) | 27.9 (26.8-29.0) | -55.8 (-56.5 to -55.2) | -4.58 (-4.65 to -4.49) | -10.8 (-13.5 to -8.9) | -1.53 (-1.89 to -1.17) |
| Pali | 59.1 (54.3-63.3) | 50.9 (44.9-57.2) | 39.3 (38.1-40.4) | -34.4 (-35.3 to -33.6) | -2.37 (-2.44 to -2.29) | -23.5 (-24.8 to -22.3) | -3.60 (-3.80 to -3.37) |
| Pratapgarh | 58.7 (53.8-63.5) | 55.4 (48.5-62.1) | 42.0 (40.8-43.4) | -29.4 (-30.3 to -28.7) | -1.95 (-2.02 to -1.89) | -24.9 (-26.5 to -23.9) | -3.85 (-4.11 to -3.65) |
| Rajsamand | 53.9 (48.1-59.9) | 53.6 (46.1-61.0) | 38.9 (37.8-40.2) | -28.7 (-30.0 to -27.7) | -1.90 (-2.00 to -1.81) | -28.1 (-29.9 to -27.2) | -4.45 (-4.75 to -4.25) |
| Sawai Madhopur | 54.4 (49.4-59.2) | 41.3 (35.0-48.3) | 33.3 (32.3-34.0) | -39.7 (-40.5 to -38.9) | -2.84 (-2.92 to -2.76) | -19.9 (-22.4 to -18.5) | -2.99 (-3.38 to -2.70) |
| Sikar | 50.7 (47.8-53.9) | 33.8 (28.6-40.2) | 23.8 (23.0-24.5) | -54.1 (-54.6 to -53.4) | -4.36 (-4.41 to -4.28) | -30.1 (-32.2 to -28.3) | -4.85 (-5.20 to -4.45) |
| Sirohi | 62.6 (59.0-66.8) | 65.8 (58.8-72.4) | 43.3 (41.8-44.5) | -31.8 (-32.6 to -31.0) | -2.15 (-2.21 to -2.08) | -35.0 (-36.5 to -34.2) | -5.79 (-6.06 to -5.60) |
| Sri Ganganagar | 52.9 (47.1-58.6) | 36.9 (31.4-43.2) | 29.2 (28.0-30.8) | -45.7 (-47.0 to -45.0) | -3.44 (-3.56 to -3.36) | -21.3 (-24.0 to -19.6) | -3.25 (-3.67 to -2.90) |
| Tonk | 57.1 (51.9-62.0) | 37.8 (30.1-46.2) | 32.9 (31.6-34.0) | -43.3 (-44.3 to -42.6) | -3.19 (-3.28 to -3.11) | -12.8 (-16.6 to -10.9) | -1.87 (-2.39 to -1.48) |
| Udaipur | 55.0 (50.6-59.4) | 61.9 (55.7-68.5) | 43.6 (42.3-44.8) | -21.7 (-22.9 to -20.6) | -1.36 (-1.44 to -1.28) | -30.3 (-31.8 to -29.5) | -4.86 (-5.12 to -4.68) |
| **Chhattisgarh** | **59.0 (53.6-64.1)** | **42.6 (40.8-45.2)** | **33.4 (32.3-34.9)** | **-44.3 (-44.9 to -43.8)** | **-3.29 (-3.34 to -3.23)** | **-22.5 (-23.5 to -21.8)** | **-3.48 (-4.29 to -2.79)** |
| Balod | 70.4 (58.9-82.3) | 45.8 (39.8-51.9) | 32.9 (31.7-34.4) | -54.0 (-55.3 to -53.2) | -4.36 (-4.50 to -4.25) | -28.9 (-30.5 to -27.9) | -4.60 (-4.88 to -4.37) |
| Baloda Bazar | 46.8 (41.5-52.6) | 38.5 (32.5-44.4) | 32.6 (31.8-33.5) | -31.2 (-32.5 to -30.1) | -2.10 (-2.20 to -2.00) | -15.7 (-18.0 to -14.2) | -2.29 (-2.62 to -2.00) |
| Balrampur | 63.9 (55.0-72.3) | 44.7 (37.5-51.7) | 32.1 (31.3-33.1) | -50.4 (-51.8 to -49.8) | -3.95 (-4.09 to -3.87) | -28.6 (-30.6 to -27.7) | -4.55 (-4.89 to -4.33) |
| Bastar | 69.3 (59.8-78.2) | 47.3 (38.4-56.1) | 40.4 (39.3-41.8) | -42.4 (-43.8 to -41.3) | -3.11 (-3.23 to -2.99) | -14.7 (-17.8 to -13.1) | -2.15 (-2.59 to -1.82) |
| Bemetara | 61.0 (53.8-68.8) | 42.9 (37.1-48.7) | 34.8 (33.9-35.6) | -43.6 (-44.8 to -42.9) | -3.23 (-3.33 to -3.14) | -19.3 (-21.1 to -17.9) | -2.88 (-3.16 to -2.62) |
| Bijapur | 60.6 (50.2-71.6) | 48.9 (39.4-58.2) | 39.8 (38.3-41.0) | -34.8 (-37.4 to -33.3) | -2.42 (-2.63 to -2.27) | -18.9 (-21.6 to -17.6) | -2.85 (-3.23 to -2.56) |
| Bilaspur | 44.0 (39.8-48.6) | 40.1 (34.3-45.8) | 32.9 (32.2-33.6) | -25.9 (-27.0 to -25.0) | -1.68 (-1.76 to -1.60) | -18.4 (-20.5 to -17.2) | -2.74 (-3.06 to -2.49) |
| Dantewada | 63.6 (54.0-73.1) | 47.8 (39.3-56.4) | 40.1 (39.1-41.0) | -37.6 (-39.3 to -36.7) | -2.66 (-2.80 to -2.57) | -16.5 (-19.4 to -15.0) | -2.44 (-2.87 to -2.14) |
| Dhamtari | 63.4 (52.9-73.7) | 39.0 (32.2-45.3) | 32.9 (32.0-33.9) | -48.7 (-50.5 to -47.7) | -3.77 (-3.94 to -3.63) | -16.1 (-18.5 to -14.6) | -2.36 (-2.72 to -2.07) |
| Durg | 64.3 (54.1-75.0) | 40.6 (34.4-47.2) | 30.8 (29.5-31.8) | -52.7 (-54.3 to -51.9) | -4.22 (-4.38 to -4.09) | -24.7 (-26.7 to -23.6) | -3.84 (-4.16 to -3.59) |
| Gariaband | 65.4 (58.0-72.3) | 40.3 (34.5-46.4) | 35.7 (34.8-36.7) | -46.3 (-47.3 to -45.6) | -3.49 (-3.59 to -3.42) | -11.9 (-14.2 to -10.4) | -1.67 (-2.01 to -1.41) |
| Janjgir-Champa | 42.7 (37.6-48.3) | 39.3 (32.0-45.7) | 31.9 (30.9-32.9) | -25.9 (-27.4 to -24.7) | -1.68 (-1.79 to -1.58) | -19.0 (-22.0 to -17.7) | -2.84 (-3.30 to -2.58) |
| Jashpur | 64.7 (55.6-73.5) | 46.8 (40.0-54.0) | 30.6 (29.4-31.7) | -53.4 (-54.5 to -52.7) | -4.29 (-4.41 to -4.20) | -35.2 (-36.8 to -34.0) | -5.83 (-6.13 to -5.56) |
| Kabirdham | 63.5 (56.0-72.0) | 45.7 (38.2-53.1) | 37.2 (35.8-38.5) | -42.1 (-43.5 to -41.5) | -3.08 (-3.21 to -3.01) | -19.1 (-20.9 to -17.6) | -2.85 (-3.12 to -2.56) |
| Kondagaon | 68.4 (58.3-79.1) | 45.4 (37.3-52.6) | 40.6 (39.5-41.8) | -41.2 (-42.7 to -40.3) | -3.00 (-3.12 to -2.89) | -10.8 (-14.2 to -9.4) | -1.52 (-2.00 to -1.24) |
| Korba | 48.6 (42.0-56.4) | 42.1 (35.3-48.7) | 30.7 (29.7-31.5) | -37.5 (-38.9 to -36.3) | -2.65 (-2.77 to -2.53) | -27.7 (-29.7 to -26.8) | -4.39 (-4.72 to -4.17) |
| Korea | 64.4 (57.5-71.5) | 44.5 (37.9-51.1) | 30.4 (29.8-31.1) | -53.6 (-54.5 to -53.0) | -4.31 (-4.40 to -4.23) | -32.2 (-33.8 to -31.0) | -5.24 (-5.52 to -4.96) |
| Mahasamund | 57.8 (52.9-62.7) | 39.0 (32.9-44.2) | 34.3 (33.4-35.0) | -41.5 (-42.3 to -40.8) | -3.01 (-3.09 to -2.94) | -12.6 (-15.2 to -11.3) | -1.78 (-2.16 to -1.54) |
| Mungeli | 50.6 (45.0-56.4) | 42.1 (35.8-48.7) | 35.1 (34.2-36.0) | -31.4 (-32.6 to -30.6) | -2.12 (-2.21 to -2.04) | -17.1 (-19.0 to -15.6) | -2.52 (-2.80 to -2.24) |
| Narayanpur | 69.3 (58.4-80.5) | 47.4 (38.5-55.7) | 41.1 (39.8-42.5) | -41.3 (-43.1 to -40.4) | -3.01 (-3.16 to -2.90) | -13.4 (-16.5 to -12.2) | -1.94 (-2.38 to -1.69) |
| North Bastar Kanker | 68.2 (57.6-79.0) | 44.1 (37.5-51.1) | 36.0 (34.9-37.1) | -47.9 (-49.4 to -46.9) | -3.68 (-3.81 to -3.55) | -19.0 (-21.5 to -17.3) | -2.83 (-3.22 to -2.50) |
| Raigarh | 56.2 (49.2-62.7) | 43.0 (36.6-49.3) | 32.2 (31.3-33.1) | -43.6 (-44.9 to -42.9) | -3.23 (-3.35 to -3.14) | -25.7 (-27.6 to -24.9) | -4.02 (-4.33 to -3.82) |
| Raipur | 58.6 (48.1-69.2) | 38.2 (31.0-45.3) | 31.0 (29.8-32.0) | -47.7 (-49.3 to -46.8) | -3.66 (-3.81 to -3.54) | -19.2 (-21.7 to -17.7) | -2.89 (-3.26 to -2.58) |
| Rajnandgaon | 70.7 (62.6-79.6) | 46.6 (41.9-52.1) | 33.3 (32.4-34.3) | -53.7 (-54.5 to -53.1) | -4.32 (-4.41 to -4.24) | -29.2 (-30.7 to -28.3) | -4.65 (-4.91 to -4.46) |
| Sukma | 61.4 (53.3-69.1) | 48.1 (39.8-56.4) | 40.3 (39.0-41.5) | -35.2 (-36.6 to -34.4) | -2.44 (-2.55 to -2.36) | -16.6 (-19.3 to -15.4) | -2.46 (-2.84 to -2.19) |
| Surajpur | 68.3 (60.9-76.2) | 45.3 (38.9-51.7) | 30.8 (30.1-31.5) | -55.8 (-56.6 to -55.1) | -4.57 (-4.66 to -4.48) | -32.6 (-34.4 to -31.5) | -5.33 (-5.64 to -5.07) |
| Surguja | 65.3 (54.9-75.8) | 45.5 (37.7-53.7) | 30.2 (28.9-31.6) | -54.4 (-55.7 to -53.7) | -4.41 (-4.55 to -4.31) | -34.1 (-36.4 to -33.2) | -5.63 (-6.05 to -5.40) |
| **Odisha** | **58.0 (54.4-61.3)** | **39.2 (37.6-41.3)** | **31.9 (30.7-33.1)** | **-46.0 (-46.2 to -45.8)** | **-3.46 (-3.48 to -3.43)** | **-19.5 (-20.3 to -18.9)** | **-2.94 (-3.68 to -2.32)** |
| Angul | 54.9 (50.2-59.6) | 40.2 (35.2-45.9) | 29.7 (28.9-30.8) | -46.7 (-47.7 to -46.0) | -3.54 (-3.63 to -3.45) | -26.7 (-28.6 to -25.2) | -4.19 (-4.52 to -3.88) |
| Balangir | 66.6 (62.1-71.3) | 41.6 (37.2-46.3) | 40.4 (39.5-41.6) | -40.2 (-41.1 to -39.6) | -2.89 (-2.96 to -2.83) | -3.6 (-5.5 to -1.9) | -0.40 (-0.66 to -0.14) |
| Balasore | 56.4 (52.0-61.1) | 41.1 (36.6-45.8) | 33.1 (31.9-34.2) | -42.2 (-43.3 to -41.5) | -3.09 (-3.19 to -3.01) | -20.3 (-21.9 to -19.3) | -3.05 (-3.30 to -2.84) |
| Bargarh | 62.5 (57.9-67.0) | 43.7 (38.5-48.9) | 38.6 (37.6-39.7) | -39.1 (-39.9 to -38.5) | -2.79 (-2.85 to -2.73) | -12.2 (-14.1 to -11.0) | -1.72 (-1.99 to -1.50) |
| Bhadrak | 56.7 (51.6-62.0) | 37.8 (32.9-43.0) | 27.6 (26.4-28.8) | -52.3 (-53.2 to -51.7) | -4.15 (-4.24 to -4.07) | -27.7 (-29.4 to -26.4) | -4.39 (-4.66 to -4.10) |
| Boudh | 62.8 (58.1-67.3) | 44.2 (39.1-49.6) | 37.7 (36.6-38.9) | -40.9 (-41.9 to -40.3) | -2.96 (-3.05 to -2.90) | -15.3 (-17.2 to -14.1) | -2.22 (-2.49 to -1.98) |
| Cuttack | 54.5 (49.5-59.0) | 29.2 (24.7-33.6) | 19.8 (19.2-20.6) | -64.6 (-65.3 to -64.2) | -5.78 (-5.88 to -5.71) | -32.7 (-34.2 to -31.2) | -5.35 (-5.60 to -5.00) |
| Deogarh | 58.0 (52.9-63.0) | 46.4 (40.2-52.2) | 31.8 (30.5-32.8) | -46.1 (-47.1 to -45.4) | -3.48 (-3.57 to -3.40) | -32.3 (-34.0 to -31.5) | -5.25 (-5.56 to -5.07) |
| Dhenkanal | 55.8 (50.9-60.5) | 37.7 (32.2-43.0) | 25.6 (24.6-26.5) | -55.0 (-55.8 to -54.4) | -4.47 (-4.56 to -4.39) | -32.8 (-34.5 to -31.6) | -5.35 (-5.65 to -5.09) |
| Gajapati | 56.1 (49.9-62.3) | 37.4 (30.9-44.9) | 32.8 (31.5-34.1) | -42.3 (-43.7 to -41.1) | -3.10 (-3.22 to -2.97) | -12.4 (-15.0 to -10.2) | -1.78 (-2.13 to -1.38) |
| Ganjam | 57.8 (52.6-62.9) | 33.6 (28.7-38.4) | 29.5 (28.3-31.0) | -49.8 (-50.6 to -49.0) | -3.87 (-3.95 to -3.78) | -12.6 (-15.3 to -10.5) | -1.79 (-2.19 to -1.41) |
| Jagatsinghapur | 55.4 (49.4-61.8) | 25.1 (20.6-30.3) | 16.9 (16.0-17.8) | -70.3 (-71.2 to -69.8) | -6.73 (-6.87 to -6.61) | -32.9 (-35.2 to -31.3) | -5.42 (-5.81 to -5.02) |
| Jajapur | 56.4 (51.3-61.3) | 33.4 (28.8-39.1) | 23.5 (22.5-24.4) | -59.3 (-60.1 to -58.5) | -5.03 (-5.12 to -4.91) | -30.4 (-32.4 to -29.4) | -4.89 (-5.24 to -4.65) |
| Jharsuguda | 56.5 (51.1-62.0) | 44.9 (38.7-51.4) | 33.1 (32.1-34.5) | -42.2 (-43.5 to -41.5) | -3.09 (-3.20 to -3.01) | -26.9 (-28.8 to -26.0) | -4.22 (-4.55 to -4.02) |
| Kalahandi | 61.6 (56.5-66.5) | 42.7 (37.1-47.9) | 41.7 (40.6-42.8) | -33.2 (-34.3 to -32.2) | -2.27 (-2.36 to -2.17) | -3.1 (-5.2 to -2.0) | -0.33 (-0.61 to -0.14) |
| Kandhamal | 58.2 (53.8-63.1) | 37.1 (31.6-42.6) | 36.8 (35.4-37.8) | -37.7 (-38.7 to -36.9) | -2.66 (-2.75 to -2.58) | -1.3 (-4.5 to 0.2) | -0.09 (-0.51 to 0.17) |
| Kendrapara | 55.7 (49.7-61.4) | 29.2 (24.4-34.6) | 20.0 (19.0-20.9) | -65.1 (-65.7 to -64.5) | -5.86 (-5.95 to -5.76) | -32.0 (-34.0 to -30.4) | -5.23 (-5.57 to -4.84) |
| Kendujhar | 58.4 (54.5-62.3) | 49.8 (44.6-55.0) | 40.3 (39.4-41.3) | -31.9 (-32.9 to -31.3) | -2.16 (-2.23 to -2.10) | -19.8 (-20.9 to -18.7) | -2.95 (-3.12 to -2.74) |
| Khordha | 54.7 (48.6-60.9) | 27.9 (22.7-33.6) | 18.9 (17.9-20.3) | -66.3 (-67.2 to -65.7) | -6.05 (-6.18 to -5.94) | -32.6 (-35.0 to -31.4) | -5.36 (-5.76 to -5.05) |
| Koraput | 59.3 (54.4-64.5) | 46.6 (39.9-53.2) | 43.7 (42.5-44.8) | -27.2 (-28.3 to -26.2) | -1.78 (-1.86 to -1.69) | -6.7 (-9.1 to -5.7) | -0.87 (-1.20 to -0.68) |
| Malkangiri | 61.3 (55.7-67.7) | 48.0 (38.9-56.7) | 45.8 (44.4-47.0) | -26.1 (-27.4 to -25.1) | -1.70 (-1.79 to -1.61) | -4.7 (-8.2 to -2.6) | -0.60 (-1.06 to -0.23) |
| Mayurbhanj | 57.5 (53.6-61.5) | 48.4 (43.7-53.0) | 41.4 (40.4-42.2) | -29.0 (-29.8 to -28.3) | -1.92 (-1.98 to -1.86) | -15.3 (-16.7 to -14.4) | -2.21 (-2.41 to -2.03) |
| Nabarangpur | 62.2 (55.7-68.3) | 47.6 (40.3-55.2) | 44.5 (43.8-45.5) | -29.2 (-30.6 to -28.2) | -1.94 (-2.04 to -1.85) | -6.8 (-9.4 to -5.4) | -0.89 (-1.24 to -0.64) |
| Nayagarh | 52.4 (47.2-57.3) | 31.0 (26.1-36.5) | 24.4 (23.5-25.4) | -54.2 (-55.3 to -53.5) | -4.39 (-4.50 to -4.28) | -21.5 (-23.9 to -19.8) | -3.28 (-3.64 to -2.93) |
| Nuapada | 63.6 (58.2-68.9) | 39.7 (33.2-46.1) | 39.6 (38.3-40.9) | -38.6 (-39.7 to -37.8) | -2.74 (-2.83 to -2.66) | -0.5 (-3.8 to 1.7) | 0.02 (-0.41 to 0.38) |
| Puri | 54.8 (49.2-60.7) | 25.6 (20.9-30.7) | 17.0 (16.1-17.9) | -70.0 (-70.6 to -69.3) | -6.67 (-6.76 to -6.53) | -34.0 (-36.8 to -32.6) | -5.65 (-6.12 to -5.28) |
| Rayagada | 56.9 (52.1-61.8) | 43.9 (37.3-50.3) | 40.2 (39.0-41.2) | -30.3 (-31.3 to -29.4) | -2.03 (-2.10 to -1.95) | -8.9 (-12.0 to -7.6) | -1.21 (-1.65 to -0.97) |
| Sambalpur | 60.3 (55.6-65.3) | 47.5 (42.0-53.2) | 34.3 (33.1-35.4) | -44.0 (-44.8 to -43.4) | -3.26 (-3.33 to -3.19) | -28.5 (-30.1 to -27.5) | -4.53 (-4.80 to -4.31) |
| Sonepur | 66.6 (61.1-72.2) | 48.5 (42.4-54.2) | 40.5 (39.2-41.8) | -40.1 (-41.1 to -39.6) | -2.88 (-2.97 to -2.83) | -17.2 (-19.2 to -15.9) | -2.53 (-2.83 to -2.28) |
| Sundargarh | 59.4 (54.4-64.1) | 50.8 (45.5-56.4) | 37.0 (36.1-38.0) | -38.5 (-39.4 to -37.7) | -2.73 (-2.81 to -2.66) | -27.9 (-29.4 to -27.0) | -4.40 (-4.66 to -4.21) |
| **Assam** | **42.9 (38.6-49.1)** | **32.2 (31.1-33.5)** | **26.9 (25.9-27.8)** | **-38.4 (-38.9 to -38.0)** | **-2.72 (-2.76 to -2.68)** | **-17.4 (-19.3 to -16.3)** | **-2.61 (-3.20 to -2.08)** |
| Baksa | 43.9 (36.9-52.0) | 29.7 (23.5-36.8) | 22.8 (21.7-23.6) | -48.8 (-50.1 to -47.7) | -3.78 (-3.89 to -3.63) | -23.3 (-26.6 to -21.4) | -3.64 (-4.15 to -3.21) |
| Barpeta | 52.6 (47.2-58.0) | 33.3 (27.8-38.9) | 27.6 (26.9-28.3) | -48.4 (-49.3 to -47.6) | -3.72 (-3.81 to -3.63) | -17.4 (-20.2 to -15.3) | -2.58 (-3.01 to -2.18) |
| Biswanath | 36.7 (30.5-43.7) | 33.5 (24.6-42.8) | 23.8 (22.8-25.0) | -35.5 (-37.1 to -34.2) | -2.49 (-2.60 to -2.34) | -28.6 (-32.4 to -27.3) | -4.63 (-5.25 to -4.27) |
| Bongaigaon | 55.6 (48.3-63.6) | 33.4 (26.6-40.5) | 28.9 (27.4-30.0) | -48.9 (-49.9 to -47.8) | -3.78 (-3.88 to -3.64) | -13.5 (-17.2 to -11.5) | -1.98 (-2.48 to -1.57) |
| Cachar | 42.2 (35.7-49.4) | 33.0 (26.4-39.9) | 27.0 (25.7-28.3) | -36.4 (-38.4 to -35.5) | -2.57 (-2.72 to -2.45) | -18.0 (-21.2 to -16.7) | -2.71 (-3.17 to -2.40) |
| Charaideo | 28.6 (24.3-33.3) | 20.7 (14.6-28.6) | 23.2 (22.1-24.6) | -19.4 (-21.5 to -17.6) | -1.21 (-1.34 to -1.06) | 14.2 (7.2 to 16.9) | 1.85 (1.14 to 2.38) |
| Chirang | 51.9 (45.4-59.0) | 30.9 (24.1-38.3) | 24.5 (23.6-25.6) | -53.5 (-54.5 to -52.4) | -4.30 (-4.40 to -4.16) | -20.3 (-24.0 to -18.7) | -3.13 (-3.67 to -2.75) |
| Darrang | 44.8 (39.8-50.4) | 36.1 (29.8-42.0) | 28.2 (27.3-28.9) | -38.0 (-39.4 to -37.0) | -2.69 (-2.81 to -2.59) | -22.4 (-25.2 to -21.2) | -3.45 (-3.89 to -3.16) |
| Dhemaji | 25.7 (22.0-29.9) | 22.1 (15.6-30.4) | 20.9 (20.2-21.5) | -19.3 (-21.1 to -17.7) | -1.20 (-1.31 to -1.07) | -3.8 (-10.3 to -0.5) | -0.60 (-1.38 to 0.07) |
| Dhubri | 60.9 (55.7-68.1) | 33.9 (28.4-39.4) | 30.9 (29.4-32.0) | -50.1 (-51.1 to -49.2) | -3.91 (-4.00 to -3.79) | -9.1 (-11.7 to -7.0) | -1.25 (-1.60 to -0.88) |
| Dibrugarh | 27.8 (23.6-32.5) | 22.7 (16.0-31.5) | 23.7 (22.7-24.7) | -15.1 (-17.2 to -13.3) | -0.91 (-1.04 to -0.77) | 6.7 (-0.5 to 10.3) | 0.86 (0.07 to 1.54) |
| Dima Hasao | 32.4 (28.5-36.7) | 29.6 (23.2-37.5) | 21.6 (21.1-22.1) | -34.1 (-35.5 to -32.9) | -2.35 (-2.46 to -2.23) | -27.0 (-29.7 to -25.4) | -4.30 (-4.72 to -3.92) |
| Goalpara | 56.4 (50.7-62.3) | 34.6 (28.6-41.1) | 29.8 (28.9-30.7) | -48.1 (-48.9 to -47.2) | -3.69 (-3.76 to -3.58) | -14.3 (-17.2 to -12.9) | -2.07 (-2.49 to -1.80) |
| Golaghat | 34.5 (30.6-39.2) | 28.0 (20.7-36.0) | 21.8 (21.4-22.3) | -37.6 (-38.8 to -36.4) | -2.66 (-2.76 to -2.54) | -21.5 (-25.6 to -20.3) | -3.35 (-3.95 to -3.01) |
| Hailakandi | 47.1 (41.6-52.5) | 32.7 (27.6-38.2) | 28.1 (27.3-29.1) | -41.1 (-42.4 to -40.3) | -2.98 (-3.09 to -2.89) | -14.4 (-17.0 to -13.1) | -2.09 (-2.45 to -1.83) |
| Hojai | 42.9 (36.6-50.0) | 41.1 (32.5-49.9) | 31.4 (30.4-32.8) | -27.5 (-29.4 to -26.3) | -1.82 (-1.95 to -1.70) | -23.7 (-26.8 to -22.9) | -3.71 (-4.17 to -3.46) |
| Jorhat | 31.6 (27.2-36.7) | 24.9 (17.8-32.7) | 23.0 (22.1-24.0) | -27.6 (-29.5 to -26.1) | -1.83 (-1.96 to -1.69) | -6.4 (-11.7 to -2.9) | -0.94 (-1.61 to -0.28) |
| Kamrup | 44.9 (40.0-50.0) | 33.5 (28.3-38.9) | 25.8 (25.1-26.4) | -43.4 (-44.5 to -42.4) | -3.20 (-3.30 to -3.09) | -23.5 (-25.9 to -22.2) | -3.62 (-4.01 to -3.35) |
| Kamrup Metropolitan | 38.0 (31.1-45.1) | 34.2 (27.8-41.0) | 25.3 (24.2-26.5) | -33.9 (-37.1 to -32.4) | -2.36 (-2.60 to -2.20) | -26.3 (-29.3 to -24.0) | -4.17 (-4.64 to -3.67) |
| Karbi Anglong | 42.2 (36.2-48.7) | 35.2 (26.3-44.3) | 25.4 (24.4-26.4) | -40.5 (-42.3 to -39.4) | -2.93 (-3.09 to -2.81) | -27.6 (-31.1 to -25.7) | -4.43 (-4.99 to -3.97) |
| Karimganj | 55.9 (49.8-61.3) | 35.0 (30.6-39.7) | 33.6 (32.3-34.8) | -40.6 (-41.9 to -39.7) | -2.94 (-3.05 to -2.84) | -4.5 (-6.8 to -3.2) | -0.55 (-0.85 to -0.32) |
| Kokrajhar | 57.2 (50.0-65.7) | 32.9 (26.0-40.6) | 28.7 (27.5-29.7) | -50.6 (-51.6 to -49.8) | -3.97 (-4.06 to -3.86) | -12.7 (-16.3 to -11.0) | -1.85 (-2.35 to -1.49) |
| Lakhimpur | 29.0 (24.8-33.5) | 24.9 (17.9-32.8) | 22.1 (21.6-22.8) | -24.3 (-26.3 to -22.8) | -1.57 (-1.70 to -1.44) | -10.0 (-15.7 to -8.9) | -1.50 (-2.24 to -1.17) |
| Majuli | 32.9 (28.0-38.6) | 25.3 (18.2-33.4) | 23.5 (22.4-24.8) | -28.9 (-31.1 to -27.3) | -1.93 (-2.09 to -1.78) | -5.5 (-11.1 to -3.5) | -0.82 (-1.51 to -0.35) |
| Morigaon | 44.2 (38.9-50.6) | 40.2 (32.5-47.1) | 31.8 (30.5-32.9) | -28.8 (-30.0 to -27.5) | -1.91 (-1.99 to -1.80) | -21.2 (-23.9 to -19.8) | -3.23 (-3.65 to -2.92) |
| Nagaon | 44.7 (38.6-51.8) | 41.1 (32.3-50.3) | 32.2 (31.1-33.3) | -28.6 (-30.1 to -27.4) | -1.90 (-2.00 to -1.79) | -21.7 (-24.7 to -20.7) | -3.34 (-3.79 to -3.08) |
| Nalbari | 47.2 (40.6-54.6) | 32.1 (26.2-38.2) | 25.4 (24.4-26.5) | -47.0 (-48.4 to -45.8) | -3.58 (-3.71 to -3.43) | -21.1 (-24.0 to -19.4) | -3.22 (-3.67 to -2.86) |
| Sivasagar | 28.8 (24.9-33.2) | 22.2 (15.8-30.1) | 22.6 (21.7-23.6) | -22.1 (-24.0 to -20.3) | -1.41 (-1.53 to -1.26) | 3.6 (-3.2 to 6.6) | 0.46 (-0.31 to 1.06) |
| Sonitpur | 42.2 (36.1-48.8) | 38.6 (30.1-47.4) | 28.4 (27.2-29.4) | -33.4 (-35.2 to -32.3) | -2.30 (-2.43 to -2.18) | -26.5 (-30.0 to -24.9) | -4.21 (-4.77 to -3.83) |
| South Salmara Mancachar | 60.8 (53.6-68.1) | 32.8 (28.2-38.0) | 29.9 (28.6-31.1) | -51.7 (-52.9 to -51.0) | -4.09 (-4.22 to -4.00) | -9.2 (-11.9 to -7.8) | -1.26 (-1.64 to -1.00) |
| Tinsukia | 30.6 (26.3-35.2) | 27.2 (19.4-36.2) | 28.1 (26.9-29.1) | -8.6 (-11.4 to -7.1) | -0.49 (-0.64 to -0.37) | 4.9 (-1.3 to 8.6) | 0.65 (-0.04 to 1.31) |
| Udalguri | 41.8 (36.2-47.9) | 32.4 (26.2-38.8) | 24.0 (23.1-25.0) | -43.1 (-44.9 to -42.3) | -3.19 (-3.34 to -3.08) | -26.0 (-29.2 to -24.1) | -4.11 (-4.62 to -3.69) |
| West Karbi Anglong | 38.2 (33.4-43.2) | 39.0 (31.0-47.4) | 29.7 (28.6-30.9) | -22.8 (-24.6 to -21.7) | -1.45 (-1.57 to -1.36) | -23.9 (-26.7 to -22.3) | -3.72 (-4.16 to -3.36) |
| **Andhra Pradesh** | **49.1 (45.4-53.3)** | **32.9 (32.0-33.8)** | **27.2 (26.3-28.2)** | **-45.5 (-45.9 to -45.2)** | **-3.41 (-3.45 to -3.37)** | **-18.0 (-19.3 to -16.9)** | **-2.69 (-3.20 to -2.21)** |
| Anantapur | 48.8 (43.8-53.9) | 39.3 (35.1-44.1) | 31.7 (30.7-32.5) | -36.0 (-37.0 to -35.3) | -2.51 (-2.59 to -2.44) | -20.1 (-21.6 to -18.6) | -3.01 (-3.24 to -2.73) |
| Chittoor | 40.5 (36.5-44.5) | 28.0 (23.2-32.6) | 25.4 (24.4-26.2) | -38.1 (-39.3 to -37.4) | -2.71 (-2.80 to -2.63) | -9.6 (-12.3 to -8.0) | -1.34 (-1.69 to -1.04) |
| East Godavari | 54.7 (48.7-61.2) | 33.5 (25.8-41.6) | 26.1 (25.1-27.4) | -53.1 (-54.1 to -52.5) | -4.25 (-4.36 to -4.16) | -21.9 (-25.5 to -20.4) | -3.40 (-3.93 to -3.03) |
| Guntur | 52.7 (46.3-59.8) | 30.5 (25.8-35.2) | 26.1 (24.7-27.2) | -51.4 (-52.5 to -50.4) | -4.05 (-4.16 to -3.93) | -14.9 (-17.2 to -13.1) | -2.17 (-2.50 to -1.83) |
| Krishna | 48.8 (43.4-54.4) | 31.7 (27.2-36.6) | 24.7 (23.7-25.8) | -50.3 (-51.5 to -49.4) | -3.93 (-4.05 to -3.82) | -22.5 (-24.7 to -20.9) | -3.45 (-3.79 to -3.12) |
| Kurnool | 47.8 (43.8-52.6) | 41.1 (35.8-46.3) | 32.9 (31.9-34.0) | -31.9 (-32.7 to -30.8) | -2.16 (-2.22 to -2.06) | -20.5 (-22.3 to -19.4) | -3.09 (-3.37 to -2.86) |
| Prakasam | 48.5 (42.5-54.6) | 26.2 (20.4-32.8) | 24.6 (23.3-25.9) | -50.1 (-51.4 to -49.3) | -3.91 (-4.04 to -3.81) | -6.0 (-10.1 to -4.0) | -0.83 (-1.36 to -0.43) |
| Sri Potti Sriramulu Nellore | 45.3 (39.9-51.5) | 27.7 (21.2-35.5) | 24.4 (23.1-25.7) | -46.8 (-48.1 to -45.6) | -3.55 (-3.67 to -3.41) | -10.9 (-15.3 to -8.6) | -1.61 (-2.18 to -1.12) |
| Srikakulam | 55.0 (49.5-61.2) | 33.5 (27.9-40.1) | 24.4 (23.4-25.7) | -56.5 (-57.6 to -56.0) | -4.67 (-4.79 to -4.59) | -27.5 (-29.7 to -25.7) | -4.37 (-4.72 to -3.98) |
| Visakhapatnam | 51.6 (45.9-58.1) | 35.3 (30.6-41.1) | 28.9 (27.7-30.3) | -44.9 (-46.1 to -43.8) | -3.36 (-3.46 to -3.23) | -18.6 (-20.7 to -17.0) | -2.78 (-3.08 to -2.47) |
| Vizianagaram | 53.5 (47.9-59.2) | 36.5 (31.7-41.7) | 29.1 (28.0-30.0) | -46.4 (-47.6 to -45.8) | -3.51 (-3.62 to -3.43) | -20.9 (-22.5 to -19.4) | -3.15 (-3.40 to -2.86) |
| West Godavari | 46.5 (41.2-52.3) | 32.0 (25.9-39.4) | 25.9 (24.7-27.3) | -45.0 (-46.5 to -44.0) | -3.37 (-3.51 to -3.25) | -19.1 (-22.0 to -17.5) | -2.90 (-3.31 to -2.54) |
| YSR | 43.8 (38.1-49.8) | 30.8 (25.4-36.6) | 29.1 (27.6-30.1) | -34.4 (-35.9 to -33.0) | -2.38 (-2.50 to -2.24) | -5.7 (-8.8 to -3.6) | -0.75 (-1.16 to -0.37) |
| **West Bengal** | **50.9 (47.1-54.9)** | **36.4 (35.4-37.5)** | **30.2 (29.2-31.3)** | **-41.6 (-41.9 to -41.2)** | **-3.02 (-3.05 to -2.98)** | **-17.9 (-18.8 to -17.2)** | **-2.67 (-3.18 to -2.16)** |
| Alipurduar | 47.4 (40.0-55.0) | 39.7 (33.4-45.1) | 27.4 (26.1-28.6) | -42.9 (-44.1 to -41.5) | -3.17 (-3.27 to -3.01) | -31.6 (-33.8 to -30.5) | -5.14 (-5.52 to -4.87) |
| Bankura | 53.0 (49.0-57.1) | 46.7 (40.3-53.3) | 39.9 (38.7-41.0) | -25.7 (-26.9 to -24.9) | -1.66 (-1.75 to -1.59) | -15.3 (-17.1 to -13.7) | -2.21 (-2.48 to -1.92) |
| Birbhum | 55.6 (51.5-59.7) | 46.8 (42.4-51.4) | 37.3 (36.3-38.1) | -33.7 (-34.6 to -33.1) | -2.31 (-2.38 to -2.25) | -21.1 (-22.1 to -19.9) | -3.18 (-3.32 to -2.96) |
| Cooch Behar | 51.9 (46.9-57.1) | 41.2 (37.2-45.7) | 30.7 (29.3-31.9) | -41.6 (-42.8 to -40.5) | -3.03 (-3.14 to -2.92) | -26.2 (-28.0 to -25.4) | -4.10 (-4.39 to -3.91) |
| Dakshin Dinajpur | 54.2 (48.4-60.2) | 36.8 (33.0-40.7) | 33.4 (32.2-34.9) | -39.1 (-40.2 to -38.1) | -2.79 (-2.89 to -2.69) | -9.9 (-11.8 to -8.3) | -1.35 (-1.62 to -1.08) |
| Darjeeling | 44.1 (39.3-48.8) | 36.2 (31.9-40.8) | 27.8 (26.6-29.3) | -37.7 (-39.2 to -36.6) | -2.67 (-2.79 to -2.56) | -23.7 (-25.7 to -22.4) | -3.66 (-3.96 to -3.37) |
| Hooghly | 49.1 (44.6-53.7) | 30.1 (26.1-34.1) | 26.4 (25.4-27.5) | -47.1 (-48.0 to -46.3) | -3.58 (-3.67 to -3.49) | -12.9 (-14.7 to -11.2) | -1.82 (-2.09 to -1.52) |
| Howrah | 48.7 (43.6-54.1) | 27.3 (22.9-32.3) | 24.4 (23.2-25.7) | -50.7 (-52.0 to -49.9) | -3.98 (-4.11 to -3.87) | -10.9 (-13.6 to -9.9) | -1.55 (-1.90 to -1.32) |
| Jalpaiguri | 47.5 (41.0-54.3) | 39.2 (33.5-44.3) | 30.7 (29.5-32.2) | -36.0 (-37.7 to -34.5) | -2.52 (-2.65 to -2.37) | -22.3 (-24.4 to -20.7) | -3.40 (-3.74 to -3.09) |
| Jhargram | 55.6 (51.4-60.4) | 50.2 (43.9-56.6) | 39.4 (38.3-40.7) | -30.0 (-30.9 to -29.1) | -2.00 (-2.07 to -1.92) | -22.0 (-24.0 to -21.4) | -3.34 (-3.67 to -3.21) |
| Kalimpong | 32.1 (27.5-36.7) | 24.9 (20.0-30.3) | 18.8 (17.9-19.9) | -42.0 (-43.6 to -41.3) | -3.08 (-3.21 to -2.99) | -24.6 (-27.4 to -23.8) | -3.87 (-4.29 to -3.63) |
| Kolkata | 46.2 (39.0-53.7) | 27.0 (21.8-32.7) | 22.9 (21.8-24.6) | -51.2 (-52.7 to -49.6) | -4.05 (-4.19 to -3.84) | -15.5 (-19.1 to -13.3) | -2.31 (-2.82 to -1.85) |
| Maldah | 55.7 (49.2-62.0) | 38.8 (34.2-43.6) | 33.4 (32.4-34.3) | -40.9 (-42.3 to -39.9) | -2.96 (-3.09 to -2.86) | -14.7 (-16.6 to -13.5) | -2.11 (-2.40 to -1.89) |
| Murshidabad | 53.3 (48.9-57.5) | 41.0 (36.8-45.5) | 34.4 (33.1-35.4) | -36.4 (-37.4 to -35.6) | -2.55 (-2.63 to -2.47) | -16.9 (-18.9 to -15.6) | -2.47 (-2.78 to -2.22) |
| Nadia | 46.9 (43.1-50.7) | 31.3 (28.1-34.3) | 24.4 (23.3-25.8) | -48.8 (-49.6 to -47.9) | -3.76 (-3.84 to -3.66) | -22.8 (-24.1 to -21.4) | -3.48 (-3.68 to -3.21) |
| North 24 Parganas | 45.2 (40.8-50.1) | 29.0 (24.8-33.2) | 23.3 (22.3-24.6) | -49.4 (-50.5 to -48.2) | -3.83 (-3.94 to -3.69) | -20.4 (-22.8 to -18.7) | -3.08 (-3.45 to -2.74) |
| Paschim Burdwan | 50.5 (45.0-56.8) | 44.5 (38.4-51.1) | 35.5 (34.0-36.7) | -30.5 (-32.2 to -29.3) | -2.05 (-2.18 to -1.94) | -20.8 (-22.5 to -19.3) | -3.15 (-3.41 to -2.85) |
| Pashchim Medinipur | 54.9 (51.1-58.9) | 41.1 (34.9-47.4) | 35.4 (34.2-36.7) | -36.3 (-37.1 to -35.5) | -2.54 (-2.60 to -2.46) | -14.2 (-16.6 to -12.7) | -2.04 (-2.40 to -1.76) |
| Purba Burdwan | 51.6 (47.0-56.6) | 35.1 (30.7-39.6) | 30.9 (29.6-32.1) | -41.0 (-42.0 to -40.2) | -2.97 (-3.05 to -2.88) | -12.7 (-14.7 to -11.1) | -1.79 (-2.08 to -1.51) |
| Purba Medinipur | 58.4 (53.7-63.3) | 35.8 (29.8-42.3) | 29.9 (28.7-31.2) | -49.6 (-50.5 to -48.9) | -3.85 (-3.94 to -3.76) | -16.7 (-19.1 to -15.2) | -2.47 (-2.81 to -2.17) |
| Puruliya | 53.1 (46.9-59.2) | 55.3 (50.1-60.7) | 43.4 (42.3-44.2) | -18.9 (-20.6 to -17.9) | -1.17 (-1.27 to -1.08) | -22.3 (-23.5 to -21.3) | -3.38 (-3.57 to -3.19) |
| South 24 Parganas | 51.9 (47.4-56.5) | 31.9 (27.0-37.8) | 27.1 (25.8-28.4) | -48.7 (-49.7 to -47.8) | -3.75 (-3.85 to -3.65) | -15.5 (-18.2 to -13.5) | -2.26 (-2.66 to -1.88) |
| Uttar Dinajpur | 50.5 (45.1-55.2) | 38.0 (33.9-42.3) | 33.6 (32.7-34.5) | -34.3 (-35.6 to -33.4) | -2.36 (-2.46 to -2.28) | -12.3 (-14.1 to -11.0) | -1.72 (-1.98 to -1.49) |
| **Tripura** | **46.5 (41.0-53.0)** | **35.2 (33.7-36.8)** | **29.0 (27.7-30.5)** | **-38.3 (-39.6 to -37.6)** | **-2.72 (-2.83 to -2.65)** | **-18.2 (-19.5 to -16.8)** | **-2.73 (-3.48 to -2.01)** |
| Dhalai | 42.5 (36.8-48.8) | 35.4 (29.8-41.1) | 28.1 (27.0-29.5) | -34.5 (-36.1 to -33.6) | -2.39 (-2.51 to -2.29) | -21.1 (-23.5 to -19.5) | -3.20 (-3.58 to -2.88) |
| Gomati | 42.8 (37.5-48.7) | 34.2 (30.0-38.7) | 28.7 (27.3-30.0) | -33.8 (-35.5 to -32.5) | -2.33 (-2.46 to -2.21) | -16.8 (-18.5 to -15.3) | -2.47 (-2.72 to -2.18) |
| Khowai | 48.2 (41.5-54.3) | 36.4 (31.0-41.9) | 26.7 (25.7-28.4) | -45.2 (-46.7 to -44.1) | -3.40 (-3.53 to -3.26) | -27.2 (-29.5 to -26.1) | -4.30 (-4.67 to -4.05) |
| North Tripura | 45.1 (38.4-51.8) | 36.9 (30.7-43.4) | 33.6 (32.3-34.9) | -26.2 (-28.1 to -25.1) | -1.71 (-1.84 to -1.61) | -9.3 (-12.4 to -7.0) | -1.29 (-1.71 to -0.88) |
| Sepahijala | 46.7 (40.7-52.5) | 34.1 (30.3-38.3) | 28.6 (27.5-30.1) | -39.6 (-41.3 to -38.8) | -2.85 (-2.99 to -2.75) | -16.8 (-18.9 to -15.2) | -2.48 (-2.77 to -2.17) |
| South Tripura | 45.8 (38.5-52.9) | 35.1 (30.2-40.0) | 32.0 (30.7-33.4) | -30.6 (-33.0 to -28.9) | -2.07 (-2.24 to -1.90) | -9.3 (-12.0 to -7.3) | -1.30 (-1.65 to -0.92) |
| Unakoti | 49.0 (42.5-56.1) | 37.6 (32.1-43.0) | 33.3 (32.1-34.5) | -32.8 (-34.4 to -31.3) | -2.24 (-2.37 to -2.10) | -12.0 (-14.4 to -10.5) | -1.69 (-2.03 to -1.42) |
| West Tripura | 48.8 (40.7-56.6) | 34.3 (28.6-41.2) | 25.8 (24.9-27.6) | -47.7 (-50.1 to -46.5) | -3.68 (-3.90 to -3.51) | -25.1 (-28.4 to -23.3) | -3.96 (-4.47 to -3.54) |
[truncated: 205,798 more chars]
